# Brønsted-Acid-Promoted Diaza-Nazarov Cyclization to Access Tetrasubstituted Pyrazoles

**DOI:** 10.1021/acs.orglett.5c03531

**Published:** 2025-09-21

**Authors:** U. Mert Karacaoğlu, Yunus E. Türkmen

**Affiliations:** † Department of Chemistry, Faculty of Science, 52948 Bilkent University, Ankara 06800, Türkiye; ‡ National Nanotechnology Research Center (UNAM), Institute of Materials Science and Nanotechnology, Bilkent University, Ankara 06800, Türkiye

## Abstract

We have developed a new diaza-Nazarov cyclization for the synthesis of densely substituted pyrazoles. Treatment of *N*-acylazo substrates with 1–1.5 equiv of trifluoroacetic acid (TFA) as a Brønsted acid promoter at 23 °C afforded hydroxypyrazole products in good to excellent yields (up to 99%). Reactions of substrates with secondary alkyl groups at the β position of the α,β-unsaturated carbonyl moiety gave minor amounts of dihydropyridazinone compounds as side products, proposed to form via an intriguing 6π electrocyclization.

## Graphical abstract

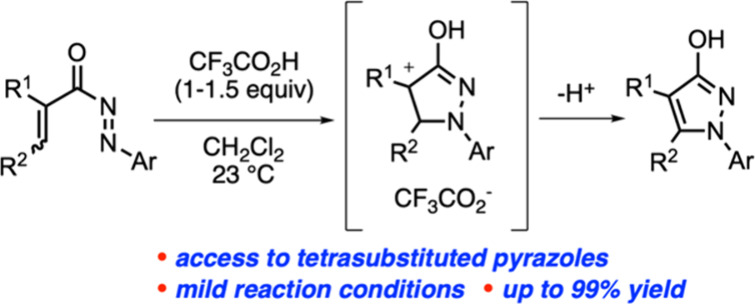

Whereas four- and six-membered rings can be readily constructed via a variety of (2 + 2) and (4 + 2) cycloadditions, access to five-membered carbocycles through analogous (3 + 2) cycloadditions remains comparatively limited.^1^ In this context, the Nazarov cyclization offers an efficient route to cyclopentenones and related analogues from acyclic five-carbon building blocks.^2^ Recently, the chemical space accessible via Nazarov-type reactions has expanded significantly with the development of new variants of this cyclization.^3,4^ These variants involve either modifications to the core five-atom framework of the cyclization precursors, as in the aza-Nazarov^5^ and oxa-Nazarov^6^ reactions, or changes to peripheral atoms, as seen in imino-^7^ and halo-Nazarov^8^ reactions. Pioneering work by Würthwein and co-workers on aza-Nazarov cyclizations led to the development of effective methods for the synthesis of *N*-alkoxy- and *N*-aminopyrroles.^5a,5c^ An inspiring study in this area was reported by Klumpp and co-workers in 2007, in which the aza-Nazarov reaction of *N*-acyliminium salts **1**, mediated by trifluoromethanesulfonic acid (TfOH), afforded cyclization products **2** (Scheme 1a).^9^ In 2019, Rasapalli and co-workers developed a TfOH-mediated aza-Nazarov cyclization of quinazolinonyl enones **3** to give compounds **4**, which were subsequently used as precursors to *C*-aryl luotonins and vasicinones (Scheme 1b).^10^

**Scheme 1:**
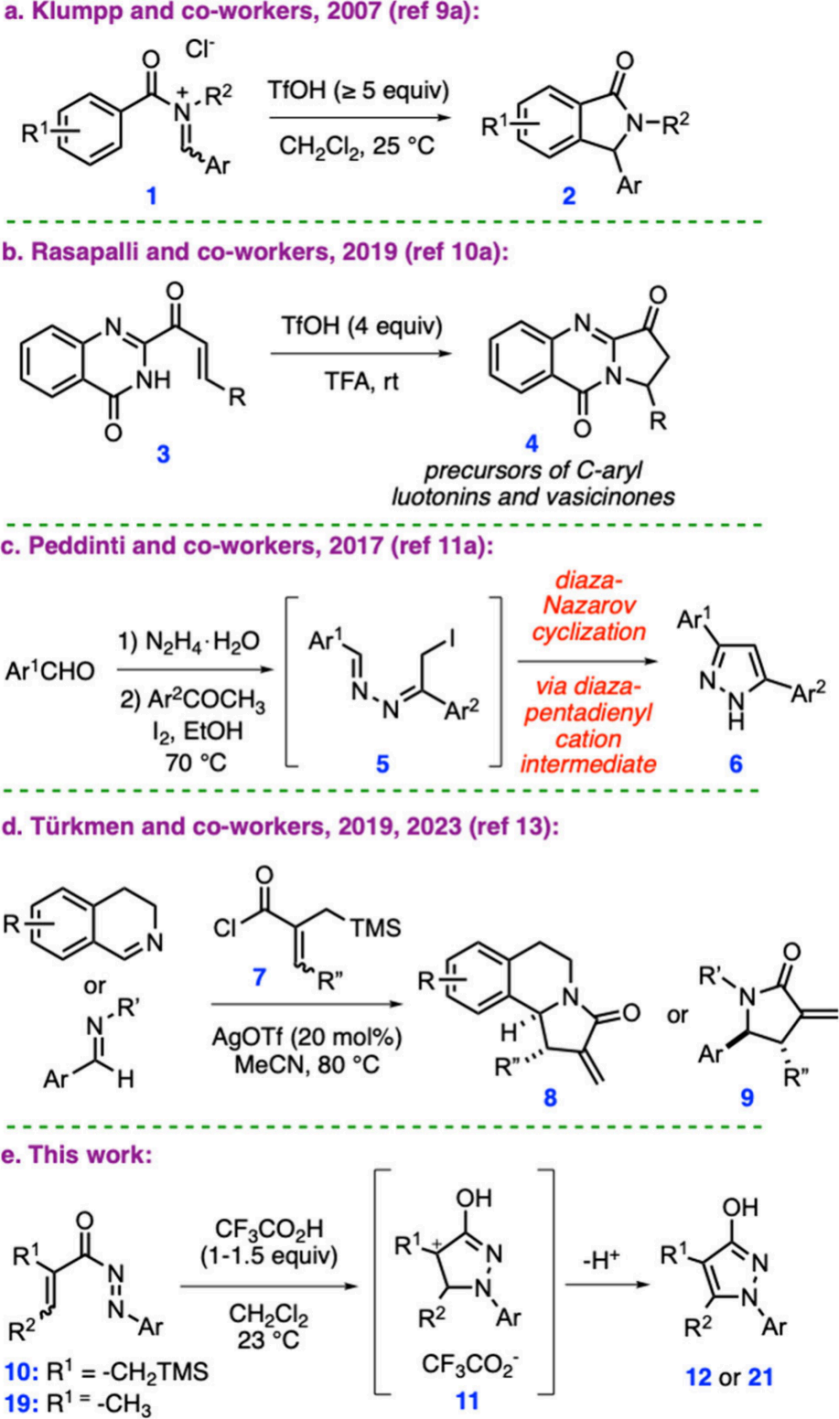
Examples of Aza-Nazarov and Diaza-Nazarov Reactions and Current Work

Despite notable advances in aza-Nazarov cyclization, variants involving more than one heteroatom in the five-atom framework of the substrates remain scarce. For instance, to our knowledge, only two examples of diaza-Nazarov cyclization have been reported, both by Peddinti and co-workers.^11^ In the first of these studies, the authors developed an I_2_-mediated diaza-Nazarov cyclization to obtain pyrazoles **6** via the intermediacy of iododiimine **5** (Scheme 1c).^11a^ In 2020, the same research group reported an iodine-catalyzed denitrative imino-diaza-Nazarov reaction, which furnished disubstituted pyrazoles.^11b^ An interesting tetraaza-Nazarov cyclization under oxidative conditions was developed by Piercey and co-workers in 2022, enabling the synthesis of nitrogen-rich molecules.^12^

Our group entered this research field in 2019, when we reported an aza-Nazarov cyclization that operates through anion-exchange catalysis.^13a^ In this reaction, 3,4-dihydroisoquinolines and α,β-unsaturated acyl chlorides **7** react to give *N*-acyliminium intermediates, which subsequently undergo an aza-Nazarov reaction in the presence of AgOTf as an anion-exchange reagent to afford tricyclic aza-Nazarov products **8** as single diastereomers (Scheme 1d).^13a^ We later examined the mechanistic details of this aza-Nazarov cyclization and extended the methodology to include acyclic imines as starting materials, thereby providing α-methylene-γ-lactam products **9** (Scheme 1d).^13b^ In these reactions, the presence and position of the trimethylsilyl (TMS) group were strategically designed to enable the required reactivity via the β-silicon effect^14^ imparted by the C–Si bond. In the current work, we opted to apply our strategy to substrates having two nitrogen atoms, aiming to achieve a novel diaza-Nazarov cyclization. To this end, we designed *N*-acylazo derivatives **10** as cyclization precursors, which feature an electron-deficient azo moiety as the electrophilic region and an allylsilane group as the nucleophilic region (Scheme 1e). Using such substrates, we developed an effective diaza-Nazarov cyclization, promoted by trifluoroacetic acid (TFA) as a Brønsted acid, to yield densely substituted pyrazole products. Please note that, although *N*-acylazo derivatives have previously been used in cycloaddition reactions, such as Diels–Alder reactions,^15^ they have not been employed in Nazarov-type cyclizations. Moreover, to the best of our knowledge, this work represents the first example of a diaza-Nazarov cyclization leading to hydroxypyrazoles, which, along with alkoxypyrazoles and pyrazolones, constitute an important scaffold in medicinal chemistry and drug discovery.^16^

To test our hypothesis on the targeted diaza-Nazarov cyclization, we first synthesized *N*-acylazo derivative **10aa** for use in the optimization studies (Scheme 2). After the completion of reaction optimization (*vide infra*), other cyclization precursors **10ab**–**10d** were prepared following the same reaction sequence. Previously known phosphonate **13** was prepared in a single step by the alkylation of commercially available triethyl phosphonoacetate with (iodomethyl)­trimethylsilane.^13^ The α,β-unsaturated esters **14a**–**14d** were prepared through the Horner–Wadsworth–Emmons reaction of **13** with various aldehydes under basic conditions in 75–90% yields. The hydrolysis of the ester groups was achieved by heating compounds **14a**–**14d** in a mixture of methanol and THF with excess KOH followed by acidic workup to afford carboxylic acids **15a**–**15d** in 67–97% yields. The subsequent hydrazide formation was found to proceed successfully via the coupling of the carboxylic acids with arylhydrazine derivatives using *N*,*N*′-dicyclohexylcarbodiimide (DCC) and 4-dimethylaminopyridine (DMAP) to give hydrazides **16aa**–**16d** in up to 81% yield. The final oxidation of hydrazides **16aa**–**16d** to *N*-acylazo products **10aa**–**10d** was accomplished via an initial N-bromination of hydrazides with NaH and *N*-bromosuccinimide (NBS) followed by the elimination of HBr under basic conditions (Scheme 2). This procedure proved to be effective for the aimed oxidation process, leading to the formation of *N*-acylazo derivatives **10aa**–**10d** in 57–86% yields. It should also be noted that most compounds in this reaction sequence were obtained as mixtures of *E* and *Z* isomers with varying diastereomeric ratios (dr). However, this is inconsequential, as both *E* and *Z* isomers of the *N*-acylazo compounds **10** give the same hydroxypyrazole products **12**, given that the final products contain no stereocenters.

**Scheme 2:**
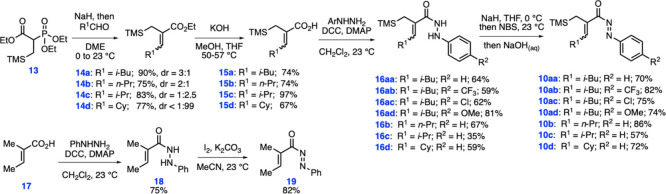
Syntheses of the *N*-Acylazo Derivatives **10** and **19**

With the cyclization precursors in hand, we next tested the targeted diaza-Nazarov cyclization of compound **10aa** in the presence of a variety of Brønsted and Lewis acids (Table 1). Surprisingly, the cyclization of **10aa** was found to proceed at 23 °C in CH_2_Cl_2_ even in the absence of an activating agent, albeit slowly, to give hydroxypyrazole product **12aa** in 24% yield after 24 h (entry 1). The time dependence of this transformation in CDCl_3_ was also monitored over a period of 12 days by ^1^H NMR spectroscopy, ultimately reaching a conversion value of 64% (Table S1 and Figure S1). When the reaction was run with 1.0 equiv of bis­(trifluoromethanesulfonyl)­imide (Tf_2_NH) as a strong Brønsted acid promoter, product **12aa** was isolated in 89% yield after 3 h (entry 2). The use of catalytic amounts of Tf_2_NH led to slightly lower product yields with prolonged reaction times (entries 3 and 4). To our delight, when 1.2 equiv of trifluoroacetic acid (TFA) was used as a milder and inexpensive Brønsted acid, diaza-Nazarov product **12aa** was obtained in 97% yield (entry 5). Lewis acids were also found to be effective promoters of this cyclization reaction. In particular, the use of BF_3_·OEt_2_ and ZnCl_2_ gave similarly high yields (88 and 93%, respectively; entries 6 and 7). We then turned our attention to testing other solvents. In this respect, heating a solution of **10aa** in CH_3_CN at 75 °C for 8 h afforded product **12aa** in 74% yield (entry 8). In order to see the effect of strong hydrogen-bonding solvents, reactions were also tested in 2,2,2-trifluoroethanol (TFE) and 1,1,1,3,3,3-hexafluoroisopropanol (HFIP).^17^ The p*K*_a_ values of TFE and HFIP were reported to be 12.8 and 9.3, respectively.^18^ Thus, the reaction efficiency was observed to correlate with the acidity and hydrogen-bond-donating ability of the solvent, where cyclization product **12aa** was isolated in 76% yield in TFE and 95% yield in HFIP (entries 9 and 10). Given its lower cost and the need for only stoichiometric amounts, unlike HFIP, which is used as a solvent, we selected TFA as the Brønsted acid for this cyclization.

**Table 1:**
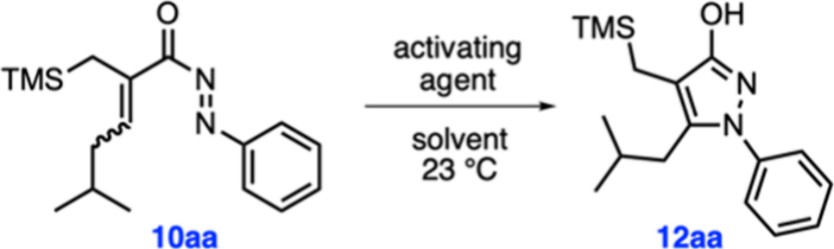
Optimization of the Diaza-Nazarov Cyclization of **10aa**

entry	activating agent (equiv)	solvent	time (h)	yield (%)^a^
1		CH_2_Cl_2_	24	24
2	Tf_2_NH (1.0)	CH_2_Cl_2_	3	89
3	Tf_2_NH (0.2)	CH_2_Cl_2_	23.5	78
4	Tf_2_NH (0.1)	CH_2_Cl_2_	30.5	56
5	TFA (1.2)	CH_2_Cl_2_	5	97
6	BF_3_·OEt_2_ (1.0)	CH_2_Cl_2_	2.75	88
7	ZnCl_2_ (1.0)	CH_2_Cl_2_	5	93
8^b^		CH_3_CN	8	74
9		TFE	2	76
10		HFIP	1	95

a Isolated product yield after purification by column chromatography.

b The reaction was run at 75 °C.

In the ^1^H NMR spectra of **12aa** in CDCl_3_, the −OH signal was occasionally observed, presumably depending on the concentrations of the samples. However, its ^1^H NMR spectrum in acetone-*d*_6_ exhibited a clear broad signal at 10.40 ppm (Figure S2), which disappeared when the spectrum was recorded again after the addition of a few drops of D_2_O (Figure S3), indicating the presence of an exchangeable proton. Another point that needs attention is the possibility of compound **12aa** to exist in different tautomeric forms. Indeed, such pyrazole derivatives can exist as hydroxypyrazole or pyrazolone tautomers.^19^ Examination of the ^13^C NMR spectra of various hydroxypyrazole and pyrazolone compounds reported in the literature shows that pyrazolones generally exhibit a signal at approximately 168–172 ppm, whereas hydroxypyrazoles display a signal around 162–164 ppm.^19,20^ Since all of the pyrazole products obtained in this work exhibit signals at 161–162 ppm in their ^13^C NMR spectra, we conclude that they exist predominantly in the hydroxypyrazole tautomeric form.

Following the completion of reaction optimization, we turned our attention to testing the applicability of these optimal conditions to other substrates (Scheme 3). As mentioned before, diaza-Nazarov cyclization product **12aa** was obtained in 97% yield under the optimized reaction conditions. The cyclization worked successfully with electron-withdrawing −CF_3_ and −Cl substituents on the phenyl ring of the *N*-acylazo moiety affording products **12ab** and **12ac** in 92 and 85% yields, respectively. We were pleased to see that hydroxypyrazole product **12ad** bearing the electron-rich *p*-methoxyphenyl group was isolated in an excellent yield (99%). These results indicate that electronic variations on the aryl ring exert minimal influence on reactivity. Next, we sought to investigate how the nature of the alkyl substituent at the β position of the cyclization precursor affects the reaction outcome. *n*-Propyl-substituted compound **10b** was found to be a competent substrate under the reaction conditions, leading to the formation of **12b** in 81% yield. Treatment of compound **10c**, substituted with the more sterically demanding isopropyl group at the β position, with TFA gave the expected diaza-Nazarov cyclization product **12c** in 74% yield along with the previously unencountered, six-membered dihydropyridazinone derivative **20a** as a side product (16% yield). In order to check the generality of this side product formation pathway, the reactivity of compound **10d** was examined under similar reaction conditions. When this reaction was run at 75 °C in CH_3_CN, the six-membered dihydropyridazinone side product **20b** was obtained in 19% yield in addition to the expected diaza-Nazarov side product **12d** (42%). At this point, we also sought to evaluate the reactivity of a *N*-acylazo derivative lacking a TMS group. For this purpose, we prepared compound **19** in two steps starting from the commercially available tiglic acid (**17**; Scheme 2). Gratifyingly, compound **19** was found to be reactive under the optimized reaction conditions, affording hydroxypyrazole product **21** in 97% yield (Scheme 3). This result demonstrates that the reactivity of the *N*-acylazo moiety is sufficiently high that the alkene group does not need to be electron-rich, such as in the form of an allylsilane. Finally, we intended to test a derivative of **10**, in which R^2^ is a phenyl group, as a substrate in the diaza-Nazarov cyclization. However, basic hydrolysis of Ph-substituted α,β-unsaturated ester **S1** led to both desilylation^21^ and ester hydrolysis, giving desilylated carboxylic acid **S2** (see the Supporting Information for details). Interestingly, our attempts to prepare the corresponding hydrazide using phenylhydrazine in the presence of DCC and DMAP resulted only in the formation of **S3**, the DCC adduct of **S2**, which was isolated and characterized.

**Scheme 3:**
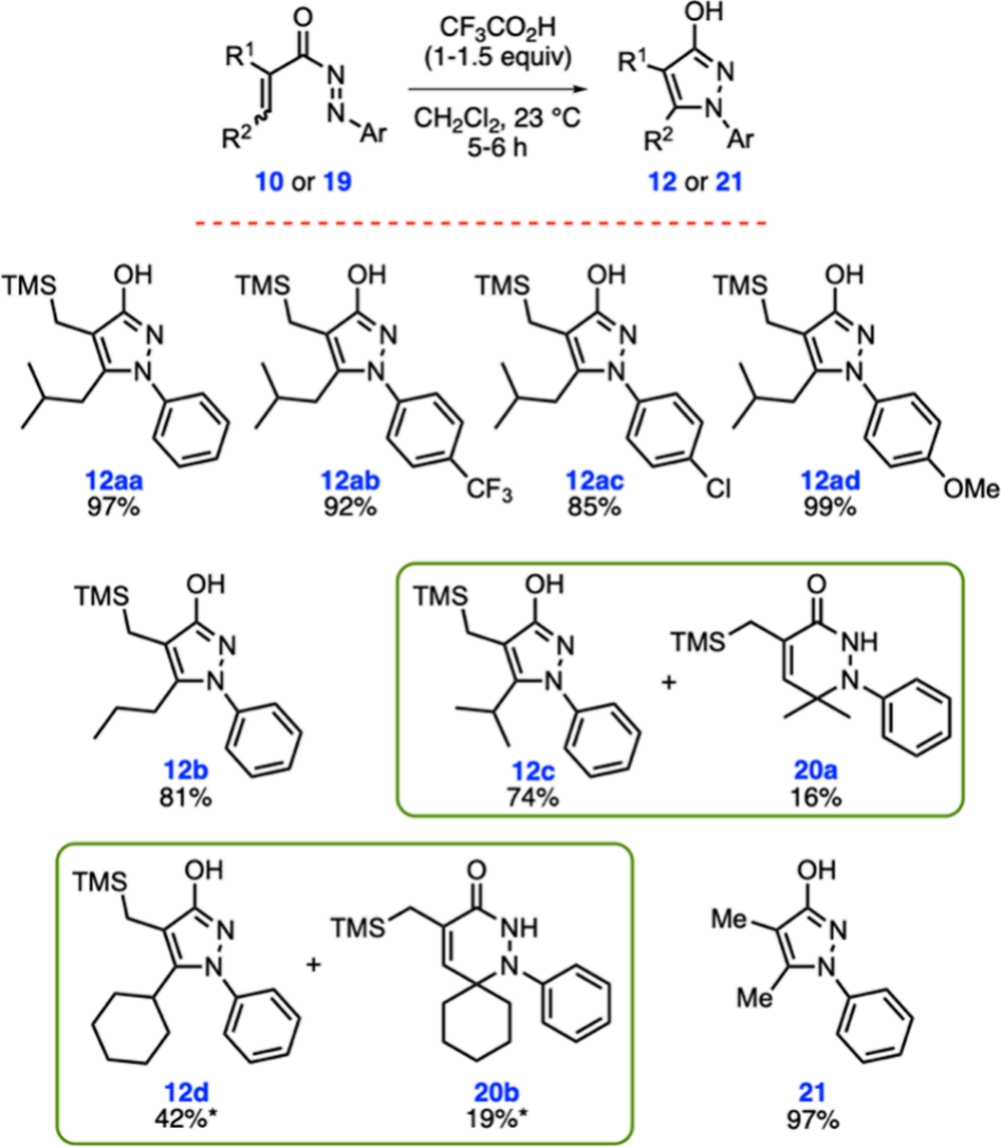
Substrate Scope Studies

* The reaction was run in MeCN at 75 °C.

Our proposed mechanisms for the formation of the diaza-Nazarov products **12** and the side product **20a** are presented in Scheme 4. Computation of the electrostatic potential map of a derivative of **10** revealed that the heteroatom with the most negative electrostatic potential is carbonyl oxygen (Figure S4), indicating that initial protonation of **10** with TFA is likely to occur at oxygen, leading to intermediate **22** (Scheme 4a). The 4π electrocyclization of **22** is expected to yield cyclic intermediate **11**, which, upon the loss of a proton, would give the final hydroxypyrazole product **12**. It should be noted that, in our previous aza-Nazarov reactions, the TMS group was lost in the final step to give an exocyclic double bond.^13^ However, in the current diaza-Nazarov cyclization, proton loss leading to the pyrazole product appears to be the dominant pathway, possibly driven by both aromatization and the increased acidity of the C–H proton adjacent to nitrogen and the carbocation. It is important to note that, in many reactions employing silyl-containing reactants, such as the Hosomi–Sakurai allylation^22^ or the Mukaiyama aldol reaction,^23^ the trialkylsilyl groups are essential for achieving the desired reactivity, although they are not incorporated into the final products. In this context, our reaction represents a rare example in which the TMS group is retained in the final product. Finally, although organosilicon compounds are not traditionally considered privileged scaffolds in medicinal chemistry, their significance in drug discovery is steadily growing.^24^

**Scheme 4:**
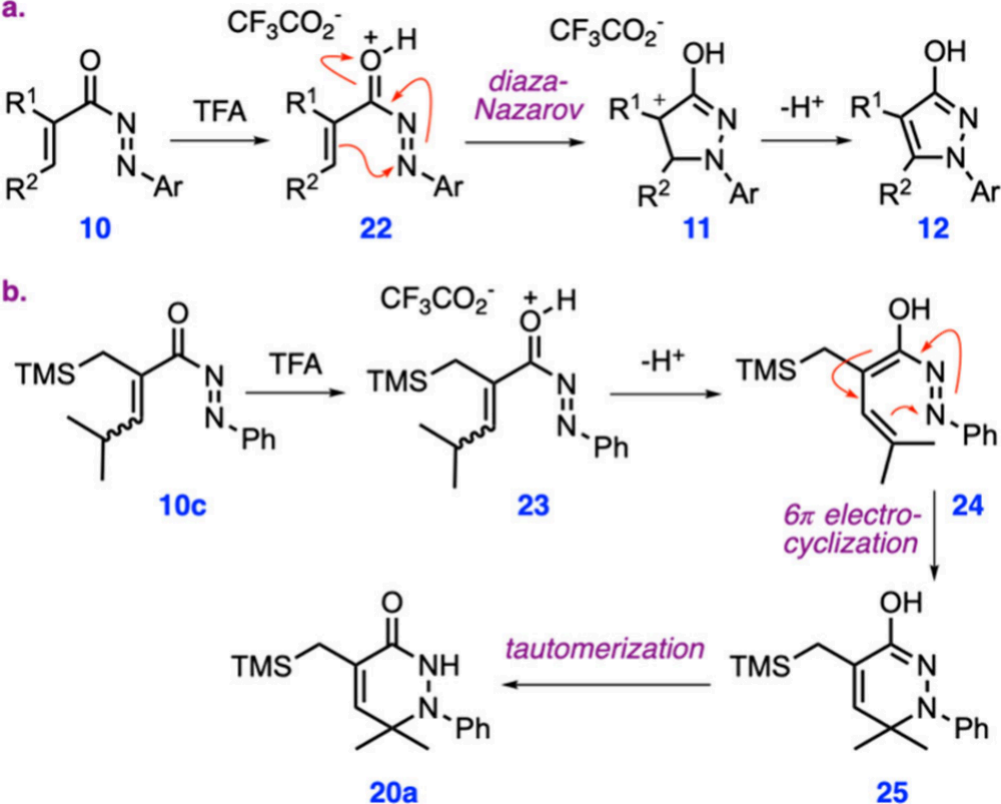
Proposed Mechanisms for the Formation of **12** and **20a**

When the cyclization precursor is substituted by a secondary alkyl group at the β position, as in the case of compounds **10c** and **10d**, dihydropyridazinone derivatives **20a** and **20b** were isolated as minor side products. Our proposed mechanism for the formation of these side products is shown in Scheme 4b. We reckon that an acid-catalyzed isomerization of **10c** may lead to azo-dienol intermediate **24**, through protonated intermediate **23**, which may be facilitated by the formation of a relatively stable, trisubstituted alkene. A subsequent 6π electrocyclization of **24** would give imidate **25**, which would then tautomerize to final product **20a**.

At the final stage of our studies, we first opted to check the scalability of the diaza-Nazarov cyclization. Pleasingly, when the cyclization of **10aa** was tested on a 1.2 mmol scale, the diaza-Nazarov product **12aa** was isolated in 88% yield (Scheme 5a). Next, we investigated the further functionalization of hydroxypyrazole **12aa**, motivated by the significance of alkoxypyrazoles in medicinal chemistry.^16a,16c^ To this end, first, compound **12aa** was O-methylated using K_2_CO_3_ and Me_2_SO_4_ to afford methoxypyrazole product **26** in 66% yield (Scheme 5b). Desilylation of **26** was then accomplished by heating its solution in THF in the presence of TBAF, leading to pyrazole **27** in 89% yield.

**Scheme 5:**
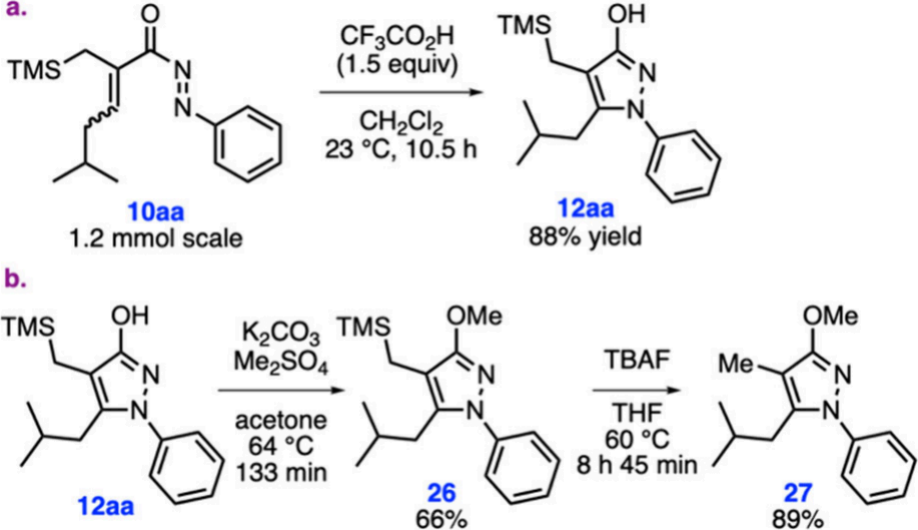
Scalability Experiment and Further Functionalization of Compound **12aa**

In conclusion, we have demonstrated that *N*-acylazo compounds undergo an efficient diaza-Nazarov cyclization to yield tetrasubstituted pyrazoles. The reaction proceeds at room temperature upon treatment of the reactants with 1–1.5 equiv of TFA, delivering a range of hydroxypyrazole products in yields of up to 99%. Interestingly, when substrates bearing secondary alkyl groups at the β position of alkene were used, side products with a dihydropyridazinone core were formed, presumably through a 6π-electrocyclization pathway. Ongoing studies are directed toward exploring other heterocyclic variants of the Nazarov cyclization.

## Supplementary Material



## Data Availability

The data underlying this study are available in the published article and its Supporting Information.

## Notes

Experimental procedures, characterization data, ^1^H, ^13^C­{^1^H}, and ^19^F NMR spectra, and HRMS data (PDF)
