# Supplementary material for: Brønsted-Acid-Promoted Diaza-Nazarov Cyclization to Access Tetrasubstituted Pyrazoles
Source: Org Lett. 2025 Sep 21;27(39):11094–9. doi: 10.1021/acs.orglett.5c03531 (PMC12501944; doi:10.1021/acs.orglett.5c03531)
Supplement: Supplementary file 1 [file ol5c03531_si_001.pdf]

Brønsted-Acid-Promoted Diaza-Nazarov Cyclization to Access Tetrasubstituted Pyrazoles

Supporting Information

U. Mert Karacaoğlu[1] and Yunus E. Türkmen*[1,2]

[1]*Department of Chemistry, Faculty of Science, Bilkent University, Ankara, 06800, Türkiye*

[2]*UNAM, National Nanotechnology Research Center, Institute of Materials Science and Nanotechnology, Bilkent University, Ankara, 06800, Türkiye*

* Corresponding author

Table of Contents

S-2 General Information and Materials
S-3 Syntheses of Esters **14** and **S1**
S-7 Syntheses of Carboxylic Acids **15** and **S2**
S-10 Syntheses of Hydrazides **16** and **18**
S-24 Syntheses of the Pyrazole Products **12** and **21**
S-32 Monitoring the Conversion of **10aa** to **12aa** in the Absence of an Acid by ^{1}H NMR Spectroscopy
S-34 ^{1}H NMR Spectra of **12aa** in Acetone-d_6 Before and After the Addition of D_2O
S-35 Electrostatic Potential Map of a Model *N*-Acylazo Derivative
S-36 References
S-37 ^{1}H, ^{13}C{^{1}H} and ^{19}F NMR spectra
S-114 HRMS Data

General Information: All air-sensitive reactions were performed using oven-dried glassware under an inert atmosphere of nitrogen. Reactions were monitored by TLC (thin-layer chromatography) using aluminum-backed plates pre-coated with silica gel (Silicycle, Silica Gel 60 F_{254}). UV light (254 nm) and/or basic $KMnO_4$ staining solutions were used for TLC visualization. Flash column chromatography was performed on Silicycle 40-63 μm (230-400 mesh) flash silica gel. NMR spectra were measured on a Bruker spectrometer at 400 MHz for ^{1}H NMR, 100 MHz for $^{13}C\{^{1}H\}$ NMR, and 376 MHz for ^{19}F NMR spectra. The spectra were calibrated from internal standard (TMS, 0 ppm) or residual solvent signals (chloroform at 7.26 ppm, DMSO at 2.50 ppm, and acetone at 2.05 ppm for ^{1}H NMR spectra, and chloroform at 77.16 ppm and DMSO at 39.52 ppm for $^{13}C\{^{1}H\}$ spectra). TFA (trifluoroacetic acid) was used as external reference (-76.55 ppm) for ^{19}F NMR spectral measurements. ^{1}H-NMR data are reported as follows: chemical shift (parts per million, ppm), integration, multiplicity (s = singlet, d = doublet, t = triplet, q = quartert, sext = sextet, sept = septet, n = nonet, dd = doublet of doublets, tt = triplet of triplets, m = multiplet, br = broad, app = apparent), coupling constant (Hz). Infrared (FTIR) spectra were recorded on a Bruker Alpha-Platinum-ATR spectrometer with only selected peaks reported. Mass spectral analyses were performed at UNAM-National Nanotechnology Research Center and Institute of Materials Science and Nanotechnology, Bilkent University, and DAYTAM-East Anatolia High Technology Application and Research Center, Atatürk University.

Materials: Anhydrous CH_3CN was obtained by distillation over P_2O_5 under an inert atmosphere of nitrogen. Anhydrous DME (1,2-dimethoxyethane) was obtained by distillation over CaH_2 under an inert atmosphere of nitrogen. Anhydrous THF and CH_2Cl_2 were purchased from Thermo Scientific (AcroSeal®) and used as received. NBS (*N*-bromosuccinimide) was recrystallized from water, dried thoroughly under vacuum, and stored in the refrigerator in an amber bottle. All other commercially available reagents were used as received unless stated otherwise.

Syntheses of Esters 14 and S1:

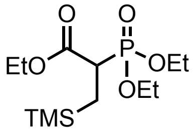

13

In an oven-dried 100-mL two-necked round bottomed flask, after 3 cycles of vacuum-N_2, triethyl phosphonoacetate (3.5 mL, 17.6 mmol, 1.0 equiv.) was dissolved in 10 mL of anhydrous DME, then the flask was immersed in an ice bath. NaH (704 mg, 17.6 mmol, 1.0 equiv., 60% dispersion in paraffin liquid) was added portion-wise, and vigorous H_2 gas evolution was observed. The reaction mixture was stirred for 30 min, then, $TMSCH_2I$ (3.0 mL, 20.1 mmol, 1.14 equiv.) was added and the flask was immersed in an oil bath at 70 °C. After stirring the mixture at this temperature for 2 h, the flask was taken out of the oil bath, and 8 mL of $NH_4Cl_{(satd.)}$ was added. The aqueous layer was extracted thrice with EtOAc. The organic layers were combined, dried over anhydrous Na_2SO_4, and concentrated *in vacuo*. The crude product was purified with flash column chromatography using a solvent system of 1:1 EtOAc:hexanes → pure EtOAc. Compound **13** was obtained as a colourless oil (4.26 g, 78% yield). The ^{1}H NMR spectrum of this compound is in agreement with the literature.[1,2]

^{1}H NMR (400 MHz; $CDCl_3$) δ: 4.18-4.08 (6H, m), 2.92 (1H, ddd, J = 22.5, 12.7, 2.4 Hz), 1.30 (6H, t, J = 7.4 Hz), 1.26 (3H, t, J = 7.1 Hz), 1.29-1.23 (1H, m), 1.05-0.97 (1H, m), -0.02 (9H, s).

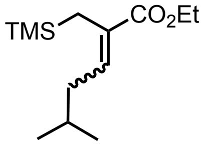

14a

In an oven-dried 50-mL round bottomed flask, after 3 cycles of vacuum-N_2, compound **13** (835 mg, 2.69 mmol, 1.0 equiv.) was dissolved in 10 mL of anhydrous DME, and the flask was immersed in an ice bath to bring the solution to 0 °C. NaH (161 mg, 4.04 mmol, 1.5 equiv., 60% dispersion in paraffin liquid) was introduced portion-wise. After the bubbling settled down, the ice bath was removed, and the mixture was brought to room temperature. It was stirred for 30 min, then isovaleraldehyde (0.40 mL, 4.04 mmol, 1.5 equiv.) was added. The reaction mixture was stirred for 30 min, then 7 mL of $NH_4Cl_{(satd.)}$ was added. The aqueous layer was extracted thrice with CH_2Cl_2. The organic layers were combined, dried over anhydrous

Na_2SO_4, and concentrated *in vacuo*. The crude product was purified with flash column chromatography using a solvent system of 1:100 EtOAc:hexanes. Compound **14a** was obtained as a colourless oil (589 mg, 90% yield, dr (*Z:E*) = 3:1). The ^{1}H NMR spectrum of this compound is in agreement with the literature.[1]

^{1}H NMR (400 MHz; $CDCl_3$) δ: 6.64 (1H, t, *J* = 7.3 Hz), 4.17 (2H, q, *J* = 7.2 Hz), 1.98 (2H, t, *J* = 7.1 Hz), 1.80 (2H, s), 1.77-1.69 (1H, m), 1.29 (3H, t, *J* = 7.1 Hz), 0.93 (6H, d, *J* = 6.6 Hz), -0.01 (9H, s).

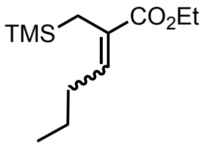

14b

In an oven-dried 50-mL two-necked round bottomed flask, after 3 cycles of vacuum-N_2, compound **13** (696 mg, 2.24 mmol, 1.0 equiv.) was dissolved in 10 mL of anhydrous DME. The flask was immersed in an ice bath, then NaH (135 mg, 3.37 mmol, 1.5 equiv., 60% dispersion in paraffin liquid) was added portion-wise. After 30 min of stirring, the flask was taken out of the ice bath, and butyraldehyde (0.30 mL, 3.37 mmol, 1.5 equiv.) was added. The reaction mixture was stirred at 23 °C for 1.5 h. Then, 0.1 mL of distilled water was added to dissolve the thick yellow phosphate salt in order to free the stir bar. Then, 6 mL of $NH_4Cl_{(satd.)}$ was added as aqueous work-up. The aqueous layer was extracted thrice with EtOAc. The organic layers were combined, dried over anhydrous Na_2SO_4, and concentrated *in vacuo*. The crude product was purified with flash column chromatography using a solvent system of 1:9 EtOAc:hexanes. Compound **14b** was obtained as a colourless oil (385 mg, 75% yield, dr (*Z:E*) = 2:1). The ^{1}H NMR spectrum of this compound is in agreement with the literature.[1]

^{1}H NMR (400 MHz; $CDCl_3$) δ: 6.58 (1H, t, *J* =7.3 Hz), 4.14 (3H, q, *J* = 7.1 Hz), 2.04 (2H, q, *J* = 7.4 Hz), 1.78 (2H, s), 1.48-1.34 (2H, m), 1.26 (3H, t, *J* = 7.1 Hz), 0.91 (3H, t, *J* = 7.4 Hz), -0.03 (9H, s).

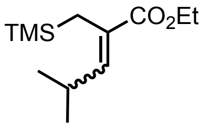

14c

In an oven-dried 50-mL two-necked round bottom flask, after 3 cycles of vacuum-N_2, compound **13** (801 mg, 2.58 mmol, 1.0 equiv.) was dissolved in 6 mL of anhydrous DME. The

flask was immersed in an ice bath, then NaH (155 mg, 3.87 mmol, 1.5 equiv., 60% dispersion in paraffin liquid) was added portion-wise. After 30 min of stirring, the flask was taken out of the ice bath, and isobutyraldehyde (0.35 mL, 3.87 mmol, 1.5 equiv.) was added. The reaction mixture was stirred at 23 °C for 80 min. Then, 0.1 mL of distilled water was added to dissolve the thick yellow phosphate salt in order to free the stir bar. Then, 4 mL of $NH_4Cl_{(satd.)}$ was added as aqueous work-up. The aqueous layer was extracted thrice with EtOAc. The organic layers were combined, dried over anhydrous Na_2SO_4, and concentrated *in vacuo*. The crude product was purified with flash column chromatography using a solvent system of 1:19 EtOAc:hexanes. Compound **14c** was obtained as a colourless oil (488 mg, 83% yield, dr (*Z:E*) = 1:2.5).

R_f = 0.47 (1:19 EtOAc: hexanes)
TLC Visualization: Visible under 254 nm UV light; stains with basic $KMnO_4$ solution.
^{1}H NMR (400 MHz; $CDCl_3$) δ; major diastereomer (*E*): 5.41 (1H, d, *J* = 9.7 Hz), 4.15 (2H, q, *J* = 7.1 Hz), 3.16-3.04 (1H, m), 1.68 (2H, d, *J* = 0.9 Hz), 1.28 (3H, t, *J* = 7.1 Hz), 0.96 (6H, d, *J* = 6.6 Hz), 0.04 (9H, s).
minor diastereomer (*Z*): 6.39 (1H, d, *J* = 10.1 Hz), 4.15 (2H, q, *J* = 7.2 Hz), 2.55-2.43 (1H, m), 1.78 (2H, d, *J* = 0.7 Hz), 1.27 (3H, t, *J* = 7.1 Hz), 0.99 (6H, d, *J* = 6.6 Hz), -0.01 (9H, s).
^{13}C{^{1}H} NMR (100 MHz; $CDCl_3$) δ; major diastereomer (*E*): 168.7, 145.9, 127.2, 60.1, 28.7, 23.1, 22.2, 14.4, -1.6.
minor diastereomer (*Z*): 168.8, 145.1, 127.9, 60.5, 28.1, 24.1, 17.1, 14.4, -1.0.
FTIR ν_{max} (ATR, film)/cm^{-1} 2956, 2925, 2869, 1712, 1635, 1465, 1372, 1305.
HRMS (APCI+) Calculated for: $C_{12}H_{24}O_2Si$ $[M+H]^+$: 229.1618, found: 229.1617.

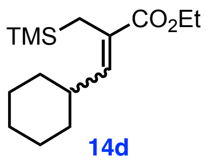

14d

In an oven-dried 50-mL two-necked round bottomed flask, after 3 cycles of vacuum-N_2, compound **13** (413 mg, 1.33 mmol, 1.0 equiv.) was dissolved in 2.6 mL of anhydrous DME. The flask was immersed in an ice bath, then NaH (79.7 mg, 1.99 mmol, 1.5 equiv., 60% dispersion in paraffin liquid) was added portion-wise. After 30 min of stirring, the flask was taken out of the ice bath, and cyclohexanecarboxaldehyde (0.24 mL, 1.99 mmol, 1.5 equiv.) was added. The reaction mixture was stirred at 23 °C for 1 h. Then, 3 mL of $NH_4Cl_{(satd.)}$ was

added as aqueous work-up. The aqueous layer was extracted thrice with EtOAc. The organic layers were combined, dried over anhydrous Na_2SO_4, and concentrated *in vacuo*. The crude product was purified with flash column chromatography using a solvent system of pure hexanes → 1:19 EtOAc:hexanes. Compound **14d** was obtained as a colourless oil (276 mg, 77% yield, dr (*E:Z*) > 99:1).

***R**$_f$* = 0.61 (1:19 EtOAc: hexanes)

TLC Visualization: Visible under 254 nm UV light; stains with basic $KMnO_4$ solution.

^{1}H NMR (400 MHz; $CDCl_3$) δ: 5.46 (1H, d, *J* = 9.6 Hz), 4.17 (2H, q, *J* = 7.1 Hz), 2.86-2.76 (1H, m), 1.70 (2H, s), 1.70 (5H, br s), 1.35-1.25 (2H, br s), 1.30 (3H, t, *J* = 7.1 Hz), 1.17 (1H, app tt, *J* = 12.3, 3.2 Hz), 1.08-1.01 (2H, m), -0.03 (9H, s).

^{13}C{^{1}H} NMR (100 MHz; $CDCl_3$) δ: 168.7, 144.7, 127.5, 60.1, 38.5, 33.4, 26.2, 25.9, 24.1, 14.4, -1.6.

FTIR ν_{max} (ATR, film)/cm^{-1} 2925, 2851, 1714, 1448, 1404.

HRMS (APCI+) Calculated for: $C_{15}H_{28}O_2Si$ $[M+H]^+$: 269.1931, found: 269.1930.

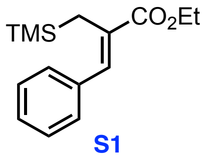

S1

In an oven-dried 50-mL two-necked round bottomed flask, after 3 cycles of vacuum-N_2, compound **13** (407 mg, 1.31 mmol, 1.0 equiv.) was dissolved in 4 mL of anhydrous DME. The flask was immersed in an ice bath, then NaH (78.8 mg, 1.97 mmol, 1.5 equiv., 60% dispersion in paraffin liquid) was added portion-wise. After 30 min of stirring, the flask was taken out of the ice bath, and benzaldehyde (0.20 mL, 1.97 mmol, 1.5 equiv.) was added. The reaction mixture was stirred at 23 °C for 1 h. Then, 3 mL of $NH_4Cl_{(satd.)}$ was added as aqueous work-up. The aqueous layer was extracted thrice with EtOAc. The organic layers were combined, dried over anhydrous Na_2SO_4, and concentrated *in vacuo*. The crude product was purified with flash column chromatography using a solvent system of 1:19 EtOAc:hexanes. Compound **S1** was obtained as a colourless oil (258 mg, 75% yield, dr (*Z:E*) > 99:1).

***R**$_f$* = 0.59 (1:19 EtOAc: hexanes)

TLC Visualization: UV active; stains with $KMnO_4$ solution.

^{1}H NMR (400 MHz; CDCl$_3$) δ: 7.57 (1H, s), 7.43-7.37 (4H, m), 7.32-7.28 (1H, m), 4.29 (2H, q, *J* = 7.2 Hz), 2.22 (2H, d, *J* = 0.7 Hz), 1.38 (3H, t, *J* = 7.1 Hz), 0.02 (9H, s).
^{13}C{^{1}H} NMR (100 MHz; CDCl$_3$) δ: 169.1, 136.8, 134.9, 132.0, 129.3, 128.4, 127.9, 61.0, 17.9, 14.5, -0.7.
FTIR ν_{max} (ATR, film)/cm^{-1} 2983, 1708, 1447, 1369, 1251.
HRMS (APCI+) Calculated for: $C_{15}H_{23}O_2Si$ $[M+H]^+$: 263.1462, found: 263.1464.

Syntheses of Carboxylic Acids 15 and S2:

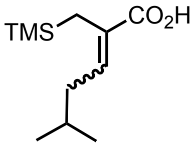

15a

In a 50-mL round bottomed flask, after 3 cycles of vacuum-N_2, compound **14a** (293 mg, 1.21 mmol, 1.0 equiv.) was dissolved in 3 mL of MeOH and 6 mL of THF, and then $KOH_{(aq)}$ (5.0 M, 6.4 mL, 19.0 equiv.) was added. The flask was immersed in an oil bath at 60 °C, and the reaction mixture was stirred for 25.5 h. Then, the flask was brought to room temperature and immersed in an ice bath. $HCl_{(conc.)}$ was added until the pH was 0-1. The aqueous layer was extracted five times with EtOAc. The organic layers were combined, dried over anhydrous Na_2SO_4, and concentrated *in vacuo*. The crude product was purified with flash column chromatography using a solvent system of 1:5 EtOAc:hexanes. Compound **15a** was obtained as a colourless oil (192 mg, 74% yield, dr (*Z:E*) = 3:1). The ^{1}H NMR spectrum of this compound is in agreement with the literature.[1]

^{1}H NMR (400 MHz; CDCl$_3$) δ: 12.03 (1H, bs), 6.82 (1H, t, *J* = 7.3 Hz), 2.02 (2H, t, *J* = 7.1 Hz), 1.80 (2H, s), 1.78-1.70 (1H, m), 0.94 (6H, d, J = 6.7 Hz), 0.02 (9H, s).

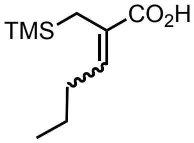

15b

In a 50-mL round bottomed flask, after 3 cycles of vacuum-N_2, compound **14b** (385 mg, 1.69 mmol, 1.0 equiv.) was dissolved in 3 mL of MeOH and 6 mL of THF, and then $KOH_{(aq)}$ (5.0 M, 4.6 mL, 19.0 equiv.) was added. The flask was immersed in an oil bath at 60 °C, and the

reaction mixture was stirred for 46.5 h. Then, the flask was brought to room temperature and immersed in an ice bath. $HCl_{(conc.)}$ was added until the pH was 0-1. The aqueous layer was extracted thrice with EtOAc. The organic layers were combined, dried over anhydrous Na_2SO_4, and concentrated *in vacuo*. The crude product was purified with flash column chromatography using a solvent system of 1:5 EtOAc:hexanes. Compound **15b** was obtained as a brownish oil (251 mg, 74% yield, dr (Z:*E*) = 2.5:1). The ^{1}H NMR spectrum of this compound is in agreement with the literature.[1]

^{1}H NMR (400 MHz; $CDCl_3$) δ: 11.49 (1H, br s), 6.78 (1H, t, *J* = 7.3 Hz), 2.11 (2H, q, *J* = 7.4 Hz), 1.80 (2H, s), 1.52-1.41 (2H, m), 0.95 (3H, t, *J* = 7.4 Hz), 0.02 (9H, s).

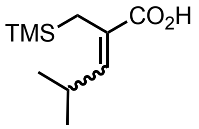

15c

In a 50-mL round bottomed flask, after 3 cycles of vacuum-N_2, compound **14c** (485 mg, 2.12 mmol, 1.0 equiv.) was dissolved in 4 mL of MeOH and 8 mL of THF, and then $KOH_{(aq)}$ (10.0 M, 4.0 mL, 19.0 equiv.) was added. The flask was immersed in an oil bath at 60 °C, and the reaction mixture was stirred for 43 h. Then, the flask was brought to room temperature and immersed in an ice bath. $HCl_{(conc.)}$ was added until the pH was 0-1. The aqueous layer was extracted thrice with EtOAc. The organic layers were combined, dried over anhydrous Na_2SO_4, and concentrated *in vacuo*. The product was purified with flash column chromatography using a solvent system of 1:9 → 1:5 EtOAc:hexanes. Compound **15c** was obtained as a brownish oil (411 mg, 97% yield, dr (*Z*:*E*) = 1:3).

R_f = 0.47 (1:19 EtOAc: hexanes)
TLC Visualization: Visible under 254 nm UV light; stains with basic $KMnO_4$ solution.
^{1}H NMR (400 MHz; $CDCl_3$) δ; major diastereomer (*E*): 10.19 (1H, br s), 5.61 (1H, d, *J* = 9.9 Hz), 3.37-3.24 (1H, m), 1.72 (2H, s), 0.99 (6H, d, *J* = 6.6 Hz), 0.01 (9H, s).
minor diastereomer (*Z*): 10.19 (1H, br s), 6.58 (1H, d, *J* = 10.2 Hz), 2.60-2.47 (1H, m), 1.80 (2H, s), 1.02 (6H, d, *J* = 6.6 Hz), 0.03 (9H, s).
^{13}C{^{1}H} NMR (100 MHz; $CDCl_3$) δ; major diastereomer (*E*): 174.5, 149.5, 126.3, 28.8, 23.1, 22.0, -1.6.
minor diastereomer (*Z*): 174.6, 147.9, 127.3, 28.4, 23.8, 16.8, -1.0.

FTIR ν_{max} (ATR, film)/cm^{-1} 2959, 2904, 2869, 1684, 1627, 1421, 1259.

HRMS (ESI-) Calculated for: $C_{10}H_{19}O_2Si$ [M-H]$^-$: 199.1160, found: 199.1160.

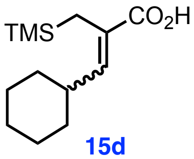

15d

In a 50-mL round bottomed flask, after 3 cycles of vacuum-N_2, compound **14d** (277 mg, 1.03 mmol, 1.0 equiv.) was dissolved in 3 mL of MeOH and 6 mL of THF, and then $KOH_{(aq)}$ (5.0 M, 3.9 mL, 19.0 equiv.) was added. The flask was immersed in an oil bath at 60 °C, and the reaction mixture was stirred for 45 h. Then, the flask was brought to room temperature and immersed in an ice bath. $HCl_{(conc.)}$ was added until the pH was 0-1. The aqueous layer was extracted thrice with EtOAc. The organic layers were combined, dried over anhydrous Na_2SO_4, and concentrated *in vacuo*. The crude product was purified with flash column chromatography using a solvent system of 1:9 → 1:5 → 1:1 EtOAc:hexanes → pure EtOAc. Compound **15d** was obtained as a white oil with yellowish tint, which solidified upon standing in the refrigerator (165 mg, 67% yield, dr (*Z*:*E*) = 1:6).

$\boldsymbol{R_f}$ = 0.23 (1:9 EtOAc: hexanes)

TLC Visualization: Visible under 254 nm UV light; stains with basic $KMnO_4$ solution.

^{1}H NMR (400 MHz; $CDCl_3$) δ: 12.14 (1H, br s), 5.63 (1H, d, *J* = 9.8 Hz), 3.03-2.94 (1H, m), 1.72 (2H, s), 1.72-1.65 (5H, br s), 1.38-0.96 (5H, m), 0.00 (9H, s).

^{13}C{^{1}H} NMR (100 MHz; $CDCl_3$) δ: 174.5, 148.0, 126.5, 38.4, 33.3, 26.2, 25.8, 23.9, -1.6.

FTIR ν_{max} (ATR, film)/cm^{-1} 2925, 2852, 1683, 1627, 1448, 1422.

HRMS (APCI-) Calculated for: $C_{13}H_{23}O_2Si$ [M-H]$^-$: 239.1473, found: 239.1473.

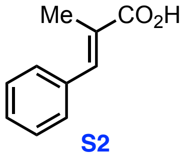

S2

In a 50-mL round bottomed flask, after 3 cycles of vacuum-N_2, compound **S1** (245 mg, 0.93 mmol, 1.0 equiv.) was dissolved in 3 mL of MeOH and 6 mL of THF, and then $KOH_{(aq)}$ (5.0 M, 3.5 mL, 19.0 equiv.) was added. The flask was immersed in an oil bath at 60 °C, and the reaction mixture was stirred for 46 h. Then, the flask was brought to room temperature and immersed in an ice bath. $HCl_{(conc.)}$ was added until the pH was 0-1. The aqueous layer was

extracted thrice with EtOAc. The organic layers were combined, dried over anhydrous Na_2SO_4, and concentrated *in vacuo*. The crude product was purified with flash column chromatography using a solvent system of 1:5 → 1:3 → 1:1 EtOAc:hexanes → pure EtOAc. Compound **S2** was obtained as a beige oil, which solidified upon standing in the refrigerator (138 mg, 85% purity, ca. 78% yield). The ^{1}H NMR spectrum of this compound is in agreement with the literature.[3]

^{1}H NMR (400 MHz; $CDCl_3$) δ: 12.16 (1H, br s), 7.88 (1H, d, J = 1.1 Hz), 7.49-7.42 (4H, m), 7.40-7.34 (1H, m), 2.19 (3H, d, J = 1.4 Hz).

Syntheses of Hydrazides 16 and 18:

General Procedure A:

The carboxylic acid derivative (1.0 equiv.) was dissolved in anhydrous CH_2Cl_2, and the phenylhydrazine derivative (1.5 equiv.), DCC (1.5 equiv.), and DMAP (0.28 equiv.) were added. The reaction mixture was stirred overnight, and 4 mL of distilled water was added as aqueous work-up. The aqueous layer was extracted thrice with EtOAc or CH_2Cl_2. The organic layers were combined, dried over anhydrous Na_2SO_4, and concentrated *in vacuo*. The arylhydrazide product was purified with flash column chromatography.

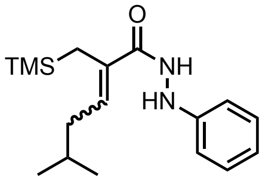

16aa

Following **General Procedure A**, the reaction was carried out using the carboxylic acid derivative **15a** (266 mg, 1.24 mmol, 1.0 equiv.), phenylhydrazine (0.18 mL, 1.86 mmol, 1.5 equiv.), DCC (384 mg, 1.86 mmol, 1.5 equiv.), DMAP (42.8 mg, 0.35 mmol, 0.28 equiv.), and 3.5 mL of anhydrous CH_2Cl_2. The crude product was purified with flash column chromatography using a solvent system of 1:19 → 1:9 → 1:5 → 1:3 EtOAc:hexanes. Compound **16aa** was obtained as an orange solid (243 mg, 64% yield, dr (Z:E) > 99:1).

R_f = 0.43 (1:5 EtOAc: hexanes)

TLC Visualization: Visible under 254 nm UV light; stains with basic $KMnO_4$ solution.

^{1}H NMR (400 MHz; CDCl$_3$) δ: 7.67 (1H, br s), 7.22 (2H, dd, J = 8.4, 7.4 Hz), 6.89 (1H, t, J = 8.0 Hz), 6.85 (2H, d, J = 7.3 Hz), 6.30 (1H, br s), 6.13 (1H, t, J = 7.0 Hz), 1.99 (2H, t, J = 7.0 Hz), 1.85 (2H, s), 1.72 (1H, n, J = 6.8 Hz), 0.95 (6H, d, J = 6.6 Hz), 0.02 (9H, s).

^{13}C{^{1}H} NMR (100 MHz; CDCl$_3$) δ: 170.6, 148.5, 134.1, 131.3, 129.2, 121.4, 114.0, 37.9, 28.6, 22.7, 18.2, -0.9.

FTIR ν_{max} (ATR, film)/cm^{-1} 3241 (br), 2952, 1652, 1626, 1599, 1530, 1493.

HRMS (APCI+) Calculated for: $C_{17}H_{29}N_2OSi$ [M+H]$^+$: 305.2044, found: 305.2041.

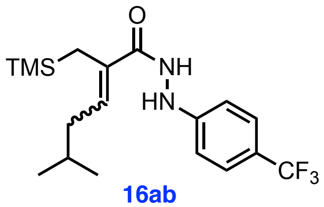

16ab

Following **General Procedure A**, the reaction was carried out using the carboxylic acid derivative **15a** (55 mg, 0.25 mmol, 1.0 equiv.), 4-(trifluoromethyl)phenylhydrazine (67 mg, 0.38 mmol, 1.5 equiv.), DCC (78 mg, 0.38 mmol, 1.5 equiv.), DMAP (8.6 mg, 0.070 mmol, 0.28 equiv.) and 2 mL of anhydrous CH_2Cl_2. The crudpe roduct was purified with flash column chromatography using a solvent system of 1:19 → 1:9 → 1:5 EtOAc:hexanes. Compound **16ab** was obtained as an orange solid (54.6 mg, 59% yield, dr (*Z*:*E*) > 99:1).

R$_f$ = 0.31 (1:5 EtOAc: hexanes)

TLC Visualization: Visible under 254 nm UV light; stains with basic $KMnO_4$ solution.

^{1}H NMR (400 MHz; CDCl$_3$) δ: 7.58 (1H, br d, J = 2.7 Hz), 7.44 (2H, d, J = 8.6 Hz), 6.87 (2H, d, J = 8.5 Hz), 6.55 (1H, br d, J = 2.7 Hz), 6.18 (1H, t, J = 7.1 Hz), 2.01 (2H, t, J = 7.0 Hz), 1.86 (2H, s), 1.73 (1H, n, J = 6.8 Hz), 0.96 (6H, d, J = 6.7 Hz), 0.03 (9H, s).

^{13}C{^{1}H} NMR (100 MHz; CDCl$_3$) δ: 170.7, 151.4, 133.9, 132.0, 126.7 (q, $J_{C\text{-}F}$ = 3.7 Hz), 124.7 (q, $J_{C\text{-}F}$ = 270.7 Hz), 123.0 (q, $J_{C\text{-}F}$ = 33.0 Hz), 113.1, 38.0, 28.6, 22.7, 18.3, -0.8.

^{19}F NMR (376 MHz; CDCl$_3$) δ: -60.4.

FTIR ν_{max} (ATR, film)/cm^{-1} 3247 (br), 2957, 1653, 1615, 1531, 1519, 1467.

HRMS (APCI+) Calculated for: $C_{18}H_{28}F_3N_2OSi$ [M+H]$^+$: 373.1918, found: 373.1917.

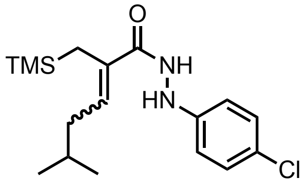

16ac

Following **General Procedure A**, the reaction was carried out using the carboxylic acid derivative **15a** (70.0 mg, 0.33 mmol, 1.0 equiv.), 4-chlorophenylhydrazine hydrochloride (88 mg, 0.49 mmol, 1.5 equiv.), Et_3N (68 µL, 0.49 mmol, 1.5 equiv.), DCC (101 mg, 0.49 mmol, 1.5 equiv.), DMAP (11.2 mg, 0.092 mmol, 0.28 equiv.), and 2 mL of anhydrous CH_2Cl_2. The crude product was purified with flash column chromatography using a solvent system of 1:9 → 1:5 → 1:1 EtOAc:hexanes. Compound **64c** was obtained as an orange solid (69.6 mg, 62% yield, dr (*Z*:*E*) = 3:1).

***R**$_f$* = 0.32 (1:5 EtOAc: hexanes)

TLC Visualization: Visible under 254 nm UV light; stains with basic $KMnO_4$ solution.

^{1}H NMR (400 MHz; $CDCl_3$) δ; major diastereomer (*Z*): 7.67 (1H, br s), 7.17-7.13 (2H, m), 6.79-6.75 (2H, m), 6.34 (1H, br d, *J* = 3.3 Hz), 6.12 (1H, t, *J* = 7.1 Hz), 1.99 (2H, t, *J* = 7.0 Hz), 1.84 (2H, s), 1.76-1.61 (1H, m), 0.94 (6H, d, J = 6.7 Hz), 0.01 (9H, s).

minor diastereomer (*E*): 7.36 (1H, br d, *J* = 4.2 Hz), 7.17-7.13 (2H, m), 6.79-6.75 (2H, m), 6.37 (1H, br s), 5.47 (1H, t, *J* = 7.6 Hz), 2.12 (2H, t, *J* = 7.2 Hz), 1.71 (2H, s), 1.76- 1.61 (1H, m), 0.92 (6H, d, *J* = 6.7 Hz), 0.04 (9H, s).

^{13}C{^{1}H} NMR (100 MHz; $CDCl_3$) δ; major diastereomer (*Z*): 170.6, 147.2, 133.9, 131.6, 129.1, 126.1, 115.2, 38.0, 28.6, 22.7, 18.2, -0.8.

minor diastereomer (*E*): 170.7, 147.0, 132.6, 131.3, 129.1, 126.2, 115.4, 38.8, 29.2, 24.9, 22.5, -1.3.

FTIR ν_{max} (ATR, film)/cm^{-1} 3261 (broad), 2955, 1656, 1626, 1597, 1490.

HRMS (ESI-) Calculated for: $C_{17}H_{26}ClN_2OSi$ [M-H]$^-$: 337.1508, found: 337.1509.

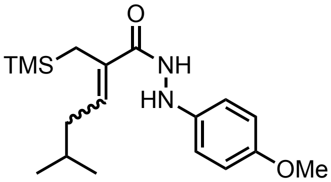

16ad

Following **General Procedure A**, the reaction was carried out using the carboxylic acid derivative **15a** (70 mg, 0.33 mmol, 1.0 equiv.), 4-methoxyphenylhydrazine hydrochloride (86 mg, 0.49 mmol, 1.5 equiv.), Et_3N (68 µL, 0.49 mmol, 1.5 equiv.), DCC (101 mg, 0.49 mmol, 1.5 equiv.), DMAP (11.2 mg, 0.092 mmol, 0.28 equiv.) and 2 mL anhydrous of CH_2Cl_2. The crude product was purified with flash column chromatography using a solvent system of 1:19 → 1:9 → 1:5 → 1:1 EtOAc:hexanes → pure EtOAc. Compound **16ad** was obtained as an orange oil (89.2 mg, 81% yield, dr (*Z*:*E*) = 3:1).

R*f* = 0.26 (1:5 EtOAc: hexanes)

TLC Visualization: Visible under 254 nm UV light; stains with basic $KMnO_4$ solution.

^{1}H NMR (400 MHz; $CDCl_3$) δ; major diastereomer (*Z*): 7.85 (1H, br s), 6.85-6.75 (4H, m), 6.25 (1H, br s), 6.09 (1H, t, *J* = 7.1 Hz), 3.73 (3H, s), 1.96 (2H, t, *J* = 7.0 Hz), 1.83 (2H, s), 1.73-1.60 (1H, m), 0.92 (6H, d, J = 6.6 Hz), 0.00 (9H, s).

minor diastereomer (*E*): 7.50 (1H, br s), 6.85-6.75 (4H, m), 5.43 (1H, t, *J* = 7.7 Hz), 3.74 (3H, s), 2.10 (2H, t, *J* = 7.2 Hz), 1.70 (2H, s), 1.73-1.60 (1H, m), 0.90 (6H, d, *J* = 6.6 Hz), 0.02 (9H, s).

^{13}C{^{1}H} NMR (100 MHz; $CDCl_3$) δ; major diastereomer (*Z*): 170.6, 154.8, 142.1,134.0, 131.2, 115.9, 114.6, 55.7, 37.9, 28.5, 22.6, 18.1, -0.9.

minor diastereomer (*E*): 170.6, 154.9, 141.9, 132.9, 130.7, 116.3, 114.6, 55.7, 38.6, 29.1, 24.9, 22.4, -1.3.

FTIR ν_{max} (ATR, film)/cm^{-1} 3248 (br), 2996, 1654, 1625, 1508, 1466.

HRMS (APCI-) Calculated for: $C_{18}H_{29}N_2O_2Si$ [M-H]$^-$: 333.2004, found: 333.2006.

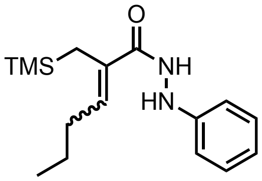

16b

Following **General Procedure A**, the reaction was carried out using the carboxylic acid derivative **15b** (128 mg, 0.64 mmol, 1.0 equiv.), phenylhydrazine (90 µL, 0.96 mmol, 1.5 equiv.), DCC (198 mg, 0.96 mmol, 1.5 equiv.), DMAP (34.2 mg, 0.18 mmol, 0.28 equiv.) and 1.5 mL of anhydrous CH_2Cl_2. The crude product was purified with flash column chromatography using a solvent system of 1:19 → 1:9 → 1:5 → 1:3 → 1:1 EtOAc:hexanes. Compound **16b** was obtained as an orange solid (125 mg, 67% yield, dr (*Z*:*E*) = 4.5:1).

R*f* = 0.39 (1:5 EtOAc: hexanes)

TLC Visualization: Visible under 254 nm UV light; stains with basic $KMnO_4$ solution.

^{1}H NMR (400 MHz; $CDCl_3$) δ; major diastereomer (*Z*): 8.07 (1H, br d, *J* = 3.5 Hz), 7.18 (2H, t, *J* = 7.9 Hz), 6.87 (1H, t, *J* = 7.3 Hz), 6.81 (1H, d, *J* = 7.7 Hz), 6.44 (1H, br d, *J* = 3.8 Hz),

6.05 (1H, t, J = 7.0 Hz), 2.04 (2H, q, J = 7.2 Hz), 1.84 (2H, s), 1.43 (2H, sext, J = 7.5 Hz), 0.95 (3H, t, J = 7.4 Hz), 0.01 (9H, s).

minor diastereomer (*E*): 7.77 (1H, br d, J = 4.0 Hz), 7.22-7.16 (2H, m), 6.91-6.86 (1H, m), 6.84-6.80 (2H, m), 6.48 (1H, br d, J = 4.2 Hz), 5.41 (1H, t, J = 7.6 Hz), 2.18 (2H, q, J = 7.4 Hz), 1.68 (2H, s), 1.47-1.38 (2H, m), 0.91 (3H, t, J = 7.4 Hz), 0.03 (9H, s).

$^{13}C\{^1H\}$ NMR (100 MHz; $CDCl_3$) δ; major diastereomer (*Z*): 170.5, 148.4, 133.2, 132.2, 129.1, 121.05, 113.9, 30.9, 22.2, 17.9, 14.0, -1.0.

minor diastereomer (*E*): 170.6, 148.2, 131.8, 129.2, 121.12, 114.0, 31.6, 24.6, 23.2, 13.7, -1.4.

FTIR ν_{max} (ATR, film)/cm^{-1} 3264 (br), 2956, 2929, 1655, 1626, 1601, 1532, 1495.

HRMS (ESI-) Calculated for: $C_{16}H_{25}N_2OSi$ $[M-H]^-$: 289.1741, found: 289.1741.

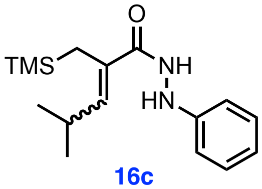

16c

Following **General Procedure A**, the reaction was carried out using the carboxylic acid derivative **15c** (411 mg, 2.05 mmol, 1.0 equiv.), phenylhydrazine (0.30 mL, 3.08 mmol, 1.5 equiv.), DCC (636 mg, 3.08 mmol, 1.5 equiv.), DMAP (70.1 mg, 0.57 mmol, 0.28 equiv.) and 6.5 mL of anhydrous CH_2Cl_2. The crude product was purified with flash column chromatography using a solvent system of 1:19 → 1:9 → 1:5 → 1:3 → 1:1 EtOAc:hexanes → pure EtOAc. Compound **16c** was obtained as an orange solid (211 mg, 35% yield, dr (Z:*E*) = 1:1.4).

R_f = 0.17 (1:9 EtOAc: hexanes)

TLC Visualization: Visible under 254 nm UV light; stains with basic $KMnO_4$ solution.

^1H NMR (400 MHz; $CDCl_3$) δ; major diastereomer (*E*): 7.39 (1H, br d, J = 3.6 Hz), 7.25-7.20 (2H, m), 6.91 (1H, t, J = 8.0 Hz), 6.86 (2H, d, J = 7.6 Hz), 6.32 (1H, br d, J = 4.2 Hz), 5.24 (1H, d, J = 10.2 Hz), 2.82-2.70 (1H, m), 1.66 (2H, d, J = 0.9 Hz), 1.01 (6H, d, J = 6.6 Hz), 0.04 (9H, s).

minor diastereomer (*Z*): 7.67 (1H, br d, J = 2.6 Hz), 7.25-7.20 (2H, m), 6.91 (1H, t, J = 8.0 Hz), 6.86 (2H, d, J = 8.6 Hz), 6.28 (1H, br d, J = 3.5 Hz), 5.87 (1H, d, J = 9.7 Hz), 2.60-2.48 (1H, m), 1.85 (2H, d, J = 0.6 Hz), 1.02 (6H, d, J = 6.6 Hz), 0.03 (9H, s).

$^{13}C\{^{1}H\}$ NMR (100 MHz; $CDCl_3$) δ; major diastereomer (*E*): 170.7, 148.5, 139.4, 129.6, 129.3, 121.4, 114.1, 29.1, 24.6, 23.6, -1.3.

minor diastereomer (*Z*): 170.7, 148.3, 139.0, 131.3, 129.2, 121.3, 114.0, 27.9, 23.6, 17.8, -1.0.

FTIR ν_{max} (ATR, film)/cm^{-1} 3250 (br), 2959, 2923, 1656, 1601, 1495, 1467, 1259.

HRMS (ESI-) Calculated for: $C_{16}H_{25}N_2OSi$ $[M-H]^-$: 289.1741, found: 289.1742.

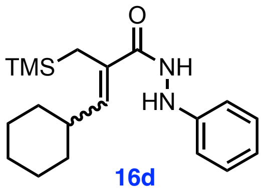

16d

Following **General Procedure A**, the reaction was carried out using the carboxylic acid derivative **15d** (165 mg, 0.68 mmol, 1.0 equiv.), phenylhydrazine (0.10 mL, 1.0 mmol, 1.5 equiv.), DCC (206 mg, 1.0 mmol, 1.5 equiv.), DMAP (23.2 mg, 0.19 mmol, 0.28 equiv.) and 3 mL of anhydrous CH_2Cl_2. The crude product was purified with flash column chromatography using a solvent system of 1:19 → 1:5 → 1:1 EtOAc:hexanes. Compound **16d** was obtained as an orange solid (132 mg, 59% yield, dr (Z:E) = 1:1.5).

R_f = 0.44 (1:5 EtOAc: hexanes)

TLC Visualization: Visible under 254 nm UV light; stains with basic $KMnO_4$ solution.

^{1}H NMR (400 MHz; $CDCl_3$) δ; major diastereomer (*E*): 7.44 (1H, br d, *J* = 3.8 Hz), 7.26-7.19 (2H, m), 6.93-6.84 (3H, m), 6.33 (1H, br d, *J* = 4.2 Hz), 5.25 (1H, d, *J* = 10.1 Hz), 2.46 (1H, qt, *J* = 10.9, 3.4 Hz), 1.77-1.65 (4H, m), 1.65 (2H, s), 1.31-1.20 (3H, m), 1.20-1.04 (3H, m), 0.04 (9H, s).

minor diastereomer (*Z*): 7.76 (1H, br d, *J* = 2.7 Hz), 7.25-7.19 (2H, m), 6.93-6.84 (3H, m), 6.29 (1H, br d, *J* = 3.5 Hz), 5.89 (1H, d, *J* = 9.7 Hz), 2.24-2.15 (1H, m), 1.85 (2H, s), 1.77-1.65 (4H, m), 1.31-1.20 (3H, m), 1.20-1.04 (3H, m), 0.03 (9H, s).

$^{13}C\{^{1}H\}$ NMR (100 MHz; $CDCl_3$) δ; major diastereomer (*E*): 170.8, 148.3, 137.7, 130.2, 129.2, 121.4, 114.2, 38.7, 33.8, 26.0, 25.8, 24.6, -1.4.

minor diastereomer (*Z*): 170.8, 148.5, 137.4, 131.5, 129.2, 121.2, 114.0, 37.8, 32.4, 26.0, 25.8, 17.8, -1.0.

FTIR ν_{max} (ATR, film)/cm^{-1} 3257 (br), 2925, 2851, 1655, 1628, 1602, 1495, 1467, 1449.

HRMS (APCI-) Calculated for: $C_{19}H_{29}N_2OSi$ $[M-H]^-$: 329.2055, found: 329.2051.

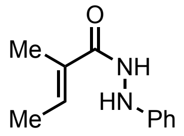

18

Following **General Procedure A**, the reaction was carried out using tiglic acid (100 mg, 1.0 mmol, 1.0 equiv.), phenylhydrazine (0.15 mL, 1.5 mmol, 1.5 equiv.), DCC (310 mg, 1.5 mmol, 1.5 equiv.), DMAP (34.2 mg, 0.28 mmol, 0.28 equiv.), and 3 mL of anhydrous CH_2Cl_2. The crude product was purified with flash column chromatography using a solvent system of 1:9 → 1:7 → 1:5 EtOAc:hexanes. Compound **18** was obtained as an orange solid (143 mg, 75% yield).

R*f* = 0.15 (1:3 EtOAc: hexanes)

TLC Visualization: Visible under 254 nm UV light; stains with basic $KMnO_4$ solution.

^{1}H NMR (400 MHz; $CDCl_3$) δ: 8.24 (1H, br s), 7.17 (2H, dd, *J* = 8.4, 1.2 Hz), 6.86 (1H, t, *J* = 7.3 Hz), 6.77 (2H, d, *J* = 8.5 Hz), 6.48-6.45 (1H, br m), 1.79 (3H, t, *J* = 1.2 Hz), 1.70 (3H, dd, *J* = 6.9, 1.0 Hz).

^{13}C{^{1}H} NMR (100 MHz; $CDCl_3$) δ: 169.7, 148.3, 132.3, 130.0, 129.0, 120.9, 113.7, 13.9, 12.0.

FTIR ν_{max} (ATR, film)/cm^{-1} 3275 (br), 3052, 3023, 2926, 2855, 1664, 1629, 1600, 1493, 1435, 1383.

HRMS (APCI+) Calculated for: $C_{11}H_{15}N_2O$ $[M+H]^+$: 191.1179, found: 191.1179.

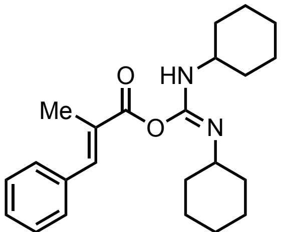

S3

In a 25-mL round bottomed flask, after 3 cycles of vacuum-N_2, compound **S2** (309 mg, 1.91 mmol, 1.0 equiv.) was dissolved in 5 mL of anhydrous CH_2Cl_2, and phenylhydrazine (0.28 mL, 2.86 mmol, 1.5 equiv.), DCC (590 mg, 2.86 mmol, 1.5 equiv.), and DMAP (64.8 mg, 0.53 mmol, 0.28 equiv.) were added. The reaction mixture was stirred for 24 h, then 5 mL of distilled water was added as aqueous work-up. Then, the aqueous layer was extracted twice with EtOAc.

After passing the aqueous layer through a cotton-plugged funnel to filter the white precipitate, it was extracted three more times with EtOAc. The organic layers were combined, dried over anhydrous Na_2SO_4, and concentrated *in vacuo*. The crude product was purified with flash column chromatography using a solvent system of 1:9 → 1:5 → 1:3 → 1:1 EtOAc:hexanes. Compound **S3** was obtained as an orange solid (97.4 mg).

$\boldsymbol{R_f}$ = 0.33 (1:5 EtOAc: hexanes)

TLC Visualization: Visible under 254 nm UV light; stains with basic $KMnO_4$ solution.

^{1}H NMR (400 MHz; $CDCl_3$) δ: 7.36 (2H, app t, *J* = 7.5 Hz), 7.29-7.26 (3H, m), 7.10 (1H, br d, *J* = 7.4 Hz), 6.73 (1H, s), 3.99 (1H, tt, *J* = 11.9 Hz, 3.1 Hz), 3.64 (1H, ddquint, *J* = 11.2 Hz, 7.8 Hz, 3.8 Hz), 2.13-2.09 (1H, m), 2.11 (3H, d, *J* = 1.4 Hz), 1.90-1.87 (2H, m), 1.80 (4H, d, *J* = 10.4 Hz), 1.68-1.54 (4H, m), 1.37-1.11 (9H, m).

^{13}C{^{1}H} NMR (100 MHz; $CDCl_3$) δ: 174.9, 154.4, 135.7, 134.4, 130.2, 129.2, 128.5, 127.8, 58.3, 49.7, 32.9, 30.9, 26.6, 25.6, 25.4, 24.7, 15.9.

FTIR ν_{max} (ATR, film)/cm^{-1} 3312 (br), 2930, 2855, 1703, 1683, 1645, 1620, 1525, 1450.

HRMS (ESI+) Calculated for: $C_{23}H_{33}N_2O_2$ $[M+H]^+$: 369.2537, found: 369.2539.

Syntheses of *N*-Acylazo Derivatives 10 and 19:

General Procedure B:

In an oven-dried round bottomed flask, after three cycles of vacuum-N_2, the hydrazide derivative (1.0 equiv.) was dissolved in anhydrous THF, and the flask was immersed in an ice bath. Then, NaH (1.2 equiv.) was added portion-wise. After 30 min of stirring, the ice bath was removed, NBS (1.1 equiv.) was added, and the mixture was stirred for 30 min at 23 °C. Then, $NaOH_{(aq)}$ (10.0 equiv.) was added, and the reaction mixture was stirred for 30 min. Then, 3 mL of $NH_4Cl_{(satd)}$ was added as aqueous work-up. The aqueous layer was extracted thrice with EtOAc. The organic layers were combined, dried over anhydrous Na_2SO_4, and concentrated *in vacuo*. The *N*-acylazo product was purified with flash column chromatography.

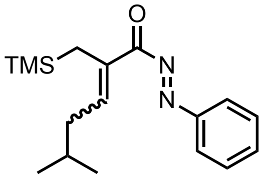

10aa

Following **General Procedure B**, the reaction was carried out using the hydrazide derivative **16aa** (211 mg, 0.69 mmol, 1.0 equiv.), NaH (33.3 mg, 0.83 mmol, 1.2 equiv., 60% dispersion in paraffin liquid), NBS (135 mg, 0.76 mmol, 1.1 equiv.), $NaOH_{(aq)}$ (3.5 M, 2.0 mL, 10.0 equiv.), and 5.0 mL of anhydrous THF. The crude product was purified with flash column chromatography using a solvent system of 1:9 EtOAc:hexanes. Compound **10aa** was obtained as an orange oil (147 mg, 70% yield, dr (*Z*:*E*) = 12.5:1).

R_f = 0.55 (1:19 EtOAc: hexanes)
TLC Visualization: Visible under 254 nm UV light; stains with basic $KMnO_4$ solution.
^{1}H NMR (400 MHz; $CDCl_3$) δ: 7.91-7.89 (2H, m), 7.56-7.52 (3H, m), 6.65 (1H, t, *J* = 7.3 Hz), 2.13 (2H, t, *J* = 7.1 Hz), 1.98 (2H, s), 1.73 (1H, n, *J* = 6.7 Hz), 0.92 (6H, d, *J* = 6.7 Hz), 0.08 (9H, s).
^{13}C{^{1}H} NMR (100 MHz; $CDCl_3$) δ: 183.5, 152.3, 145.9, 134.9, 132.8, 129.4, 123.4, 39.0, 28.5, 22.7, 17.2, -0.7.
FTIR ν_{max} (ATR, film)/cm^{-1} 2954, 2869, 1695, 1621, 1502, 1453.
HRMS (APCI+) Calculated for: $C_{17}H_{27}N_2OSi$ $[M+H]^+$: 303.1888, found: 303.1888.

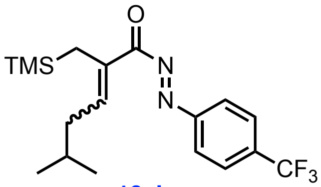

10ab

Following **General Procedure B**, the reaction was carried out using the hydrazide derivative **16ab** (46.7 mg, 0.13 mmol, 1.0 equiv.), NaH (6.0 mg, 0.15 mmol, 1.2 equiv., 60% dispersion in paraffin liquid), NBS (24.9 mg, 0.14 mmol, 1.1 equiv.), and $NaOH_{(aq)}$ (3.5 M, 0.4 mL, 10.0 equiv.), and 2.0 mL of anhydrous THF. The crude product was purified with flash column chromatography using a solvent system of 1:5 EtOAc:hexanes. Compound **10ab** was obtained as an orange oil (38.1 mg, 82% yield).

R$_f$ = 0.69 (1:19 EtOAc: hexanes)

TLC Visualization: Visible under 254 nm UV light; stains with basic $KMnO_4$ solution.

^{1}H NMR (400 MHz; $CDCl_3$) δ: 7.98 (2H, d, *J* = 8.2 Hz), 7.80 (2H, d, *J* = 8.4 Hz), 6.59 (1H, t, *J* = 7.2 Hz), 2.14 (2H, t, *J* = 7.1 Hz), 1.99 (2H, s), 1.73 (1H, n, *J* = 6.7 Hz), 0.93 (6H, d, *J* = 6.6 Hz), 0.08 (9H, s).

^{13}C{^{1}H} NMR (100 MHz; $CDCl_3$) δ: 183.0, 153.8, 146.5, 134.7, 134.1 (q, $J_{C\text{-}F}$ = 32.9 Hz), 126.7 (q, $J_{C\text{-}F}$ = 3.7 Hz), 123.8 (q, $J_{C\text{-}F}$ = 272.5 Hz), 123.6, 39.0, 28.5, 22.7, 17.3, -0.8.

^{19}F NMR (376 MHz; $CDCl_3$) δ: -62.4.

FTIR ν_{max} (ATR, film)/cm^{-1} 2957, 2929, 1701, 1621, 1518.

HRMS (APCI+) Calculated for: $C_{18}H_{26}F_3N_2OSi$ $[M+H]^+$: 371.1761, found: 371.1761.

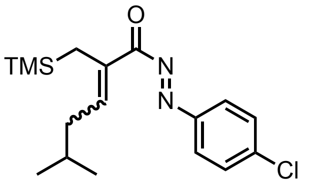

10ac

Following **General Procedure B**, the reaction was carried out using the hydrazide derivative **16ac** (44.9 mg, 0.13 mmol, 1.0 equiv.), NaH (6.4 mg, 0.16 mmol, 1.2 equiv., 60% dispersion in paraffin liquid), NBS (24.9 mg, 0.14 mmol, 1.1 equiv.), and $NaOH_{(aq)}$ (3.5 M, 0.37 mL, 10.0 equiv.), and 1.0 mL of anhydrous THF. The crude product was purified with flash column chromatography using a solvent system of 1:9 EtOAc:hexanes. Compound **10ac** was obtained as an orange oil (33.0 mg, 75% yield, dr (*Z*:*E*) = 3.6:1).

R$_f$ = 0.54 (1:19 EtOAc: hexanes)

TLC Visualization: Visible under 254 nm UV light; stains with basic $KMnO_4$ solution.

^{1}H NMR (400 MHz; $CDCl_3$) δ; major diastereomer (*Z*): 7.86-7.82 (2H, m), 7.52-7.49 (2H, m), 6.63 (1H, t, *J* = 7.3 Hz), 2.13 (2H, t, *J* = 7.0 Hz), 1.97 (2H, s), 1.78-1.68 (1H, m), 0.92 (6H, d, *J* = 6.6 Hz), 0.07 (9H, s).

minor diastereomer (*E*): 7.86-7.82 (2H, m), 7.52-7.49 (2H, m), 6.11 (1H, t, *J* = 7.9 Hz), 2.12 (2H, t, *J* = 6.5 Hz), 1.88 (2H, s), 1.64-1.54 (1H, m), 0.83 (6H, d, *J* = 6.6 Hz), 0.05 (9H, s).

^{13}C{^{1}H} NMR (100 MHz; $CDCl_3$) δ; major diastereomer (*Z*): 183.1, 150.6, 146.2, 139.1, 134.9, 129.7, 124.7, 39.0, 28.5, 22.7, 17.2, -0.7.

minor diastereomer (*E*): 185.5, 150.4, 143.3, 139.3, 132.3, 129.8, 124.8, 40.6, 29.3, 23.8, 22.4, -1.3.

FTIR ν_{max} (ATR, film)/cm^{-1} 2956, 2926, 1699, 1499.

HRMS (APCI+) Calculated for: $C_{17}H_{26}ClN_2OSi$ $[M+H]^+$: 337.1498, found: 337.1497.

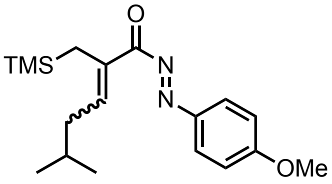

10ad

Following **General Procedure B**, the reaction was carried out using the hydrazide derivative **16ad** (75.5 mg, 0.23 mmol, 1.0 equiv.), NaH (10.8 mg, 0.27 mmol, 1.2 equiv., 60% dispersion in paraffin liquid), NBS (44.5 mg, 0.25 mmol, 1.1 equiv.), and $NaOH_{(aq)}$ (3.5 M, 0.66 mL, 10.0 equiv.), and 1.5 mL of anhydrous THF. The crude product was purified with flash column chromatography using a solvent system of 1:9 EtOAc:hexanes. Compound **10ad** was obtained as an orange oil (55.3 mg, 74% yield, dr (*Z*:*E*) = 7:1).

R_f = 0.65 (1:5 EtOAc: hexanes)

TLC Visualization: Visible under 254 nm UV light; stains with basic $KMnO_4$ solution.

^{1}H NMR (400 MHz; $CDCl_3$) δ; major diastereomer (*Z*): 7.93-7.89 (2H, m), 7.02-6.98 (2H, m), 6.75 (1H, t, *J* = 7.3 Hz), 3.89 (3H, s), 2.13 (2H, t, *J* = 7.1 Hz), 1.97 (2H, s), 1.79-1.68 (1H, m), 0.93 (6H, d, *J* = 6.7 Hz), 0.07 (9H, s).

minor diastereomer (*E*): 7.93-7.87 (2H, m), 7.02-6.98 (2H, m), 6.07 (1H, t, *J* = 7.9 Hz), 3.89 (3H, s), 2.17-2.13 (2H, m), 1.89 (2H, s), 1.63-1.57 (1H, m), 0.83 (6H, d, *J* = 6.6 Hz), 0.04 (9H, s).

^{13}C{^{1}H} NMR (100 MHz; $CDCl_3$) δ; major diastereomer (*Z*): 183.2, 163.7, 146.8, 145.5, 135.3, 125.8, 114.4, 55.8, 39.0, 28.5, 22.7, 17.2, -0.7.

minor diastereomer (*E*): 185.7, 163.8, 146.6, 142.4, 132.8, 125.9, 114.5, 55.8, 40.4, 29.3, 23.9, 22.4, -1.3.

FTIR ν_{max} (ATR, film)/cm^{-1} 2956, 1692, 1602, 1505, 1466, 1421.

HRMS (APCI+) Calculated for: $C_{18}H_{29}N_2O_2Si$ $[M+H]^+$: 333.1993, found: 333.1993.

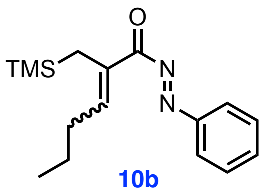

10b

Following **General Procedure B**, the reaction was carried out using the hydrazide derivative **16b** (123 mg, 0.42 mmol, 1.0 equiv.), NaH (20.4 mg, 0.51 mmol, 1.2 equiv., 60% dispersion in paraffin liquid), NBS (82 mg, 0.46 mmol, 1.1 equiv.), and $NaOH_{(aq)}$ (3.5 M, 1.2 mL, 10.0 equiv.), and 2.0 mL of anhydrous THF. The crude product was purified with flash column chromatography using a solvent system of 1:9 EtOAc:hexanes. Compound **10b** was obtained as an orange oil (104.5 mg, 86% yield, dr (*Z*:*E*) = 6.7:1).

R_f = 0.78 (1:9 EtOAc: hexanes)

TLC Visualization: Visible under 254 nm UV light; stains with basic $KMnO_4$ solution.

^{1}H NMR (400 MHz; $CDCl_3$) δ; major diastereomer (*Z*): 7.91-7.88 (2H, m), 7.55-7.50 (3H, m), 6.62 (1H, t, *J* = 7.3 Hz), 2.22 (2H, q, *J* = 7.4 Hz), 1.99 (2H, s), 1.47 (2H, sext, *J* = 7.4 Hz), 0.94 (3H, t, *J* = 7.4 Hz), 0.09 (9H, s).

minor diastereomer (*E*): 7.91-7.88 (2H, m), 7.55-7.50 (2H, m), 6.10 (1H, t, *J* = 7.9 Hz), 2.22 (2H, q, *J* = 7.4 Hz), 1.89 (2H, s), 1.39-1.30 (2H, m), 0.79 (3H, t, *J* = 7.4 Hz), 0.06 (9H, s).

^{13}C{^{1}H} NMR (100 MHz; $CDCl_3$) δ; major diastereomer (*Z*): 183.5, 152.2, 146.8, 134.3, 132.8, 129.3, 123.4, 32.0, 22.0, 17.1, 14.1, -0.8.

minor diastereomer (*E*): 185.8, 152.1, 146.8, 144.1, 133.0, 129.4, 123.5, 33.7, 23.6, 23.1, 13.7, -1.4.

FTIR ν_{max} (ATR, film)/cm^{-1} 2957, 2927, 1698, 1624, 1504, 1454.

HRMS (ESI+) Calculated for: $C_{16}H_{25}N_2OSi$ $[M+H]^+$: 289.1731, found: 289.1731.

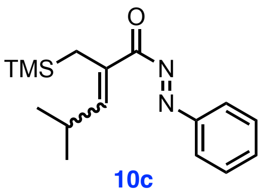

10c

Following **General Procedure B**, the reaction was carried out using the hydrazide derivative **16c** (67.4 mg, 0.23 mmol, 1.0 equiv.), NaH (11.2 mg, 0.28 mmol, 1.2 equiv., 60% dispersion in paraffin liquid), NBS (46.3 mg, 0.26 mmol, 1.1 equiv.), and $NaOH_{(aq)}$ (3.5 M, 0.66 mL, 10.0

equiv.), and 2.0 mL of anhydrous THF. The crude product was purified with flash column chromatography using a solvent system of 1:9 → 1:5 EtOAc:hexanes. Compound **10c** was obtained as an orange oil (37.5 mg, 57% yield, dr (*Z*:*E*) = 1:1).

R_f = 0.47 (1:19 EtOAc: hexanes)

TLC Visualisation: Visible under 254 nm UV light; stains with basic $KMnO_4$ solution.

^{1}H NMR (400 MHz; $CDCl_3$) δ (diastereomer *Z*): 7.93-7.85 (2H, m), 7.57-7.49 (3H, m), 6.41 (1H, d, *J* = 10.0 Hz), 2.73-2.62 (1H, m), 1.97 (2H, s), 1.03 (6H, d, *J* = 6.6 Hz), 0.09 (9H, s).

^{1}H NMR (400 MHz; $CDCl_3$) δ (diastereomer *E*): 7.93-7.85 (2H, m), 7.57-7.49 (3H, m), 5.84 (1H, d, *J* = 10.3 Hz), 2.86-2.74 (1H, m), 1.84 (2H, s), 0.91 (6H, d, *J* = 6.5 Hz), 0.05 (9H, s).

^{13}C{^{1}H} NMR (100 MHz; $CDCl_3$) δ (diastereomer *Z*): 183.9, 150.7, 152.1, 132.8, 130.9, 129.3, 123.51, 30.5, 23.5, 22.9, -0.9.

^{13}C{^{1}H} NMR (100 MHz; $CDCl_3$) δ (diastereomer *E*): 186.0, 153.0, 152.3, 133.0, 132.0, 129.4, 123.49, 29.0, 21.9, 16.9, -1.4.

FTIR ν_{max} (ATR, film)/cm^{-1} 2962, 2870, 1697, 1623, 1503, 1454.

HRMS (APCI+) Calculated for: $C_{16}H_{25}N_2OSi$ $[M+H]^+$: 289.1731, found: 289.1741.

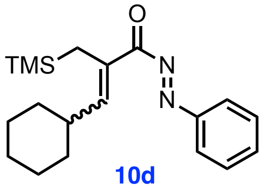

10d

Following **General Procedure B**, the reaction was carried out using the hydrazide derivative **16d** (132 mg, 0.40 mmol, 1.0 equiv.), NaH (19.2 mg, 0.248 mmol, 1.2 equiv., 60% dispersion in paraffin liquid), NBS (79.3 mg, 0.44 mmol, 1.1 equiv.), and $NaOH_{(aq)}$ (3.5 M, 1.14 mL, 10.0 equiv.), and 3.0 mL of anhydrous THF. The crude product was purified with flash column chromatography using a solvent system of 1:9 EtOAc:hexanes. Compound **10d** was obtained as an orange oil (95.0 mg, 72% yield, dr (*Z*:*E*) = 1:1.3).

R_f = 0.42 (1:19 EtOAc: hexanes)

TLC Visualization: Visible under 254 nm UV light; stains with basic $KMnO_4$ solution.

^{1}H NMR (400 MHz; $CDCl_3$) δ; major diastereomer (*E*): 7.91-7.88 (2H, m), 7.55-7.50 (3H, m), 5.89 (1H, d, *J* = 10.3 Hz), 2.45-2.31 (1H, m), 1.86 (2H, s), 1.76-1.66 (3H, m), 1.32-0.92 (7H, m), 0.05 (9H, s).

minor diastereomer (*Z*): 7.91-7.88 (2H, m), 7.55-7.50 (3H, m), 6.43 (1H, d, *J* = 10.0 Hz), 2.45-2.31 (1H, m), 1.98 (2H, s), 1.76-1.66 (3H, m), 1.32-0.92 (7H, m), 0.09 (9H, s).

$^{13}C\{^1H\}$ NMR (100 MHz; $CDCl_3$) δ; major diastereomer (*E*): 185.9, 152.0, 149.7, 133.0, 132.3, 129.4, 123.51, 40.4, 33.0, 25.8, 25.5, 23.5, -1.5.

minor diastereomer (*Z*): 183.9, 152.3, 151.5, 132.8, 129.8, 129.3, 123.45, 38.9, 31.9, 25.9, 25.6, 16.9, -0.9.

FTIR ν_{max} (ATR, film)/cm^{-1} 2926, 2852, 1696, 1621, 1502, 1451.

HRMS (APCI+) Calculated for: $C_{19}H_{29}N_2OSi$ $[M+H]^+$: 329.2044, found: 329.2044.

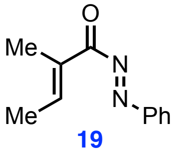

19

In an oven-dried 25-mL Schlenk flask, after 3 cycles of vacuum-N_2, hydrazide derivative **18** (30.0 mg, 0.16 mmol, 1.0 equiv.), was dissolved in 1 mL of anhydrous MeCN. Then, K_2CO_3 (66.3 mg, 0.48 mmol, 3.0 equiv.) was added, and the flask walls were washed with 1 mL of MeCN. After 10 min of stirring at room temperature, I_2 (48.2 mg, 0.19 mmol, 1.2 equiv.) was added, and the walls were washed with 0.5 mL of MeCN. The reaction mixture was stirred for 1 h at 23 °C, then 1 mL of $Na_2S_2O_{3(satd.)}$ was added as aqueous work-up. The organic layers were extracted from the aqueous layer thrice with EtOAc. They were combined, dried over anhydrous Na_2SO_4, and concentrated *in vacuo*. The crude product was purified with flash column chromatography using a solvent system of 1:5 EtOAc:hexanes. Compound **19** was obtained as an orange oil (24.4 mg, 82% yield).

R_f = 0.60 (1:5 EtOAc: hexanes)

TLC Visualization: Visible under 254 nm UV light; stains with basic $KMnO_4$ solution.

^1H NMR (400 MHz; $CDCl_3$) δ: 7.89 (2H, dd, *J* = 7.4, 1.5 Hz), 7.57-7.50 (3H, m), 6.86 (1H, q, *J* = 7.0 Hz), 2.00 (3H, d, *J* = 0.9 Hz), 1.92 (3H, d, *J* = 7.0 Hz).

$^{13}C\{^1H\}$ NMR (100 MHz; $CDCl_3$) δ: 183.6, 152.1, 145.2, 133.0, 132.4, 129.4, 123.5, 15.3, 11.6.

FTIR ν_{max} (ATR, film)/cm^{-1} 2954, 2923, 2854, 1697, 1641, 1504, 1454, 1390, 1257.

HRMS (ESI+) Calculated for: $C_{11}H_{13}N_2O$ $[M+H]^+$: 189.1022, found: 189.1021.

Syntheses of the Pyrazole Products 12 and 21:

General Procedure C for the Diaza-Nazarov Cyclization:

In a 20-mL vial or a round bottomed flask, the *N*-acyl azo derivative (1.0 equiv.) was dissolved in anhydrous CH_2Cl_2, and TFA (1.0-1.5 equiv.) was added. The reaction mixture was stirred for 5-6 h at 23 °C. Then, $NaHCO_{3(satd.)}$ was added as aqueous work-up. The aqueous layer was extracted thrice with EtOAc. The organic layers were combined, dried over anhydrous Na_2SO_4, and concentrated *in vacuo*. The hydroxypyrazole product was purified with flash column chromatography.

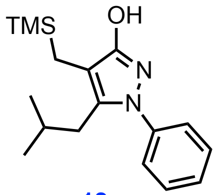

12aa

Following **General Procedure C**, the reaction was carried out using the *N*-acylazo derivative **10aa** (10.2 mg, 0.034 mmol, 1.0 equiv.), TFA (3.0 µL, 0.039 mmol, 1.2 equiv.), and 2.0 mL of anhydrous CH_2Cl_2. The crude product was purified with flash column chromatography using a solvent system of 1:9 → 1:1 EtOAc:hexanes. Compound **12aa** was obtained as a beige solid (9.9 mg, 97% yield).

***R**$_f$* = 0.63 (1:5 EtOAc: hexanes)

TLC Visualization: Visible under 254 nm UV light; stains with basic $KMnO_4$ solution.

^{1}H NMR (400 MHz; CDCl$_3$) δ: 7.42-7.38 (2H, m), 7.36-7.34 (2H, m), 7.29 (1H, t, *J* = 7.1 Hz), 2.46 (2H, d, *J* = 7.4 Hz), 1.66 (2H, s), 1.59-1.50 (1H, m), 0.71 (6H, d, *J* = 6.6 Hz), 0.03 (9H, s).

Note: When the ^{1}H NMR spectra of **12aa** were recorded in $CDCl_3$, occasionally, a broad signal at 11.70 ppm was observed, presumably due to the differences in concentration.

^{1}H NMR (400 MHz; acetone-*d*$_6$) δ: 10.40 (1H, br s), 7.49-7.41 (4H, m), 7.33 (1H, tt, *J* = 7.0, 1.7 Hz), 2.58 (2H, d, *J* = 7.4 Hz), 1.73 (2H, s), 1.56 (1H, n, *J* = 6.8 Hz), 0.72 (6H, d, *J* = 6.7 Hz), 0.05 (9H, s).

^{13}C{^{1}H} NMR (100 MHz; CDCl$_3$) δ: 161.2, 140.3, 139.9, 129.2, 127.0, 125.4, 103.5, 33.9, 28.1, 22.4, 11.2, -1.0.

FTIR ν_{max} (ATR, film)/cm^{-1} 2954, 2925, 1597, 1529, 1504, 1462.
HRMS (APCI+) Calculated for: $C_{17}H_{27}N_2OSi$ [M+H]$^+$: 303.1888, found: 303.1888.

The Diaza-Nazarov Reaction of 10aa on 1-mmol Scale:

In a 25-mL round bottomed flask, the *N*-acyl azo derivative **10aa** (370 mg, 1.22 mmol, 1.0 equiv.) was dissolved in 5.0 mL of anhydrous CH_2Cl_2, and TFA (94 µL, 1.22 mmol, 1.0 equiv.) was added. The reaction mixture was stirred for 5.5 h at 23 °C, and then 1.0 mL of additional CH_2Cl_2 was added. After the reaction mixture was stirred at 23 °C for an additional hour, more TFA (47 µL, 0.61 mmol, 0.5 equiv.) was added, and it was stirred for four more hours. Then, 4 mL of $NaHCO_{3(satd.)}$ was added as aqueous work-up. The aqueous layer was extracted thrice with EtOAc. The organic layers were combined, dried over anhydrous Na_2SO_4, and concentrated *in vacuo*. The crude product was purified with flash column chromatography using a solvent system of 1:9 → 1:5 → 1:3 → 1:1 EtOAc:hexanes. Compound **12aa** was obtained as a beige solid (326 mg, 88% yield).

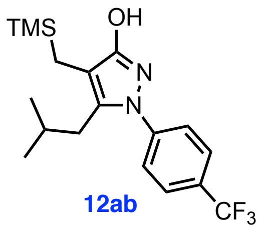

12ab

Following **General Procedure C**, the reaction was carried out using the *N*-acylazo derivative **10ab** (20.1 mg, 0.054 mmol, 1.0 equiv.), TFA (6.1 µL, 0.080 mmol, 1.5 equiv.), and 2.0 mL of anhydrous CH_2Cl_2. The crude product was purified with flash column chromatography using a solvent system of 1:9 → 1:5 EtOAc:hexanes. Compound **12ab** was obtained as a beige solid (18.4 mg, 92% yield).

R_f = 0.35 (1:5 EtOAc: hexanes)
TLC Visualization: Visible under 254 nm UV light; stains with basic $KMnO_4$ solution.
^{1}H NMR (400 MHz; CDCl$_3$) δ: 7.68 (2H, d, *J* = 8.4 Hz), 7.49 (2H, d, *J* = 8.3 Hz), 2.52 (2H, d, *J* = 7.4 Hz), 1.68 (2H, s), 1.63-1.52 (1H, m), 0.74 (6H, d, *J* = 6.6 Hz), 0.05 (9H, s).
^{13}C{^{1}H} NMR (100 MHz; CDCl$_3$) δ: 162.0, 142.9, 140.2, 128.5 (q, J_{C-F} = 32.4 Hz), 126.5 (q, J_{C-F} = 3.6 Hz), 124.6, 124.2 (q, J_{C-F} = 272 Hz), 105.2, 34.1, 28.3, 22.4, 11.3, -1.1.

^{19}F NMR (376 MHz; CDCl$_3$) δ: -61.7.

FTIR ν_{max} (ATR, film)/cm^{-1} 2959, 2926, 1617, 1528, 1465, 1414.

HRMS (APCI+) Calculated for: $C_{18}H_{26}F_3N_2OSi$ [M+H]$^+$: 371.1761, found: 371.1761.

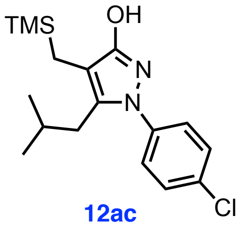

12ac

Following **General Procedure C**, the reaction was carried out using the *N*-acylazo derivative **10ac** (30.7 mg, 0.091 mmol, 1.0 equiv.), TFA (10.0 µL, 0.13 mmol, 1.4 equiv.), and 2.0 mL of anhydrous CH_2Cl_2. The crude product was purified with flash column chromatography using a solvent system of 1:9 EtOAc:hexanes. Compound **12ac** was obtained as a white solid (26.1 mg, 85% yield).

R$_f$ = 0.37 (1:9 EtOAc: hexanes)

TLC Visualization: Visible under 254 nm UV light; stains with basic $KMnO_4$ solution.

^{1}H NMR (400 MHz; CDCl$_3$) δ: 7.38 (2H, app d, *J* = 8.7 Hz), 7.29 (2H, app d, *J* = 8.7 Hz), 2.44 (2H, d, *J* = 7.4 Hz), 1.66 (2H, s), 1.60-1.50 (1H, m), 0.72 (6H, d, *J* = 6.6 Hz), 0.04 (9H, s).

^{13}C{^{1}H} NMR (100 MHz; CDCl$_3$) δ: 161.6, 139.9, 138.8, 132.4, 129.4, 126.3, 104.1, 33.9, 28.2, 22.4, 11.3, -1.1.

FTIR ν_{max} (ATR, film)/cm^{-1} 2955, 2928, 1598, 1530, 1500, 1465.

HRMS (ESI-) Calculated for: $C_{17}H_{24}ClN_2OSi$ [M-H]$^-$: 335.1351, found: 335.1344.

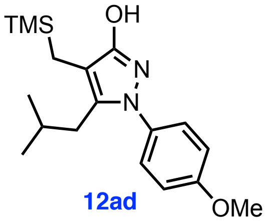

12ad

Following **General Procedure C**, the reaction was carried out using the *N*-acylazo derivative **10ad** (55.2 mg, 0.17 mmol, 1.0 equiv.), TFA (13.0 µL, 0.17 mmol, 1.0 equiv.), and 2.0 mL of anhydrous CH_2Cl_2. The crude product was purified with flash column chromatography using a

solvent system of 1:19 → 1:9 → 1:5 → 1:3 EtOAc:hexanes → pure EtOAc. Compound **12ad** was obtained as a white solid (54.9 mg, 99% yield).

$\boldsymbol{R_f}$ = 0.40 (1:5 EtOAc: hexanes)
TLC Visualization: Visible under 254 nm UV light; stains with basic $KMnO_4$ solution.
^{1}H NMR (400 MHz; $CDCl_3$) δ: 7.27 (2H, app d, J = 9.0 Hz), 6.93 (2H, app d, J = 8.9 Hz), 3.87 (3H, s), 2.41 (2H, d, J = 7.4 Hz), 1.67 (2H, s), 1.64-1.52 (1H, m), 0.74 (6H, d, J = 6.6 Hz), 0.05 (9H, s).
^{13}C{^{1}H} NMR (100 MHz; $CDCl_3$) δ: 161.1, 158.5, 140.0, 133.3, 127.0, 114.3, 102.5, 55.6, 33.9, 28.1, 22.4, 11.2, - 1.0.
FTIR ν_{max} (ATR, film)/cm^{-1} 3062, 2955, 1595, 1516, 1464.
HRMS (APCI-) Calculated for: $C_{18}H_{27}N_2O_2Si$ [M-H]$^-$: 331.1847, found: 331.1847.

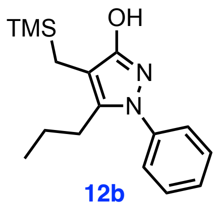

12b

Following **General Procedure C**, the reaction was carried out using the *N*-acylazo derivative **10d** (105 mg, 0.36 mmol, 1.0 equiv.), TFA (27.8 µL, 0.36 mmol, 1.0 equiv.), and 2.0 mL of anhydrous CH_2Cl_2. The crude product was purified with flash column chromatography using a solvent system of 1:19 → 1:9 → 1:5 → 1:3 → 1:1 EtOAc:hexanes. Compound **12b** was obtained as a beige/white solid (85.0 mg, 81% yield).

$\boldsymbol{R_f}$ = 0.34 (1:5 EtOAc: hexanes)
TLC Visualization: Visible under 254 nm UV light; stains with basic $KMnO_4$ solution.
^{1}H NMR (400 MHz; $CDCl_3$) δ: 11.76 (1H, br s), 7.44-7.36 (4H, m), 7.30 (1H, tt, J = 7.0, 1.4 Hz), 2.55 (2H, t, J = 7.9 Hz), 1.68 (2H, s), 1.38 (2H, sext, J = 7.4 Hz), 0.81 (3H, t, J = 7.4 Hz), 0.05 (9H, s).
^{13}C{^{1}H} NMR (100 MHz; $CDCl_3$) δ: 161.5, 140.5, 140.0, 129.1, 126.7, 124.9, 102.8, 27.0, 22.1, 14.0, 11.1, -1.0.
FTIR ν_{max} (ATR, film)/cm^{-1} 3032, 2962, 1600, 1523, 1506, 1464.
HRMS (ESI+) Calculated for: $C_{16}H_{25}N_2OSi$ [M+H]$^+$: 289.1731, found: 289.1734.

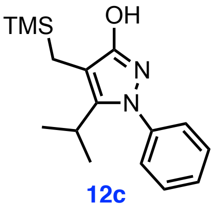

12c

and

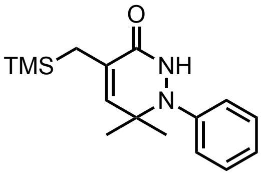

20a

Following **General Procedure C**, the reaction was carried out using the *N*-acylazo derivative **10c** (47.6 mg, 0.17 mmol, 1.0 equiv.), TFA (20.0 µL, 0.26 mmol, 1.5 equiv.), and 2.0 mL of anhydrous CH_2Cl_2. Purification with flash column chromatography using a solvent system of 1% MeOH in 1:9 EtOAc:hexanes → 1% MeOH in 1:5 EtOAc:hexanes gave pyrazole product **12c** as a beige solid (35.1 mg, 74% yield), and dihyropyridazinone side product **20a** as a white solid (7.4 mg, 16% yield).

Characterization Data for 12c:

$\boldsymbol{R_f}$ = 0.58 (1:5 EtOAc: hexanes)

TLC Visualization: Visible under 254 nm UV light; stains with basic $KMnO_4$ solution.

^{1}H NMR (400 MHz; $CDCl_3$) δ: 10.36 (1H, br s), 7.44-7.37 (2H, m), 7.34-7.31 (3H, m), 3.03 (1H, sept, J = 7.2 Hz), 1.77 (2H, s), 1.21 (6H, d, J = 7.2 Hz), 0.03 (9H, s).

^{13}C{^{1}H} NMR (100 MHz; $CDCl_3$) δ: 161.7, 145.2, 140.1, 129.0, 127.4, 126.6, 101.3, 26.1, 21.7, 11.4, -0.4.

FTIR ν_{max} (ATR, film)/cm^{-1} 3045, 2962, 1597, 1530, 1504, 1457, 1349.

HRMS (ESI+) Calculated for: $C_{16}H_{25}N_2OSi$ $[M+H]^+$: 289.1731, found: 289.1731.

Characterization Data for 20a:

$\boldsymbol{R_f}$ = 0.48 (1:3 EtOAc: hexanes)

TLC Visualization: Visible under 254 nm UV light; stains with basic $KMnO_4$ solution.

^{1}H NMR (400 MHz; $CDCl_3$) δ: 7.20 (2H, t, J = 7.9 Hz), 6.86 (1H, t, J = 7.4 Hz), 6.78 (2H, d, J = 8.3 Hz), 6.51 (1H, s), 5.89 (1H, s), 1.76 (2H, s), 1.33 (6H, s), 0.05 (9H, s).

^{13}C{^{1}H} NMR (100 MHz; $CDCl_3$) δ: 170.2, 147.8, 143.8, 133.8, 129.2, 120.9, 113.5, 63.2, 24.3, 15.6, -1.52.

FTIR ν_{max} (ATR, film)/cm^{-1} 3255 (broad), 3056, 3029, 2955, 2931, 2897, 1694, 1631, 1603, 1527, 1497, 1248.

HRMS (ESI+) Calculated for: $C_{16}H_{25}N_2OSi$ $[M+H]^+$: 289.1731, found: 289.1736.

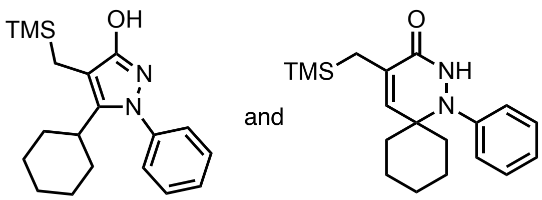

12d **20b**

In a vial, the *N*-acylazo derivative **10d** (48.6 mg, 0.15 mmol, 1.0 equiv.) was dissolved in 2 mL of anhydrous MeCN. Then TFA (11.5 µL, 0.15 mmol, 1.0 equiv.) was added, and the reaction mixture was stirred at 75 °C for 4.5 h. Purification with preparatory TLC (EtOAc:hexanes 1:5) gave pyrazole product **12d** as a beige solid (20.5 mg, 42% yield), and dihyropyridazinone side product **20b** as a white solid (9.4 mg, 19% yield).

Characterization Data for 12d:

R_f = 0.58 (1:5 EtOAc: hexanes)

TLC Visualization: Visible under 254 nm UV light; stains with basic $KMnO_4$ solution.

^{1}H NMR (400 MHz; $CDCl_3$) δ: 7.41 (2H, t, *J* = 7.5 Hz), 7.35-7.29 (3H, m), 2.62 (1H, tt, *J* = 12.4, 2.7 Hz), 1.79 (2H, s), 1.72 (3H, br d, *J* = 10.0 Hz), 1.67-1.52 (4H, m), 1.26-1.14 (3H, m), 0.04 (9H, s).

^{13}C{^{1}H} NMR (100 MHz; $CDCl_3$) δ: 161.7, 144.3, 140.0, 128.9, 127.2, 126.4, 101.6, 36.7, 31.7, 26.9, 26.0, 11.6, -0.4.

FTIR ν_{max} (ATR, film)/cm^{-1} 3060, 2929, 2854, 1680, 1598, 1529, 1504, 1450, 1246.

HRMS (ESI-) Calculated for: $C_{19}H_{27}N_2OSi$ [M-H]$^-$: 327.1898, found: 327.1899.

Characterization Data for 20b:

R_f = 0.35 (1:5 EtOAc: hexanes)

TLC Visualization: Visible under 254 nm UV light; stains with basic $KMnO_4$ solution.

^{1}H NMR (400 MHz; $CDCl_3$) δ: 7.18 (2H, t, *J* = 7.9 Hz), 6.92 (1H, br s), 6.85 (1H, t, *J* = 7.3 Hz), 6.77 (2H, d, *J* = 7.8 Hz), 5.93 (1H, s), 1.84-1.72 (3H, m), 1.78 (2H, s), 1.55-1.45 (3H, m), 1.28-1.16 (3H, m), 0.90-0.83 (1H, m), 0.05 (9H, s).

^{13}C{^{1}H} NMR (100 MHz; $CDCl_3$) δ: 169.9, 147.9, 140.4, 134.9, 129.2, 120.8, 113.4, 66.4, 34.1, 25.2, 24.0, 15.8, -1.53.

FTIR ν_{max} (ATR, film)/cm^{-1} 3263 (broad), 2930, 2857, 1693, 1626, 1603, 1497, 1453, 1247.

HRMS (ESI+) Calculated for: $C_{19}H_{29}N_2OSi$ [M+H]$^+$: 329.2044, found: 329.2045.

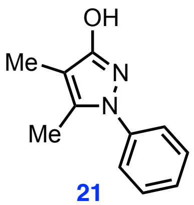

21

Following **General Procedure C**, the reaction was carried out using the *N*-acylazo derivative **19** (24.4 mg, 0.13 mmol, 1.0 equiv.), TFA (15.0 µL, 0.20 mmol, 1.5 equiv.), and 2.0 mL of anhydrous CH_2Cl_2. The crude product was purified with flash column chromatography using a solvent system of 1:5 → 1:3 → 1:1 EtOAc:hexanes → 1:3 → 1:1 CH_2Cl_2:hexanes → only CH_2Cl_2 → 1:1 CH_2Cl_2:EtOAc. Compound **21** was obtained as a white solid (23.6 mg, 97% yield).

***R**$_f$* = 0.57 (1:3 EtOAc: hexanes)

TLC Visualization: Visible under 254 nm UV light; stains with basic $KMnO_4$ solution.

^{1}H NMR (400 MHz; DMSO-*d*$_6$) δ: 9.86 (1H, br s), 7.46-7.41 (4H, m), 7.29-7.24 (1H, m), 2.20 (3H, s), 1.83 (3H, s).

^{13}C{^{1}H} NMR (100 MHz; DMSO-*d*$_6$) δ: 160.5, 140.0, 136.3, 129.0, 125.7, 123.2, 100.1, 11.2, 6.7.

FTIR ν_{max} (ATR, film)/cm^{-1} 3060, 2921, 2861, 2652, 2586, 1598, 1536, 1510, 1348, 1320, 1265, 1209.

HRMS (ESI+) Calculated for: $C_{11}H_{13}N_2O$ $[M+H]^+$: 189.1022, found: 189.1028.

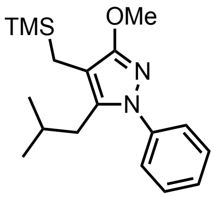

26

In a 25-mL oven-dried Schlenk flask, the hydroxypyrazole **12aa** (30.0 mg, 0.099 mmol, 1.0 equiv.) and K_2CO_3 (27.6 mg, 0.20 mmol, 2.0 equiv.) were dissolved in 2.4 mL of acetone after 3 cycles of vacuum-N_2. Then, Me_2SO_4 (11 µL, 0.12 mmol, 1.2 equiv.) was added and the flask was immersed in an oil bath at 64 °C. After stirring the reaction mixture for 2 h 13 min, 3 mL of $NH_4Cl_{(satd.)}$ was added as aqueous work-up. The aqueous layer was extracted thrice with EtOAc. The organic layers were combined, dried over anhydrous Na_2SO_4, and concentrated *in vacuo*.

Flash column chromatography was performed with a system of 1:9 EtOAc:hexanes. Compound **26** was obtained as a beige oil (20.5 mg, 66% yield).

R_f = 0.56 (1:9 EtOAc: hexanes)

TLC Visualization: Visible under 254 nm UV light; stains with basic $KMnO_4$ solution.

^{1}H NMR (400 MHz; $CDCl_3$) δ: 7.43-7.36 (4H, m), 7.30-7.27 (1H, m), 3.92 (3H, s), 2.45 (2 H, d, J = 7.4 Hz), 1.68 (2H, s), 1.57 (1H, n, J = 6.8 Hz), 0.73 (6H, d, J = 6.6 Hz), 0.03 (9H, s).

^{13}C{^{1}H} NMR (100 MHz; $CDCl_3$) δ: 162.4, 141.2, 139.7, 129.1, 126.8, 125.4, 103.1, 55.7, 34.1, 29.9, 28.2, 22.4, 11.2, -1.16.

FTIR ν_{max} (ATR, film)/cm^{-1} 2954, 2869, 1597, 1579, 1510, 1462, 1415, 1383.

HRMS (APCI+) Calculated for: $C_{18}H_{29}N_2OSi$ $[M+H]^+$: 317.2044, found: 317.2044.

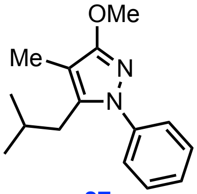

27

In an oven-dried 25-mL Schlenk flask, after three cycles of vacuum-N_2, the methoxypyrazole **26** (25.6 mg, 0.081 mmol, 1.0 equiv.) was dissolved in 1.5 mL of anhydrous THF. Then TBAF (0.22 mL, 0.22 mmol, 2.7 equiv.) was added, and after immersing the flask in an oil bath at 60 °C, the reaction mixture was stirred at this temperature for 8 h 45 min. Once the reaction flask was brought to room temperature, 2 mL of $NH_4Cl_{(satd.)}$ was added as aqueous work-up. The aqueous layer was extracted thrice with EtOAc. The organic layers were combined, dried over anhydrous Na_2SO_4, and concentrated *in vacuo*. Flash column chromatography was performed with a system of 1:9 EtOAc:hexanes. Compound **27** was obtained as a beige oil (17.6 mg, 89% yield).

R_f = 0.44 (1:9 EtOAc: hexanes)

TLC Visualization: Visible under 254 nm UV light; stains with basic $KMnO_4$ solution.

^{1}H NMR (400 MHz; $CDCl_3$) δ: 7.44-7.34 (4H, m), 7.30 (1H, t, J = 7.0 Hz), 3.96 (3H, s), 2.47 (2H, d, J = 7.4 Hz), 1.92 (3H, s), 1.66 (1H, n, J = 6.8 Hz), 0.77 (6H, d, J = 6.6 Hz).

^{13}C{^{1}H} NMR (100 MHz; $CDCl_3$) δ: 162.7, 141.6, 140.8, 129.1, 127.1, 125.8, 100.4, 55.9, 33.8, 28.5, 22.4, 7.1.

FTIR ν_{max} (ATR, film)/cm^{-1} 2953, 2930, 1596, 1515, 1458, 1415, 1379.

HRMS (ESI+) Calculated for: $C_{15}H_{21}N_2O$ $[M+H]^+$: 245.1648, found: 245.1650.

Monitoring the Conversion of 10aa to 12aa in the Absence of an Acid by ^{1}H NMR Spectroscopy:

5.0 mg of compound **10aa** was dissolved in 0.6 mL of $CDCl_3$, and the solution was transferred to an NMR tube. Prior to the experiment, $CDCl_3$ was passed through solid K_2CO_3 to remove any traces of acid. ^{1}H NMR spectra of the sample were recorded regularly over 12 days. The NMR tube was wrapped in aluminium foil and kept in a dark cabinet between the measurements.

Table S1. Time dependence of the background reaction of **10aa** in $CDCl_3$

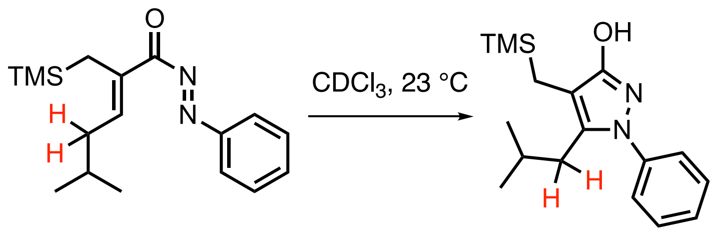

10aa **12aa**

Time (h)	^{1}H NMR signal integration ratio (product/reactant)*	Conversion (%) of **10aa** to **12aa**
0	0.14	12
9	0.18	15
24	0.26	21
48	0.37	27
72	0.52	34
96	0.67	40
145	1.07	52
168	1.29	56
192	1.48	60
264	1.74	63.5
289	1.81	64.4

* These ratios were determined by comparing the integration values of the doublet signal of **12aa** at 2.46 ppm, and the triplet signal of **10aa** at 2.13 ppm.

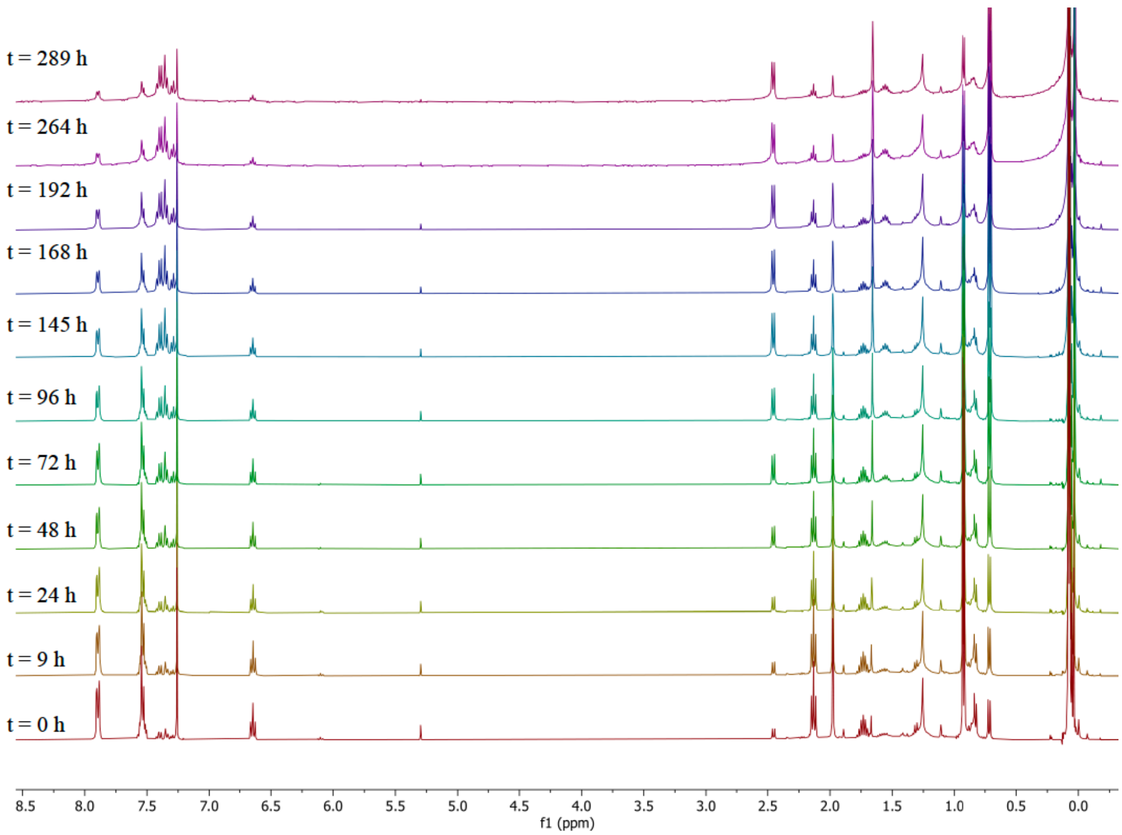

Figure S1. Stacked ^{1}H NMR spectra for the time dependence of the conversion of **10aa** to **12aa**.

^{1}H NMR Spectra of 12aa in Acetone-d_6 Before and After the Addition of D_2O:

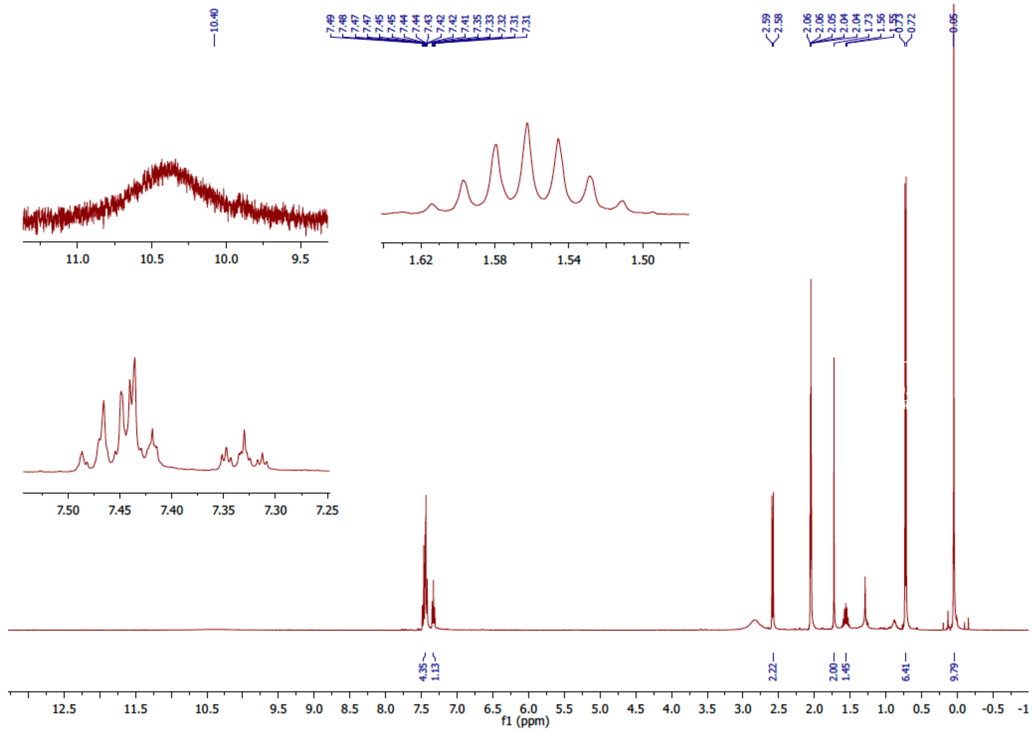

Figure S2. ^{1}H NMR spectrum of compound **12aa** in acetone-d_6 (400 MHz).

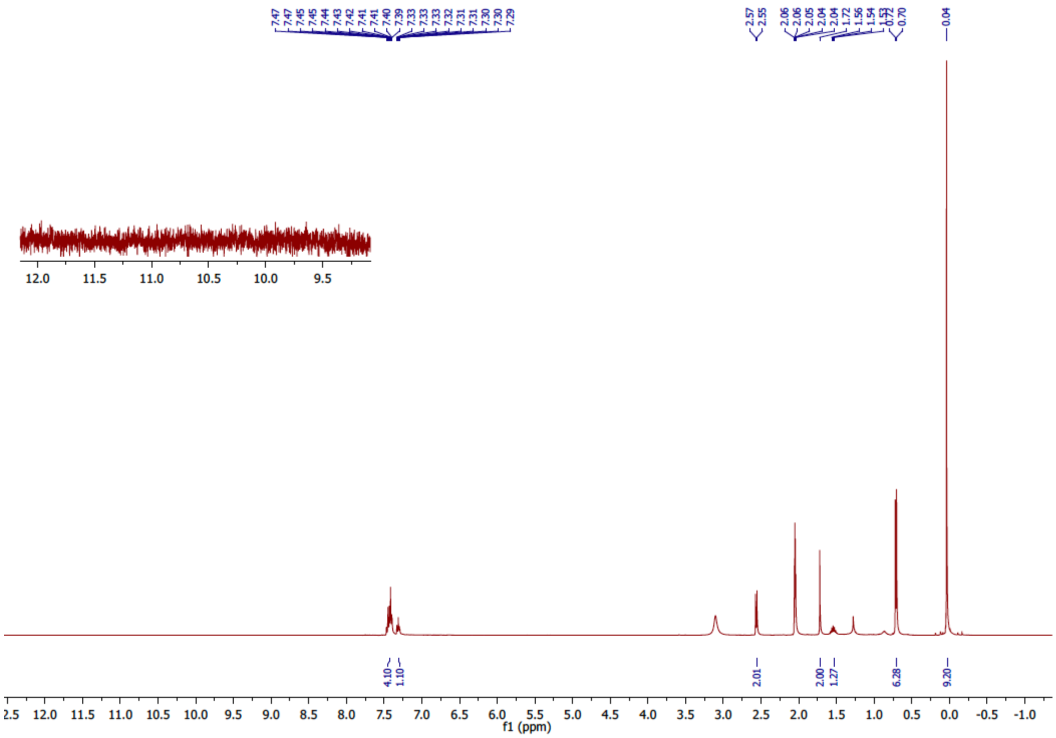

Figure S3. ^{1}H NMR spectrum of **12aa** in acetone-d_6 after the addition of a few drops of D_2O.

Electrostatic Potential Map of a Model *N*-Acylazo Derivative:

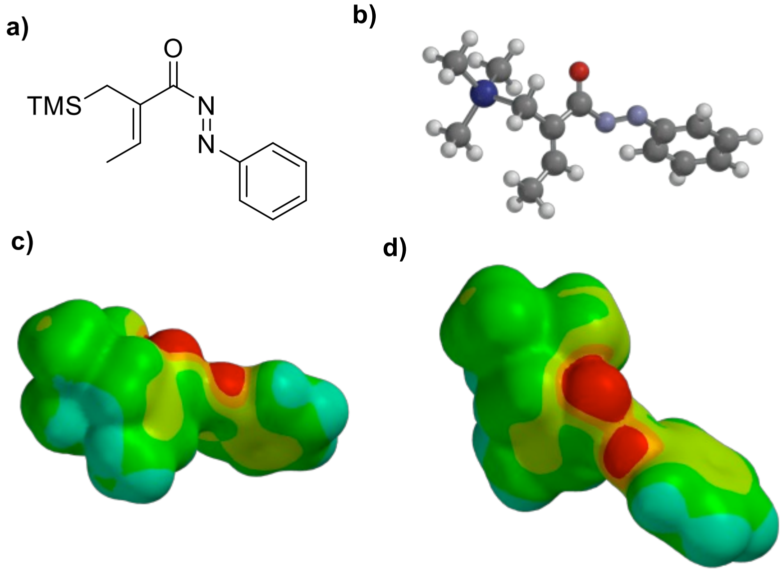

Figure S4. a) The structure of the model *N*-acylazo derivative; **b)** Optimized lowest potential energy conformation calculated using B3LYP/def2-svp level of theory;[4-7] **c** and **d)** Views of the electrostatic potential map of the model compound from different angles.

Based on the calculated electrostatic potential map, the atom with the most negative electrostatic potential was found to be the carbonyl oxygen with a value of -272.1 kJ/mol compared to those of N1 and N2, which have electrostatic potential values of -207.7 kJ/mol and -175.4 kJ/mol, respectively.

References

1. Donmez, S. E.; Soydaş, E.; Aydın, G.; Şahin, O.; Bozkaya, U.; Türkmen, Y. E. *Org. Lett.* **2019**, *21*, 554.
2. Hirose, T.; Sunazuka, T.; Shirahata, T.; Yamamoto, D.; Harigaya, Y.; Kuwajima, I.; Omura, S. *Org. Lett.* **2002**, *4*, 501.
3. Kon, Y.; Nakashima, T.; Makino, Y.; Onozawa, S.; Miyamura, H.; Kobayashi, S.; Sato, K. *Org. Biomol. Chem.* **2025**, *23*, 2125.
4. (*a*) Becke, A. D. *J. Chem. Phys.*, **1993**, *98*, 1372; (*b*) Becke, A. D. *J. Chem. Phys.*, **1993**, *98*, 5648.
5. Weigend, F.; Ahlrichs, R. *Phys. Chem. Chem. Phys.*, **2005**, *7*, 3297.
6. Neese, F. *Wiley Interdiscip. Rev.:Comput. Mol. Sci.*, **2022**, e1606.
7. Allouche, A.-R. *J. Comput. Chem.*, **2011**, *32*, 174.

^{1}H, ^{13}C{^{1}H} and ^{19}F NMR spectra:

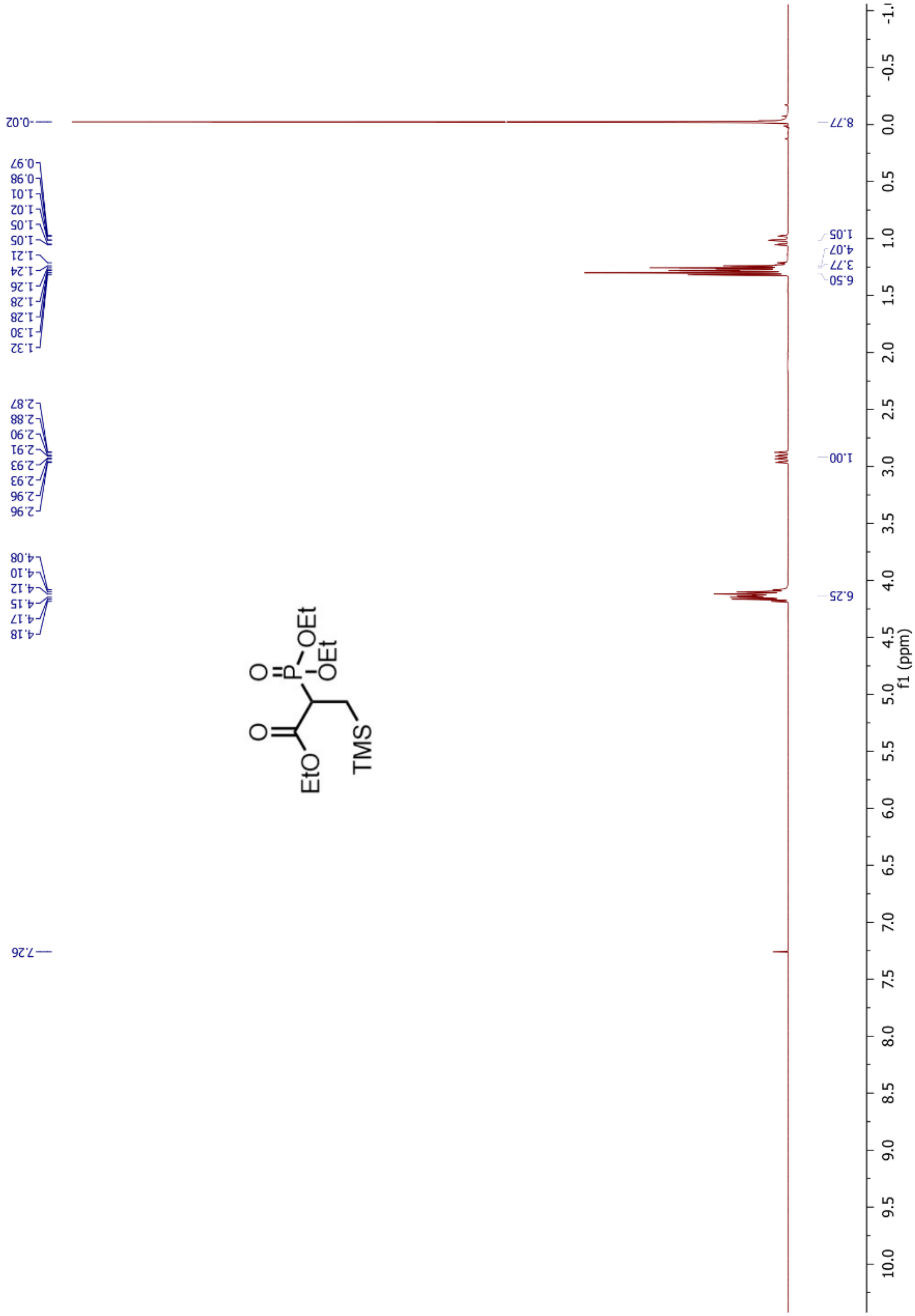

Figure S5. ^{1}H NMR spectrum of **13** in $CDCl_3$ (400 MHz).

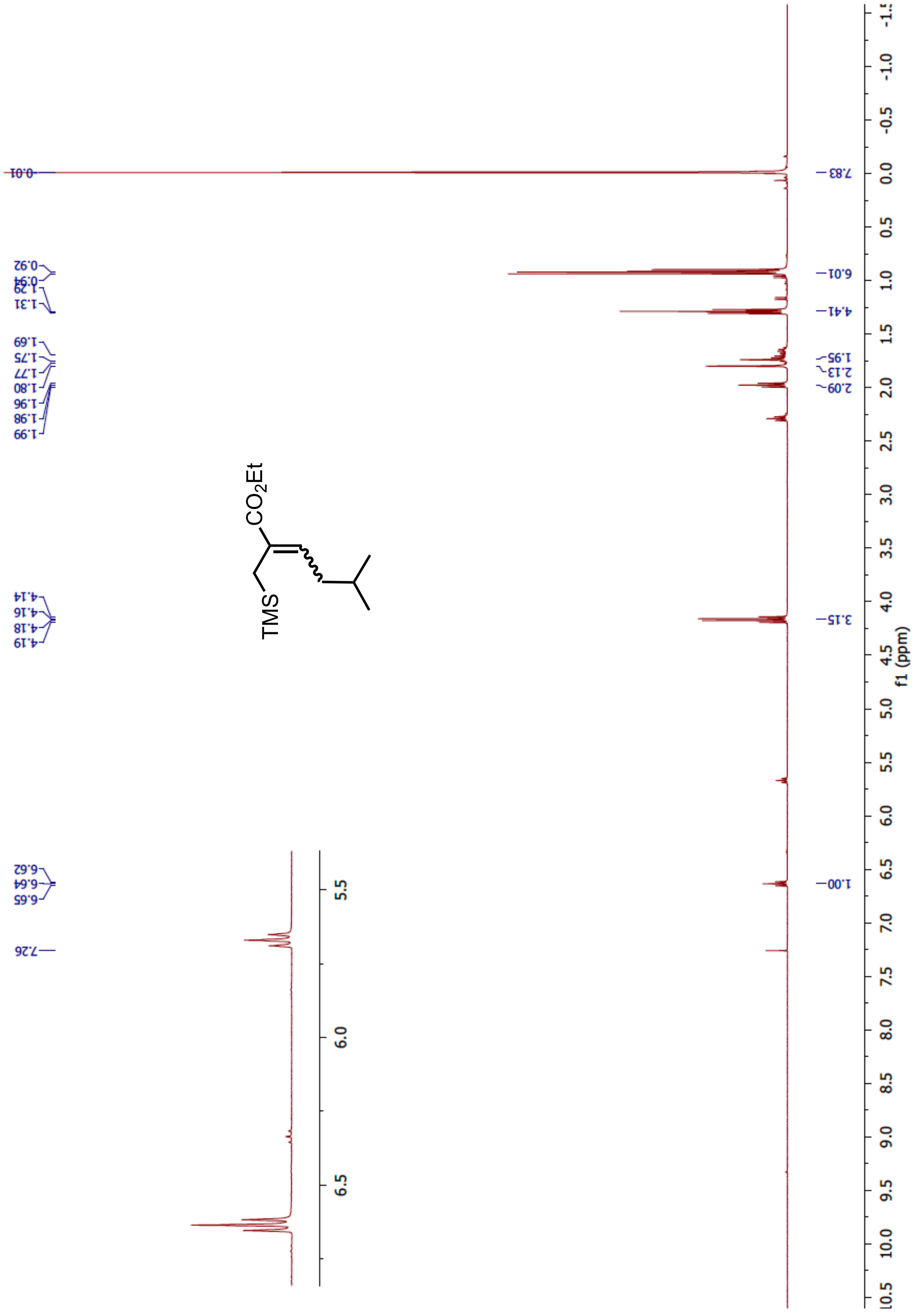

Figure S6. ^{1}H NMR spectrum of **14a** in $CDCl_3$ (400 MHz).

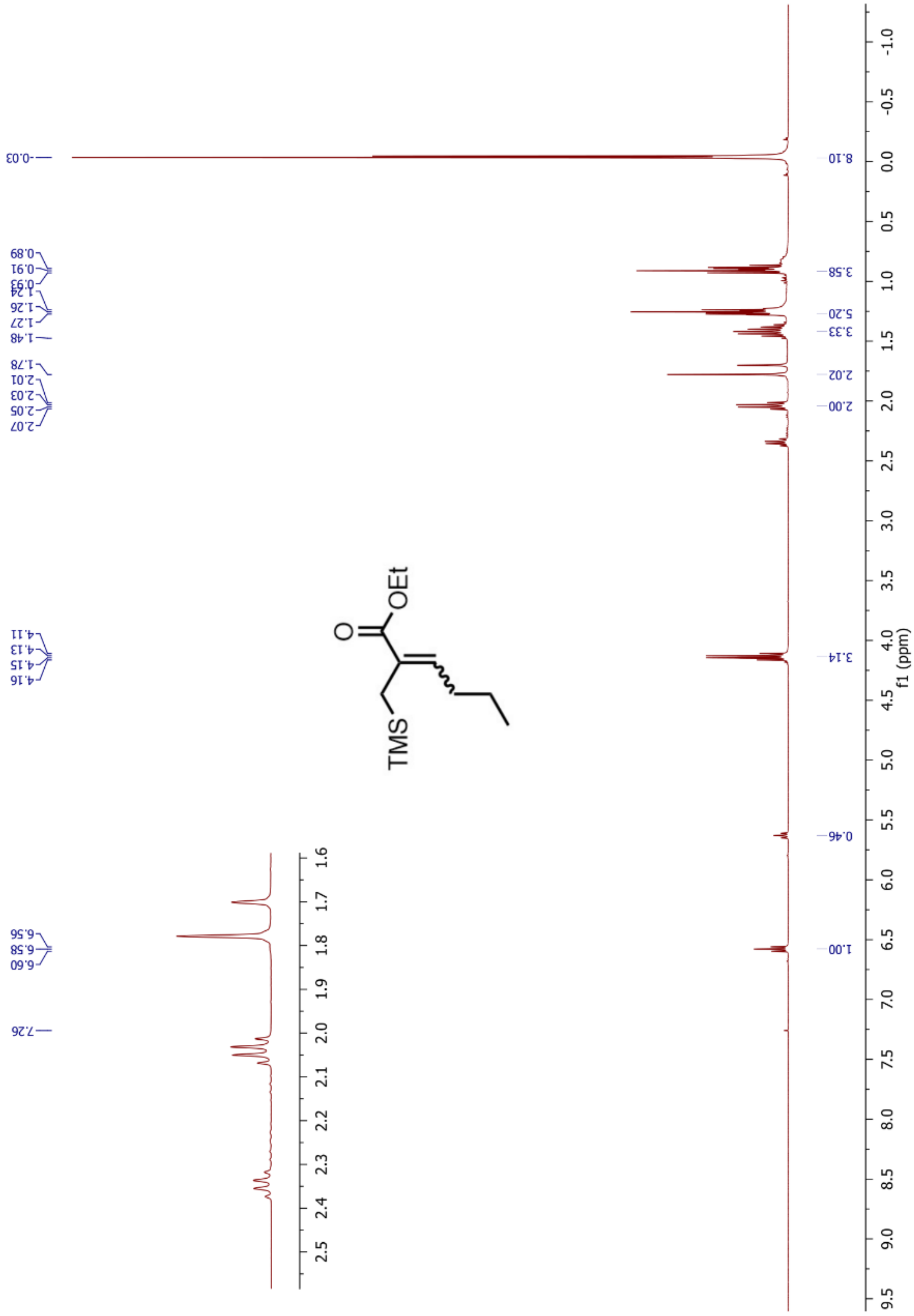

Figure S7. ^{1}H NMR spectrum of **14b** in $CDCl_3$ (400 MHz).

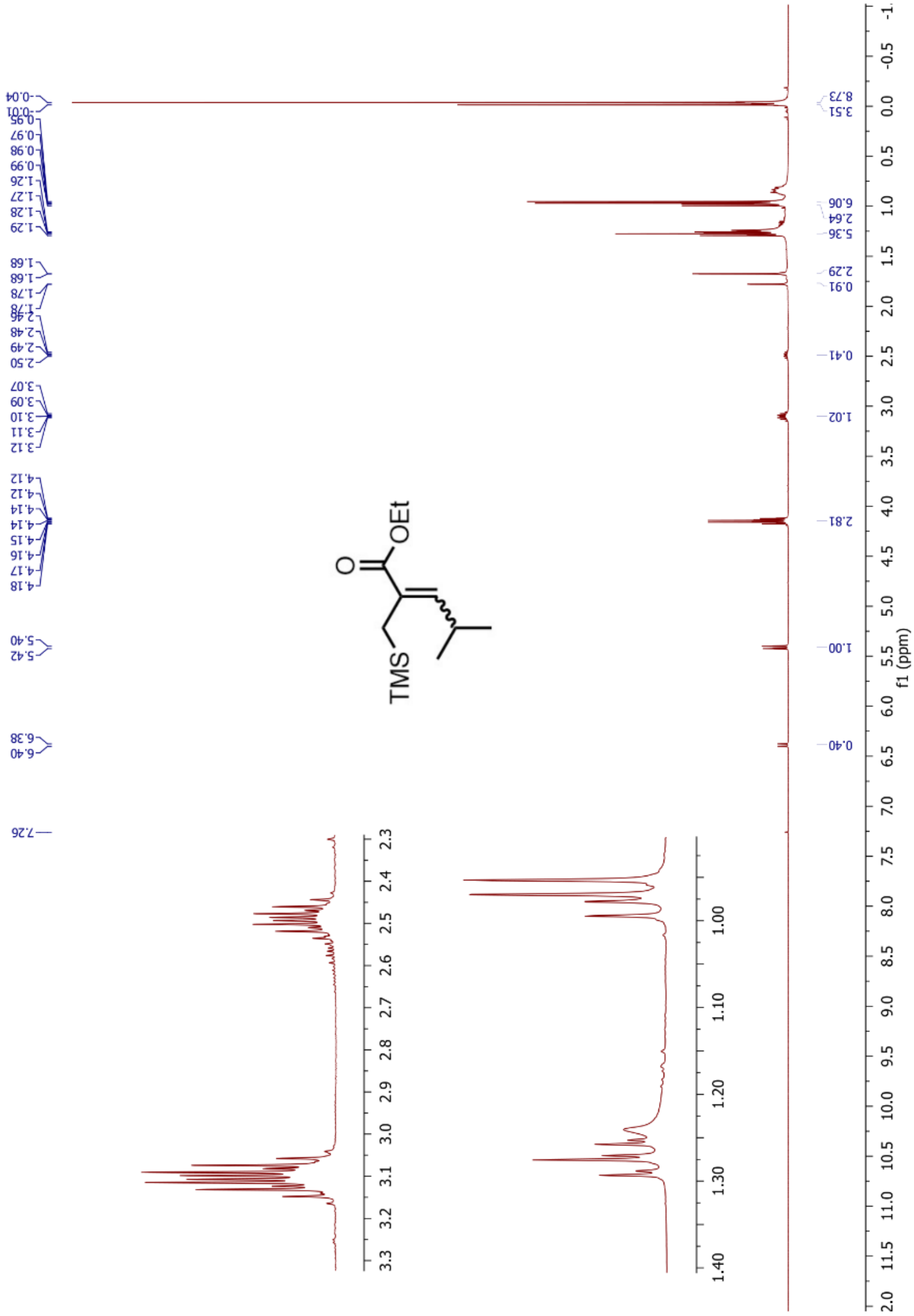

Figure S8. ^{1}H NMR spectrum of **14c** in $CDCl_3$ (400 MHz).

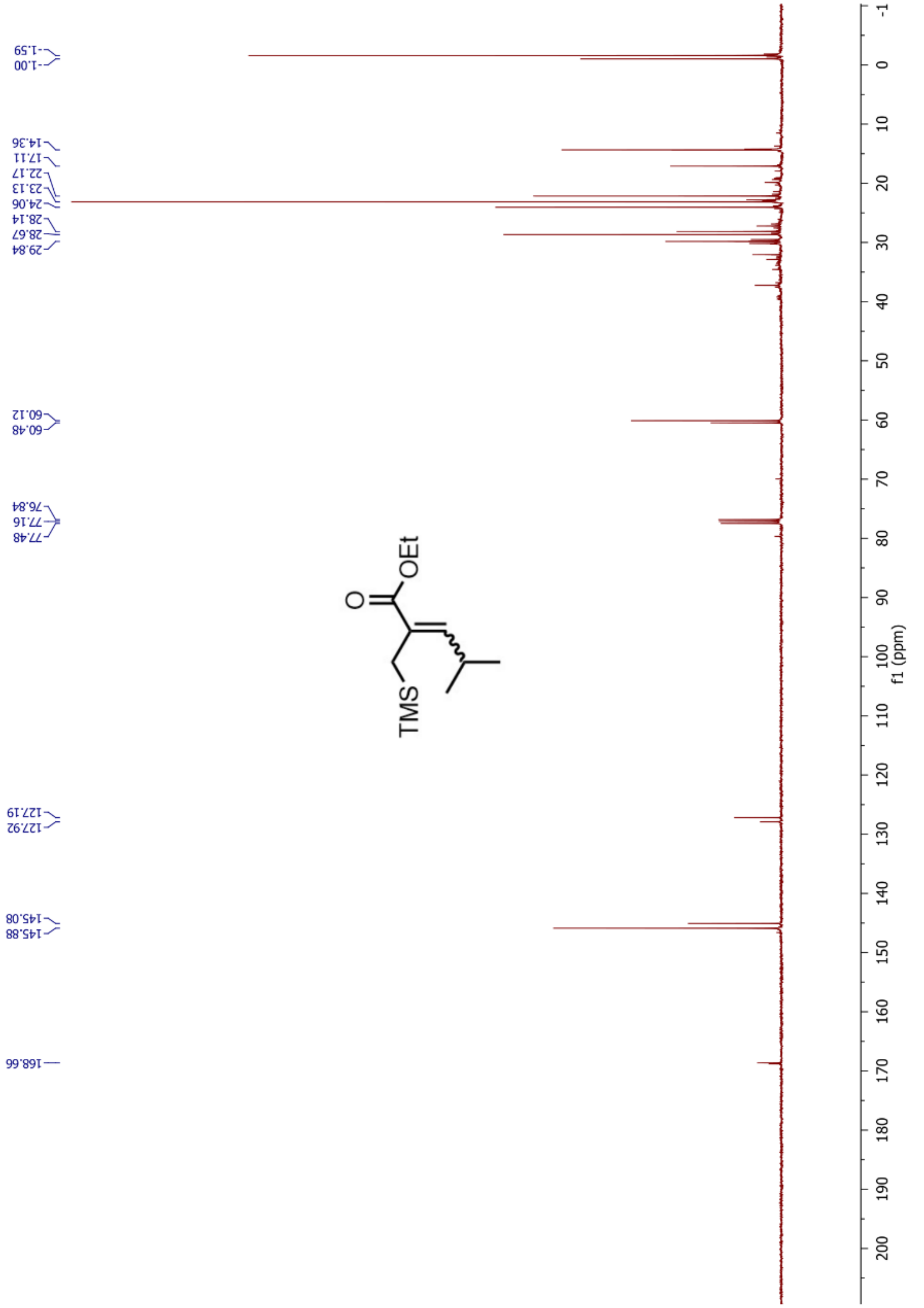

Figure S9. $^{13}C\{^{1}H\}$ NMR spectrum of **14c** in $CDCl_3$ (100 MHz).

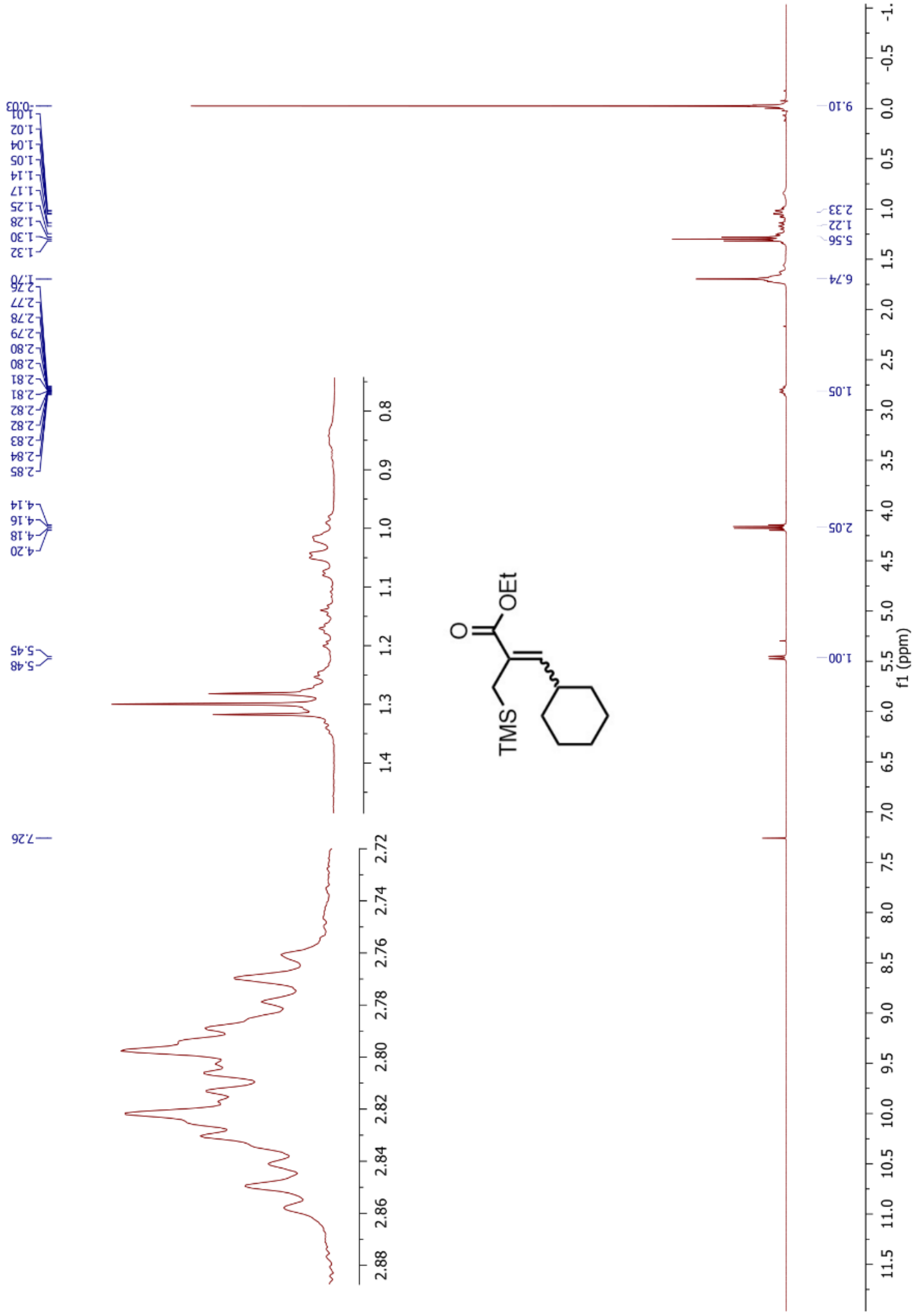

Figure S10. ^{1}H NMR spectrum of **14d** in $CDCl_3$ (400 MHz).

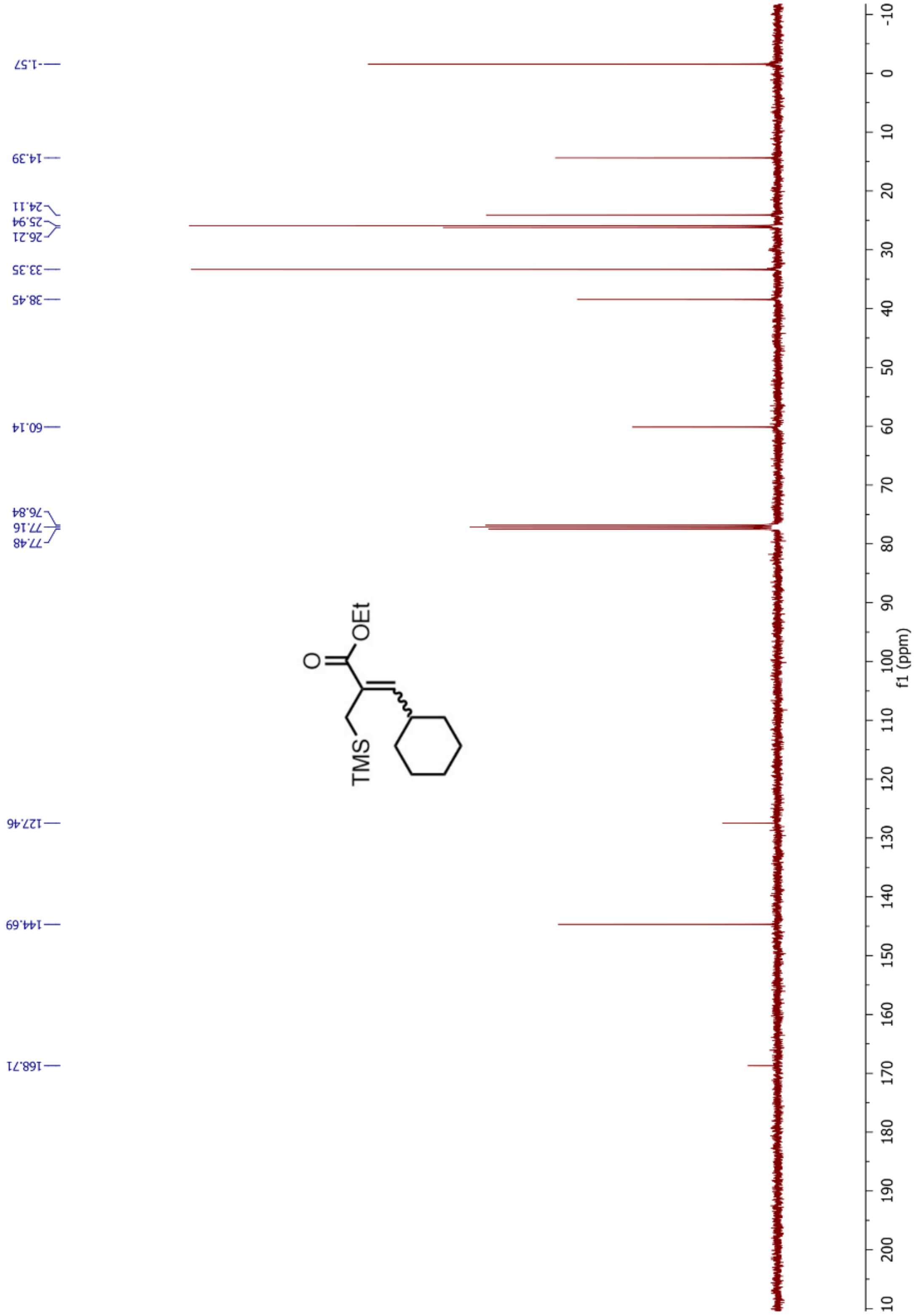

Figure S11. $^{13}C\{^{1}H\}$ NMR spectrum of **14d** in $CDCl_3$ (100 MHz).

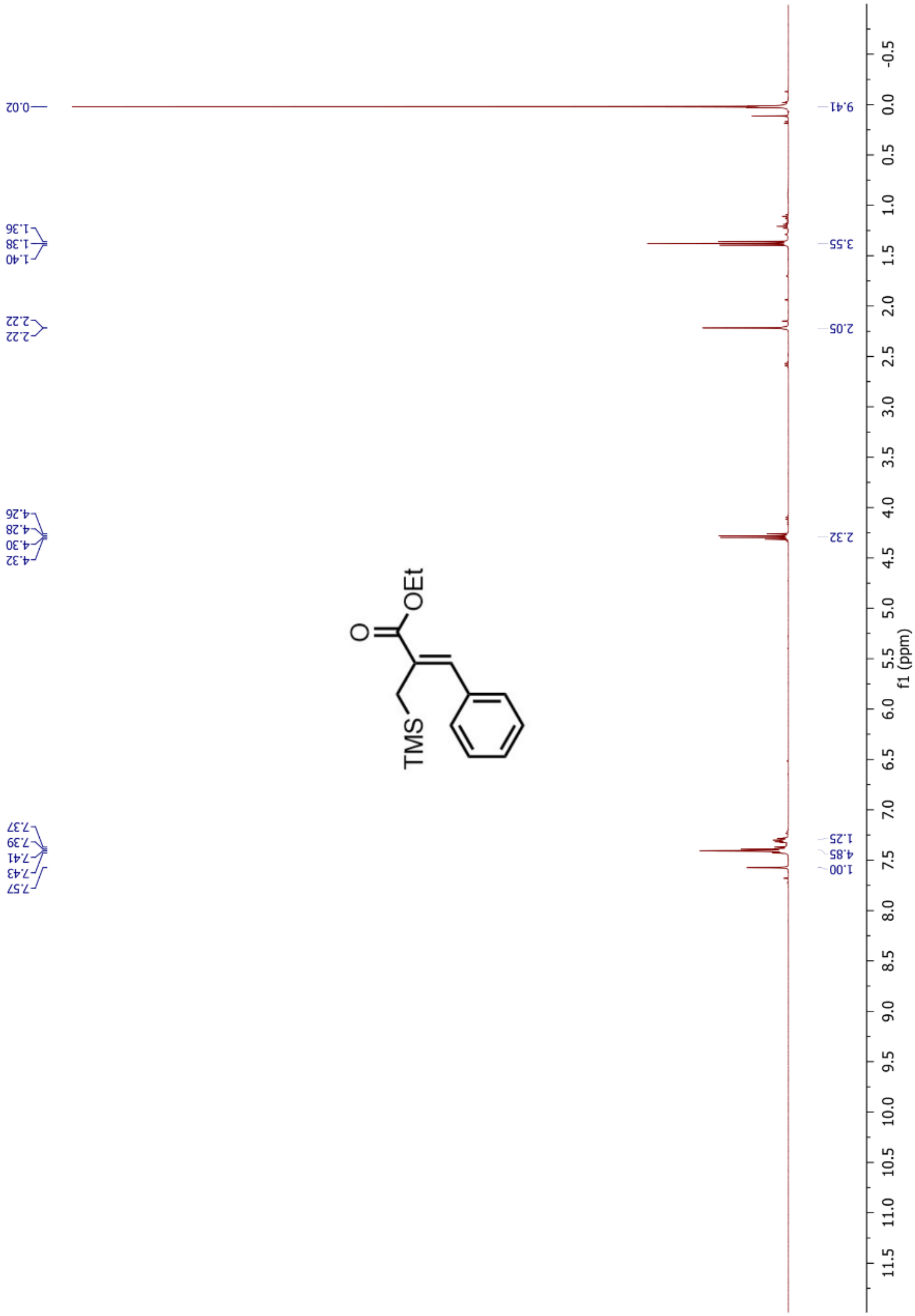

Figure S12. ^{1}H NMR spectrum of **S1** in $CDCl_3$ (400 MHz).

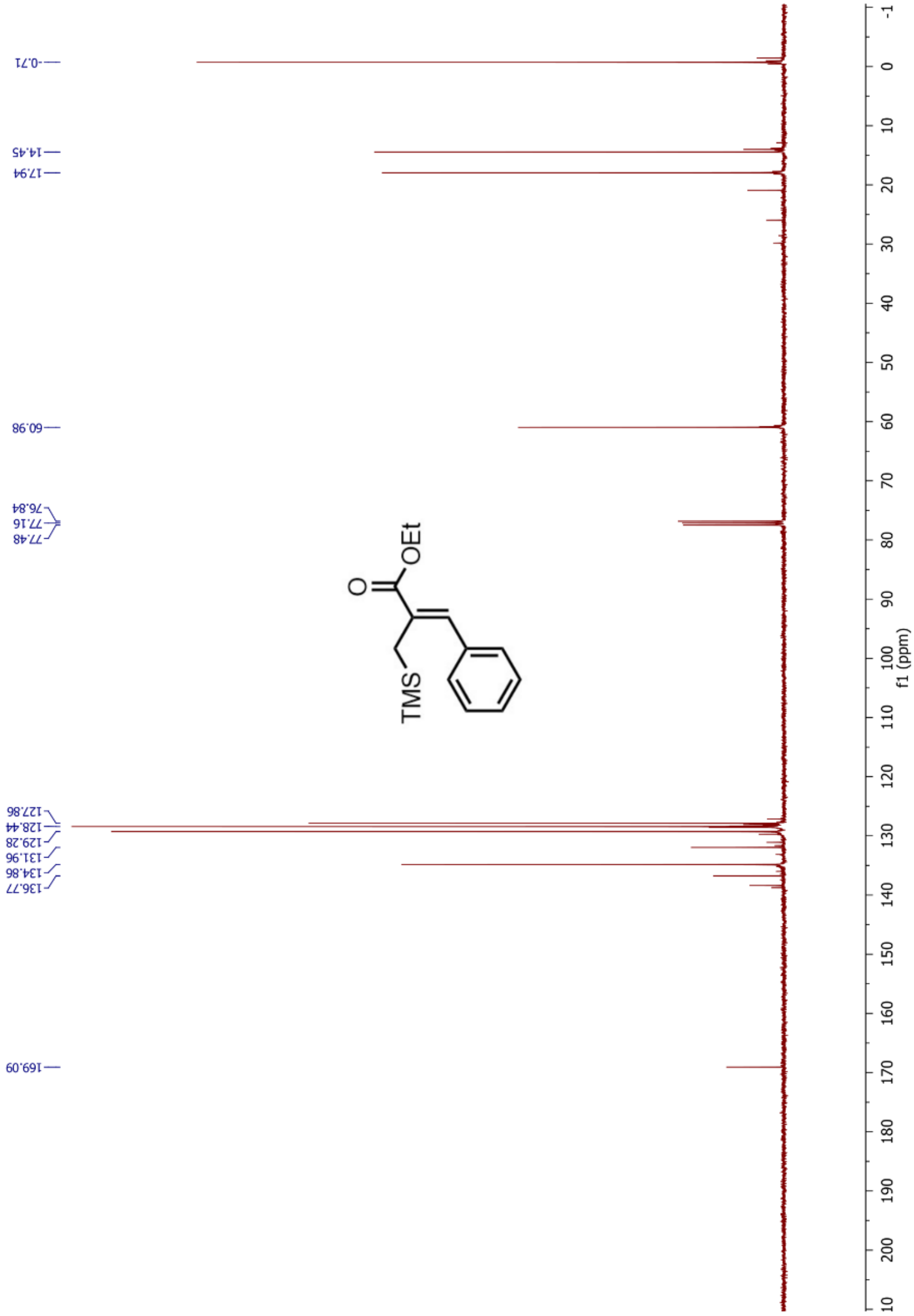

Figure S13. $^{13}C\{^{1}H\}$ NMR spectrum of **S1** in $CDCl_3$ (100 MHz).

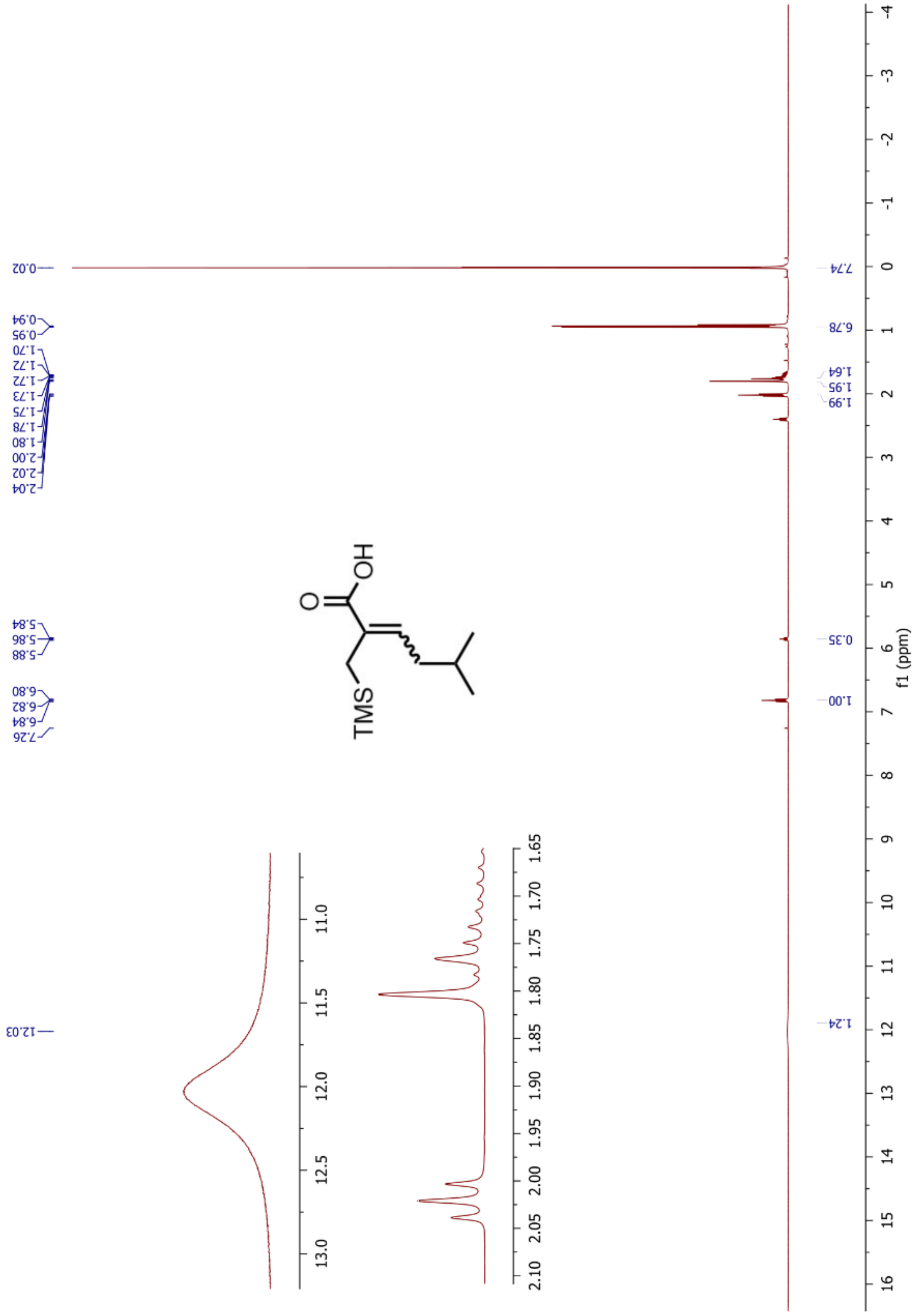

Figure S14. ^{1}H NMR spectrum of **15a** in $CDCl_3$ (400 MHz).

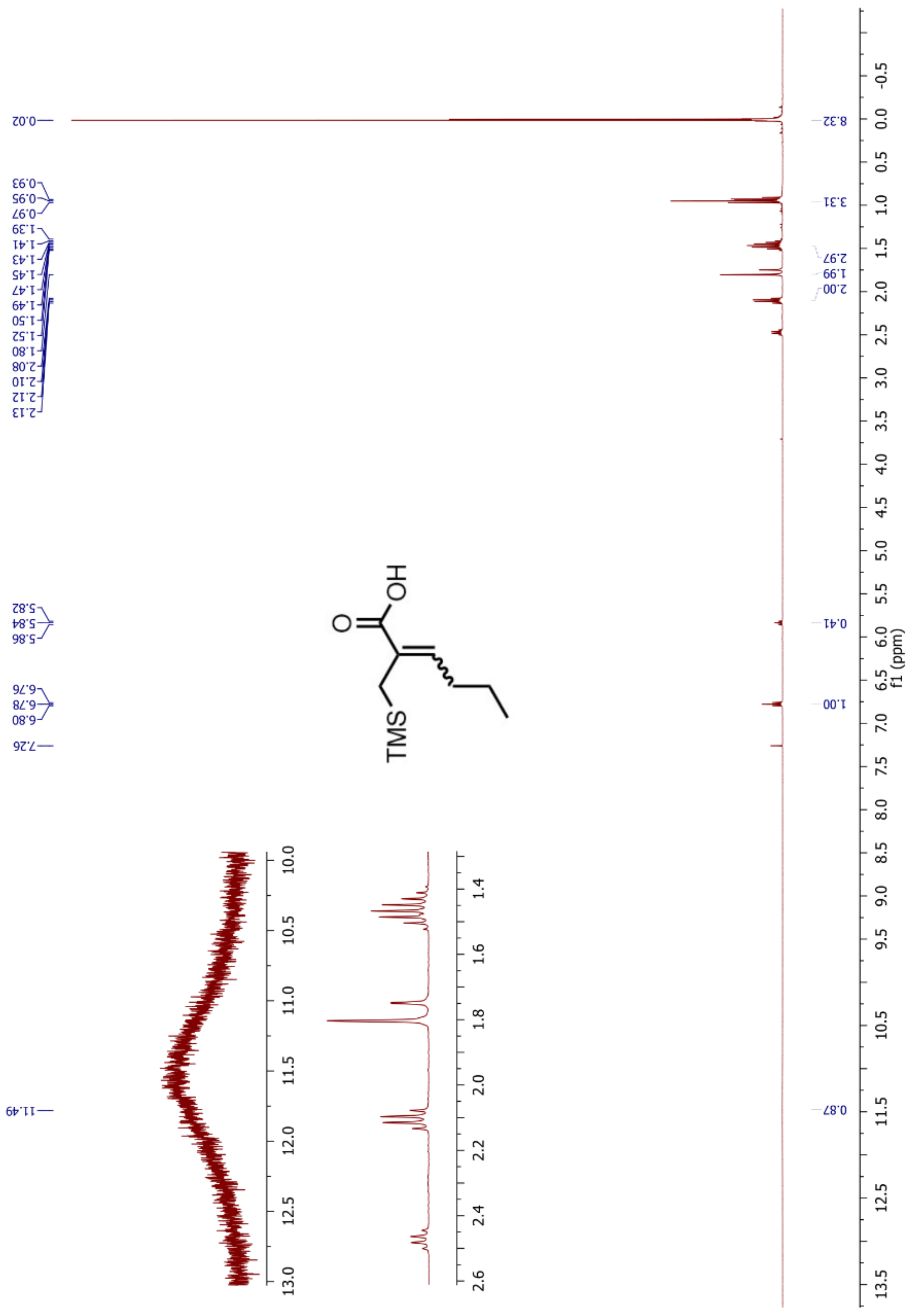

Figure S15. ^{1}H NMR spectrum of **15b** in $CDCl_3$ (400 MHz).

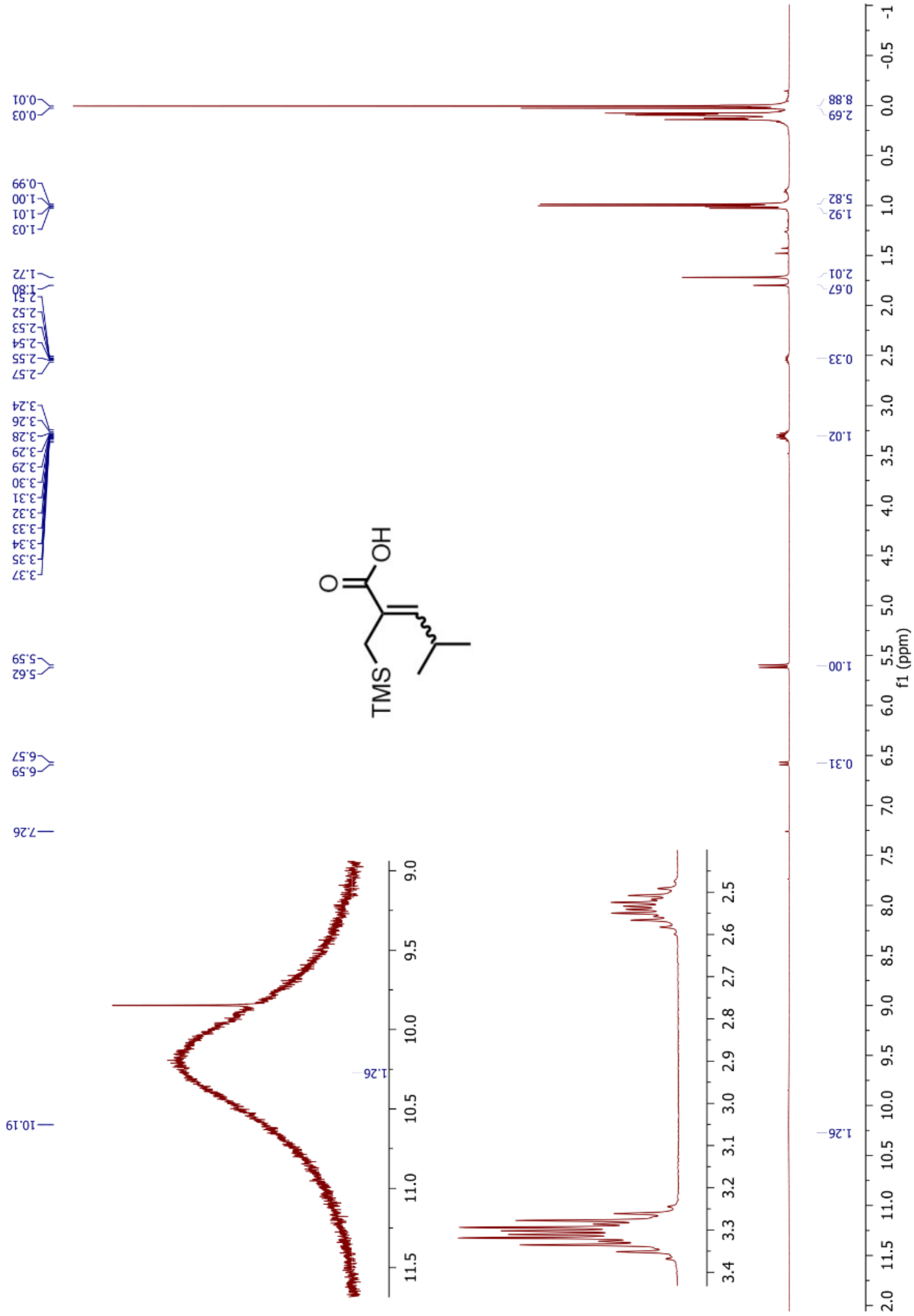

Figure S16. ^{1}H NMR spectrum of **15c** in $CDCl_3$ (400 MHz).

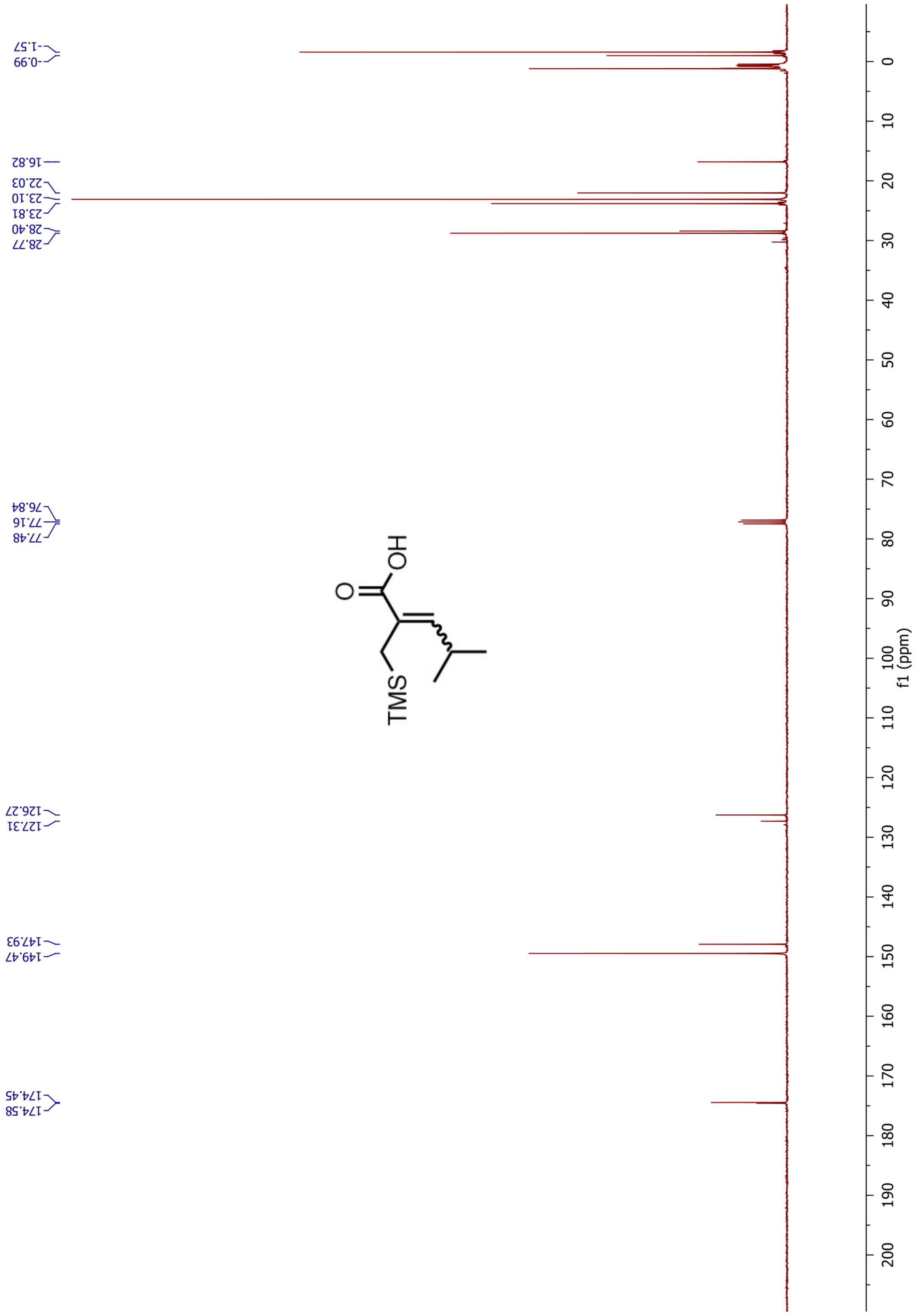

Figure S17. $^{13}C\{^{1}H\}$ NMR spectrum of **15c** in $CDCl_3$ (100 MHz).

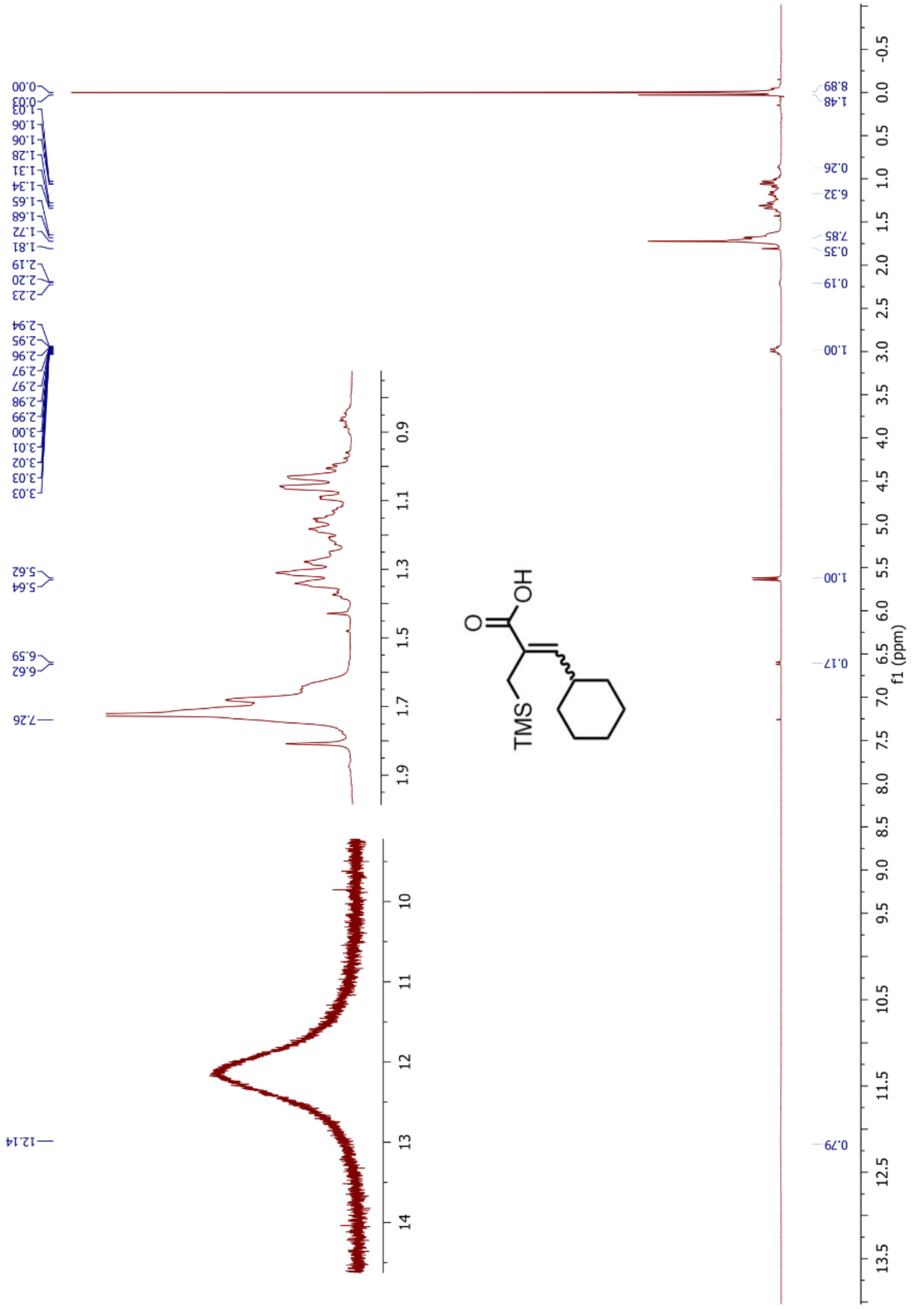

Figure S18. ^{1}H NMR spectrum of **15d** in $CDCl_3$ (400 MHz).

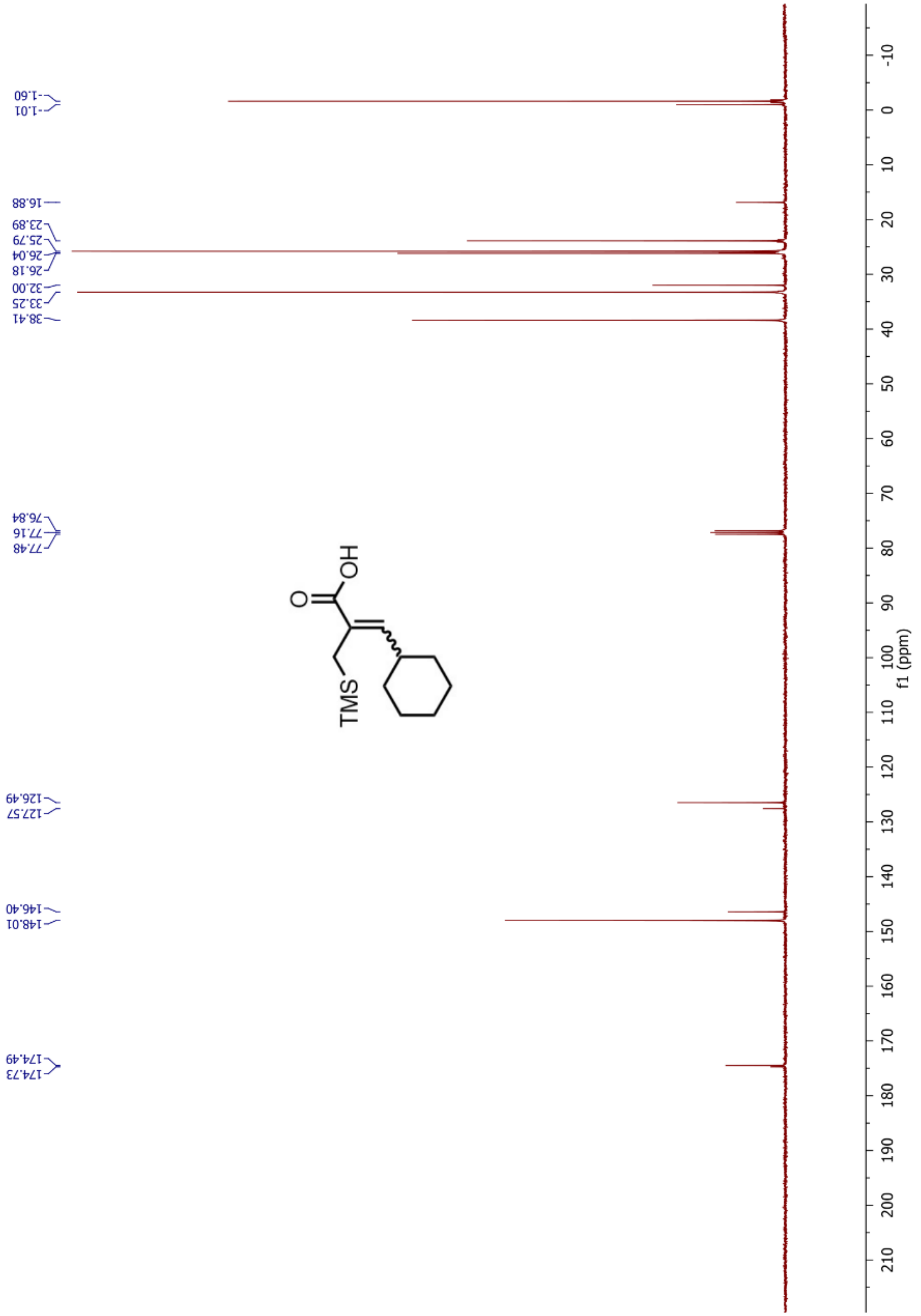

Figure S19. $^{13}C\{^{1}H\}$ NMR spectrum of **15d** in $CDCl_3$ (100 MHz).

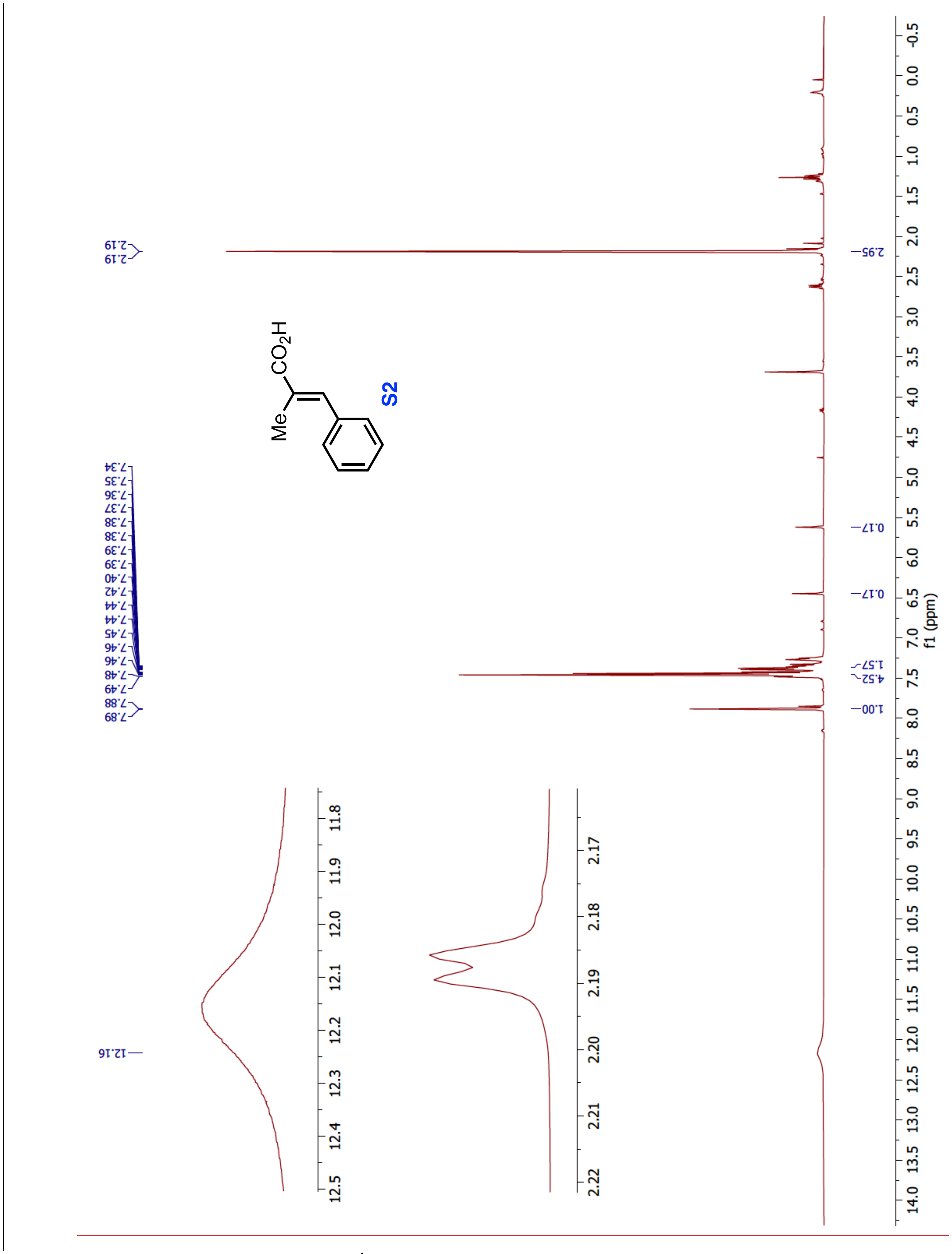

Figure S20. ^{1}H NMR spectrum of **S2** in $CDCl_3$ (400 MHz).

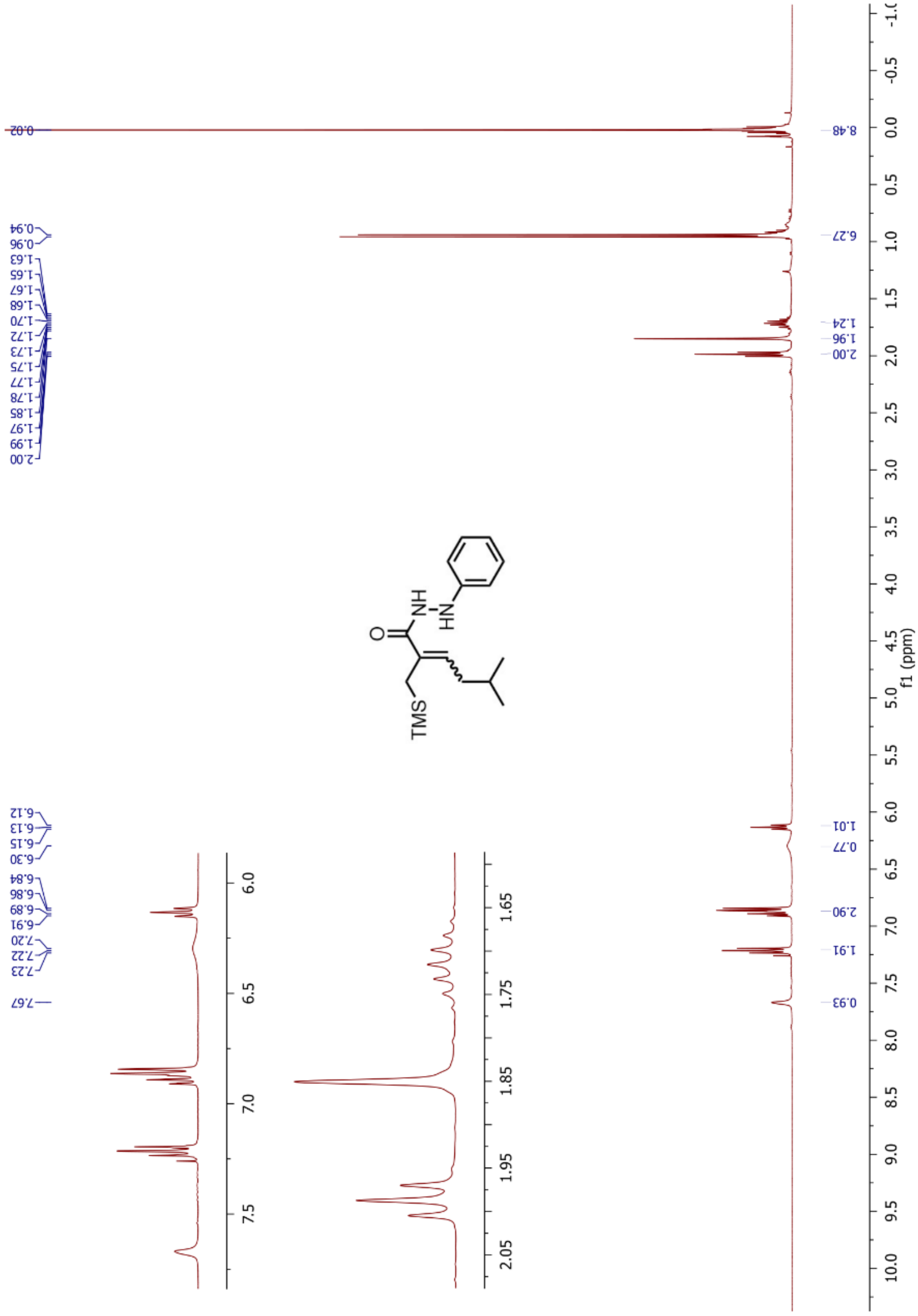

Figure S21. ^{1}H NMR spectrum of **16aa** in $CDCl_3$ (400 MHz).

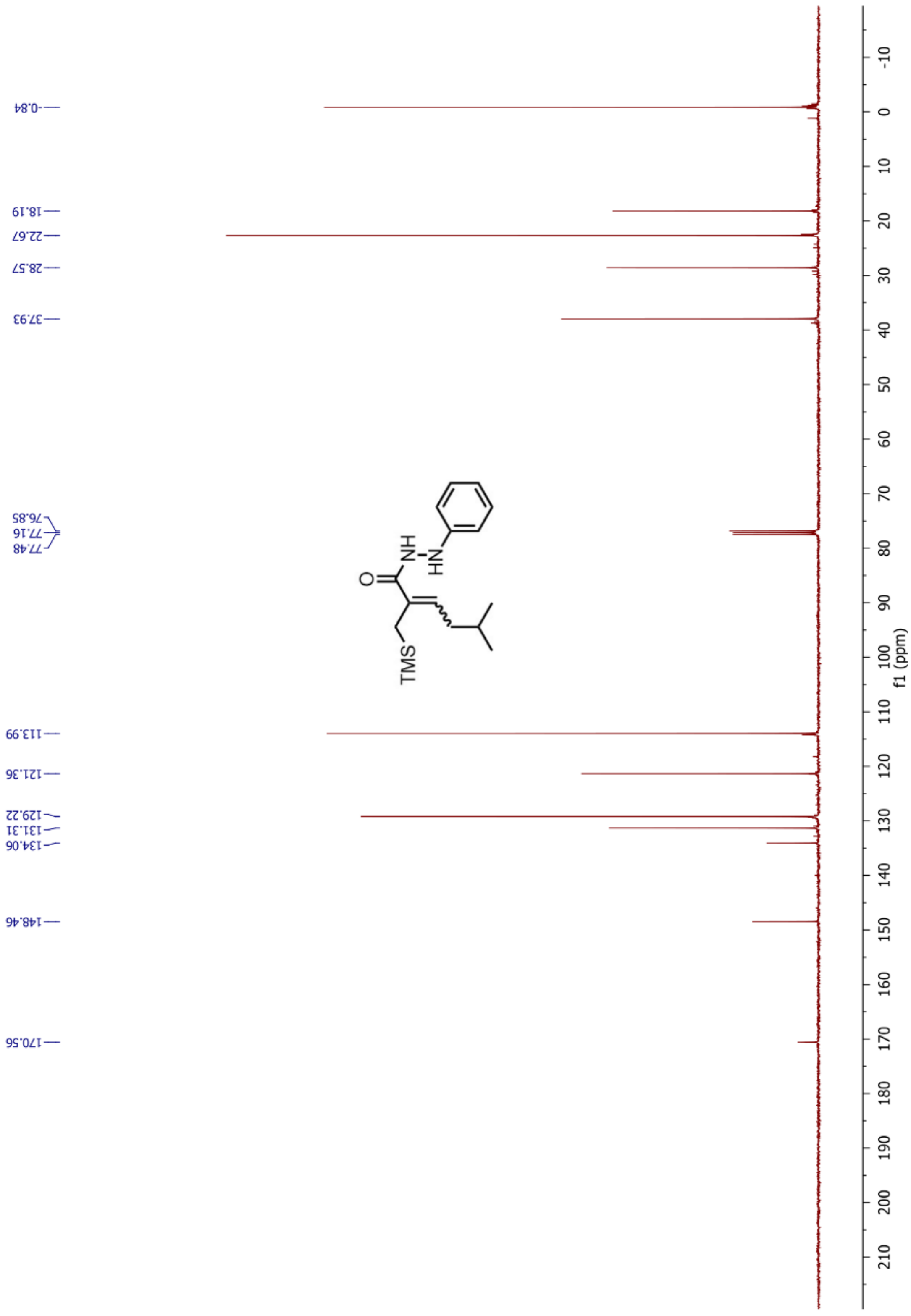

Figure S22. $^{13}C\{^{1}H\}$ NMR spectrum of **16aa** in $CDCl_3$ (100 MHz).

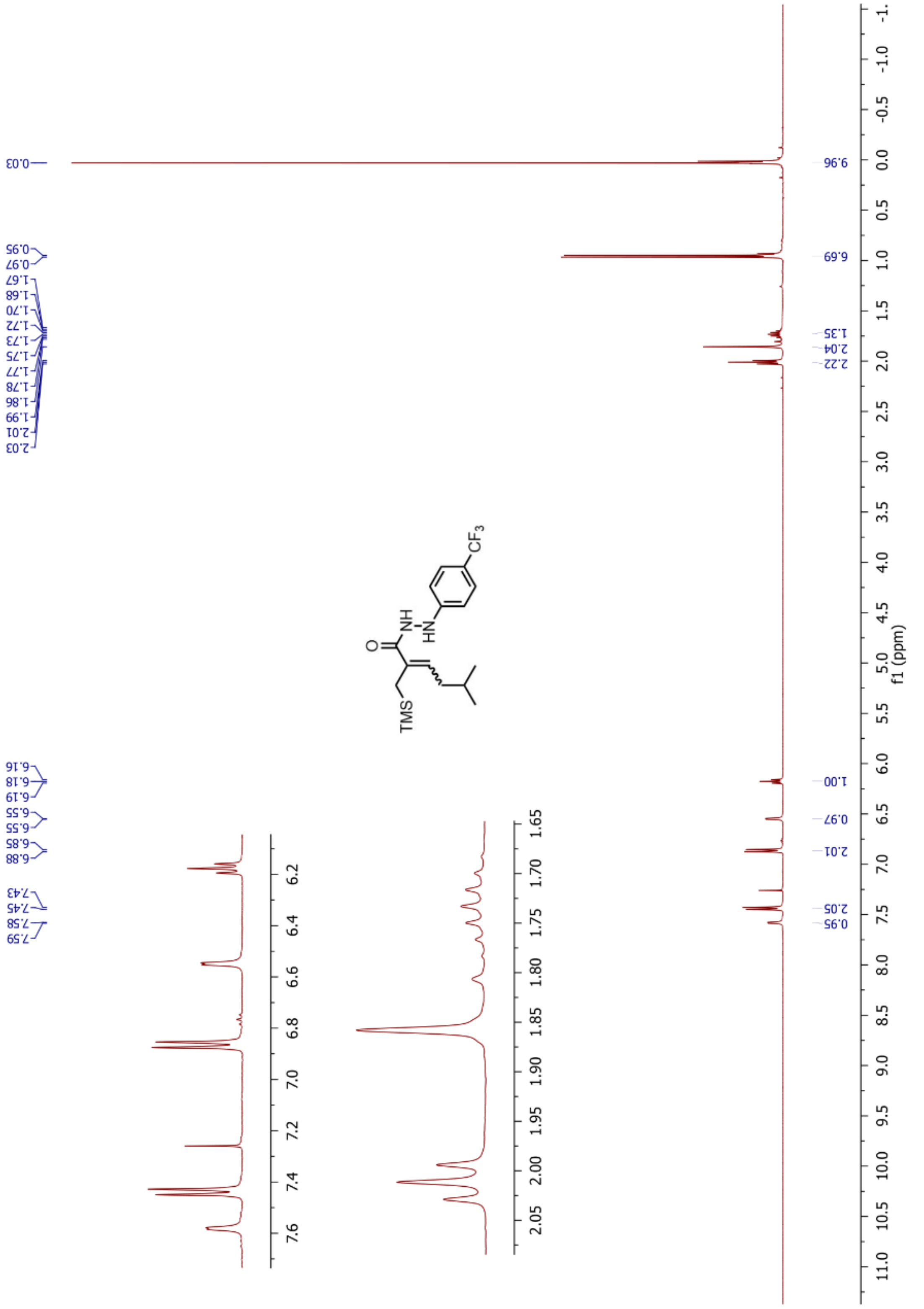

Figure S23. ^{1}H NMR spectrum of **16ab** in $CDCl_3$ (400 MHz).

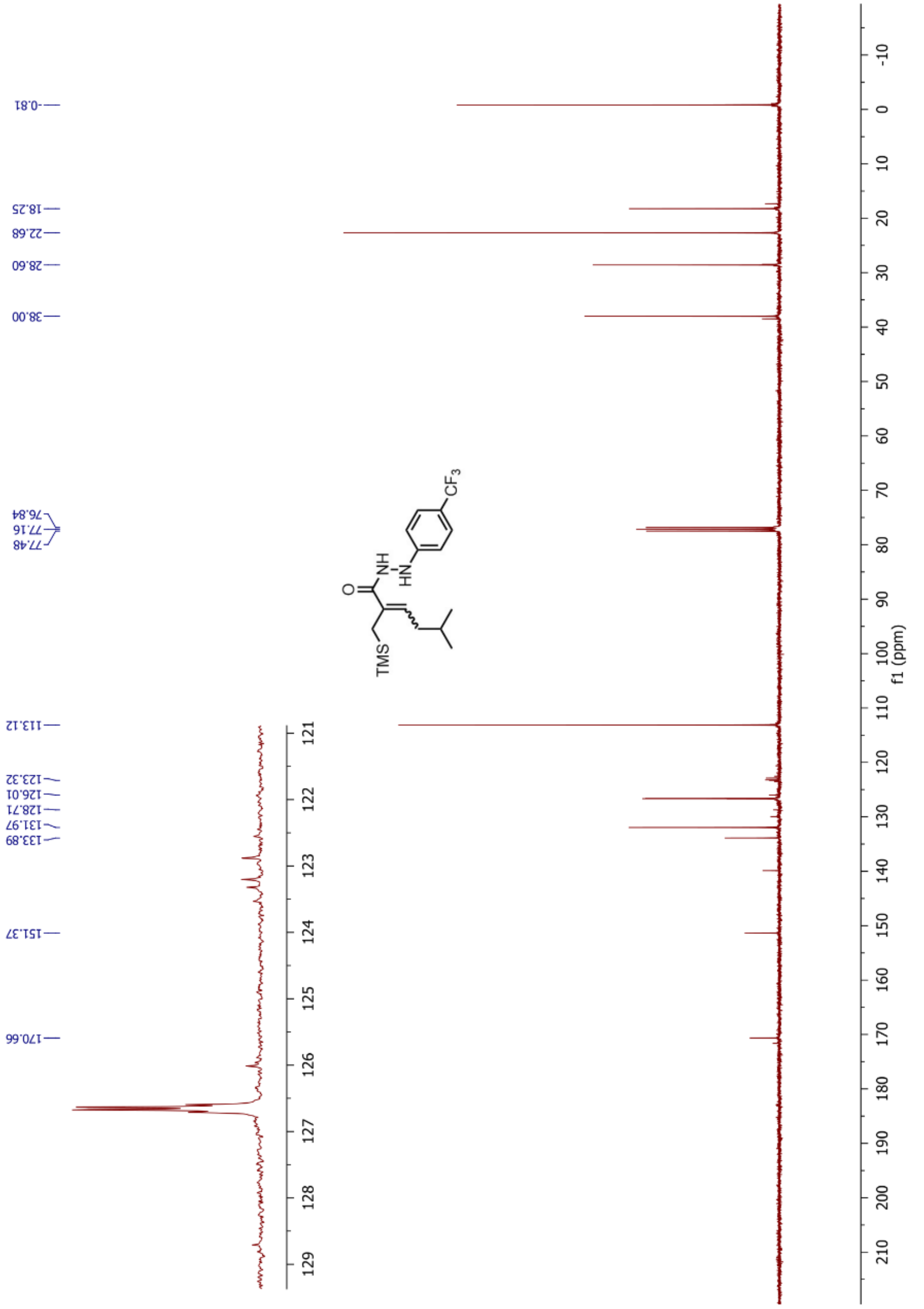

Figure S24. $^{13}C\{^{1}H\}$ NMR spectrum of **16ab** in $CDCl_3$ (100 MHz).

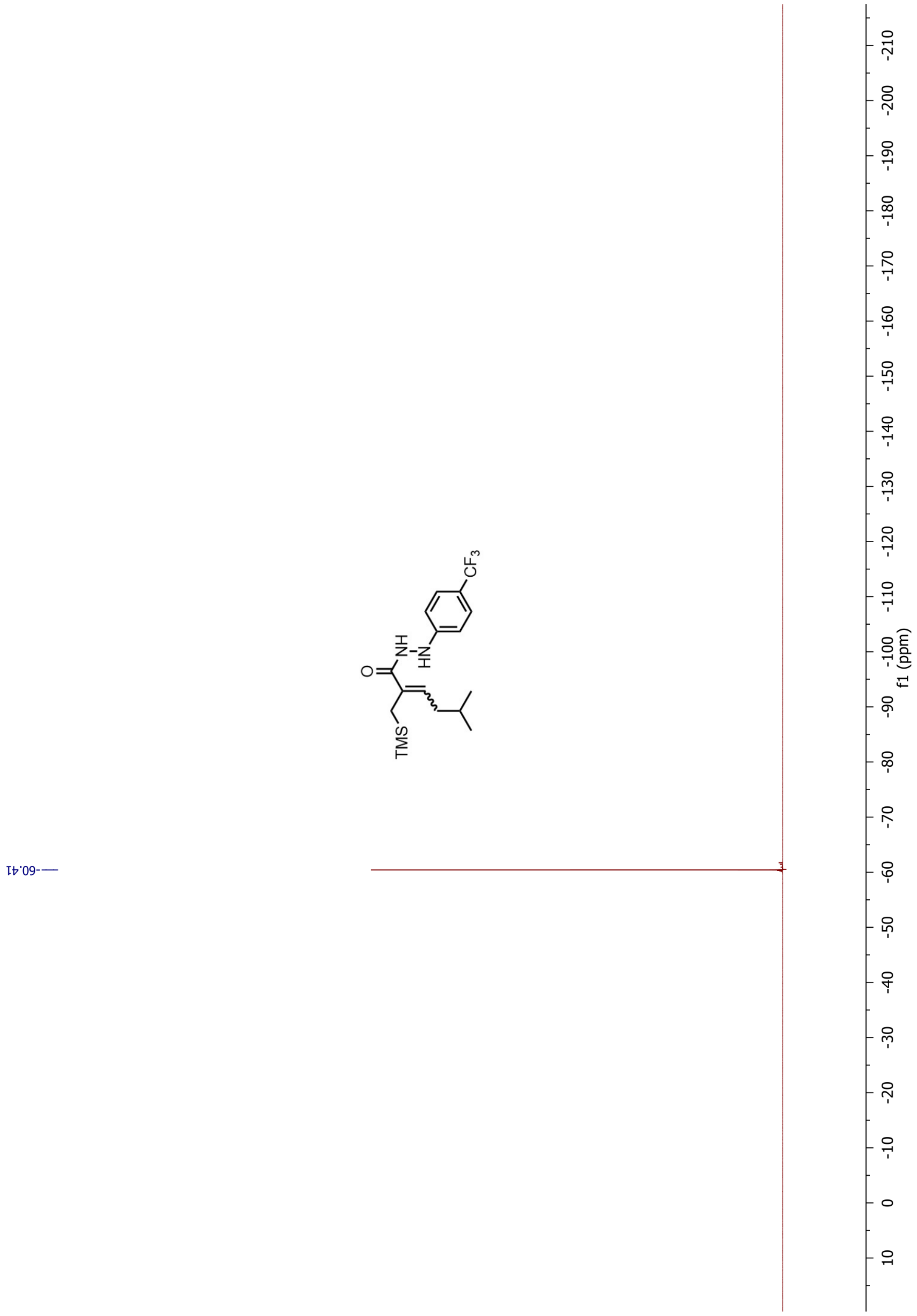

Figure S25. ^{19}F NMR spectrum of **16ab** in $CDCl_3$ (376 MHz).

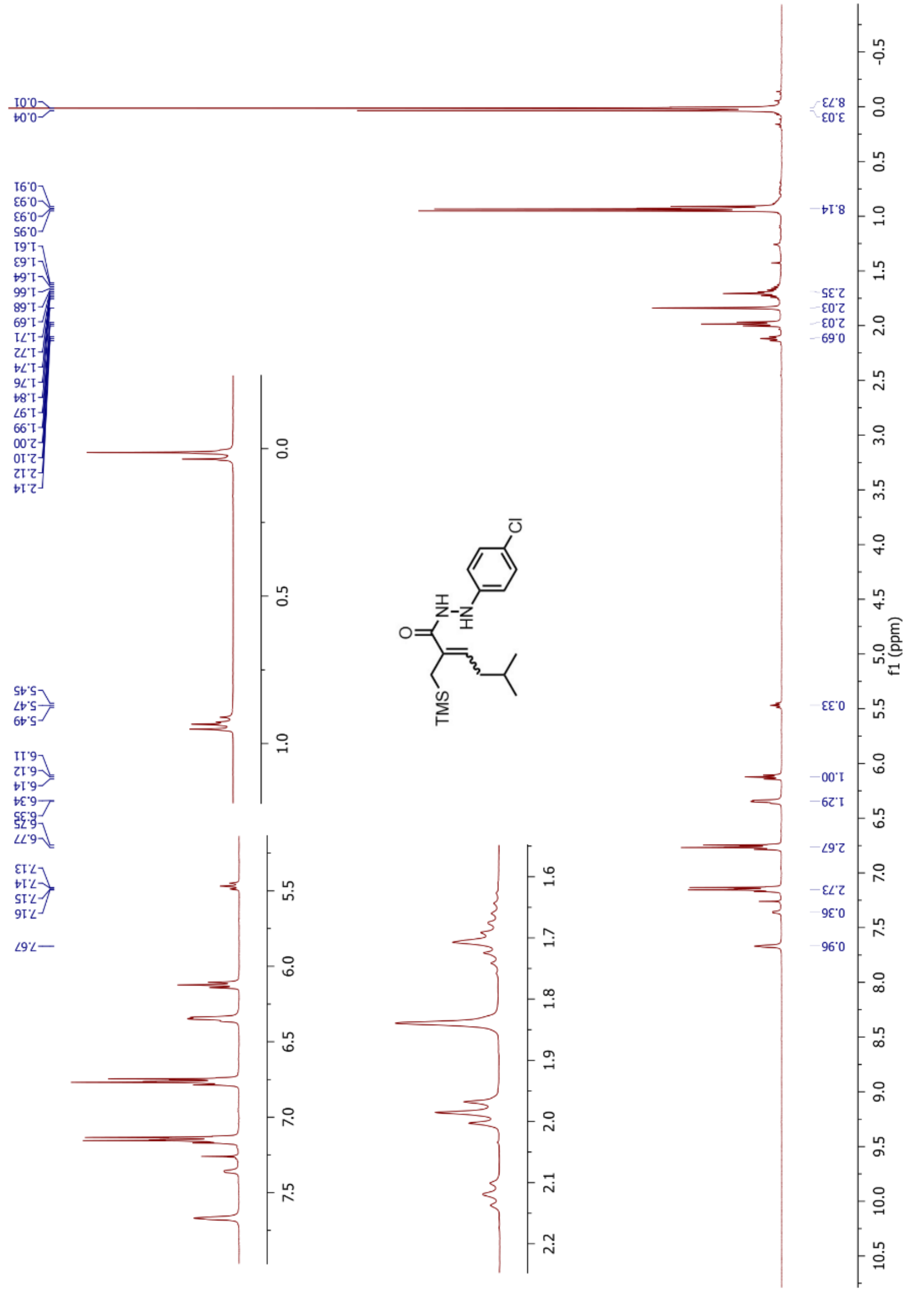

Figure S26. ^{1}H NMR spectrum of **16ac** in $CDCl_3$ (400 MHz).

x

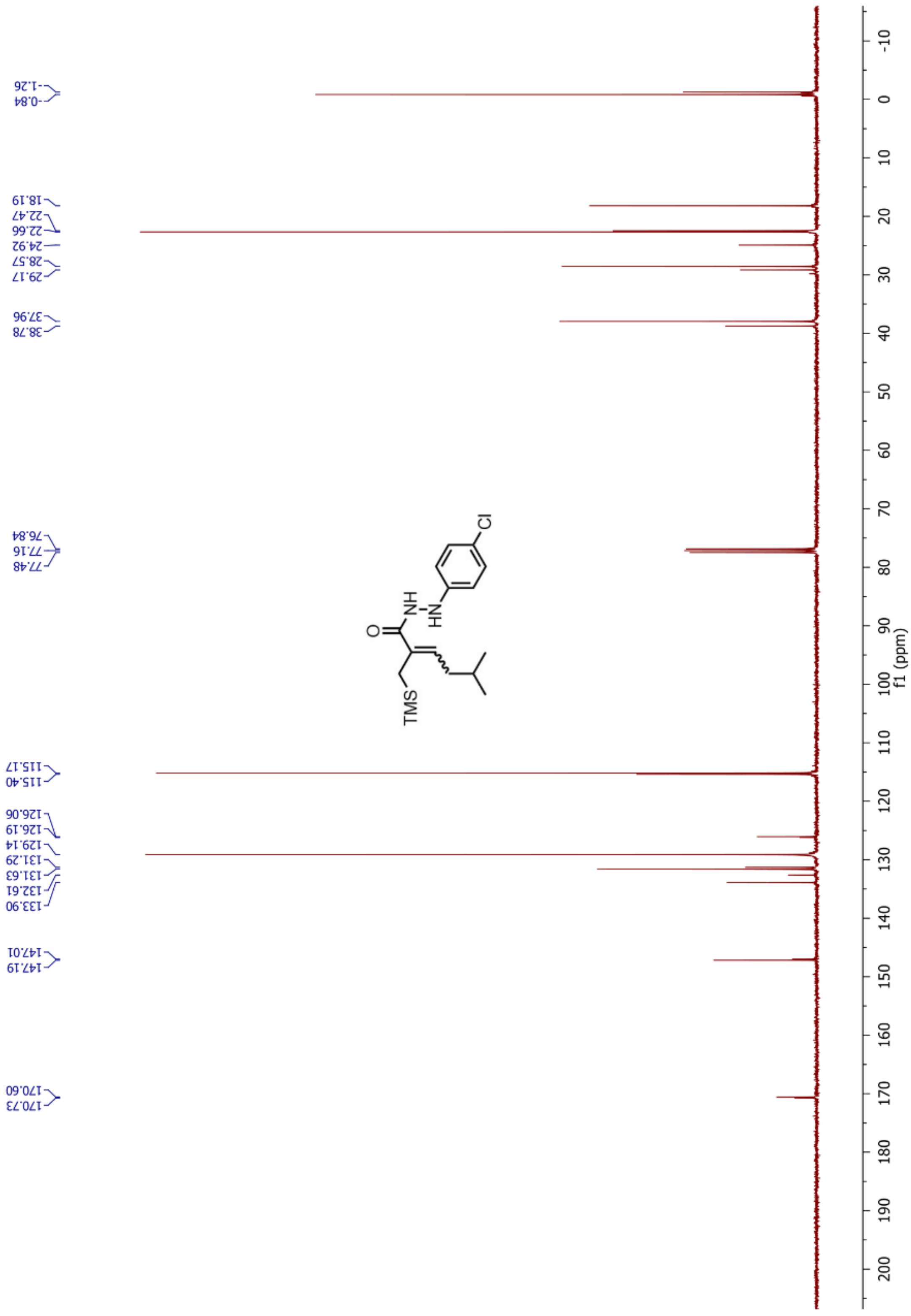

Figure S27. $^{13}C\{^{1}H\}$ NMR spectrum of **16ac** in $CDCl_3$ (100 MHz).

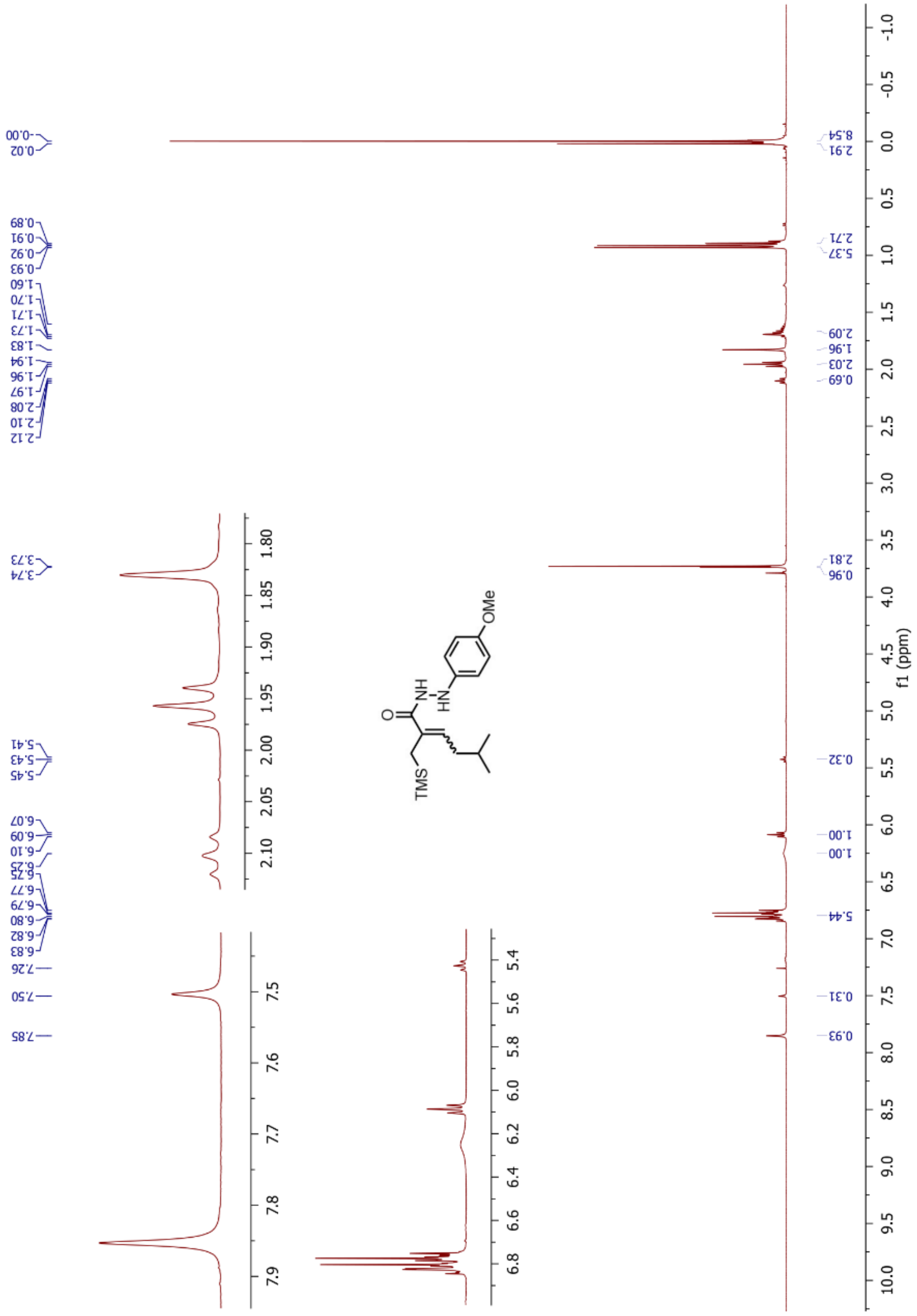

Figure S28. ^{1}H NMR spectrum of **16ad** in $CDCl_3$ (400 MHz).

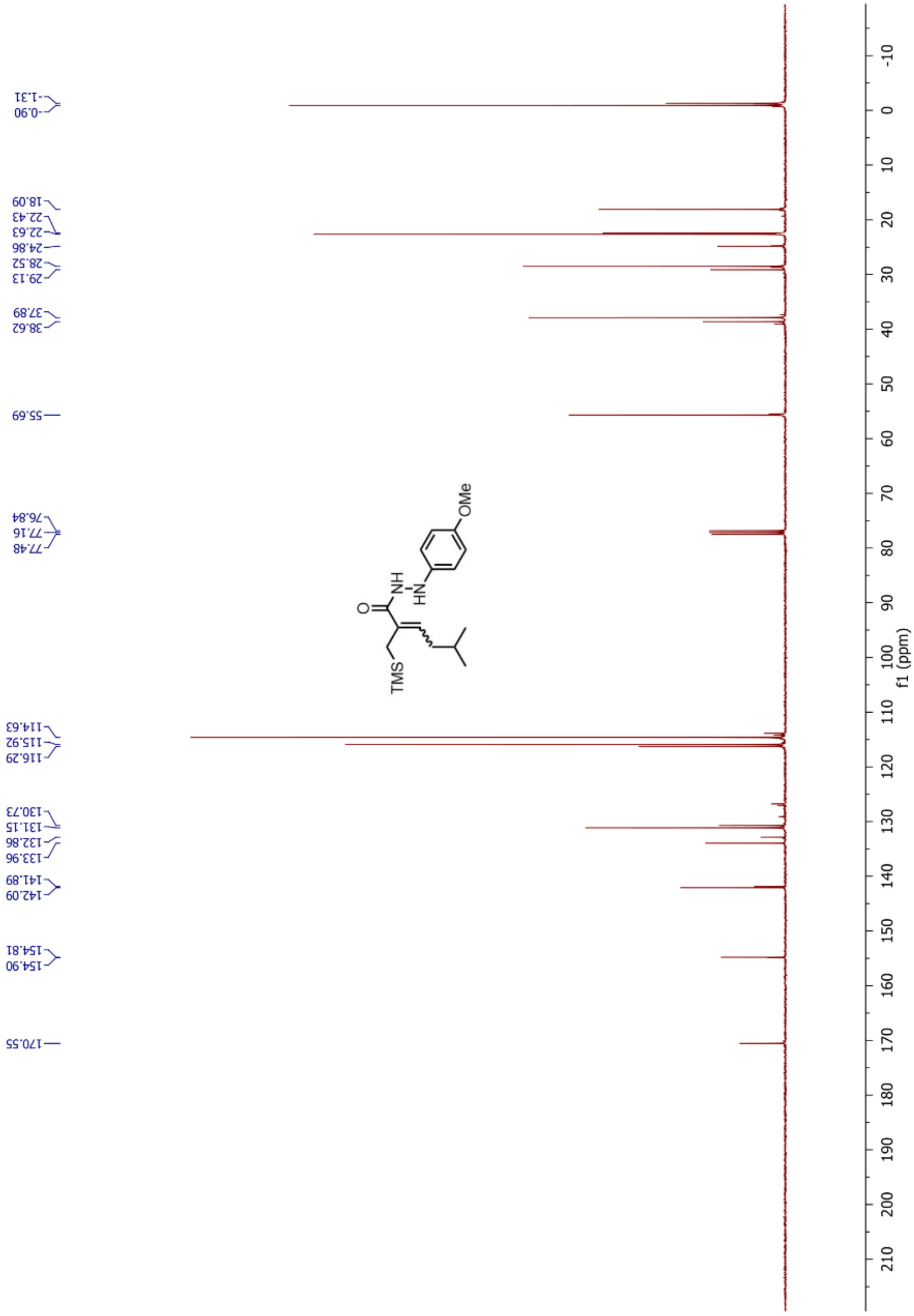

Figure S29. $^{13}C\{^{1}H\}$ NMR spectrum of **16ad** in $CDCl_3$ (100 MHz).

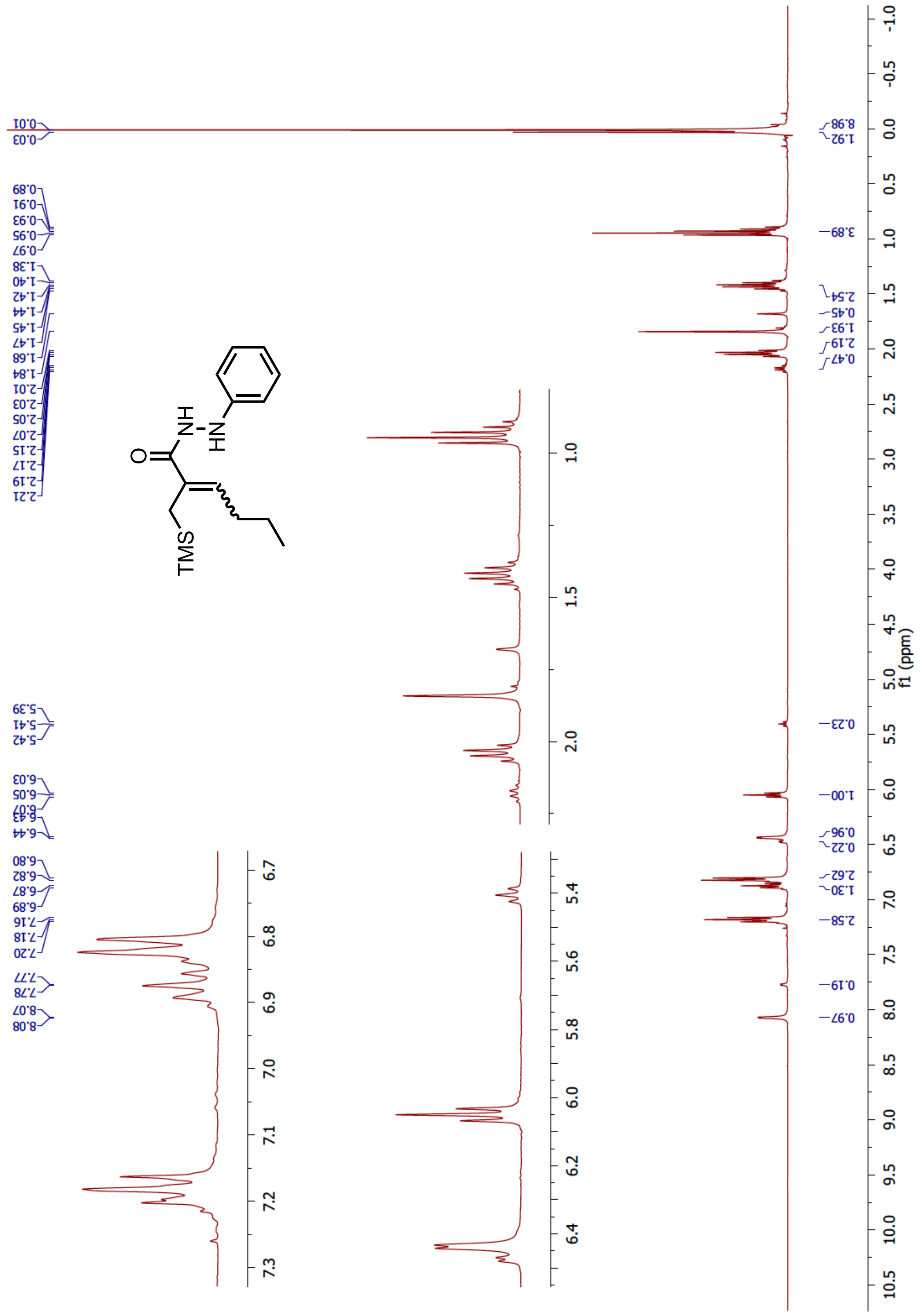

Figure S30. ^{1}H NMR spectrum of **16b** in $CDCl_3$ (400 MHz).

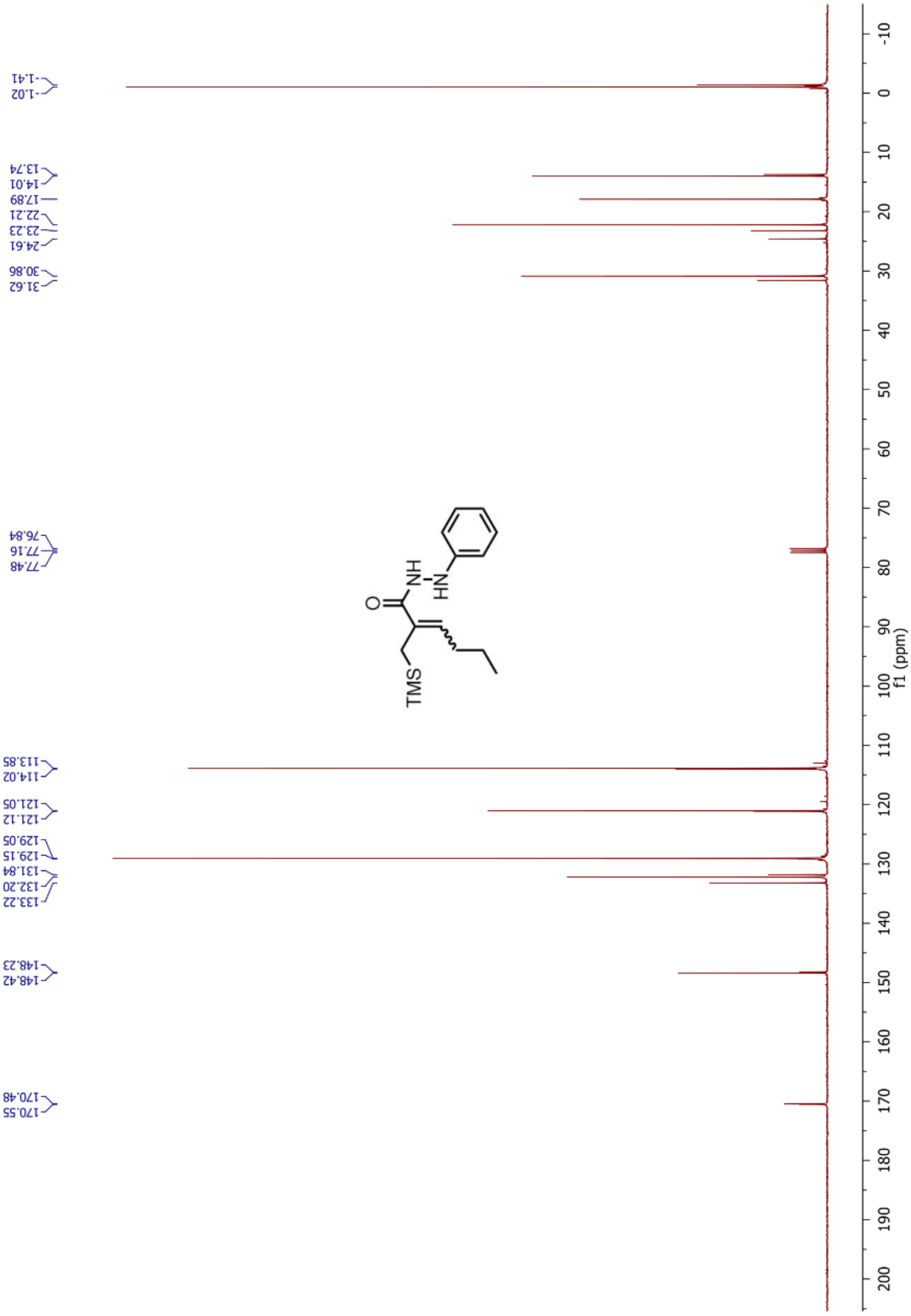

Figure S31. $^{13}C\{^1H\}$ NMR spectrum of **16b** in $CDCl_3$ (100 MHz).

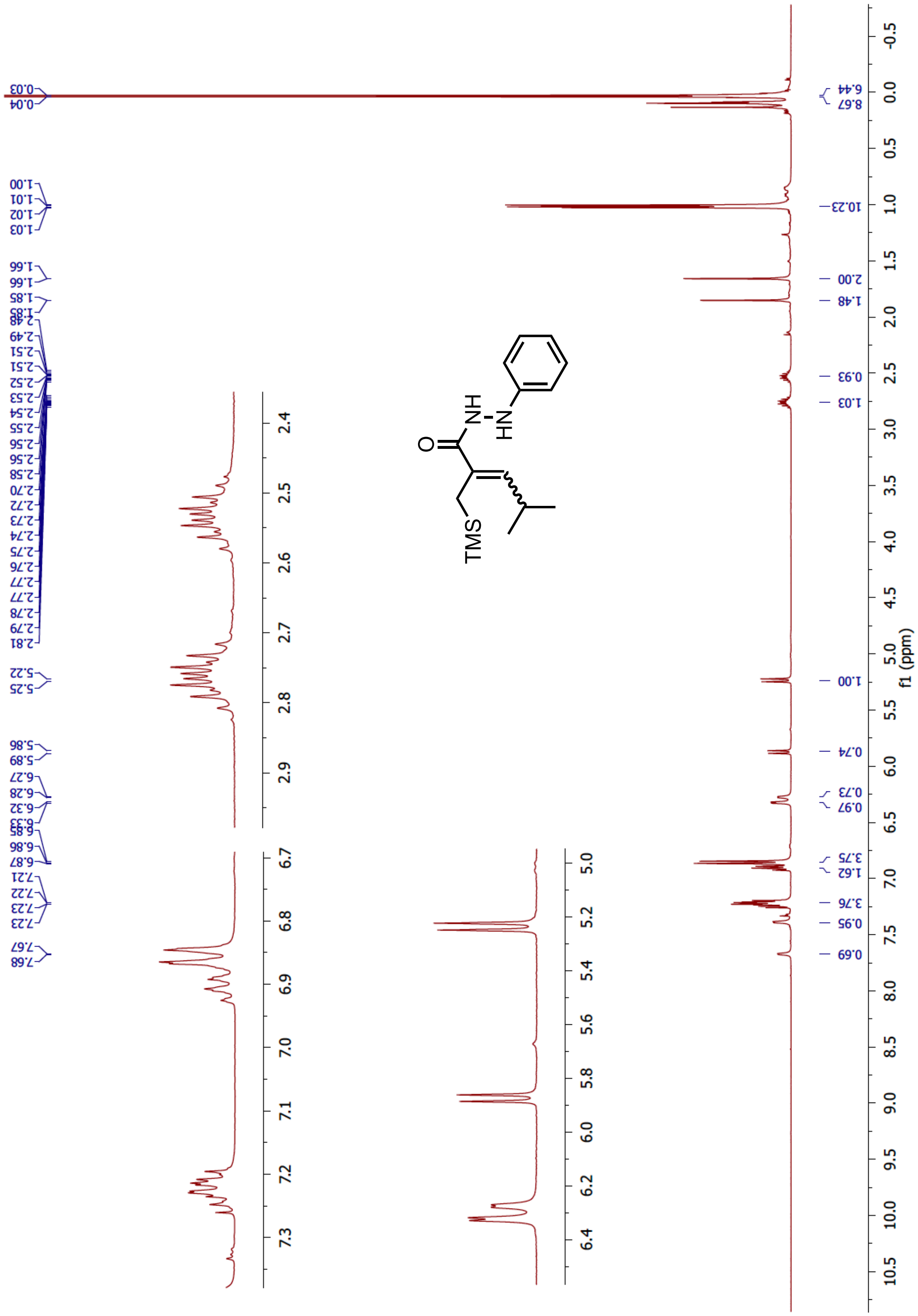

Figure S32. ^{1}H NMR spectrum of **16c** in $CDCl_3$ (400 MHz).

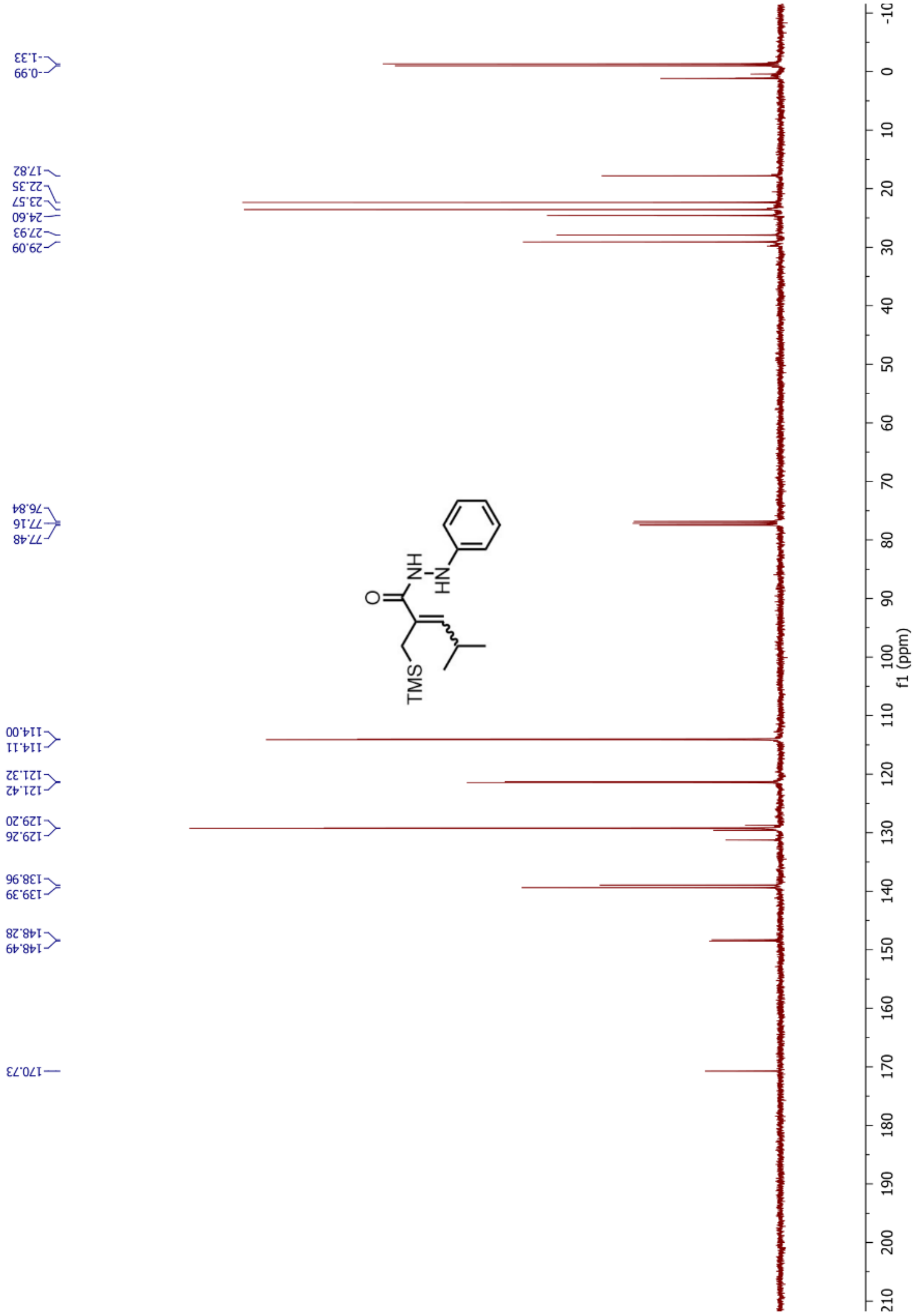

Figure S33. $^{13}C\{^{1}H\}$ NMR spectrum of **16c** in $CDCl_3$ (100 MHz).

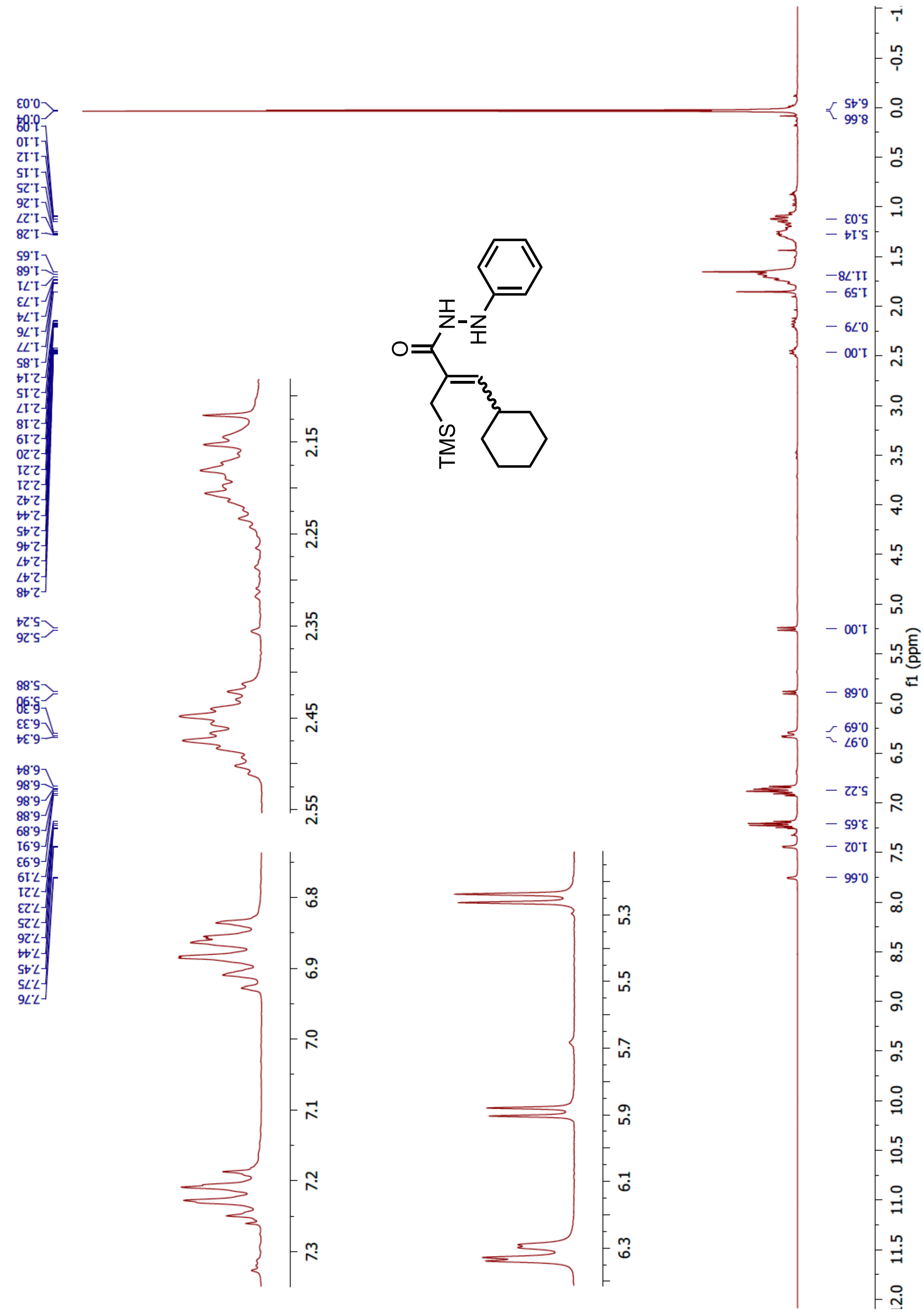

Figure S34. ^{1}H NMR spectrum of **16d** in $CDCl_3$ (400 MHz).

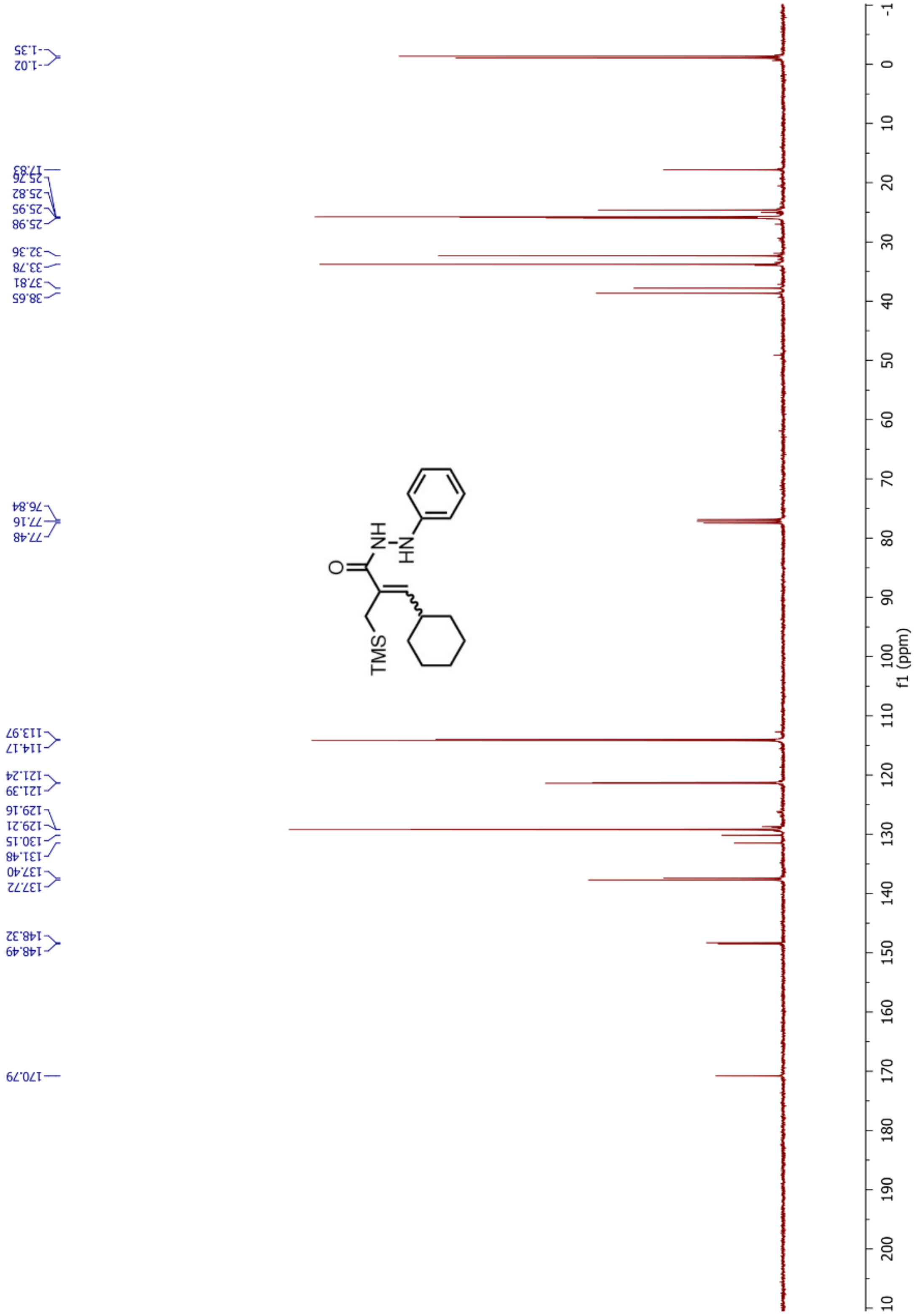

Figure S35. $^{13}C\{^1H\}$ NMR spectrum of **16d** in $CDCl_3$ (100 MHz).

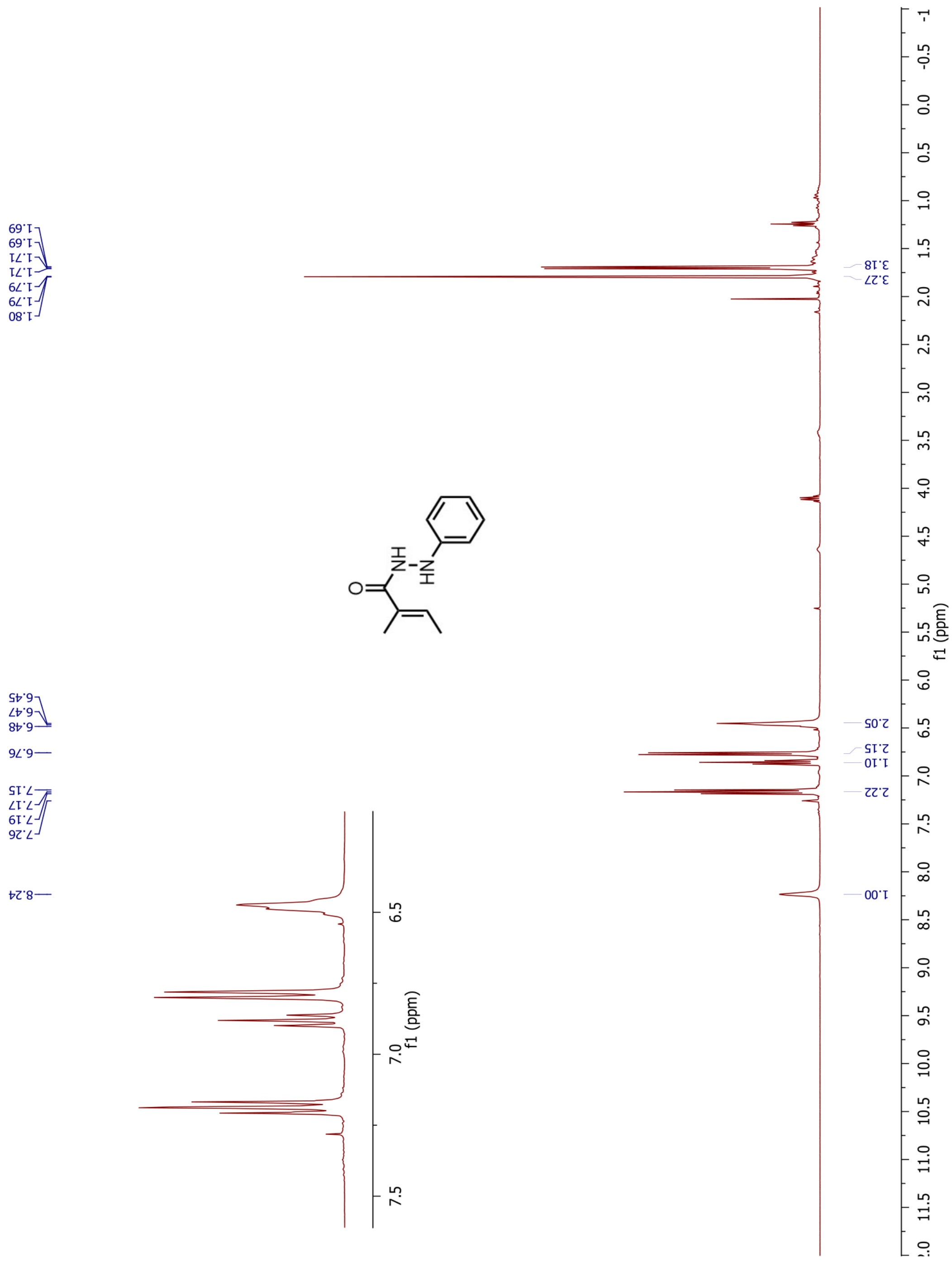

Figure S36. ^{1}H NMR spectrum of **18** in $CDCl_3$ (400 MHz).

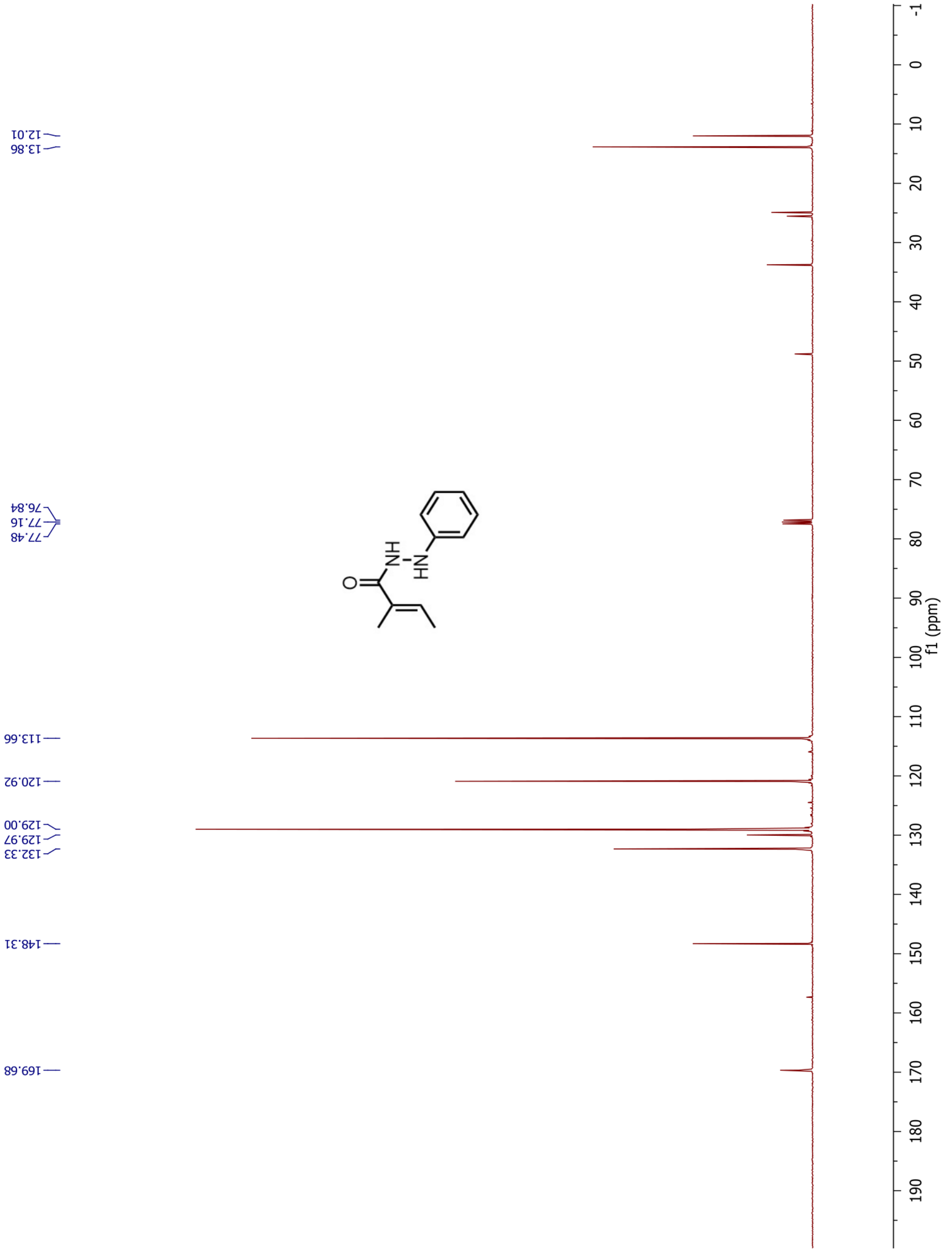

Figure S37. $^{13}C\{^1H\}$ NMR spectrum of **18** in $CDCl_3$ (100 MHz).

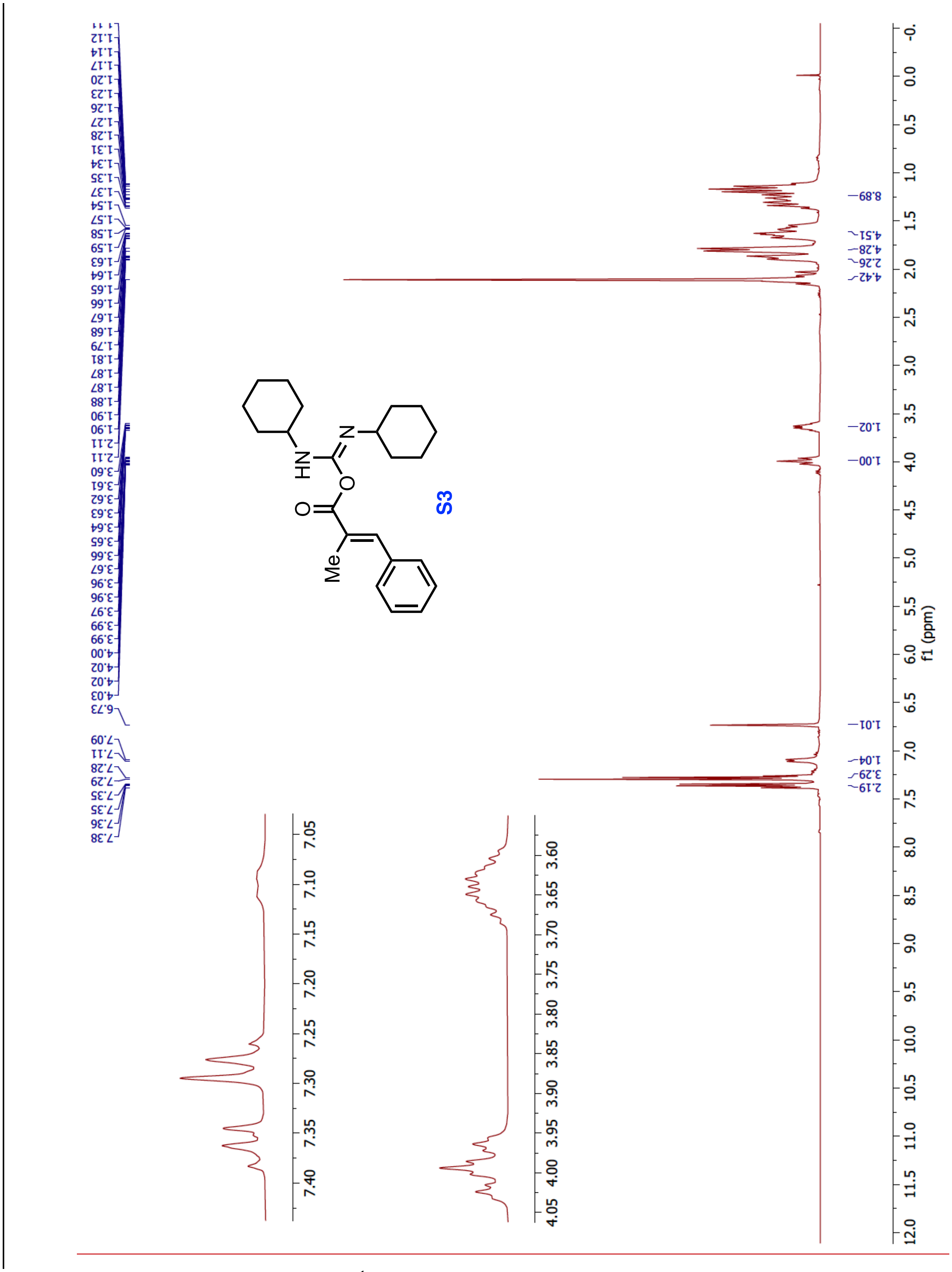

Figure S38. ^{1}H NMR spectrum of **S3** in $CDCl_3$ (400 MHz).

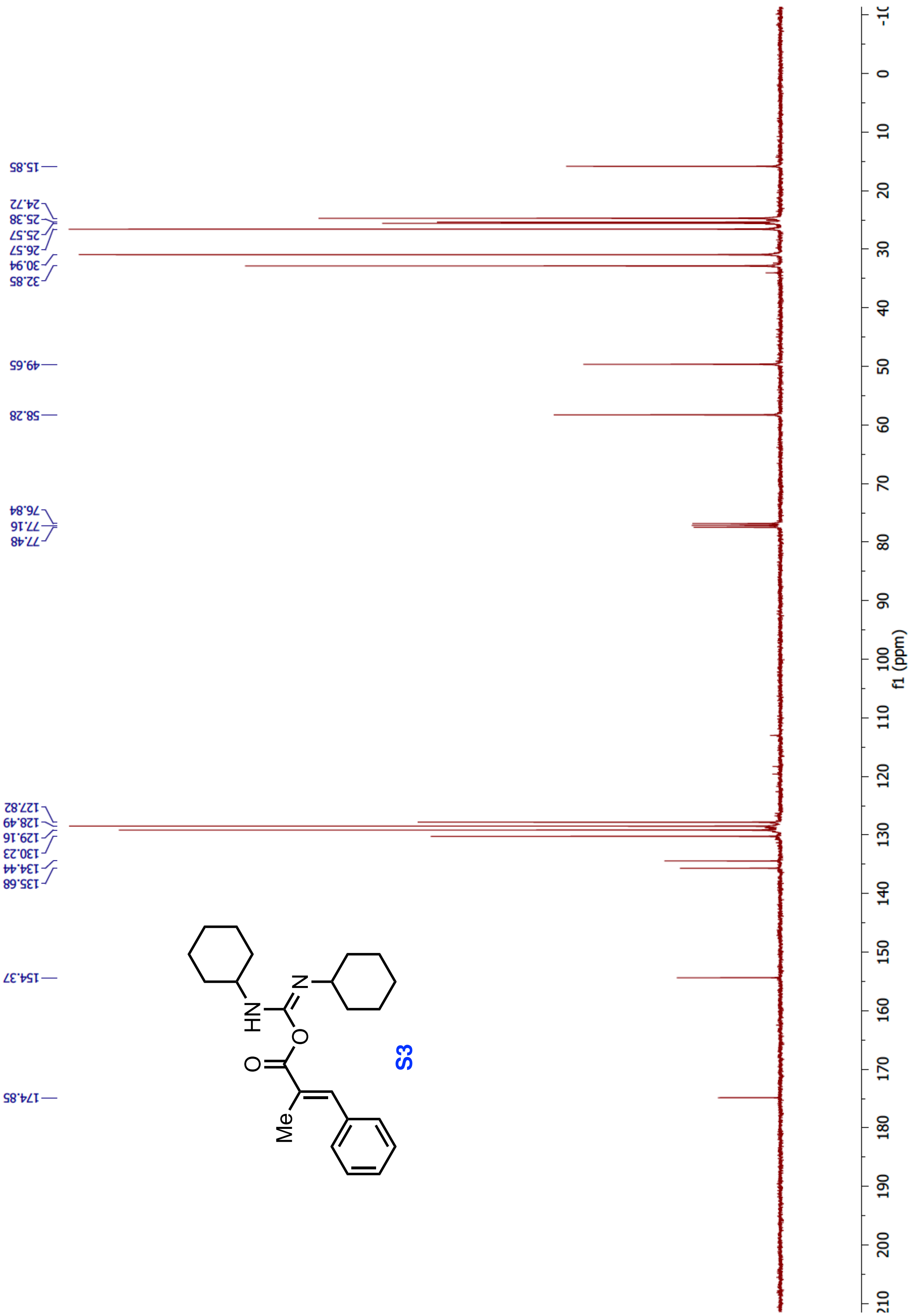

Figure S39. $^{13}C\{^{1}H\}$ NMR spectrum of **S3** in $CDCl_3$ (100 MHz).

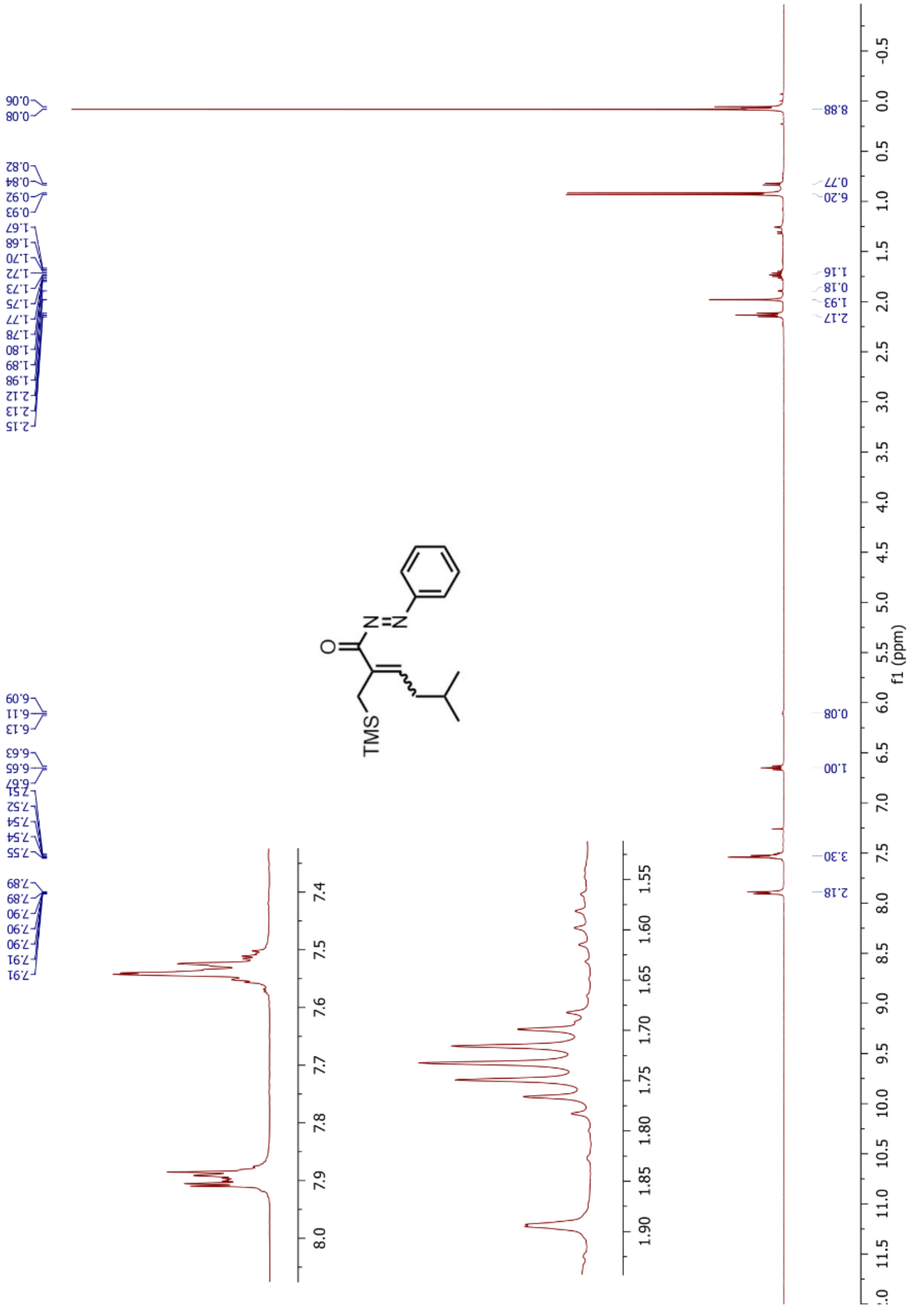

Figure S40. ^{1}H NMR spectrum of **10aa** in $CDCl_3$ (400 MHz).

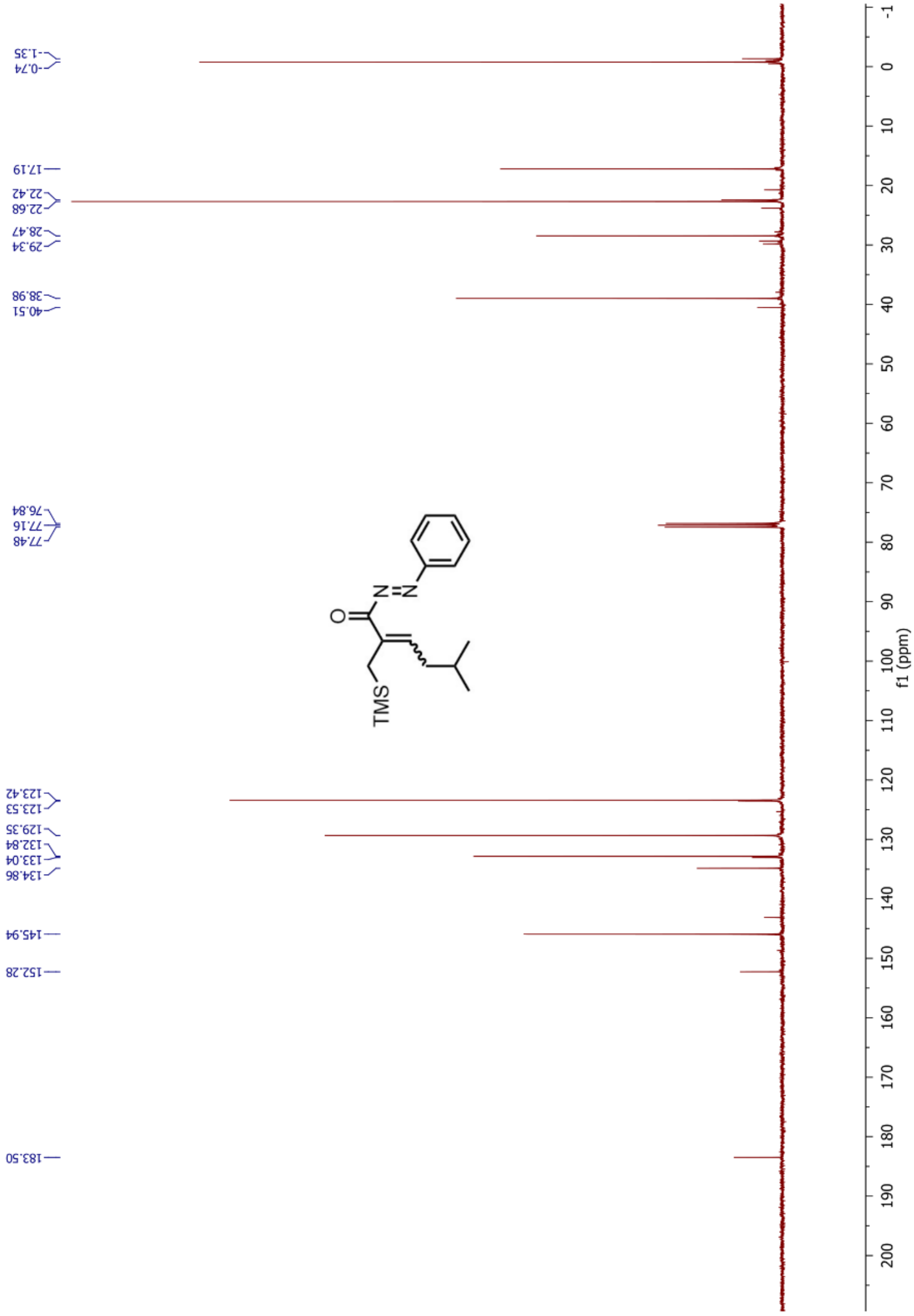

Figure S41. $^{13}C\{^{1}H\}$ NMR spectrum of **10aa** in $CDCl_3$ (100 MHz).

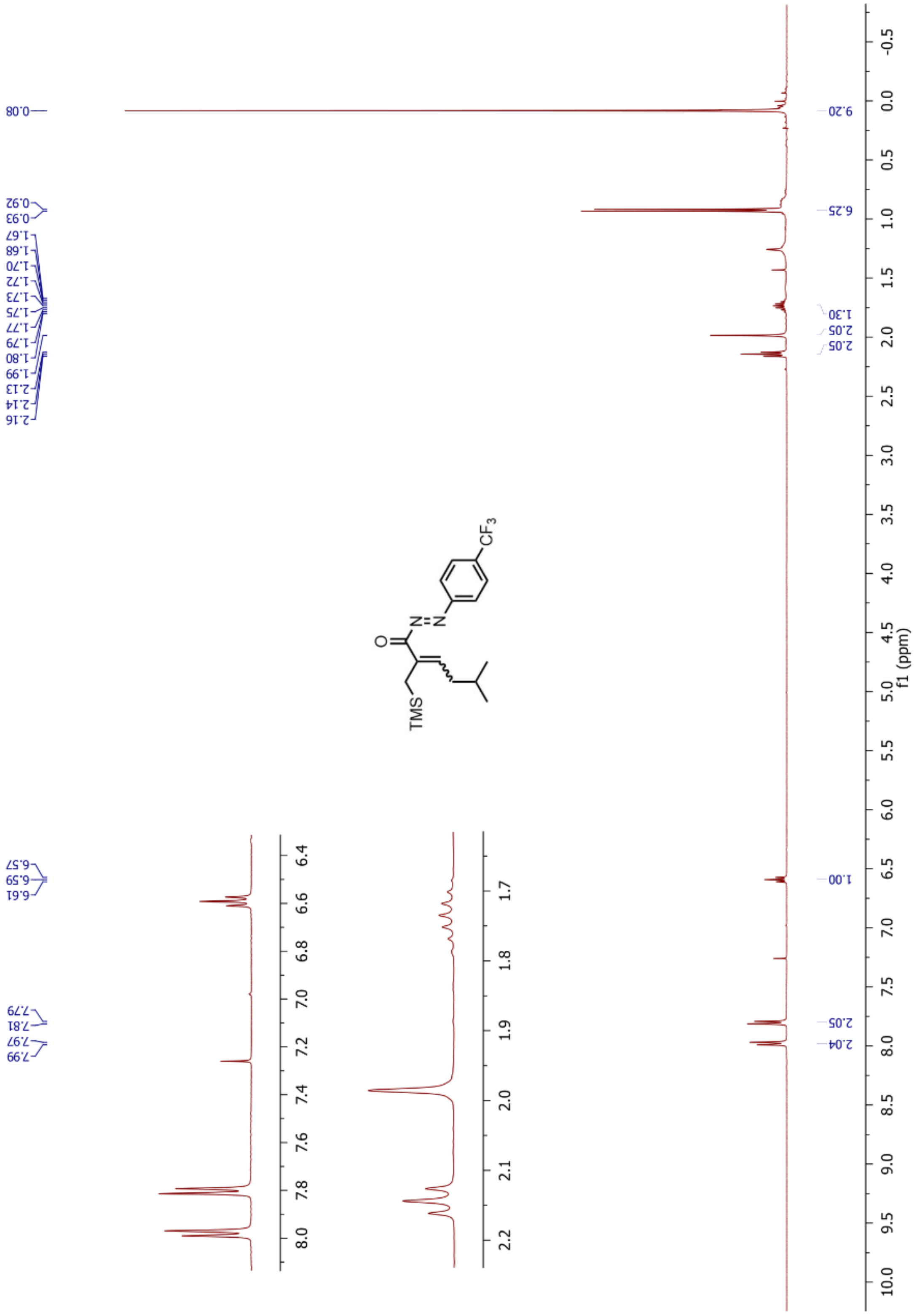

Figure S42. ^{1}H NMR spectrum of **10ab** in $CDCl_3$ (400 MHz).

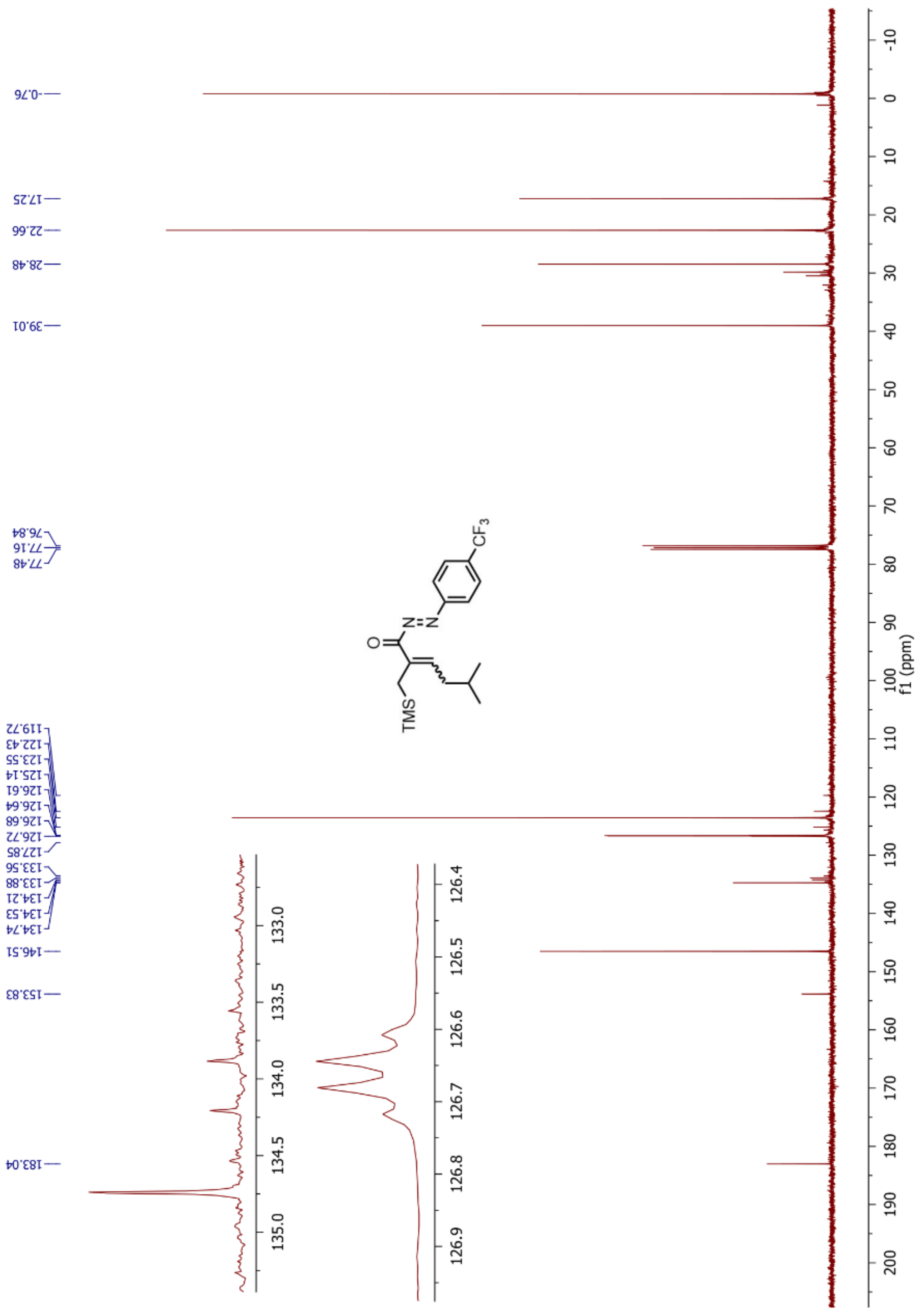

Figure S43. $^{13}C\{^{1}H\}$ NMR spectrum of **10ab** in $CDCl_3$ (100 MHz).

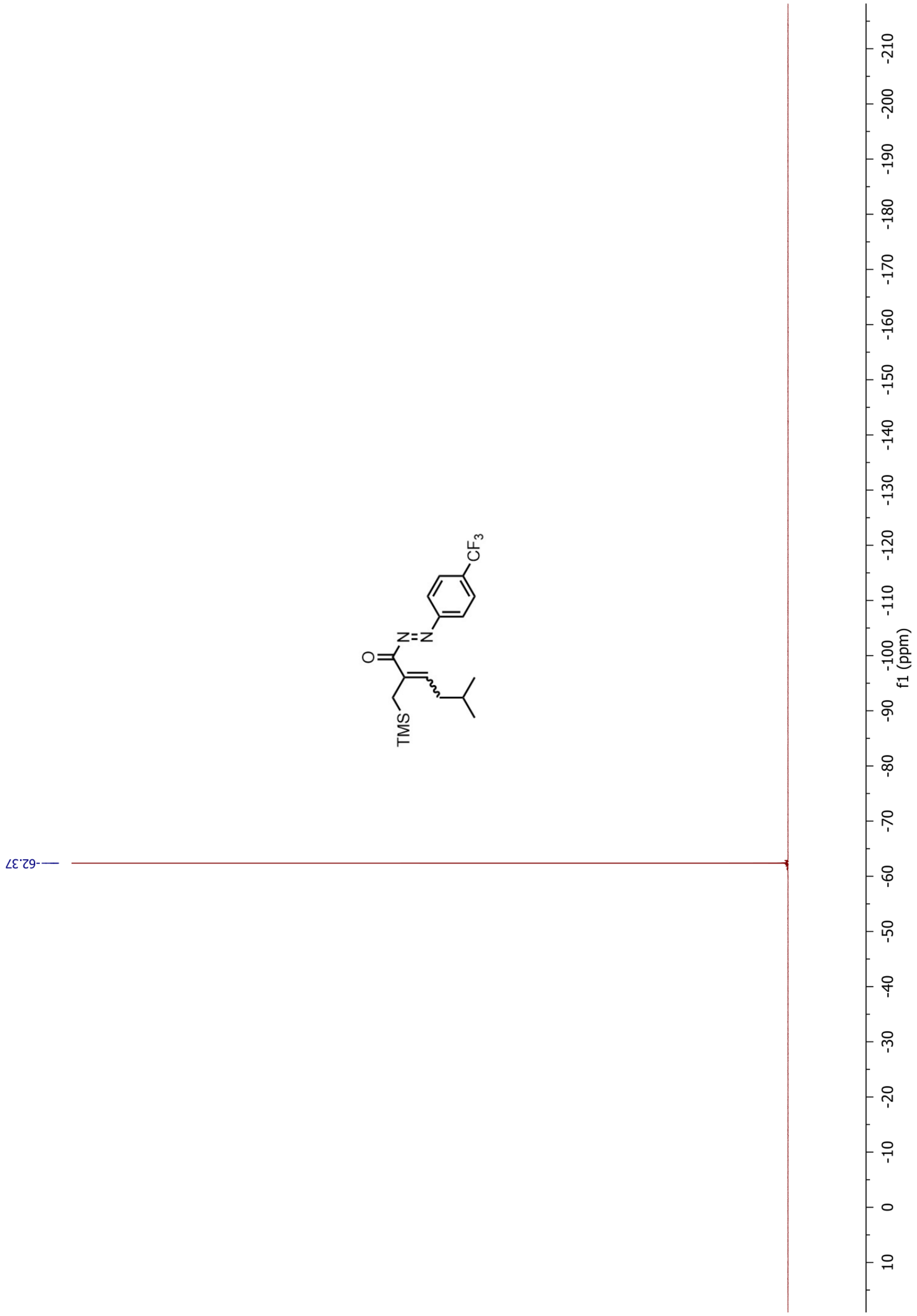

Figure S44. ^{19}F NMR spectrum of **10ab** in $CDCl_3$ (376 MHz).

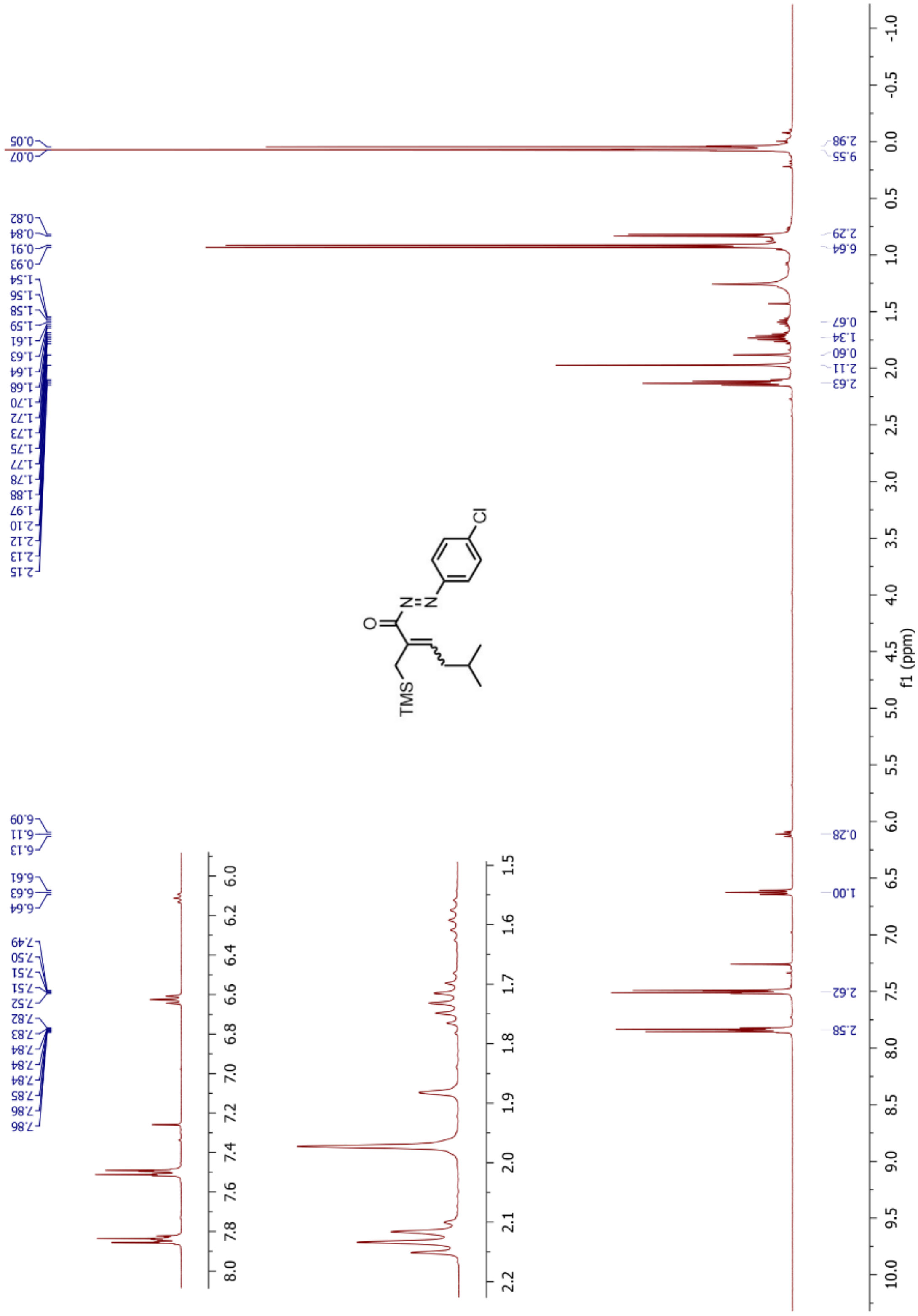

Figure S45. ^{1}H NMR spectrum of **10ac** in $CDCl_3$ (400 MHz).

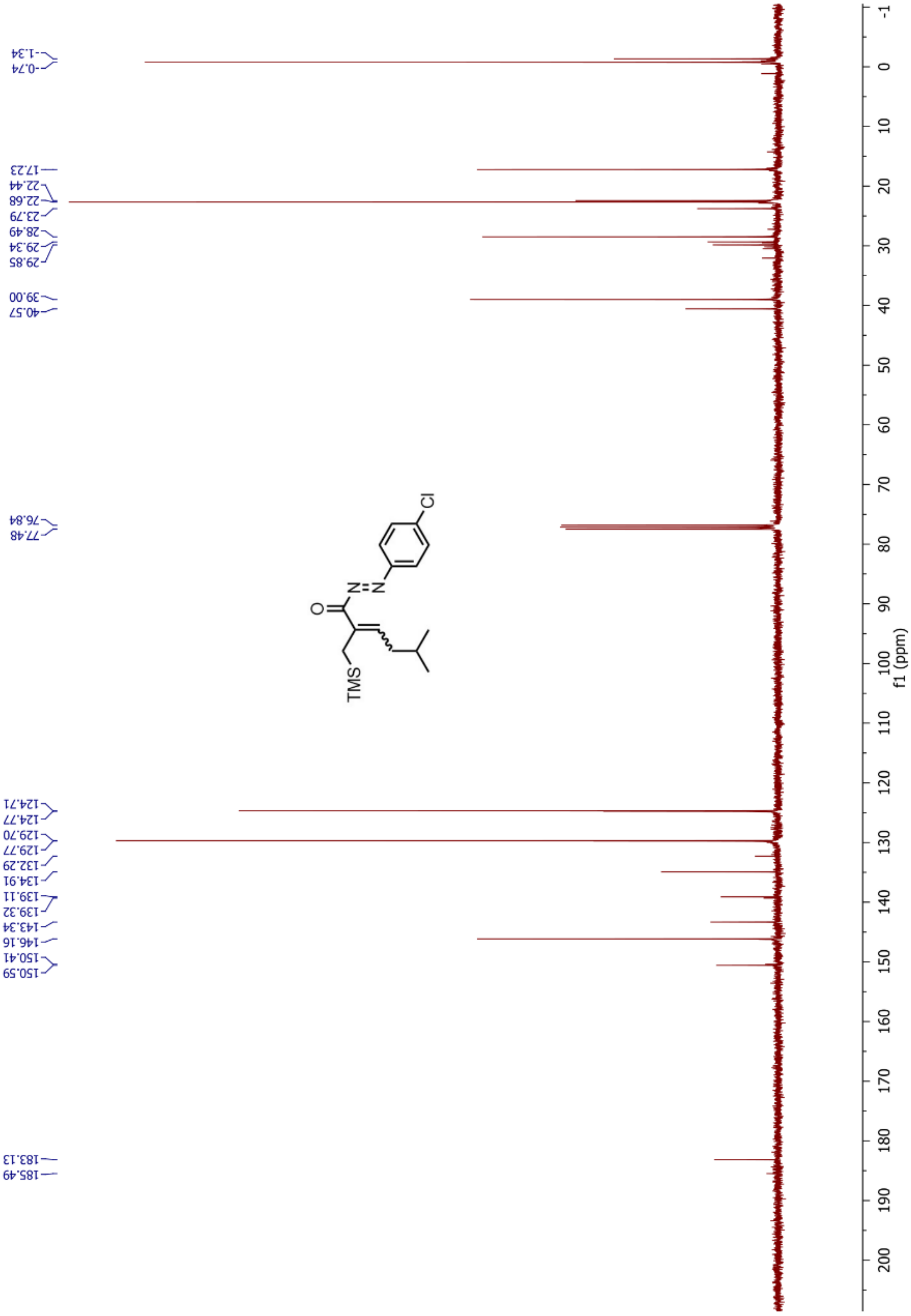

Figure S46. $^{13}C\{^{1}H\}$ NMR spectrum of **10ac** in $CDCl_3$ (100 MHz).

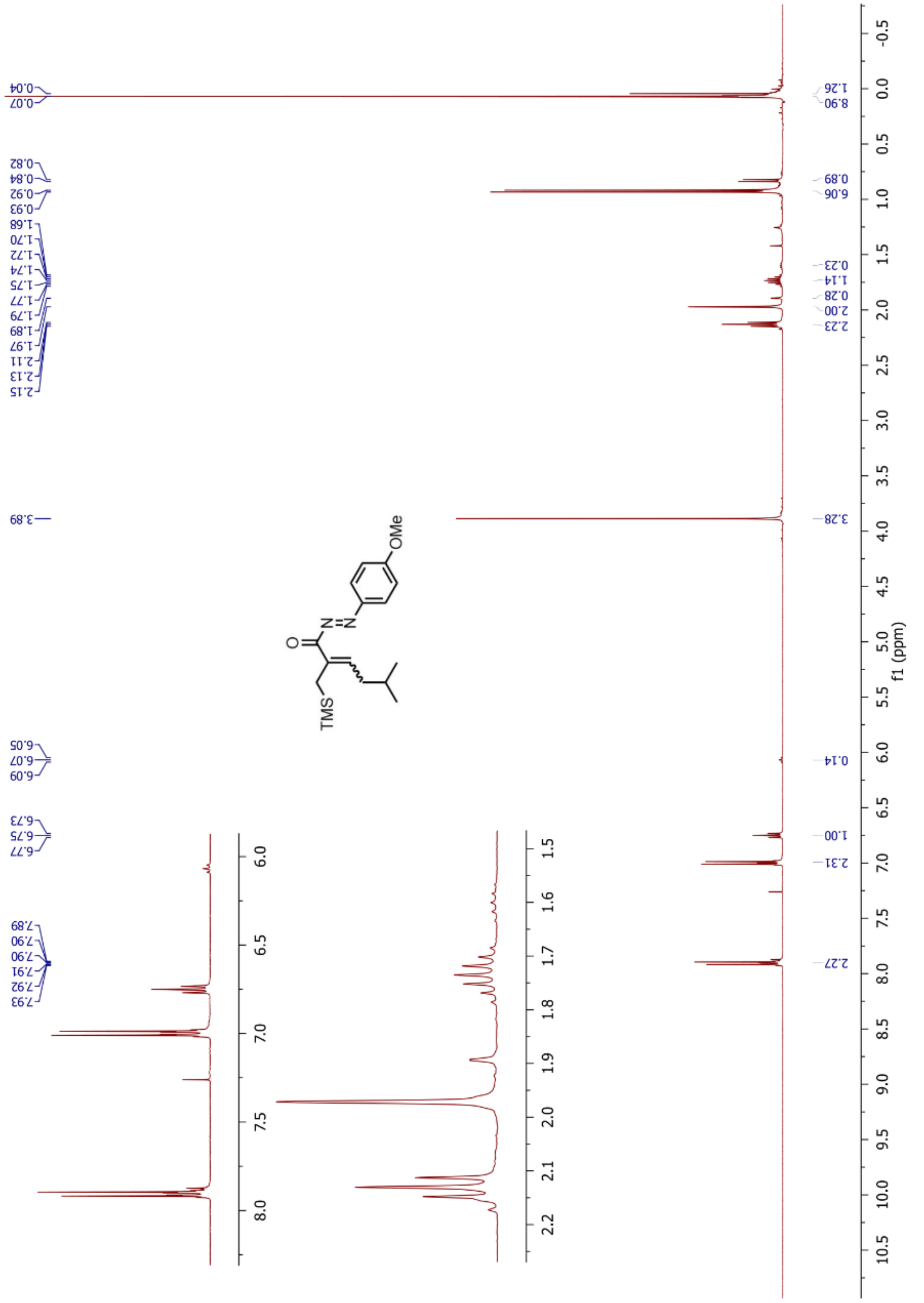

Figure S47. ^{1}H NMR spectrum of **10ad** in $CDCl_3$ (400 MHz).

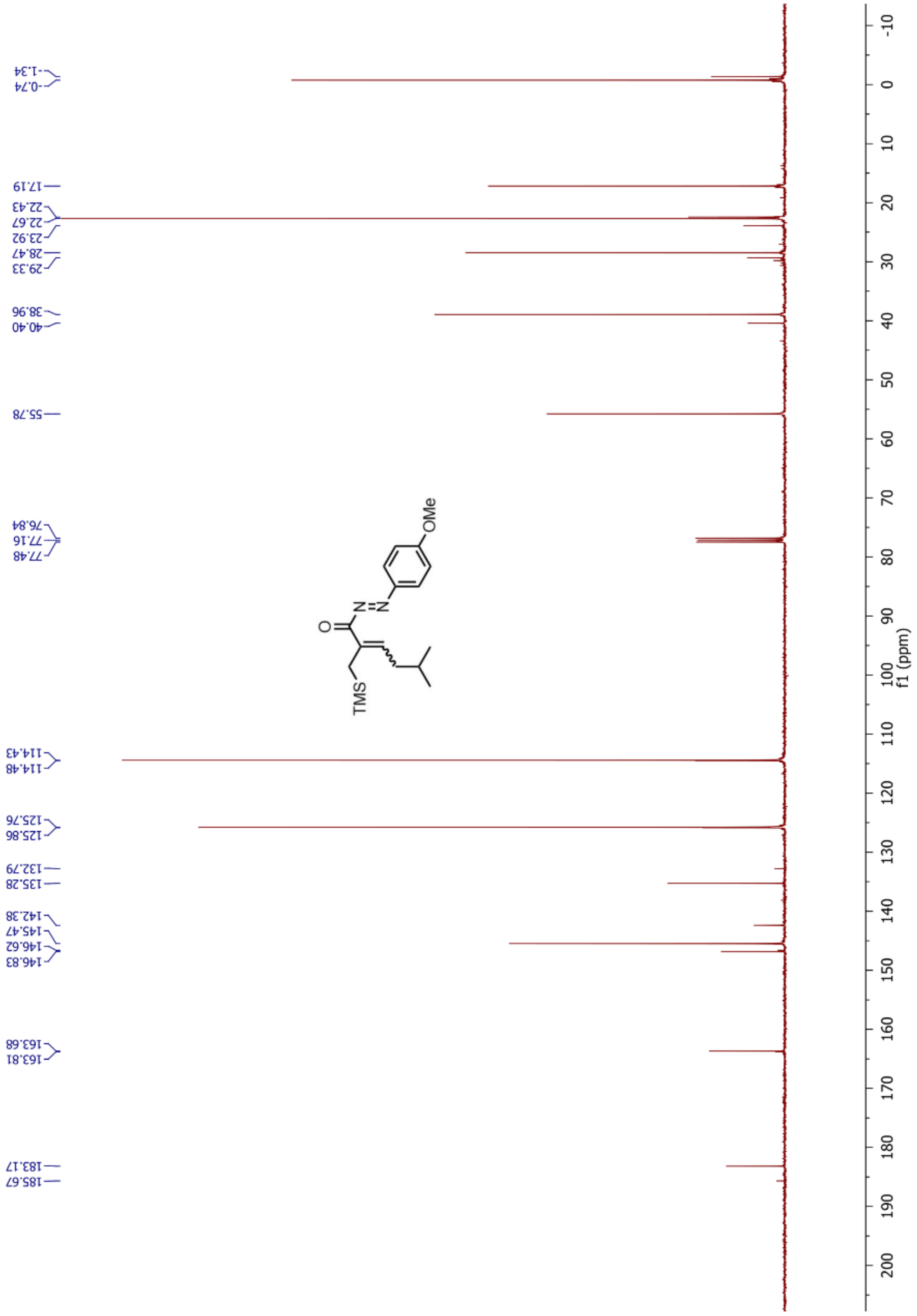

Figure S48. $^{13}C\{^1H\}$ NMR spectrum of **10ad** in $CDCl_3$ (100 MHz).

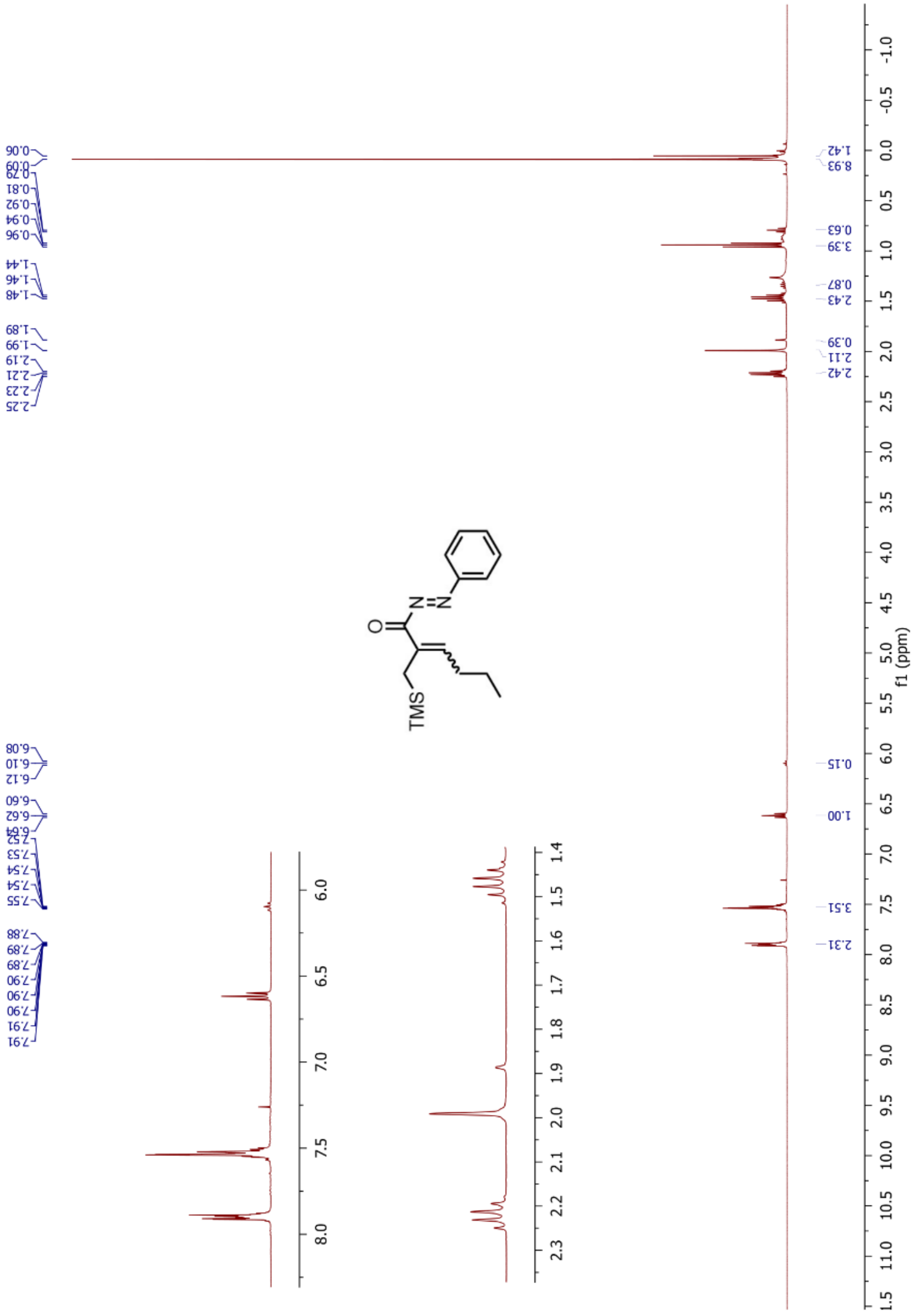

Figure S49. ^{1}H NMR spectrum of **10b** in $CDCl_3$ (400 MHz).

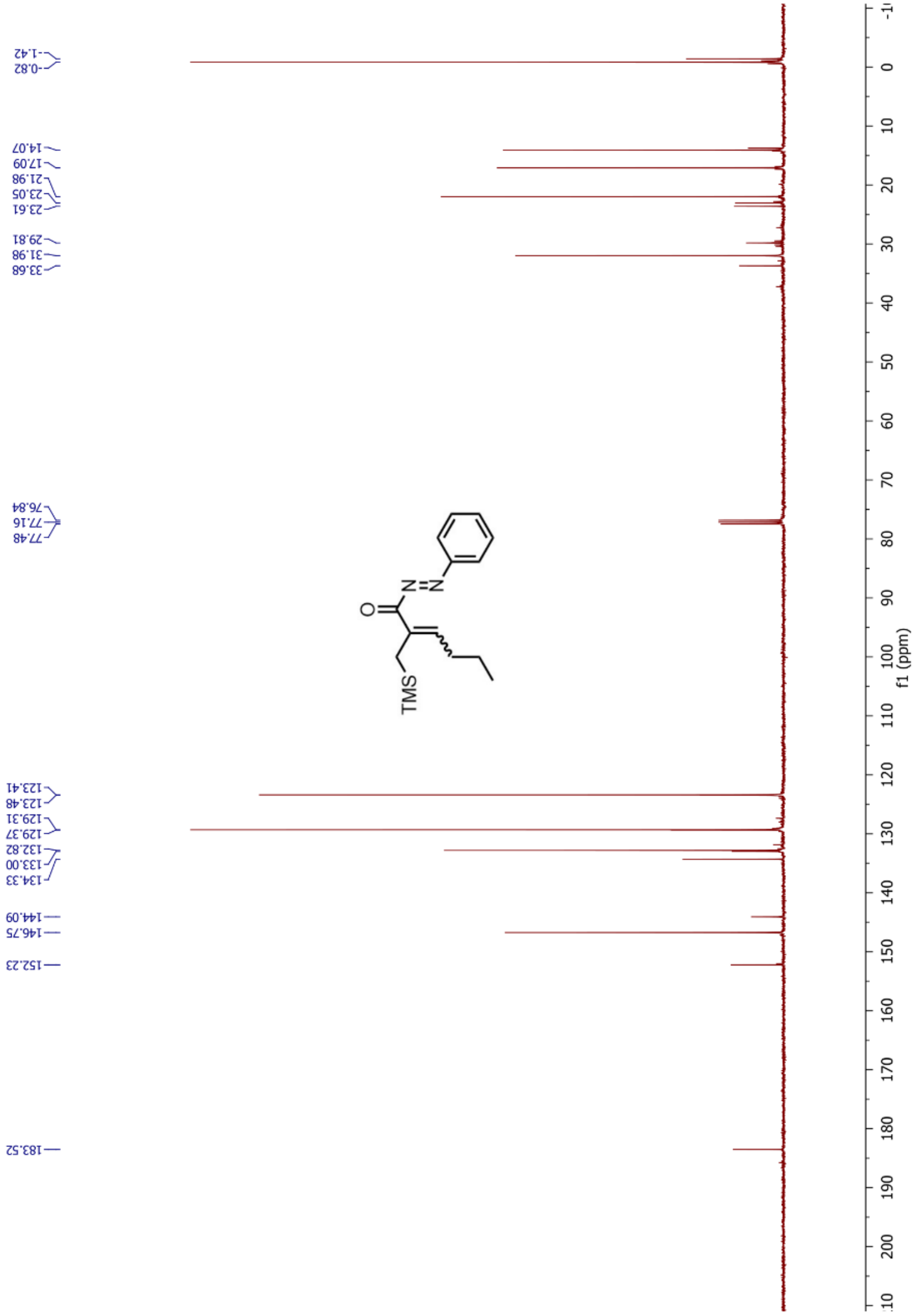

Figure S50. $^{13}C\{^{1}H\}$ NMR spectrum of **10b** in $CDCl_3$ (100 MHz).

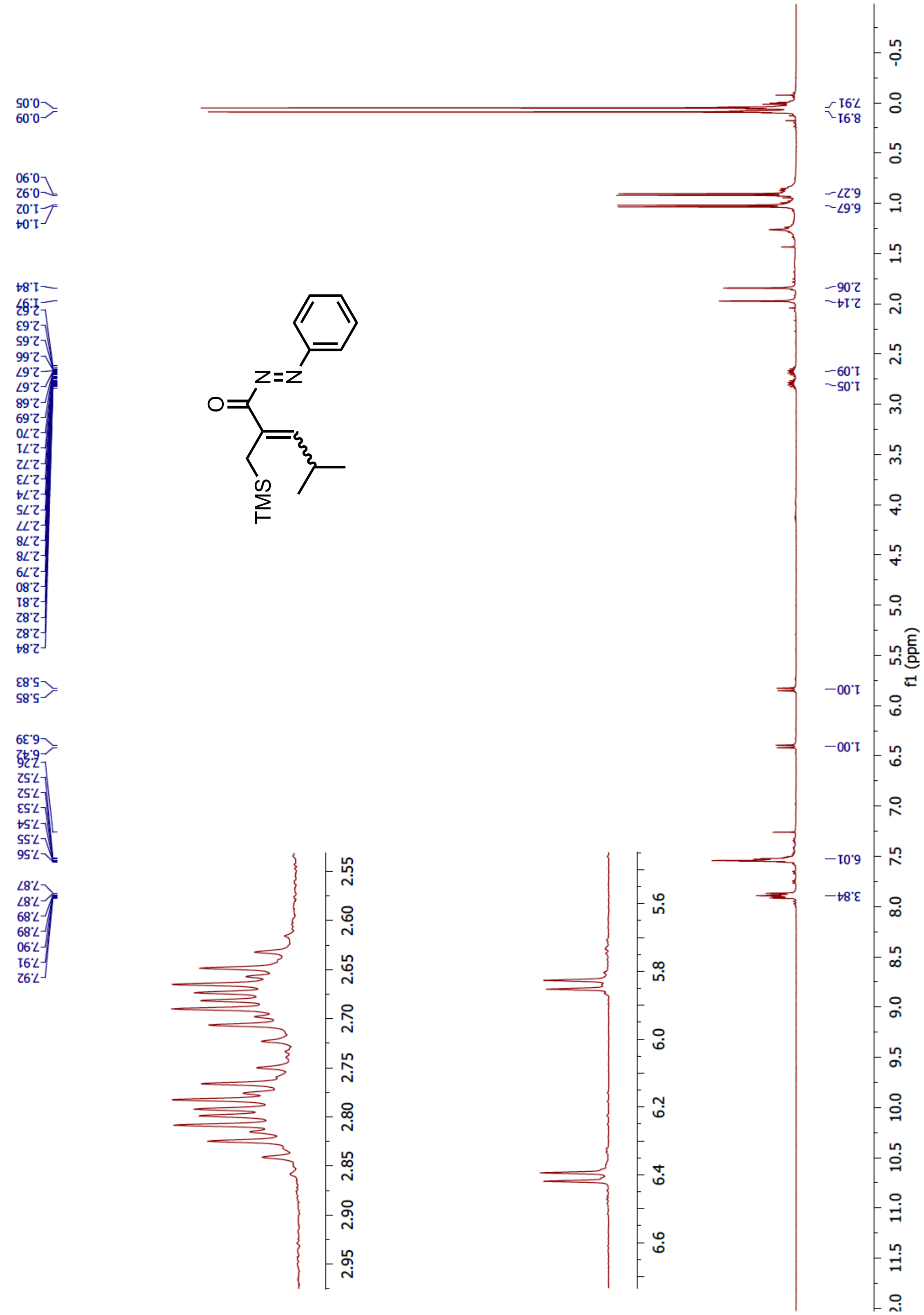

Figure S51. ^{1}H NMR spectrum of **10c** in $CDCl_3$ (400 MHz).

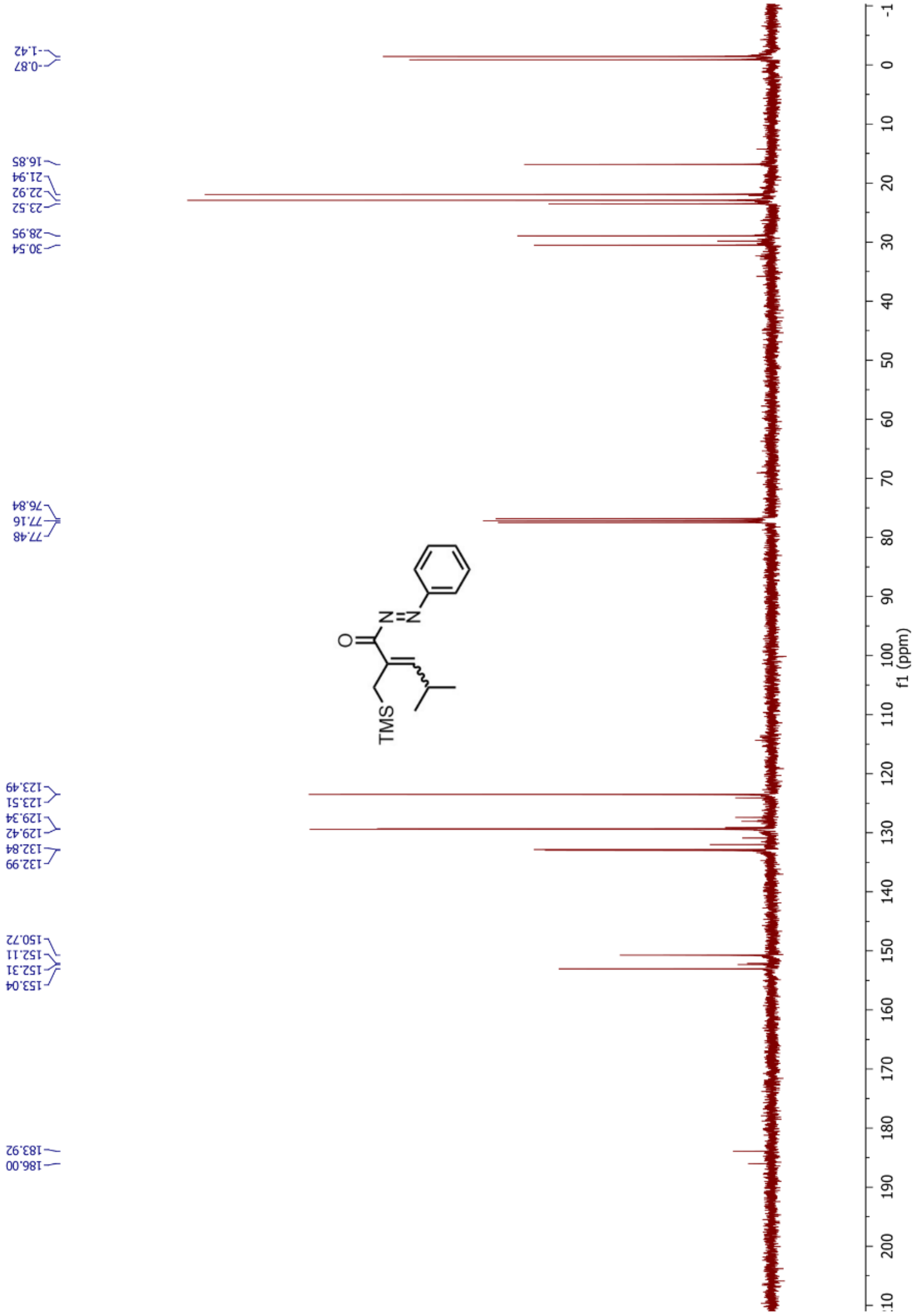

Figure S52. $^{13}C\{^{1}H\}$ NMR spectrum of **10c** in $CDCl_3$ (100 MHz).

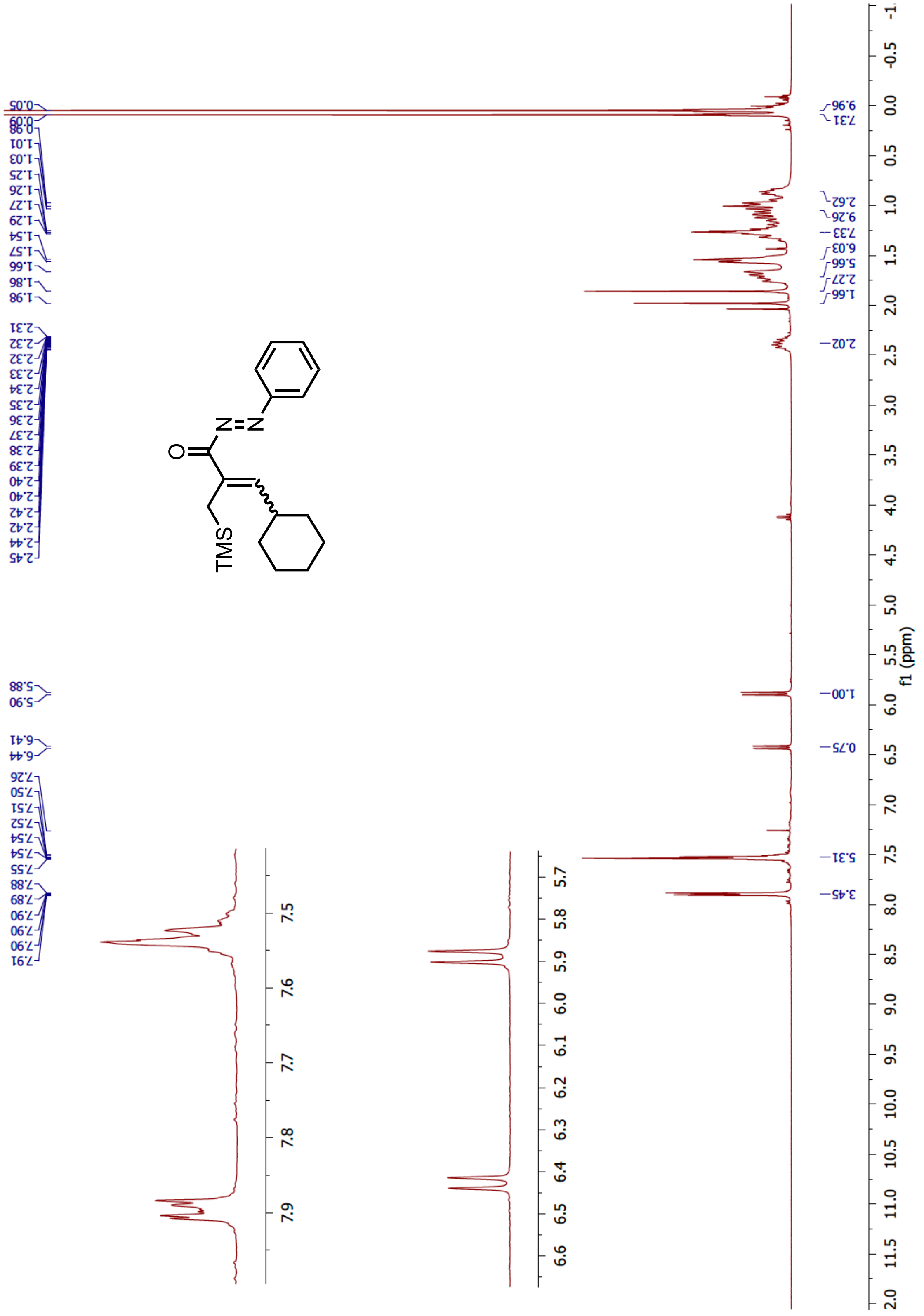

Figure S53. ^{1}H NMR spectrum of **10d** in $CDCl_3$ (400 MHz).

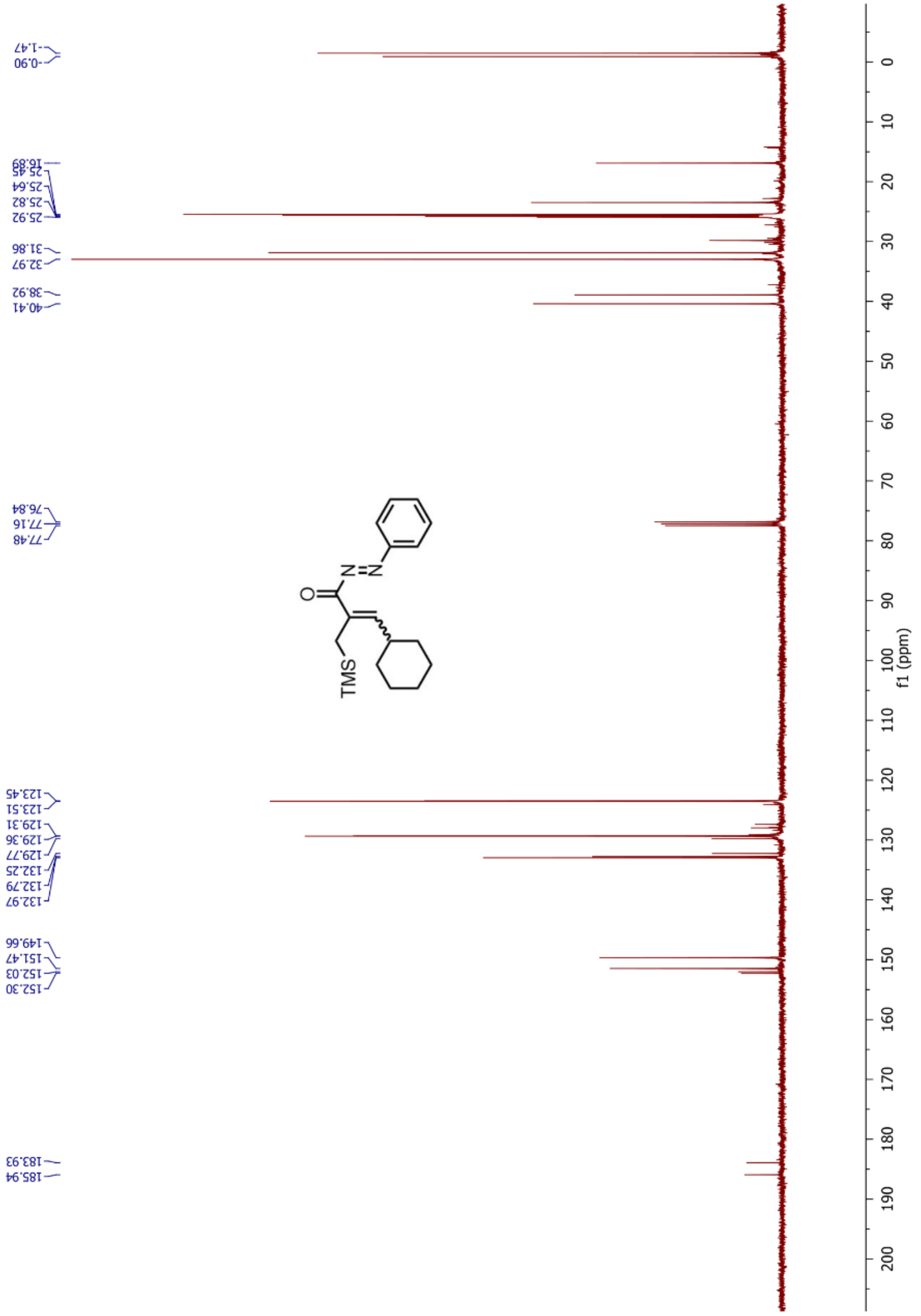

Figure S54. $^{13}C\{^{1}H\}$ NMR spectrum of **10d** in $CDCl_3$ (100 MHz).

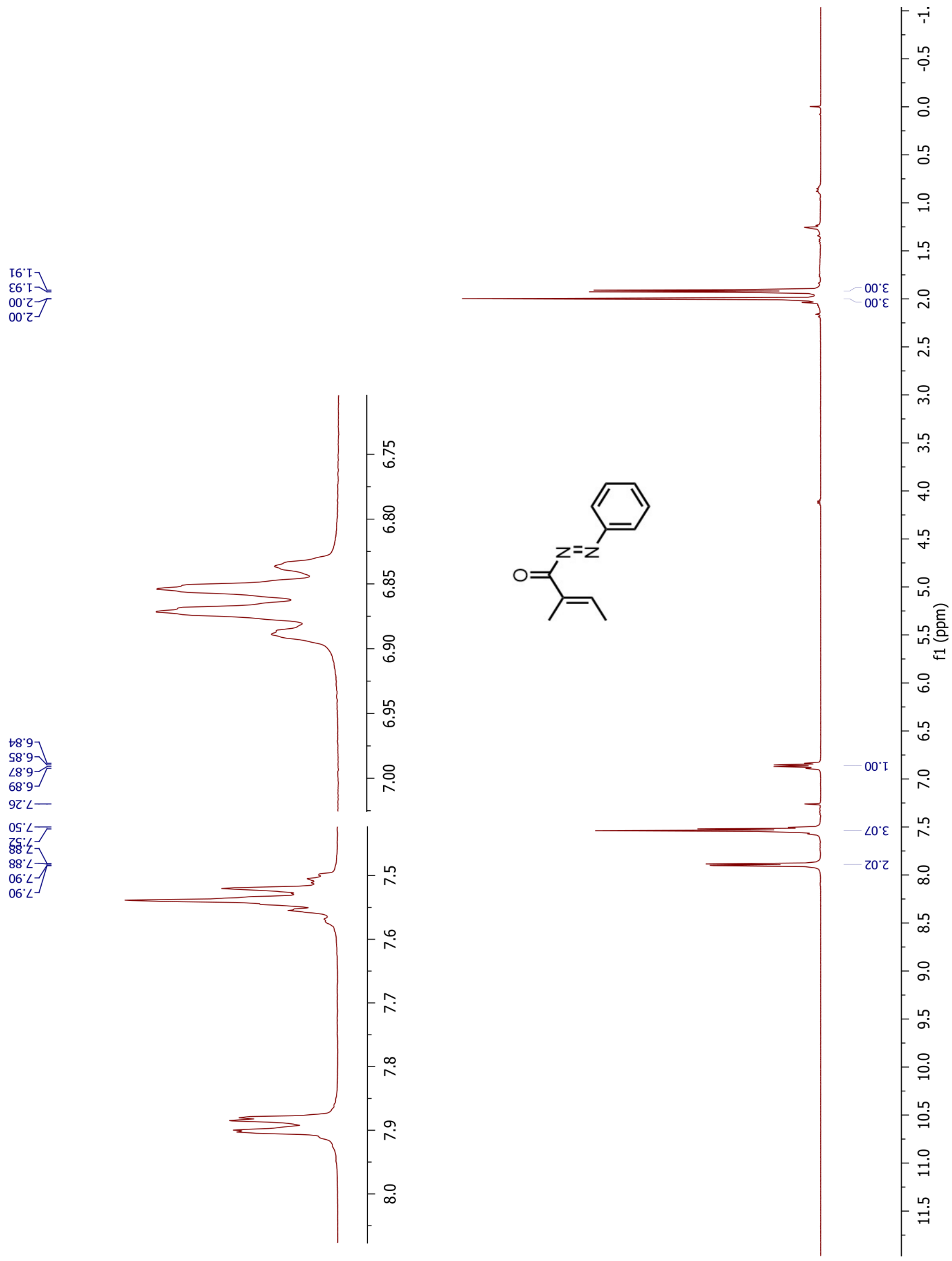

Figure S55. ^{1}H NMR spectrum of **19** in $CDCl_3$ (400 MHz).

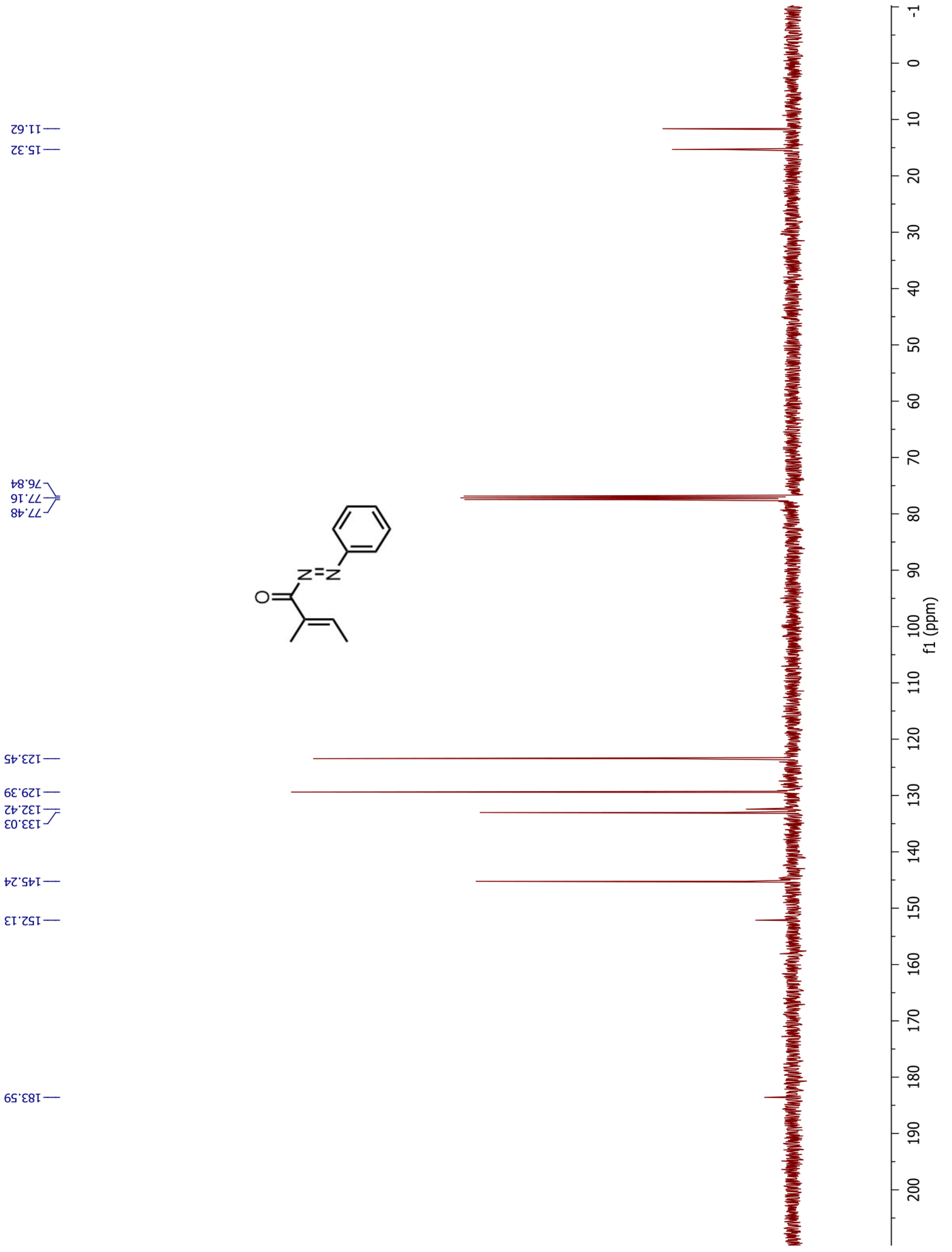

Figure S56. $^{13}C\{^{1}H\}$ NMR spectrum of **19** in $CDCl_3$ (100 MHz).

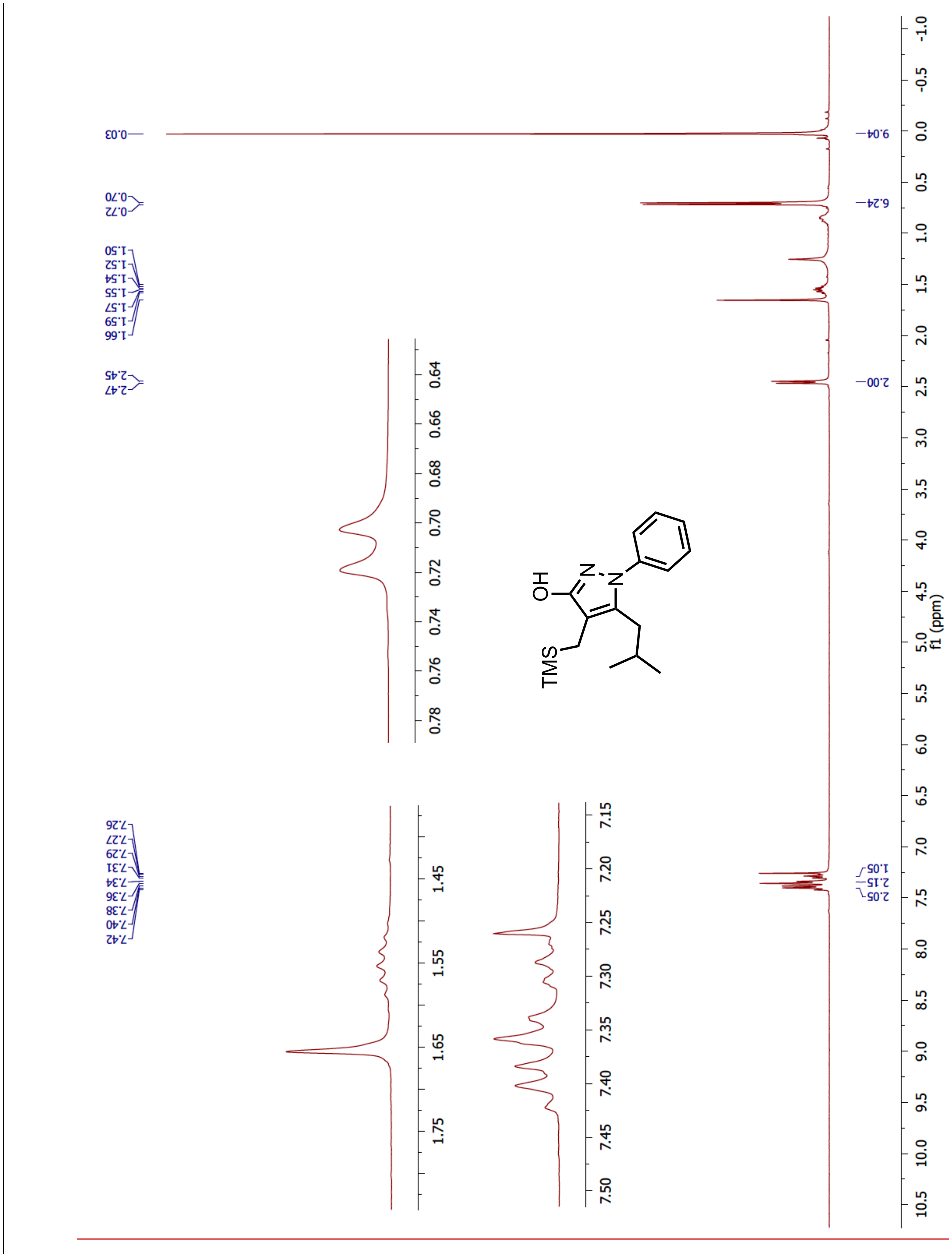

Figure S57. ^{1}H NMR spectrum of **12aa** in $CDCl_3$ (400 MHz).

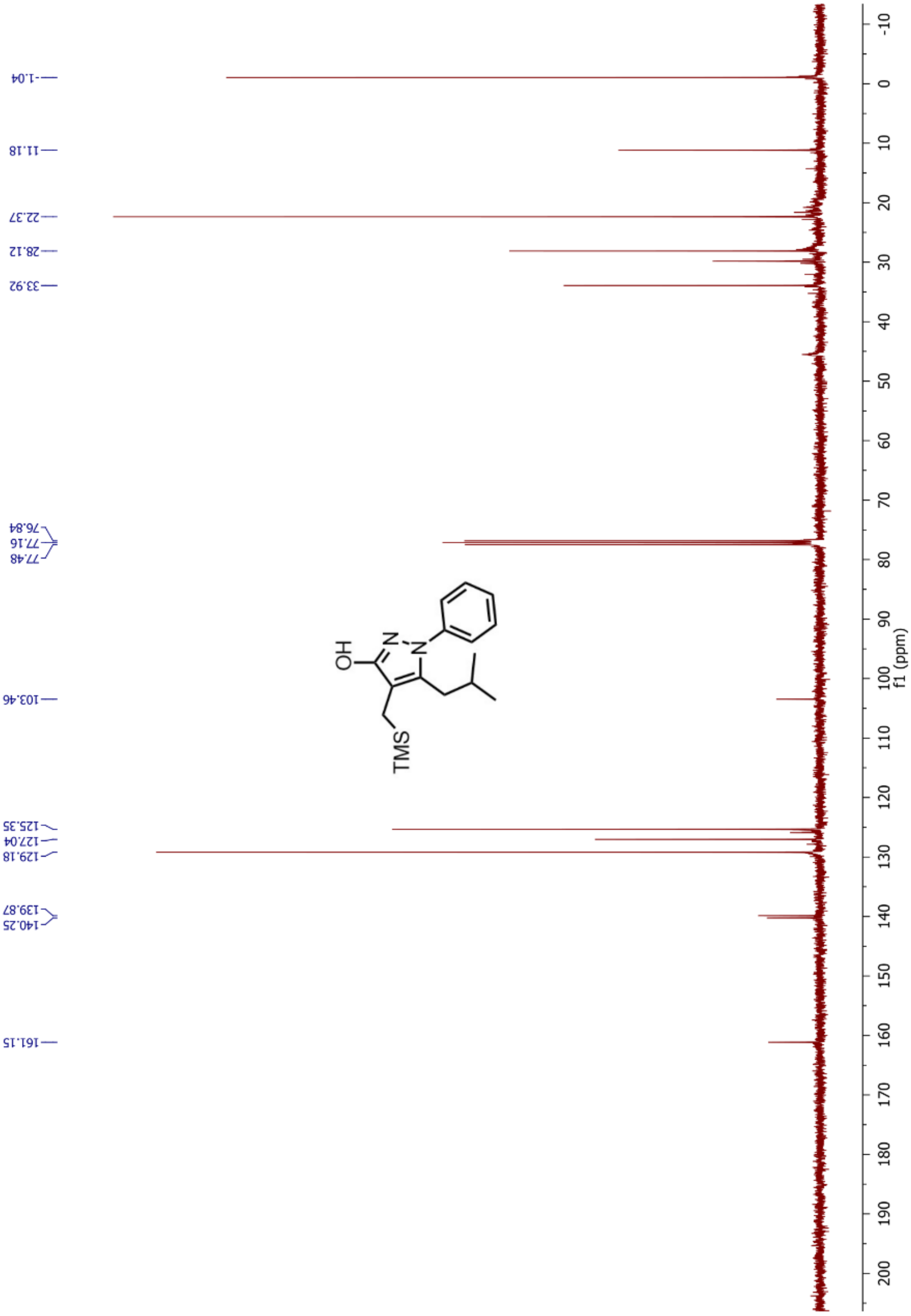

Figure S58. $^{13}C\{^{1}H\}$ NMR spectrum of **12aa** in $CDCl_3$ (100 MHz).

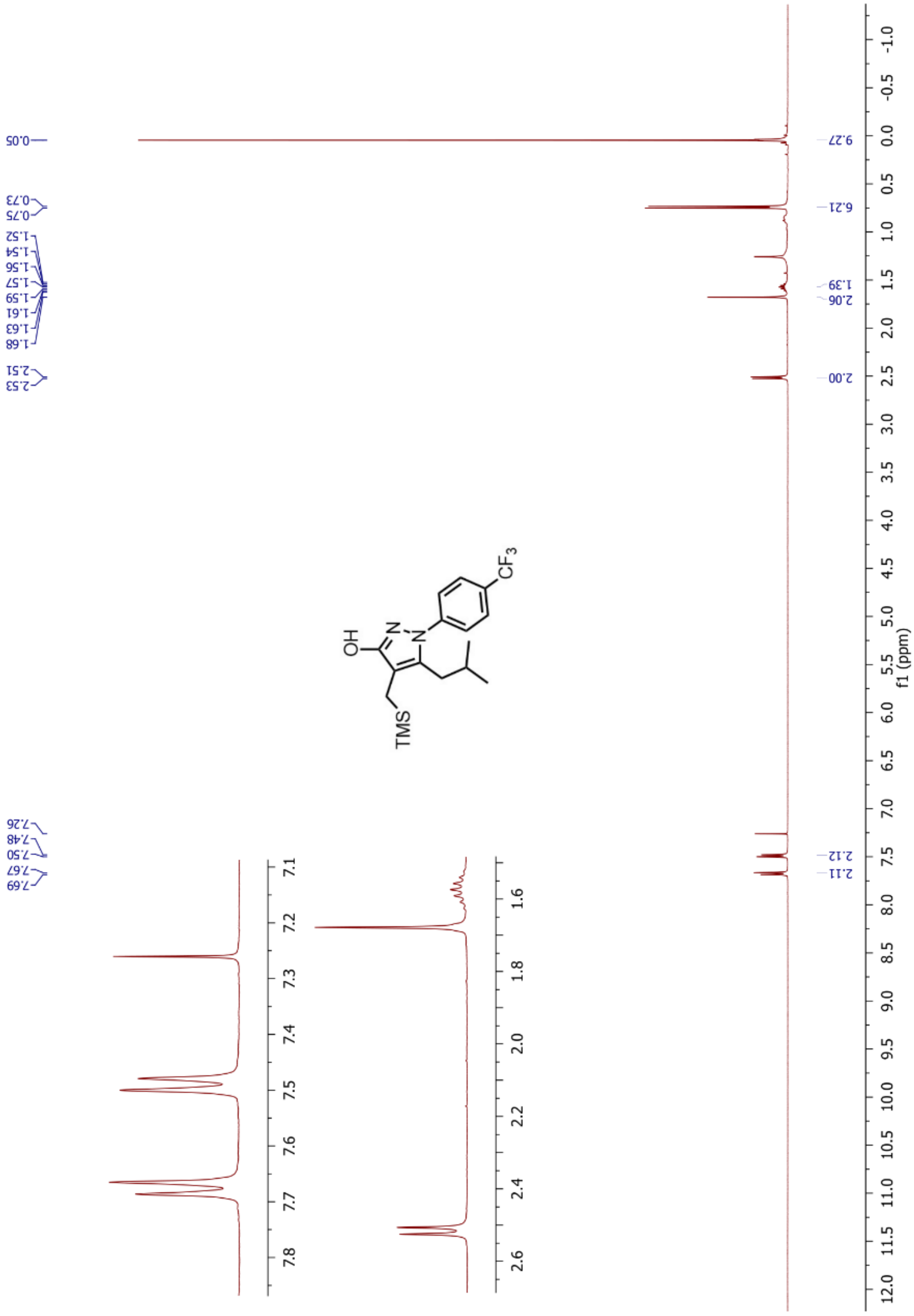

Figure S59. ^{1}H NMR spectrum of **12ab** in $CDCl_3$ (400 MHz). **3**

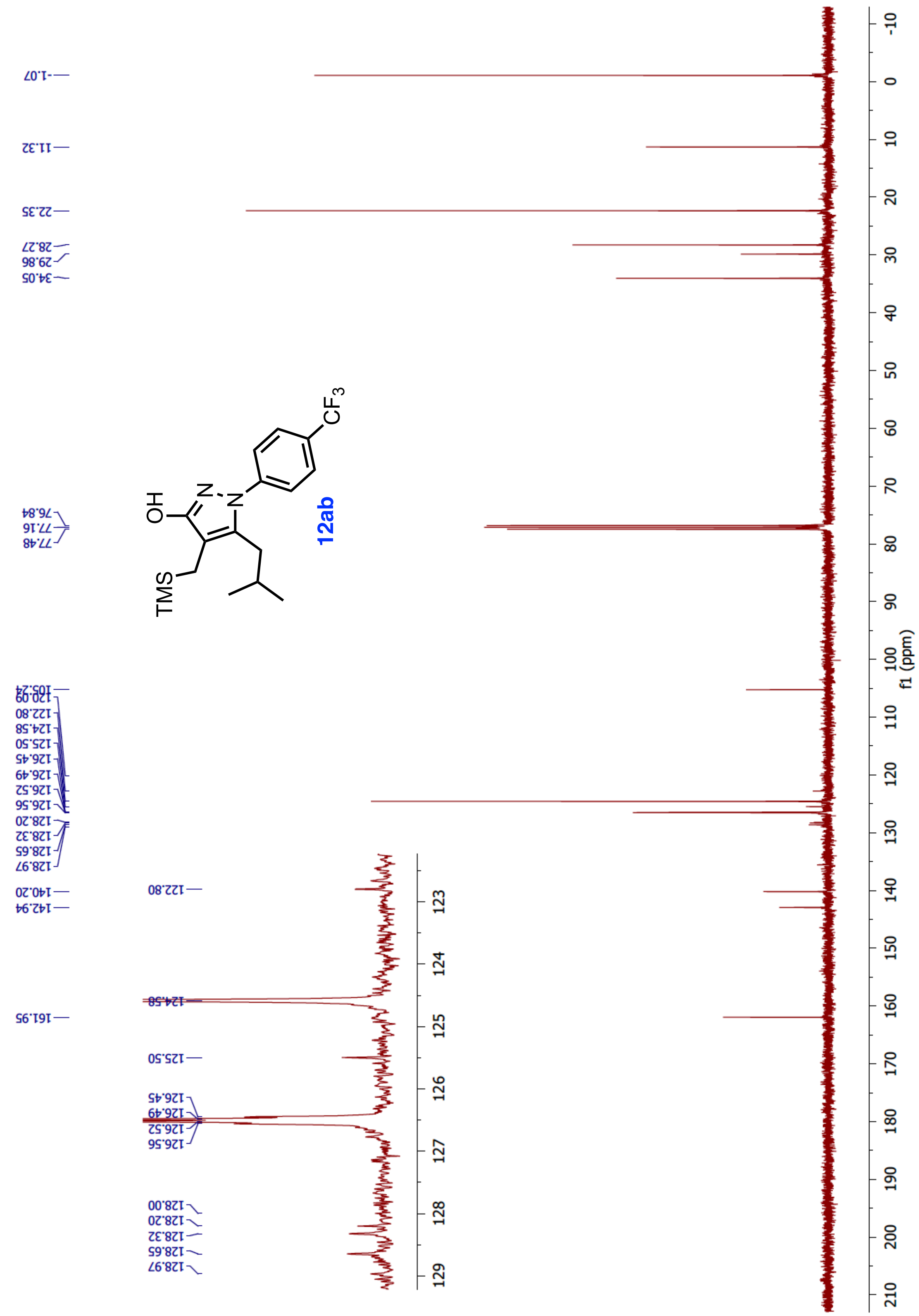

Figure S60. $^{13}C\{^{1}H\}$ NMR spectrum of **12ab** in $CDCl_3$ (100 MHz).

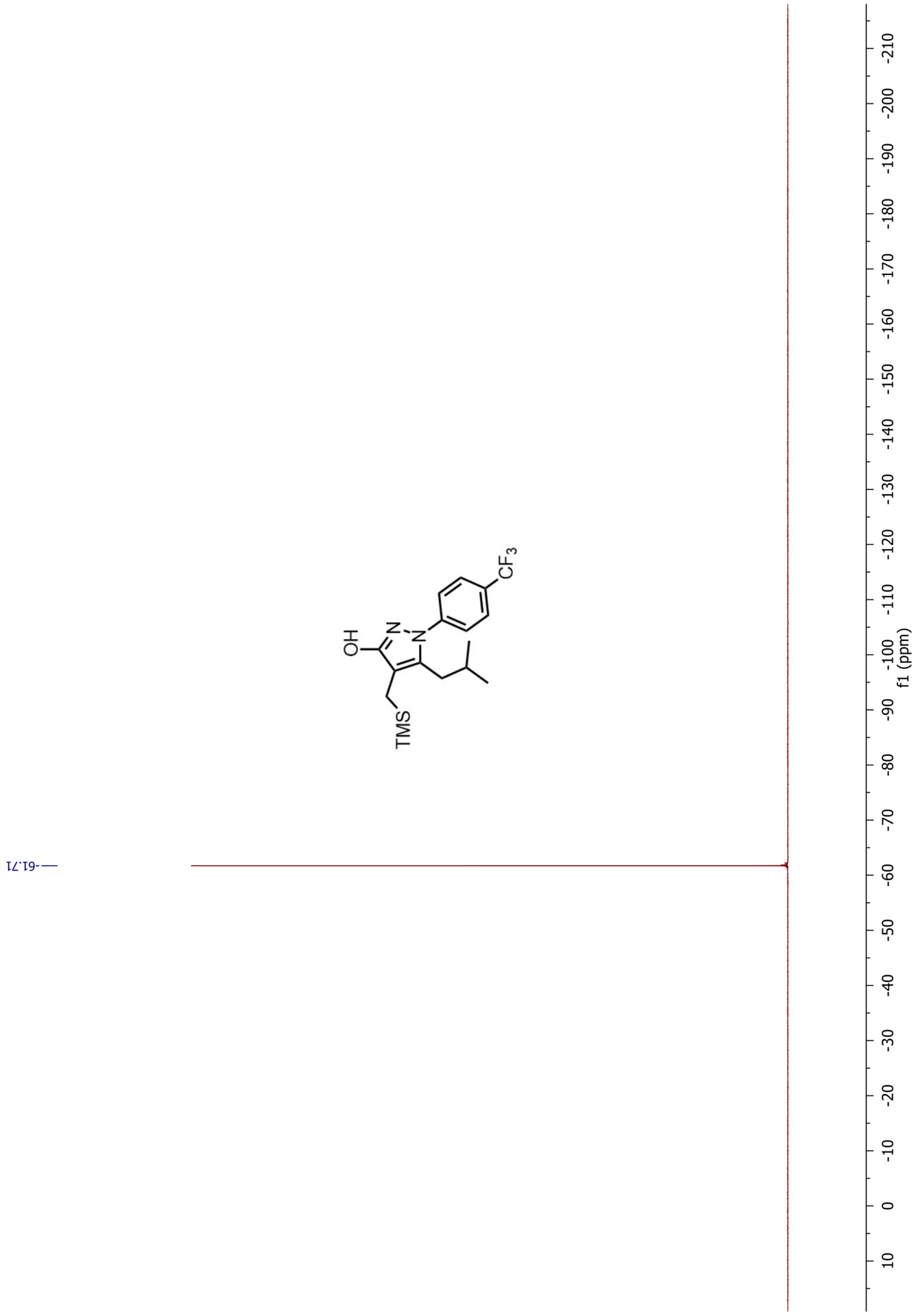

Figure S61. ^{19}F NMR spectrum of **12ab** in $CDCl_3$ (376 MHz).

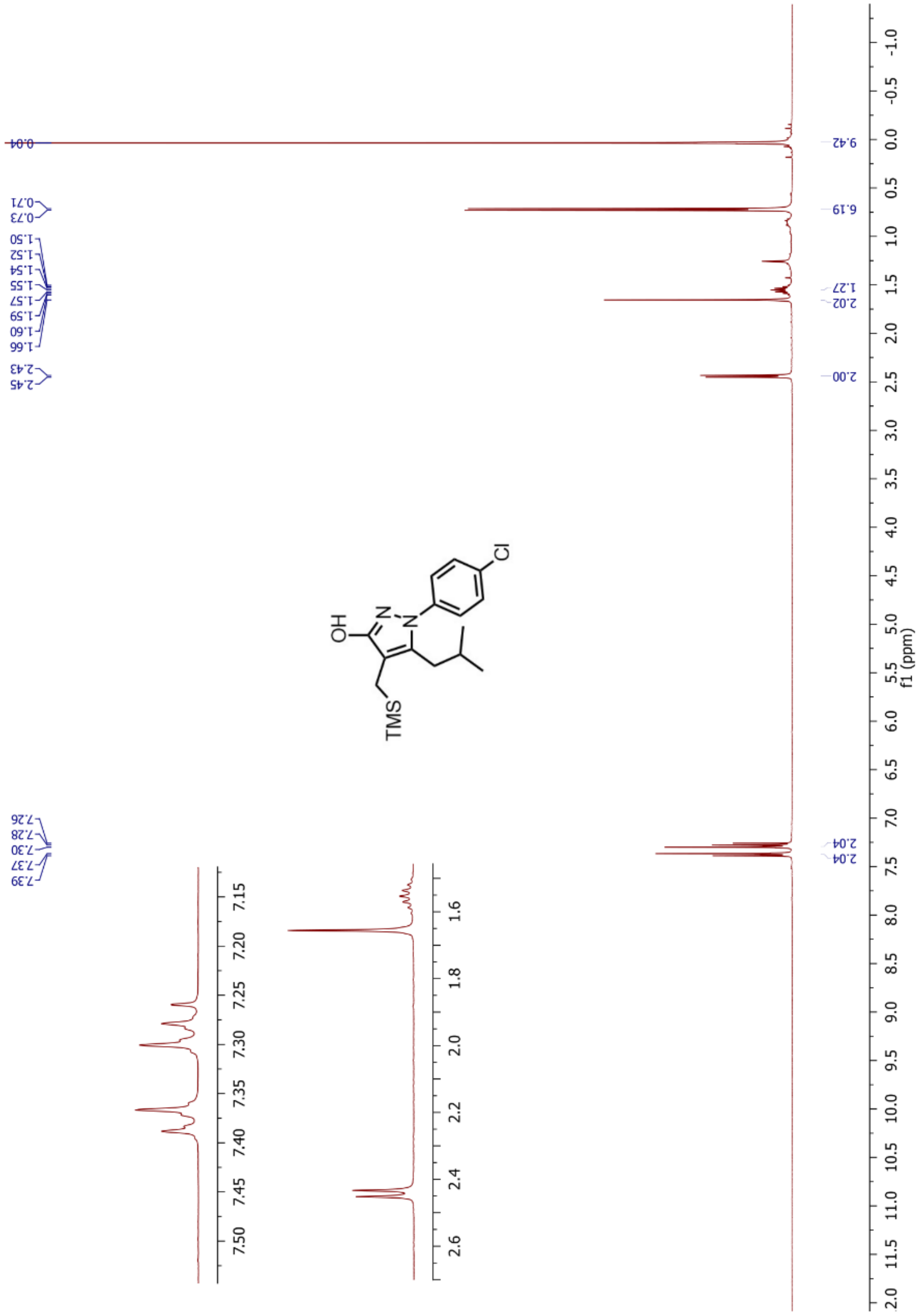

Figure S62. ^{1}H NMR spectrum of **12ac** in $CDCl_3$ (400 MHz).

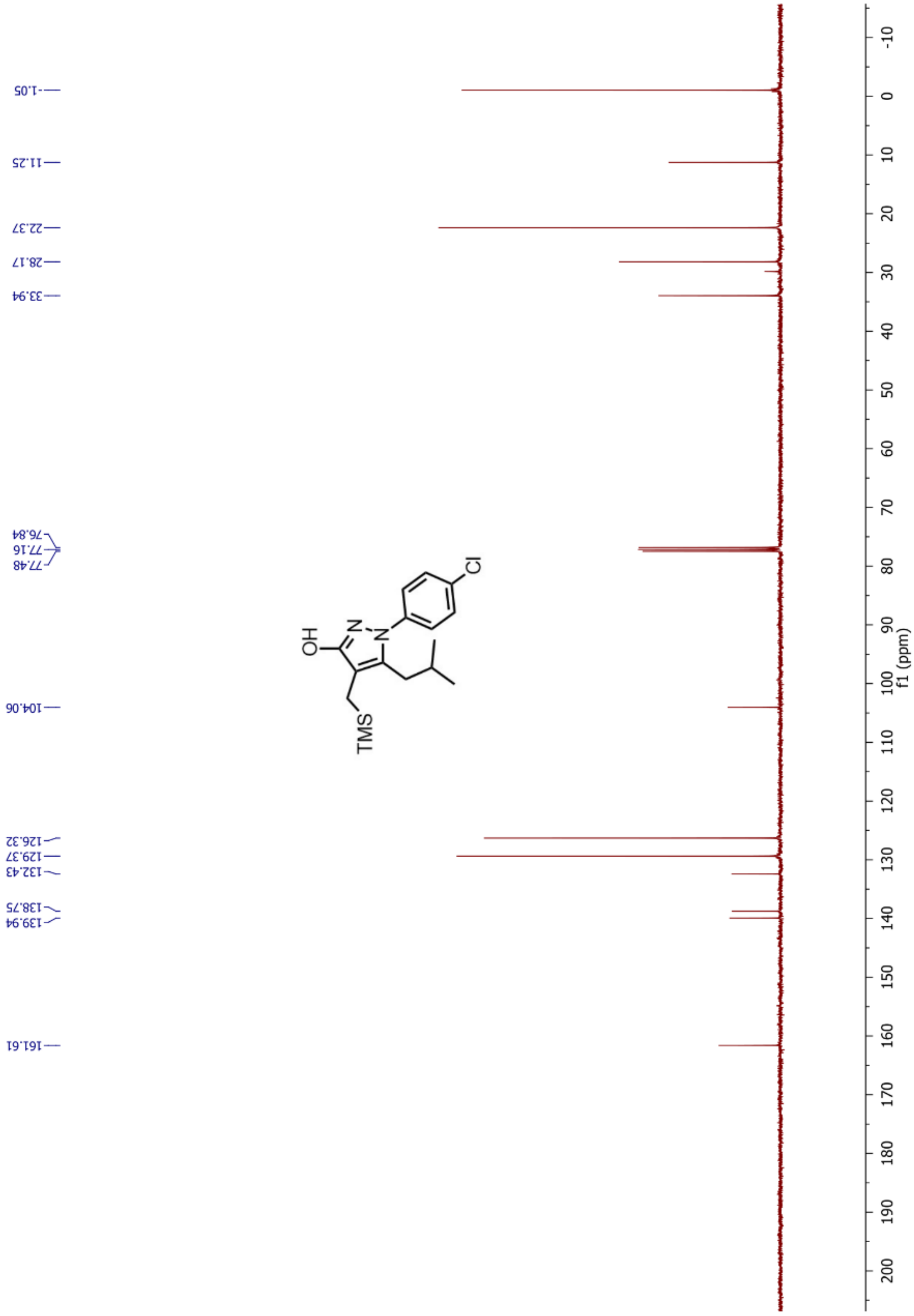

Figure S63. $^{13}C\{^{1}H\}$ NMR spectrum of **12ac** in $CDCl_3$ (100 MHz).

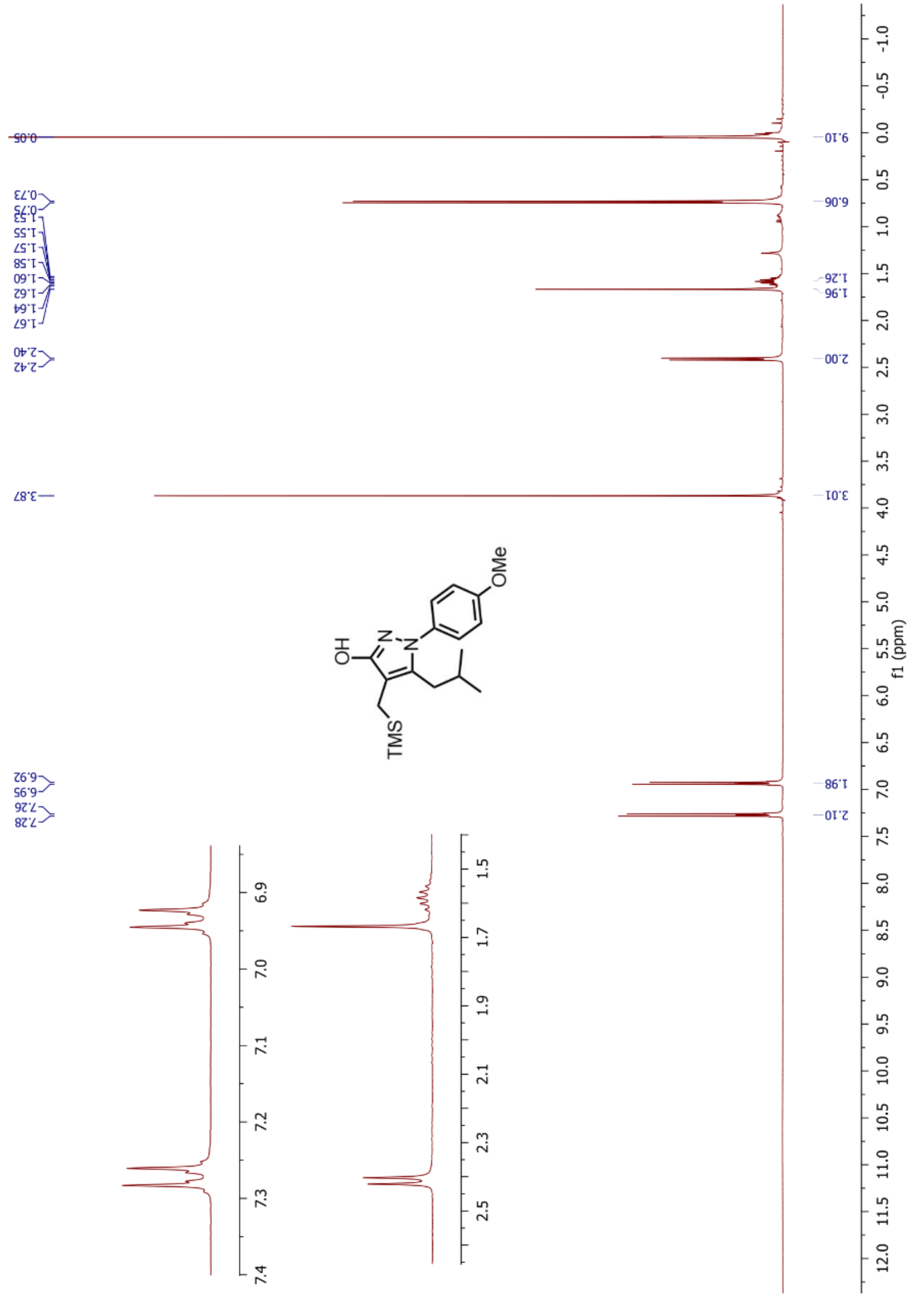

Figure S64. ^{1}H NMR spectrum of **12ad** in $CDCl_3$ (400 MHz).

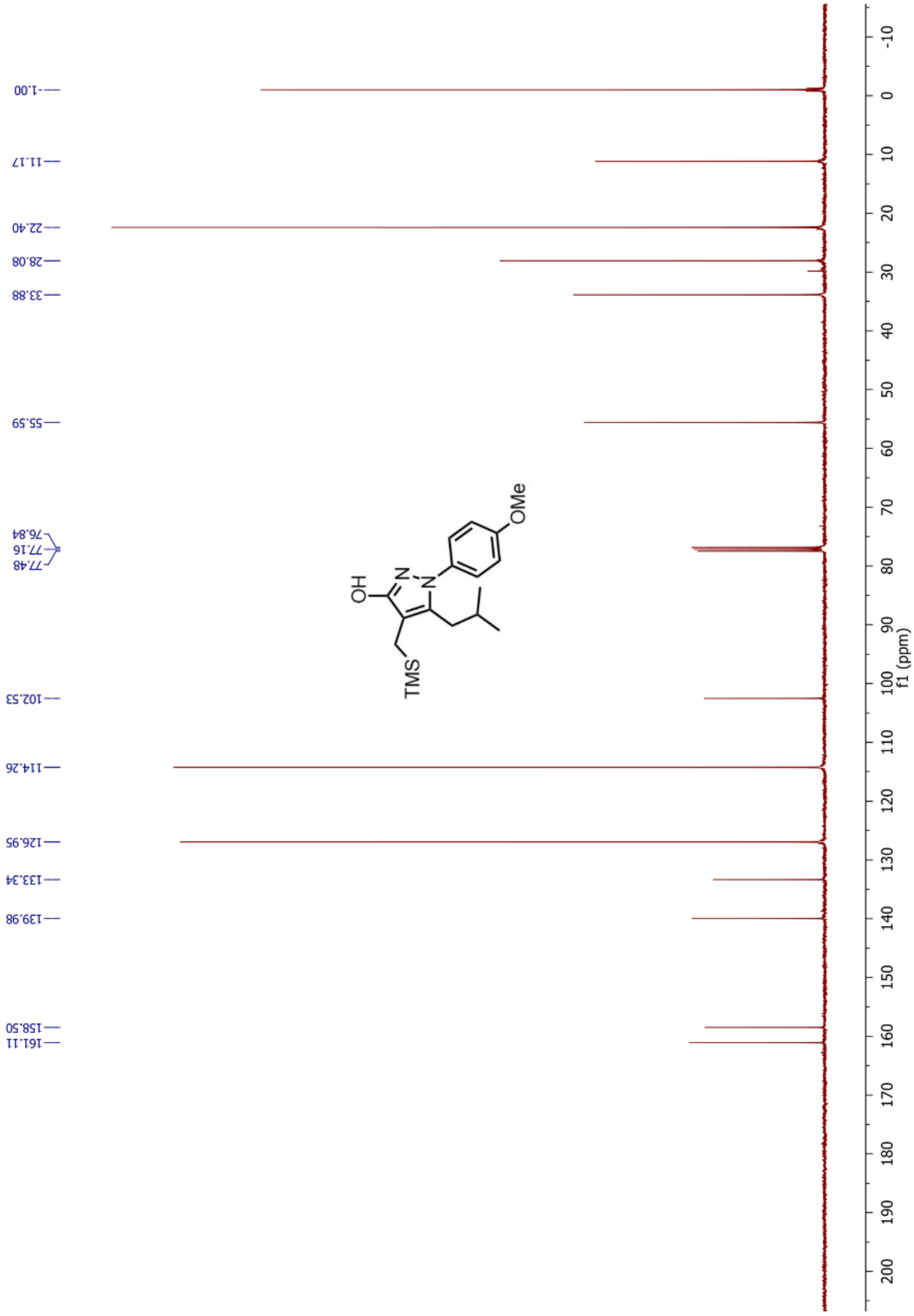

Figure S65. $^{13}C\{^1H\}$ NMR spectrum of **12ad** in $CDCl_3$ (100 MHz).

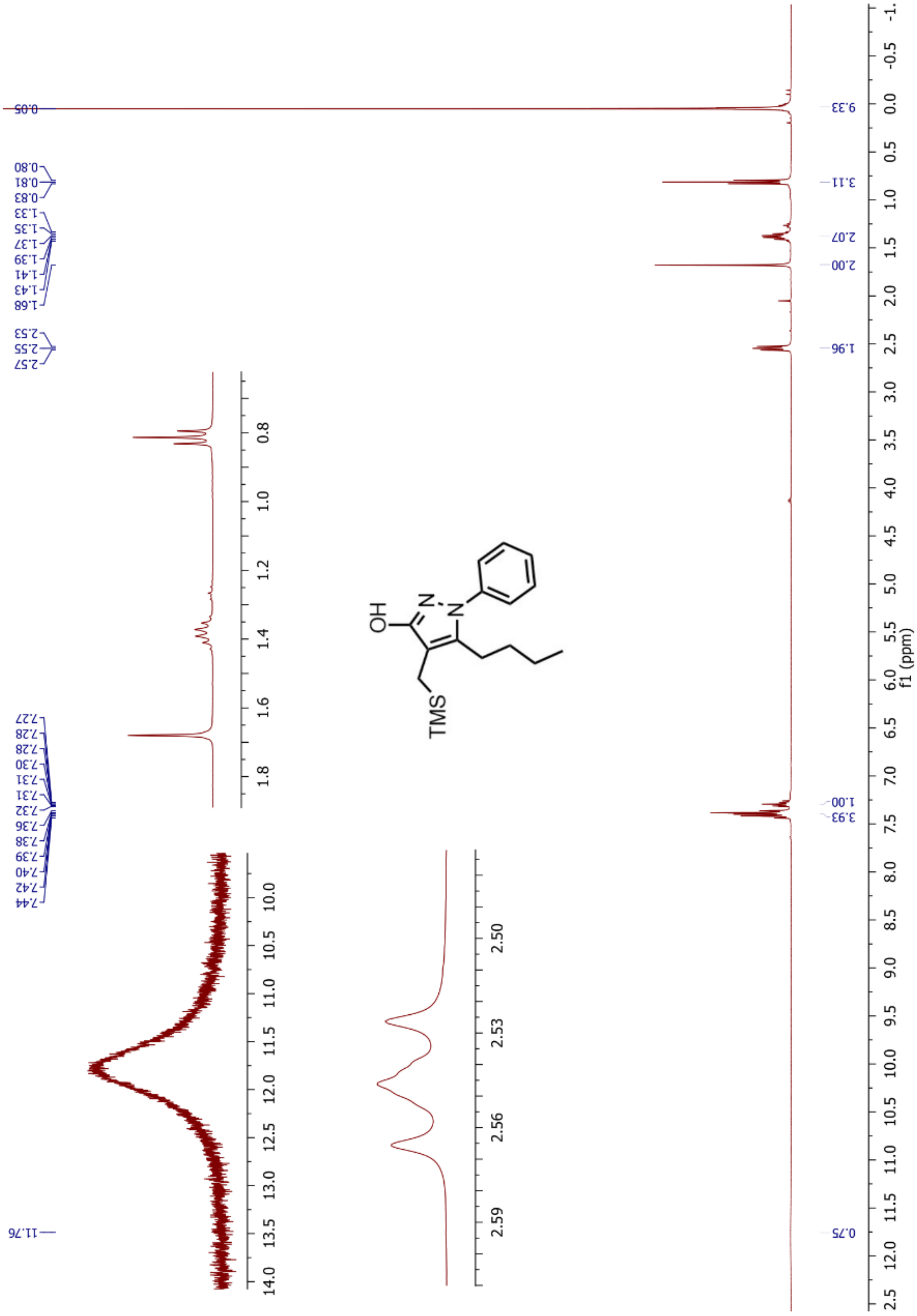

Figure S66. ^{1}H NMR spectrum of **12b** in $CDCl_3$ (400 MHz).

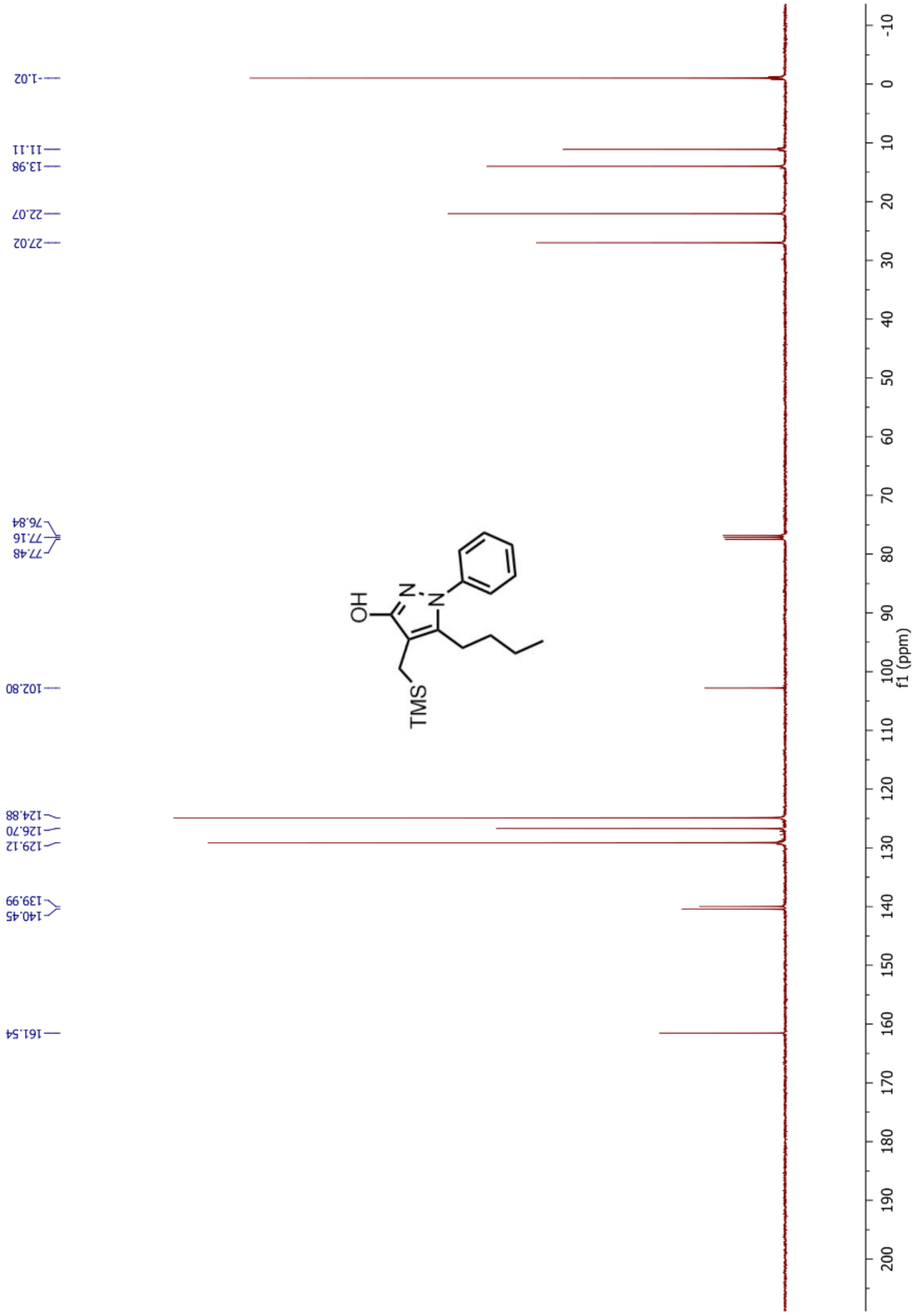

Figure S67. $^{13}C\{^{1}H\}$ NMR spectrum of **12b** in $CDCl_3$ (100 MHz).

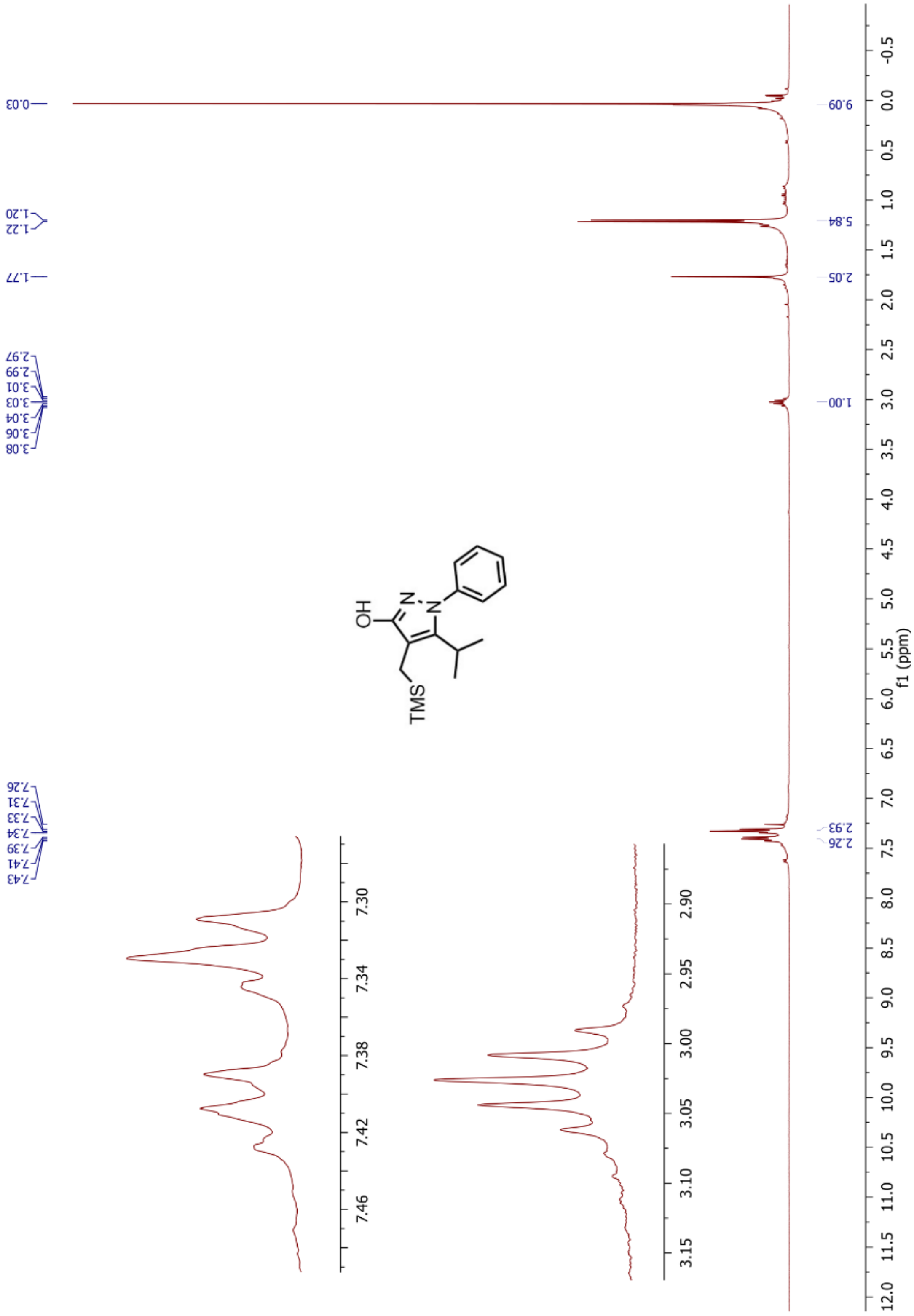

Figure S68. ^{1}H NMR spectrum of **12c** in $CDCl_3$ (400 MHz).

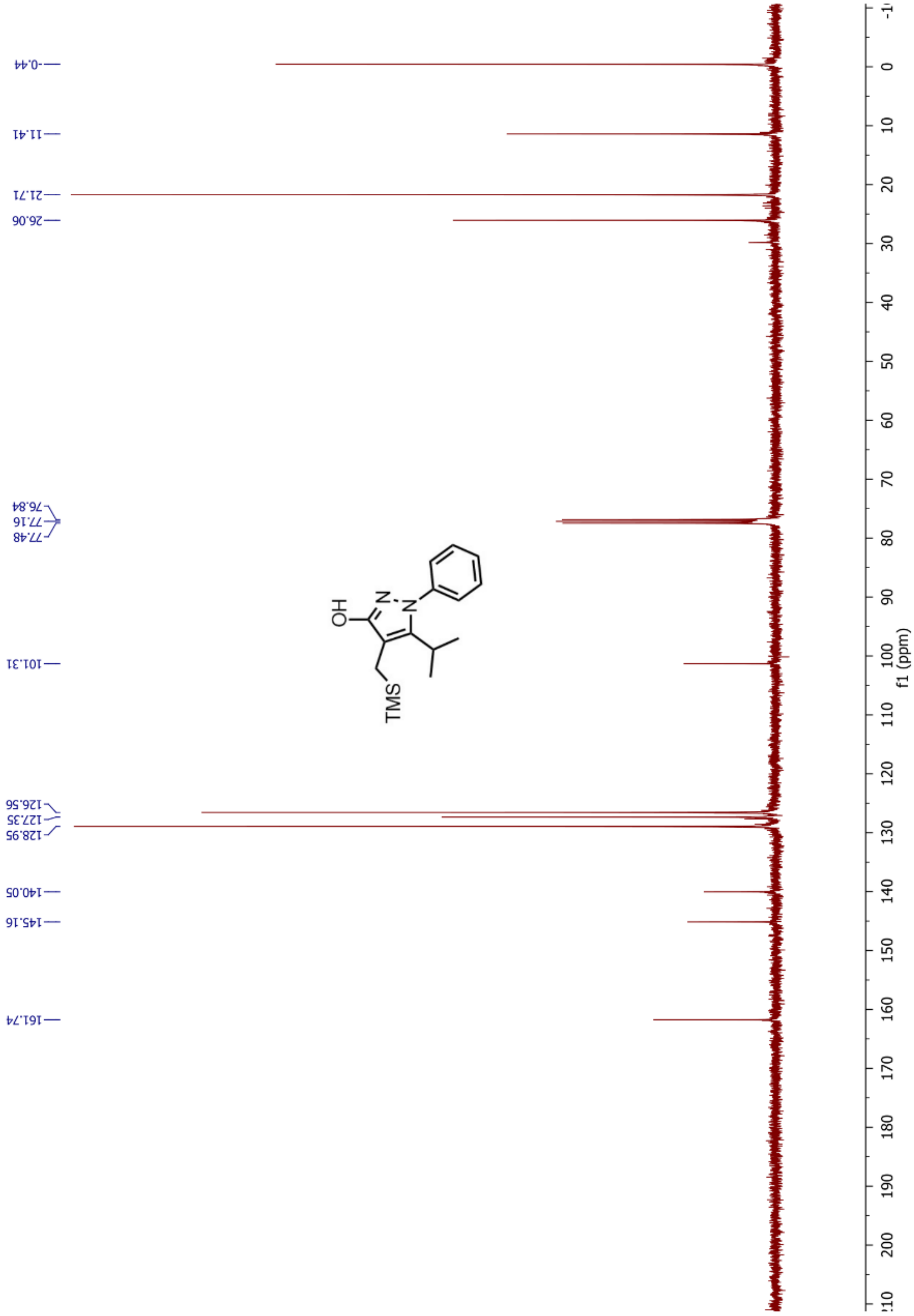

Figure S69. $^{13}C\{^{1}H\}$ NMR spectrum of **12c** in $CDCl_3$ (100 MHz).

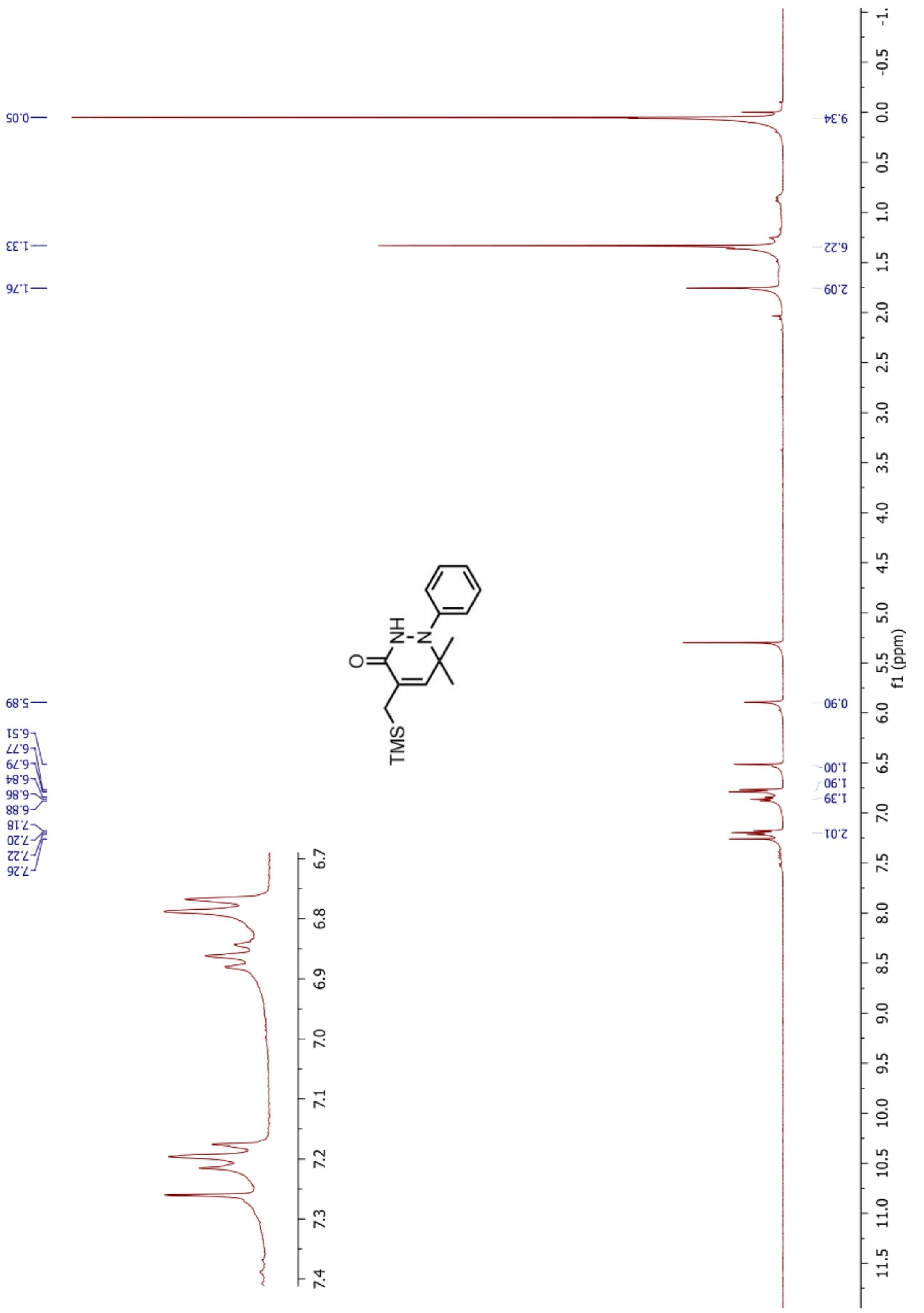

Figure S70. ^{1}H NMR spectrum of **20a** in $CDCl_3$ (400 MHz).

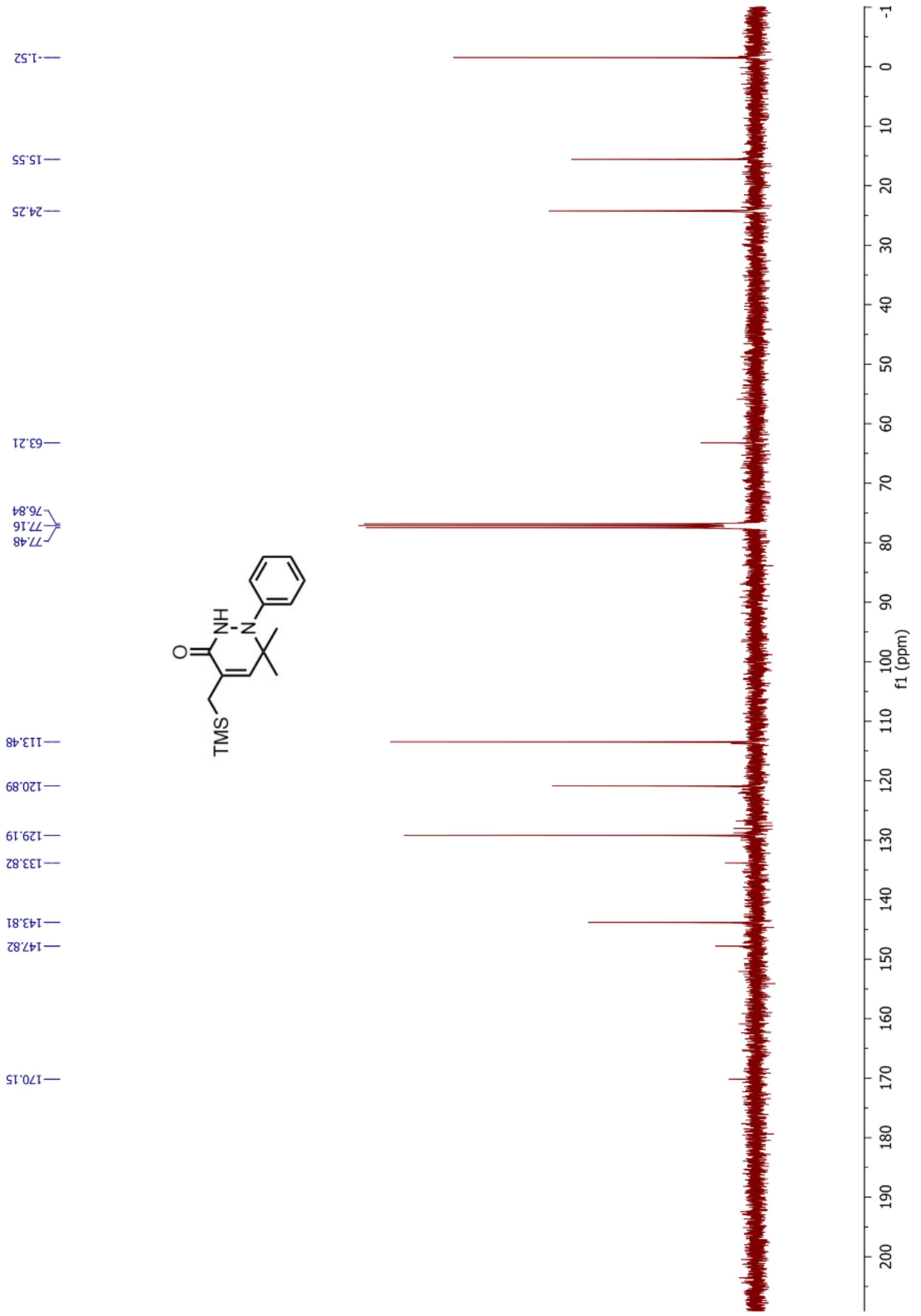

Figure S71. $^{13}C\{^{1}H\}$ NMR spectrum of **20a** in $CDCl_3$ (100 MHz).

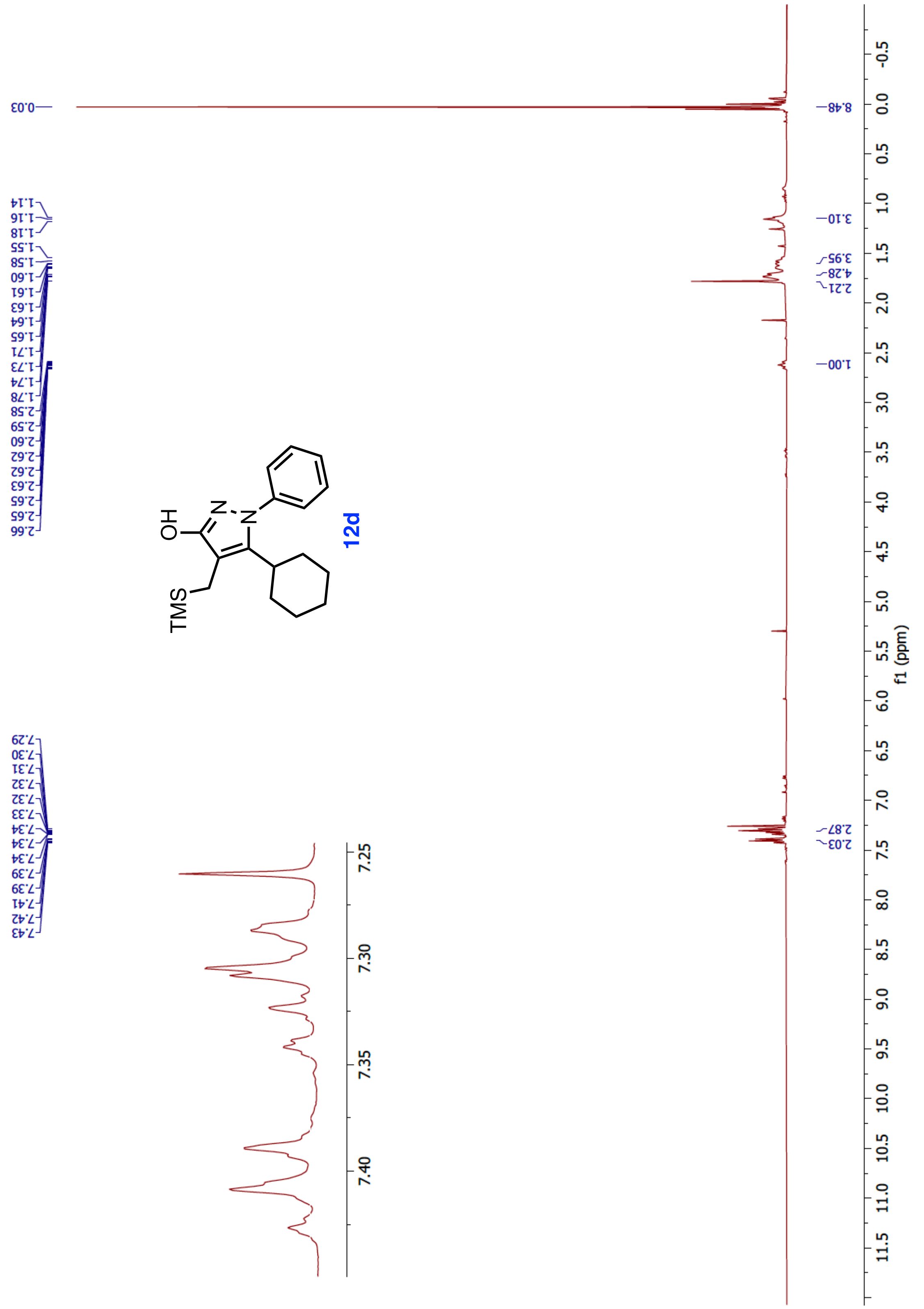

Figure S72. ^{1}H NMR spectrum of **12d** in $CDCl_3$ (400 MHz).

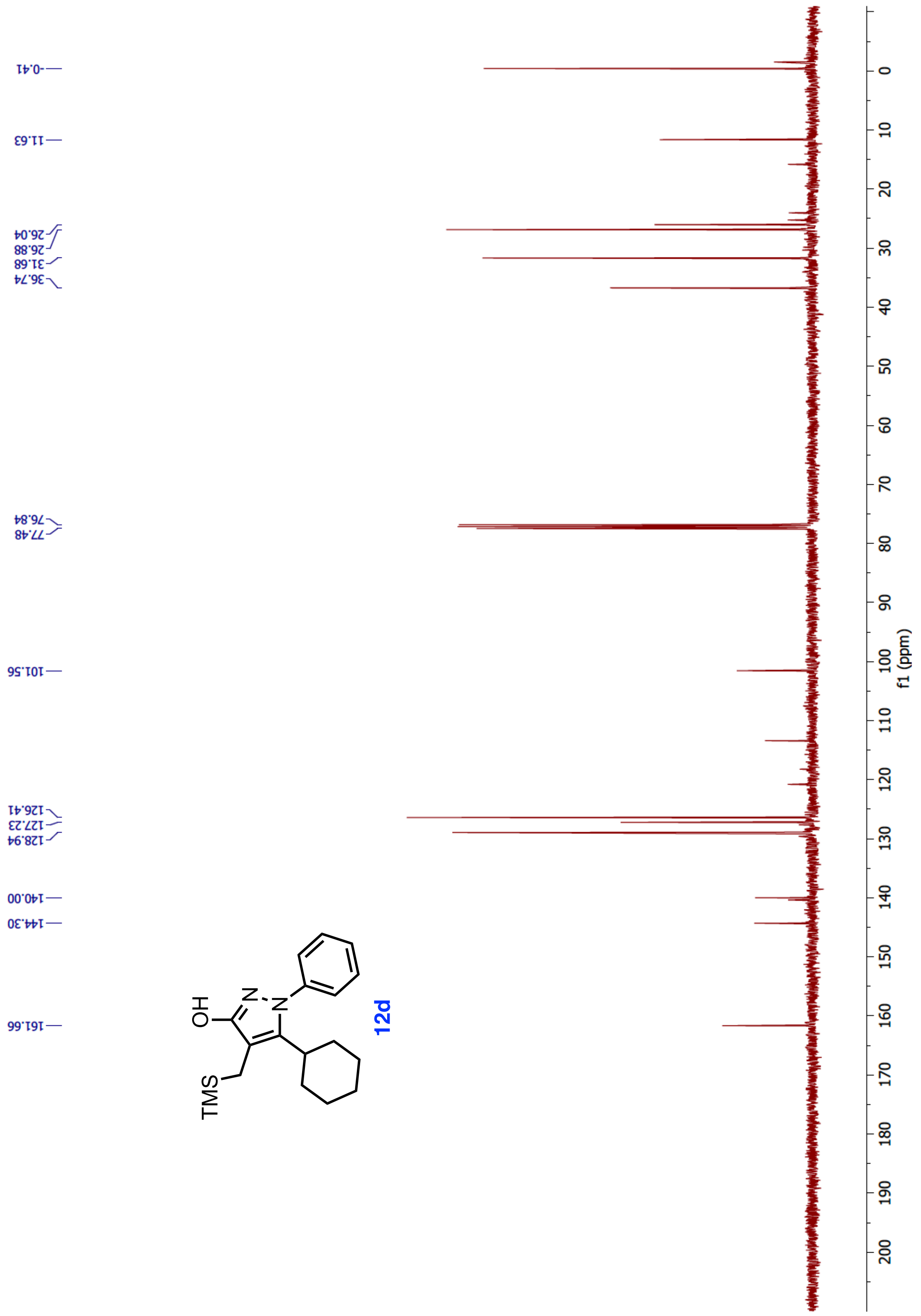

Figure S73. $^{13}C\{^{1}H\}$ NMR spectrum of **12d** in $CDCl_3$ (100 MHz).

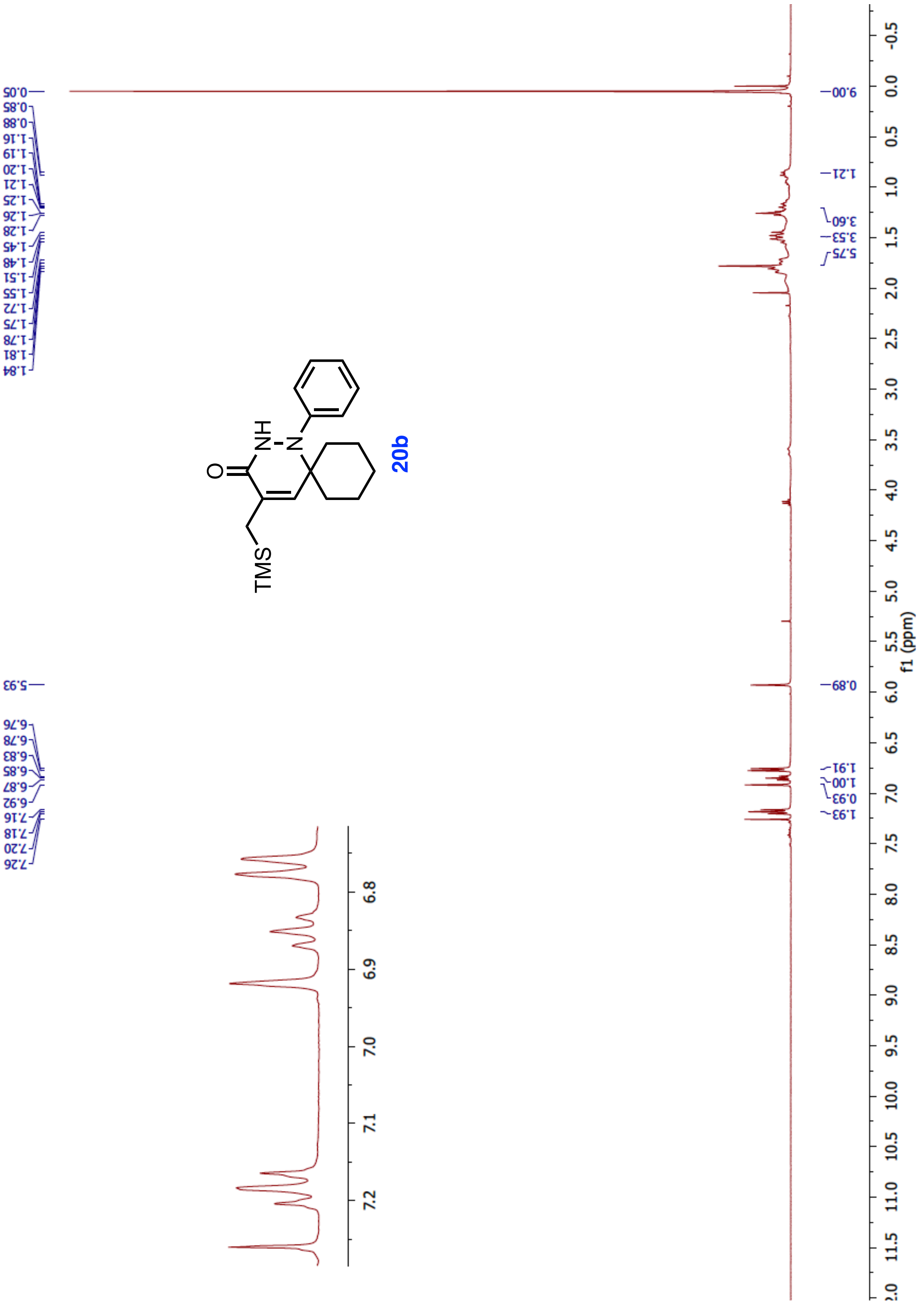

Figure S74. ^{1}H NMR spectrum of **20b** in $CDCl_3$ (400 MHz).

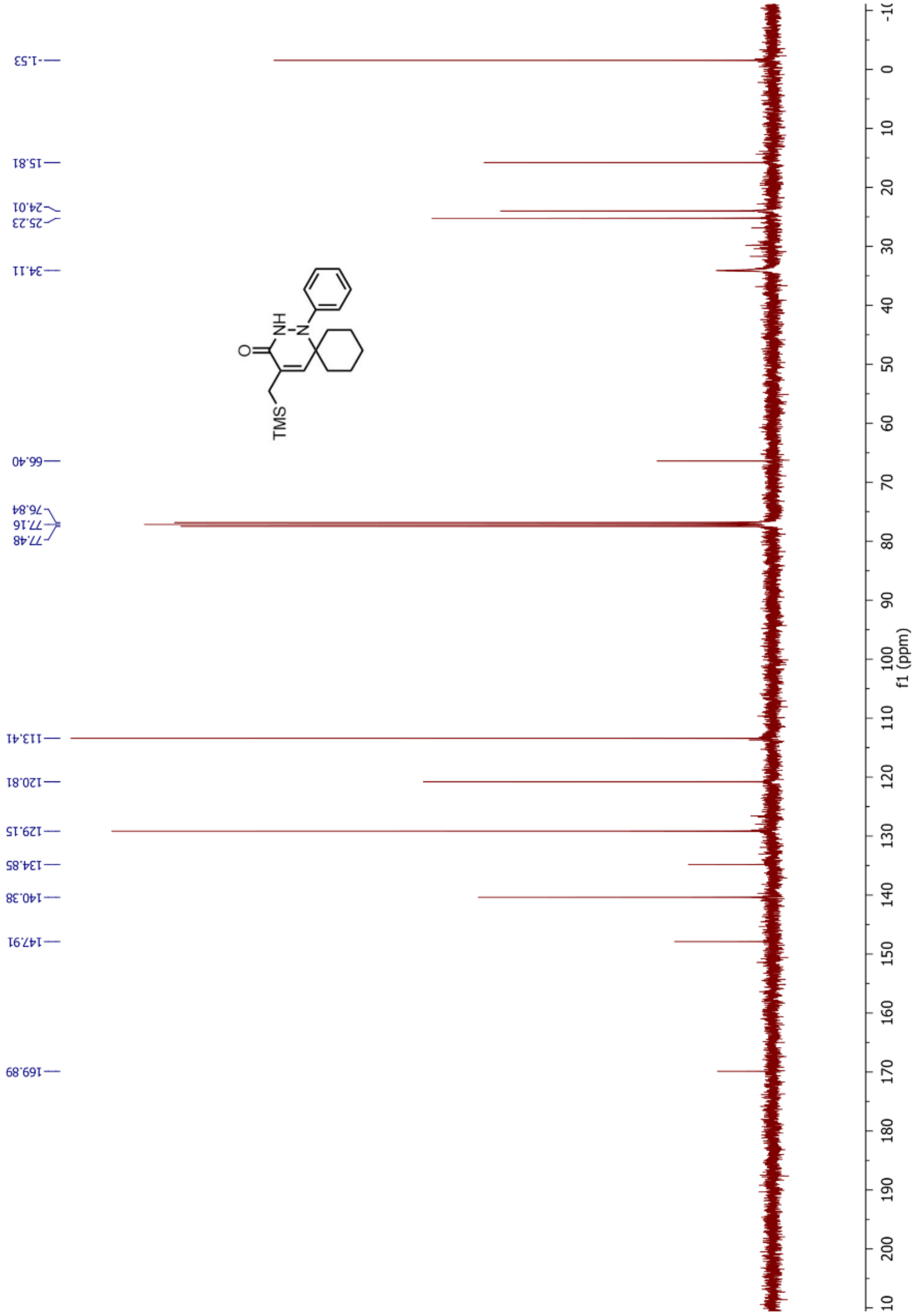

Figure S75. $^{13}C\{^{1}H\}$ NMR spectrum of **20b** in $CDCl_3$ (100 MHz).

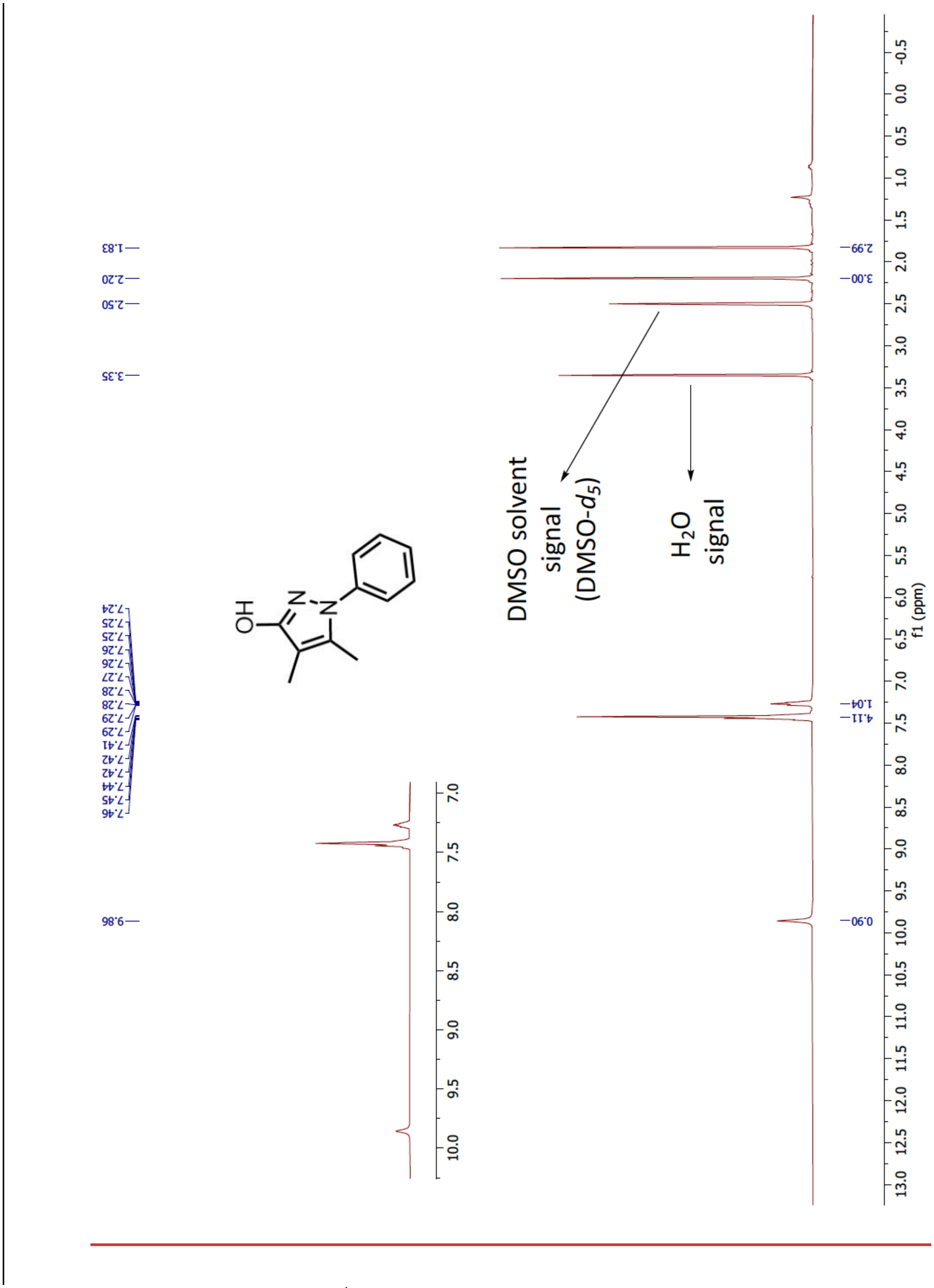

Figure S76. ^{1}H NMR spectrum of **21** in DMSO-d_6 (400 MHz).

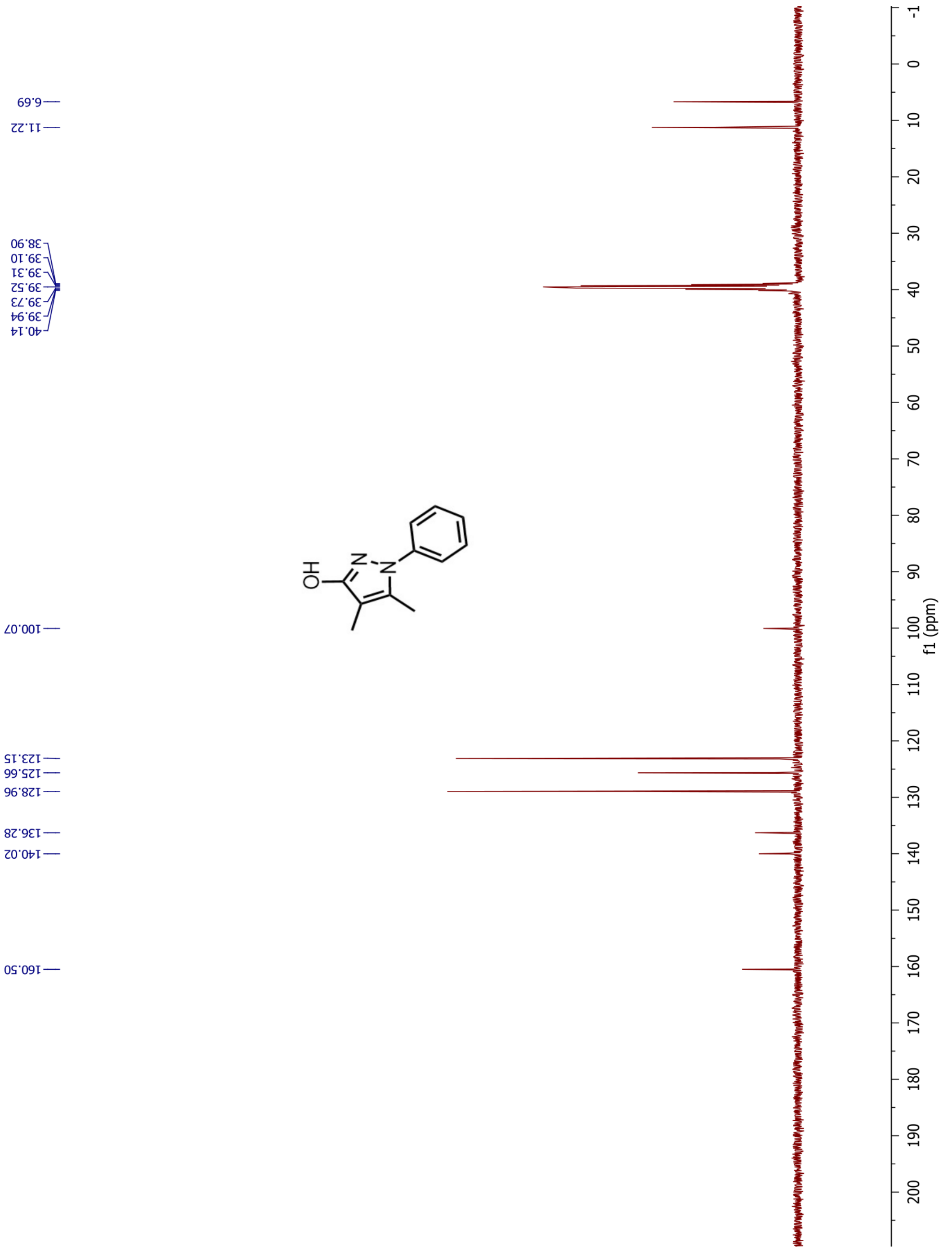

Figure S77. $^{13}C\{^{1}H\}$ NMR spectrum of **21** in DMSO-d_6 (100 MHz).

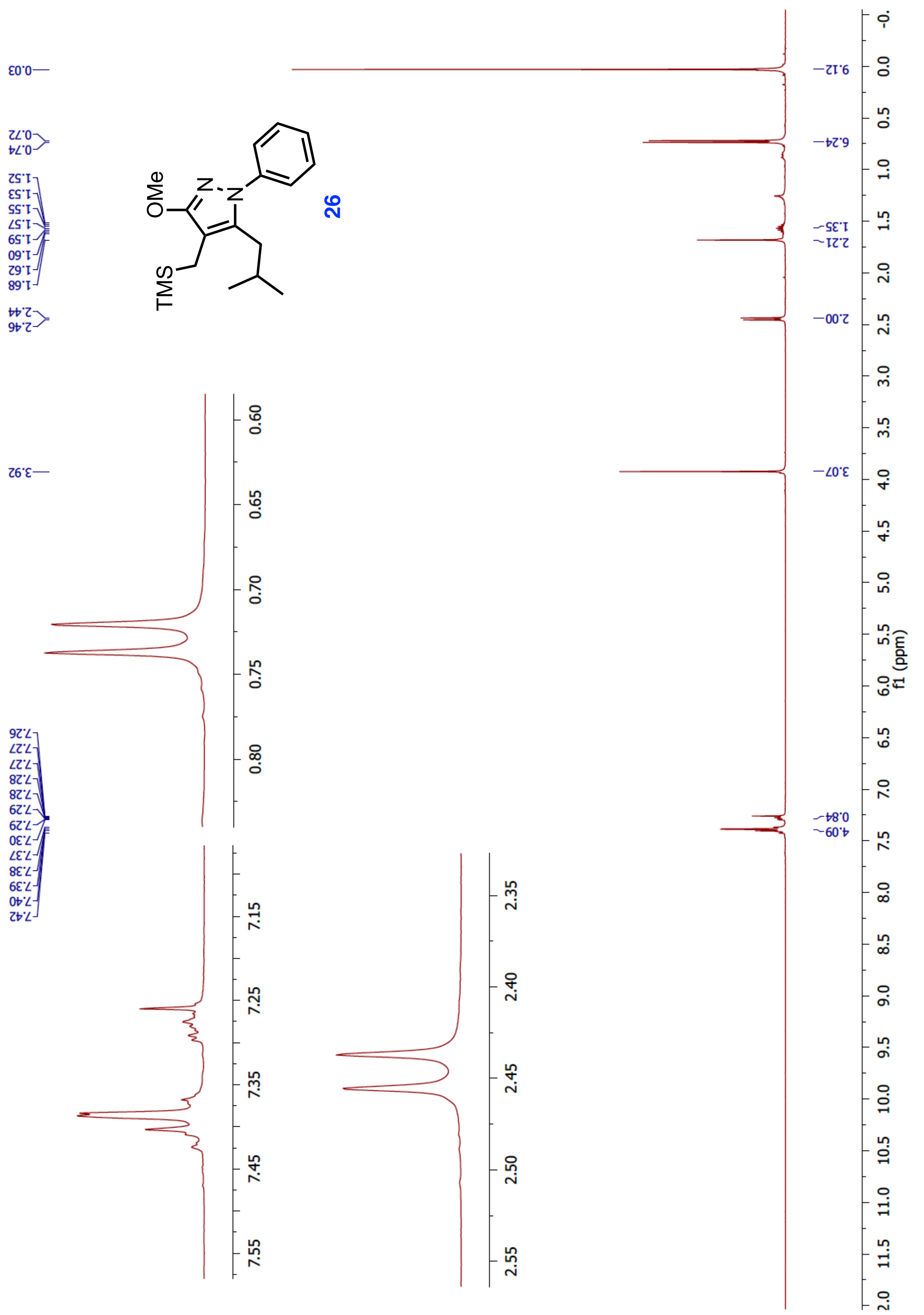

Figure S78. ^{1}H NMR spectrum of **26** in $CDCl_3$ (400 MHz).

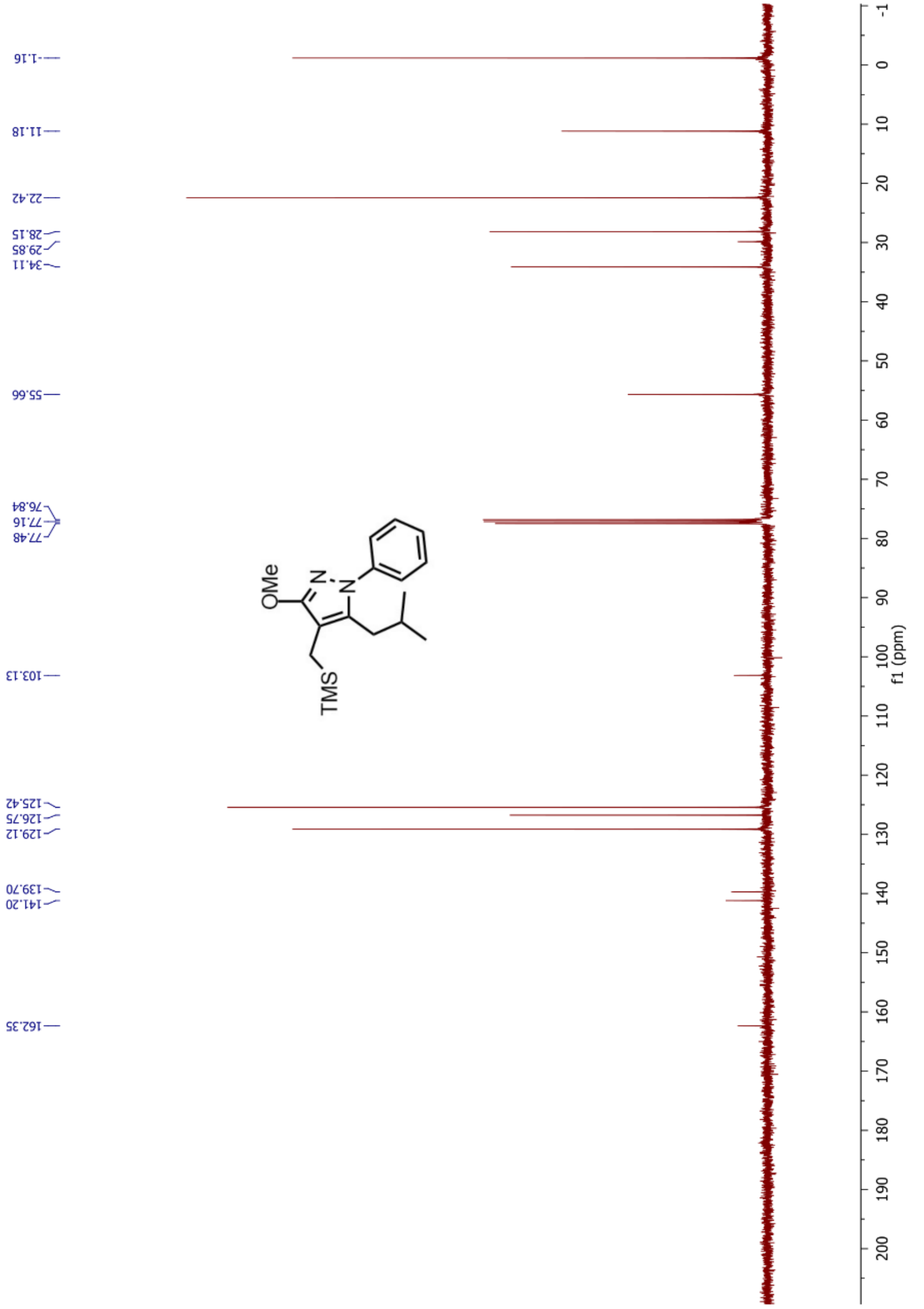

Figure S79. $^{13}C\{^{1}H\}$ NMR spectrum of **26** in $CDCl_3$ (100 MHz).

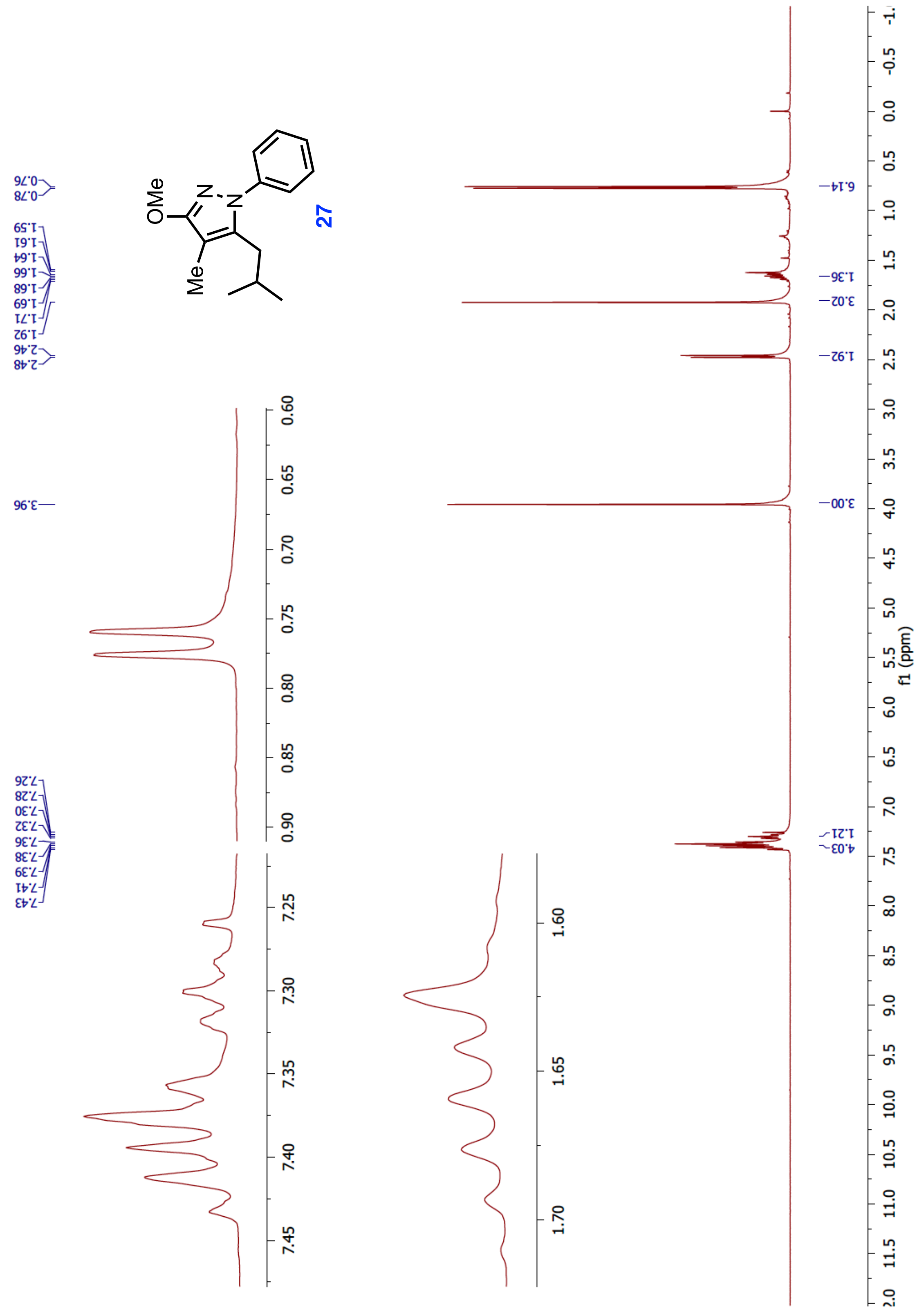

Figure S80. ^{1}H NMR spectrum of **27** in $CDCl_3$ (400 MHz).

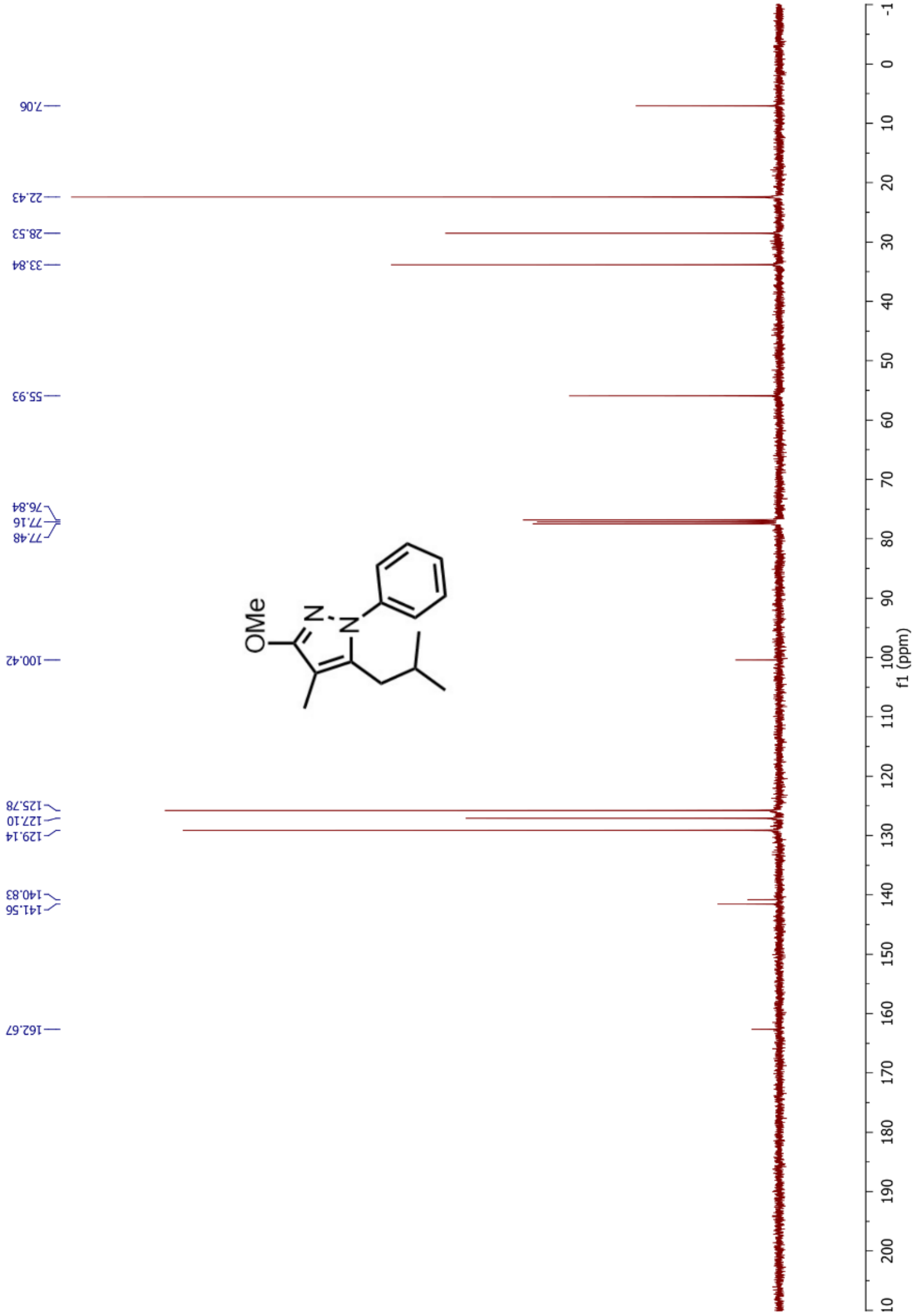

Figure S81. $^{13}C\{^{1}H\}$ NMR spectrum of **27** in $CDCl_3$ (100 MHz).

HRMS Data:

Qualitative Analysis Report

**Data Filename**	UMK-1-258b 10.d	**Sample Name**	sample
**Sample Type**	Sample	**Position**	Vial 34
**Instrument Name**	Instrument 1	**User Name**	
**Acq Method**	without column.m	**Acquired Time**	2/26/2025 10:45:59 AM
**IRM Calibration Status**	Success	**DA Method**	111.m
**Comment**			

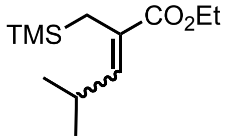

14c

**Method part to run:**	Acquisition Only	**Sample Group**	
**Info.**		**Stream Name**	LC 1
**Acquisition SW Version**	6200 series TOF/6500 series Q-TOF B.09.00 (B9044.0)		

User Chromatograms

Fragmentor Voltage 120 **Collision Energy** 0 **Ionization Mode** APCI

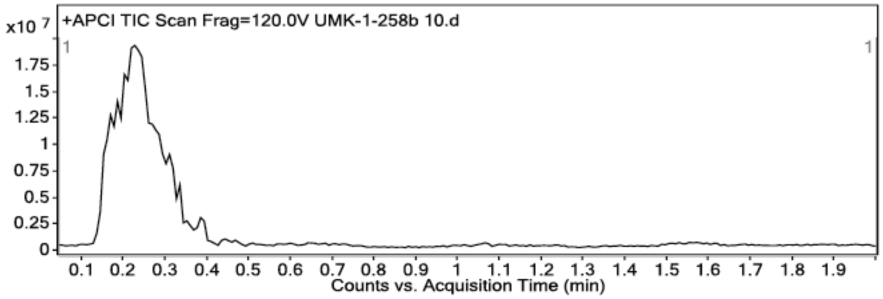

User Spectra

Fragmentor Voltage	Collision Energy	Ionization Mode
120	0	APCI

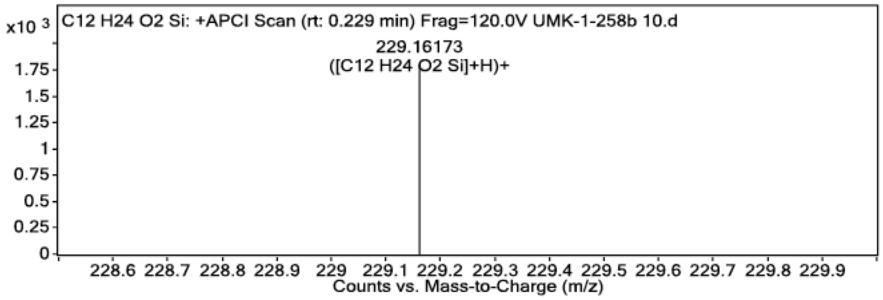

Peak List

m/z	z	Abund	Formula	Ion
73.01388		1327.37		
111.08236	1	18450.63		
112.0852	1	2213.44		
114.04053		920.86		
122.03721		715.84		
123.04722		868.78		
132.05268		420.44		
137.06832		490.48		

Agilent Technologies Page 1 of 3 Printed at: 10:51 AM on: 2/26/2025

Figure S82. High resolution mass spectrum of **14c**.

Qualitative Analysis Report

**Data Filename**	UMK-1-262c 11.d	**Sample Name**	sample
**Sample Type**	Sample	**Position**	Vial 35
**Instrument Name**	Instrument 1	**User Name**	
**Acq Method**	without column.m	**Acquired Time**	2/26/2025 10:49:09 AM
**IRM Calibration Status**	Success	**DA Method**	111.m
**Comment**			

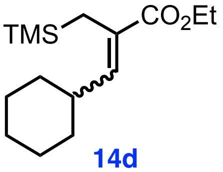

14d

**Method part to run: Info.**	Acquisition Only	**Sample Group**	
		**Stream Name**	LC 1
**Acquisition SW Version**	6200 series TOF/6500 series Q-TOF B.09.00 (B9044.0)		

User Chromatograms

Fragmentor Voltage 120 **Collision Energy** 0 **Ionization Mode** APCI

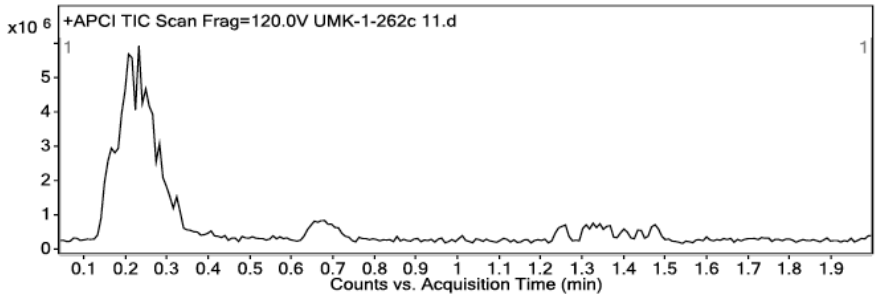

User Spectra

Fragmentor Voltage	Collision Energy	Ionization Mode
120	0	APCI

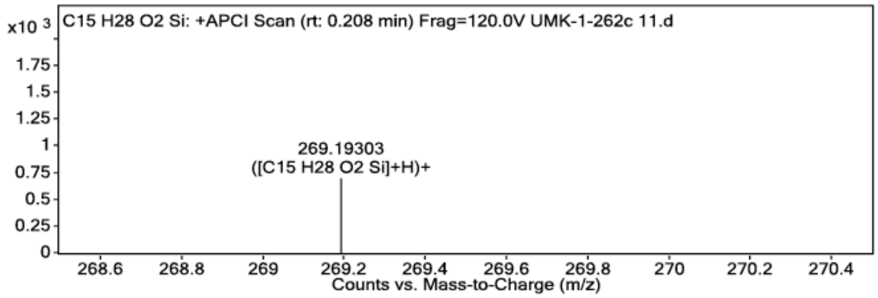

Peak List

m/z	z	Abund	Formula	Ion
73.01355		713.03		
114.03661		429.29		
121.06586		1274.43		
123.04727		350.72		
148.9977		782.64		
179.05955		358.73		
195.14483		1241.23		
223.14749	1	8859.97		

Agilent Technologies Page 1 of 3 Printed at: 10:55 AM on: 2/26/2025

Figure S83. High resolution mass spectrum of **14d**.

Qualitative Analysis Report

**Data Filename**	UMK-1-288b 02.d	**Sample Name**	sample
**Sample Type**	Sample	**Position**	Vial 2
**Instrument Name**	Instrument 1	**User Name**	
**Acq Method**	without column.m	**Acquired Time**	4/8/2025 2:12:12 PM
**IRM Calibration Status**	Success	**DA Method**	111.m
**Comment**			

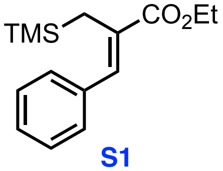

S1

**Method part to run:**	Acquisition Only	**Sample Group**	
**Info.**		**Stream Name**	LC 1
**Acquisition SW Version**	6200 series TOF/6500 series Q-TOF B.09.00 (B9044.0)		

User Chromatograms

Fragmentor Voltage 120 **Collision Energy** 0 **Ionization Mode** APCI

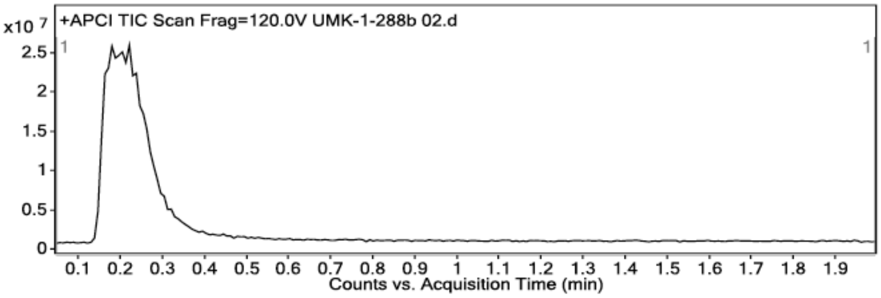

User Spectra

Fragmentor Voltage	Collision Energy	Ionization Mode
120	0	APCI

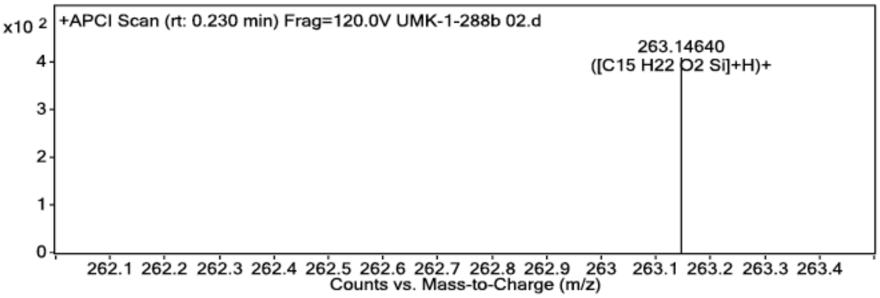

Peak List

m/z	z	Abund	Formula	Ion
73.0263		1731.21		
114.05009		8279.48		
115.05159		1257.33		
117.0458		525.61		
123.057		2071.24		
125.06483		565.03		
131.06506		1641.7		
132.06356		418.63		

Agilent Technologies　　Page 1 of 3　　Printed at: 2:23 PM on: 4/8/2025

Figure S84. High resolution mass spectrum of **S1**.

Qualitative Analysis Report

**Data Filename**	UMK-1-270b 05.d	**Sample Name**	sample
**Sample Type**	Sample	**Position**	Vial 4
**Instrument Name**	Instrument 1	**User Name**	
**Acq Method**	without column.m	**Acquired Time**	3/17/2025 4:48:00 PM
**IRM Calibration Status**	Success	**DA Method**	111.m
**Comment**			

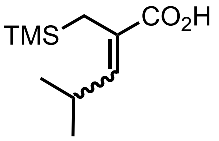

15c

**Method part to run:**	Acquisition Only	**Sample Group**	
**Info.**		**Stream Name**	LC 1
**Acquisition SW Version**	6200 series TOF/6500 series Q-TOF B.09.00 (B9044.0)		

User Chromatograms

Fragmentor Voltage 150 **Collision Energy** 0 **Ionization Mode** ESI

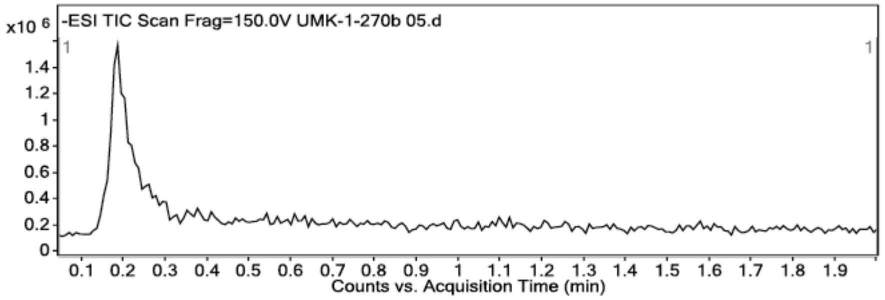

User Spectra

Fragmentor Voltage	Collision Energy	Ionization Mode
150	0	ESI

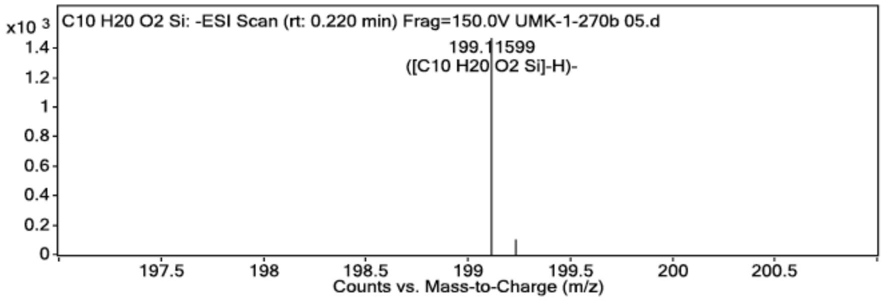

Peak List

m/z	z	Abund	Formula	Ion
56.03085		226.26		
61.98947		188.23		
91.02458		267		
98.95567		254.61		
127.07509		345.49		
187.04228		660.6		
199.11599		1470.14	C10 H20 O2 Si	(M-H)-
233.15462		1253.14		

Figure S85. High resolution mass spectrum of **15c**.

Qualitative Analysis Report

**Data Filename**	UMK-1-274c 01.d	**Sample Name**	sample
**Sample Type**	Sample	**Position**	Vial 1
**Instrument Name**	Instrument 1	**User Name**	
**Acq Method**	without column.m	**Acquired Time**	3/17/2025 4:30:34 PM
**IRM Calibration Status**	Success	**DA Method**	111.m
**Comment**			

**Method part to run:**	Acquisition Only	**Sample Group**	
**Info.**		**Stream Name**	LC 1
**Acquisition SW Version**	6200 series TOF/6500 series Q-TOF B.09.00 (B9044.0)		

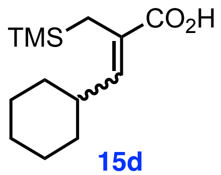

15d

User Chromatograms

Fragmentor Voltage 150 **Collision Energy** 0 **Ionization Mode** APCI

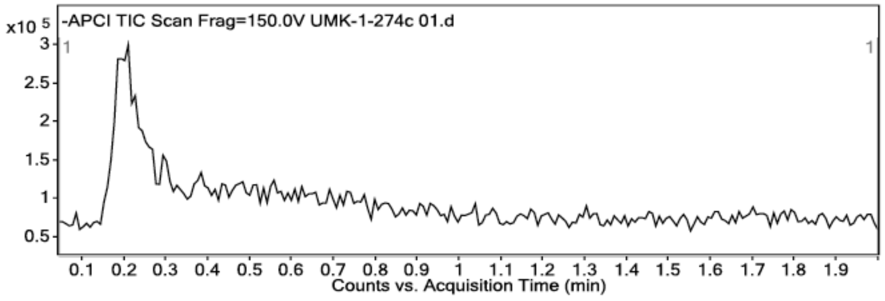

User Spectra

**Fragmentor Voltage**	**Collision Energy**	**Ionization Mode**
150	0	APCI

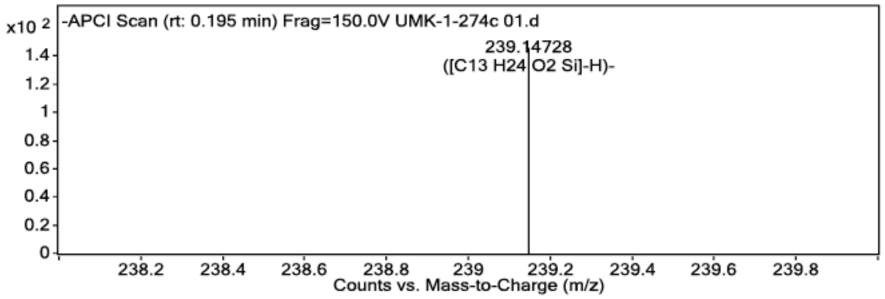

Peak List

m/z	z	Abund	Formula	Ion
61.98661		115.2		
126.94239		235.89		
239.14728		145.65	C13 H24 O2 Si	(M-H)-
248.97121		1645.15		
249.10759		117.18		
279.16377		212.78		
283.10265		188.36		
285.56834	2	192.4		

Agilent Technologies Page 1 of 3 Printed at: 5:43 PM on: 3/17/2025

Figure S86. High resolution mass spectrum of **15d**.

Qualitative Analysis Report

**Data Filename**	UMK-1-154b 01.d	**Sample Name**	Sample
**Sample Type**	Sample	**Position**	Vial 1
**Instrument Name**	Instrument 1	**User Name**	
**Acq Method**	without column.m	**Acquired Time**	10/18/2024 10:40:40 AM
**IRM Calibration Status**	Success	**DA Method**	111.m
**Comment**			

**Method part to run:**	Acquisition Only	**Sample Group**	
**Info.**		**Stream Name**	LC 1
**Acquisition SW Version**	6200 series TOF/6500 series Q-TOF B.09.00 (B9044.0)		

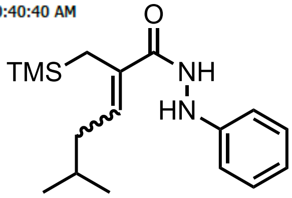

16aa

User Chromatograms

Fragmentor Voltage 150 **Collision Energy** 0 **Ionization Mode** APCI

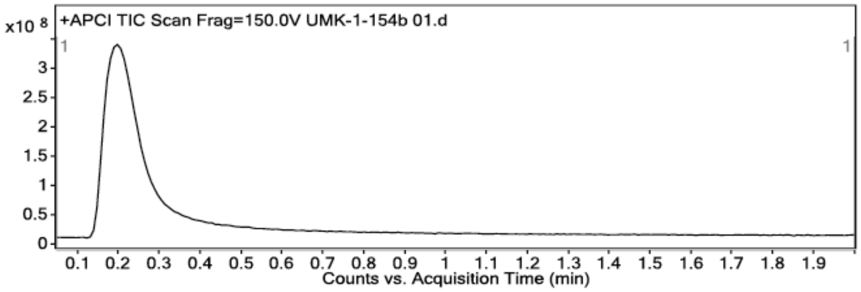

User Spectra

Fragmentor Voltage	Collision Energy	Ionization Mode
150	0	APCI

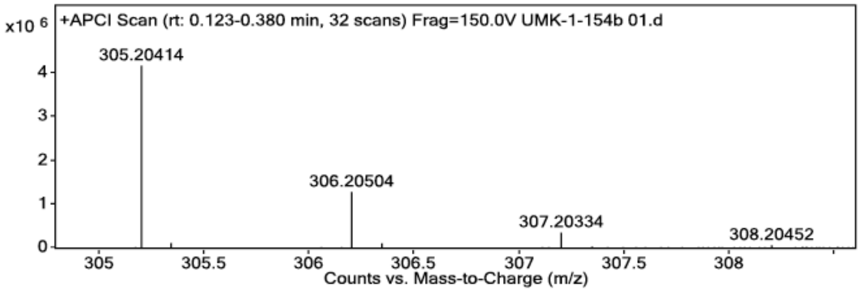

Peak List

m/z	z	Abund
260.18194	1	224731.27
289.17167	1	1170336.13
290.17395	1	285653.78
301.17222	1	384408.53
303.18741	1	2494699.75
303.33125	1	195638.59
304.18964	1	659132.19
305.20414	1	4155185

Figure S87. High resolution mass spectrum of **16aa**.

Qualitative Analysis Report

**Data Filename**	UMK-1-178b 01.d	**Sample Name**	Sample
**Sample Type**	Sample	**Position**	Vial 3
**Instrument Name**	Instrument 1	**User Name**	
**Acq Method**	without column.m	**Acquired Time**	10/18/2024 11:07:36 AM
**IRM Calibration Status**	Success	**DA Method**	111.m
**Comment**			

**Method part to run:**	Acquisition Only	**Sample Group**	
**Info.**		**Stream Name**	LC 1
**Acquisition SW Version**	6200 series TOF/6500 series Q-TOF B.09.00 (B9044.0)		

User Spectra

Fragmentor Voltage	Collision Energy	Ionization Mode
150	0	APCI

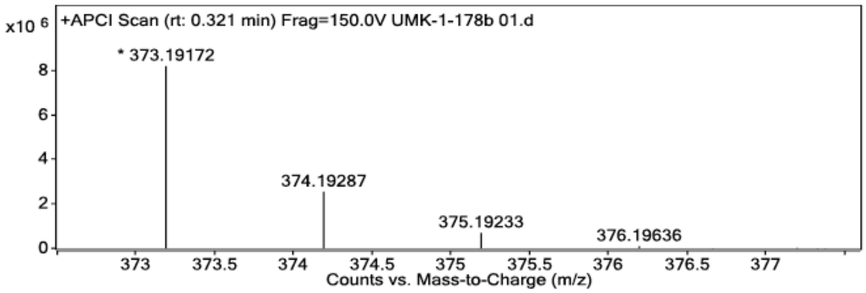

Peak List

m/z	z	Abund
199.11581	1	1210528.88
328.17034	1	1119939.38
357.16046	1	3434882
358.16253	1	908235.31
369.15999	1	1693065.38
371.18013	1	12079976
372.17877	1	5037603
373.19172	1	8189662.5
374.19287	1	2538979.25
443.21452	1	2159214.25

Formula Calculator Element Limits

Element	Min	Max
C	16	17
H	27	28
O	0	1
N	1	2
Si	0	1

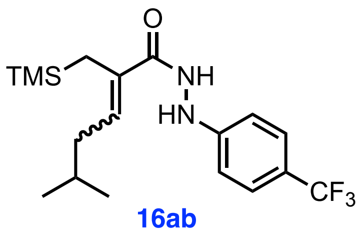

16ab

--- End Of Report ---

Figure S88. High resolution mass spectrum of **16ab**.

Qualitative Analysis Report

**Data Filename**	UMK-1-238b 10.d	**Sample Name**	sample
**Sample Type**	Sample	**Position**	Vial 2
**Instrument Name**	Instrument 1	**User Name**	
**Acq Method**	without column.m	**Acquired Time**	1/10/2025 11:46:19 AM
**IRM Calibration Status**	Success	**DA Method**	111.m
**Comment**			

**Method part to run:**	Acquisition Only	**Sample Group**	
**Info.**		**Stream Name**	LC 1
**Acquisition SW Version**	6200 series TOF/6500 series Q-TOF B.09.00 (B9044.0)		

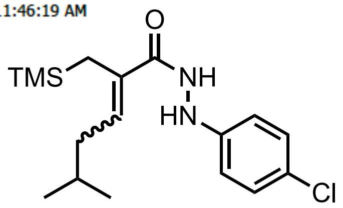

16ac

User Chromatograms

Fragmentor Voltage 120 **Collision Energy** 0 **Ionization Mode** ESI

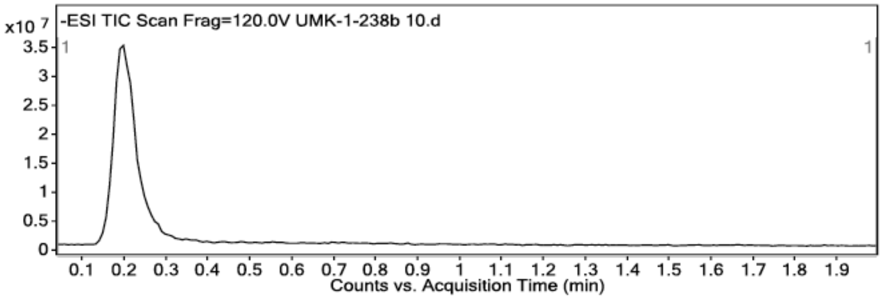

User Spectra

Fragmentor Voltage	Collision Energy	Ionization Mode
120	0	ESI

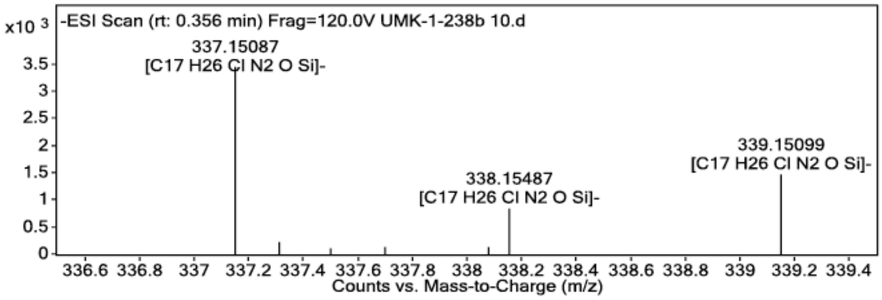

Peak List

m/z	z	Abund	Formula	Ion
61.98953		1449.69		
337.15087	1	3453.74	C17 H26 Cl N2 O Si	M-
339.15099	1	1463.75	C17 H26 Cl N2 O Si	M-
465.15614	1	3944.69		
467.15063	1	2528.28		
528.15232	1	2352.28		
530.14636	1	1439.73		
1282.96616	1	3292.28		

Agilent Technologies Page 1 of 2 Printed at: 12:01 PM on: 1/10/2025

Figure S89. High resolution mass spectrum of **16ac**.

Qualitative Analysis Report

**Data Filename**	UMK-1-244c 01.d	**Sample Name**	sample
**Sample Type**	Sample	**Position**	Vial 1
**Instrument Name**	Instrument 1	**User Name**	
**Acq Method**	without column.m	**Acquired Time**	1/16/2025 3:38:22 PM
**IRM Calibration Status**	Success	**DA Method**	111.m
**Comment**			

**Method part to run:**	Acquisition Only	**Sample Group**	
**Info.**		**Stream Name**	LC 1
**Acquisition SW Version**	6200 series TOF/6500 series Q-TOF B.09.00 (B9044.0)		

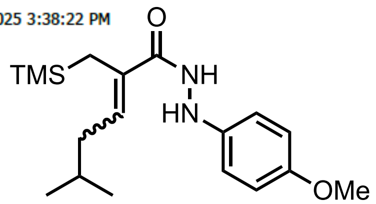

16ad

User Chromatograms

Fragmentor Voltage 150 **Collision Energy** 0 **Ionization Mode** APCI

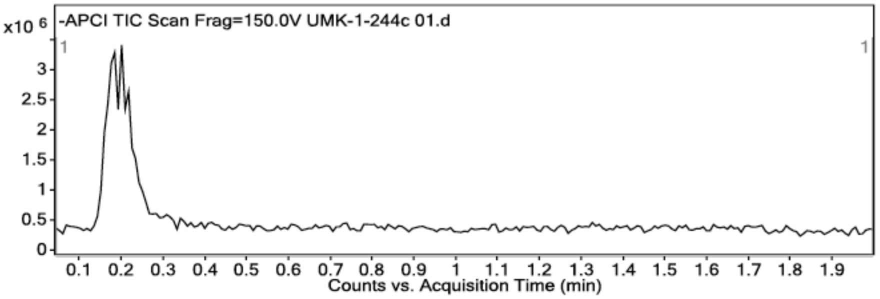

User Spectra

Fragmentor Voltage	Collision Energy	Ionization Mode
150	0	APCI

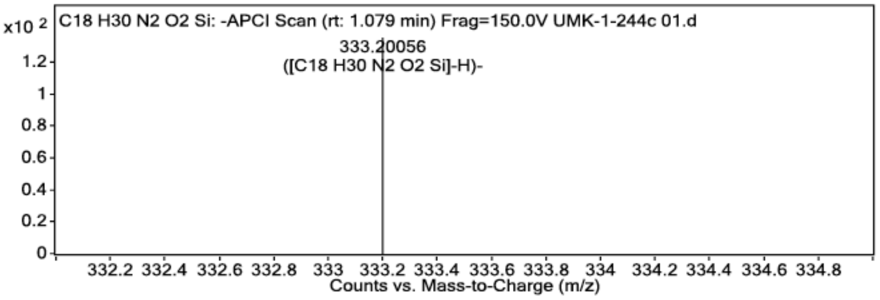

Peak List

m/z	Abund
61.98462	191.66
146.95814	351.14
160.98689	170.11
179.03678	240.84
248.96327	532.76
371.20087	259.75
373.19065	166.06
483.65484	169

Agilent Technologies Page 1 of 2 Printed at: 3:48 PM on: 1/16/2025

Figure S90. High resolution mass spectrum of **16ad**.

Qualitative Analysis Report

**Data Filename**	UMK-1-264b 02.d	**Sample Name**	sample
**Sample Type**	Sample	**Position**	Vial 31
**Instrument Name**	Instrument 1	**User Name**	
**Acq Method**	without column.m	**Acquired Time**	2/26/2025 10:02:17 AM
**IRM Calibration Status**	Success	**DA Method**	111.m
**Comment**			

**Method part to run: Info.**	Acquisition Only	**Sample Group**	
		**Stream Name**	LC 1
**Acquisition SW Version**	6200 series TOF/6500 series Q-TOF B.09.00 (B9044.0)		

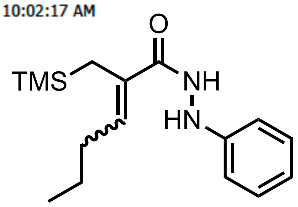

16b

User Chromatograms

Fragmentor Voltage 150 **Collision Energy** 0 **Ionization Mode** ESI

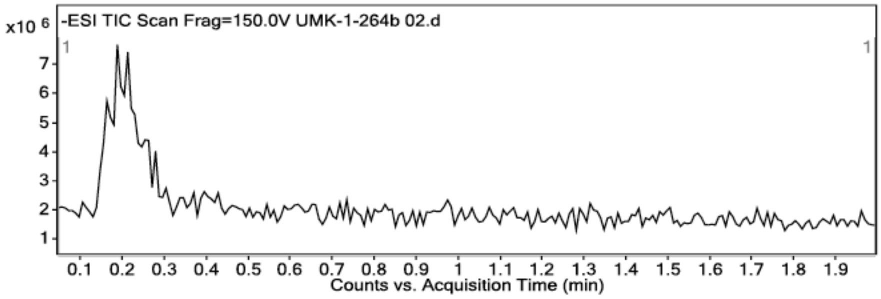

User Spectra

**Fragmentor Voltage**	**Collision Energy**	**Ionization Mode**
150	0	ESI

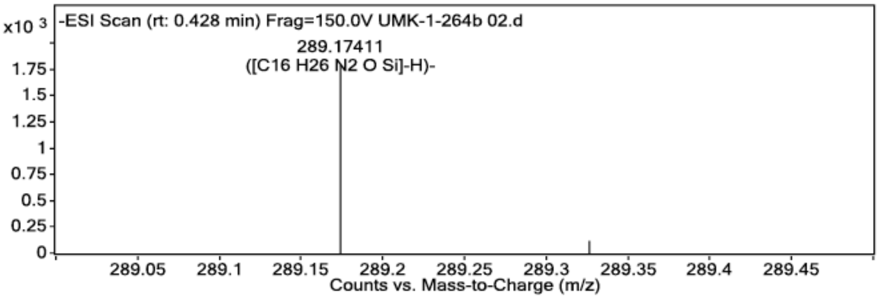

Peak List

m/z	z	Abund	Formula	Ion
109.94052		638.67		
112.98626		362.19	C7 H2 Si	(M-H)-
114.93877		375.08		
135.94547		1586.13		
137.93966		595.28		
139.93161		367.64		
248.97321	1	16204		
249.11473		723.57		

Agilent Technologies   Page 1 of 3   Printed at: 10:13 AM on: 2/26/2025

Figure S91. High resolution mass spectrum of **16b**.

Qualitative Analysis Report

**Data Filename**	UMK-1-272c 06.d	**Sample Name**	sample
**Sample Type**	Sample	**Position**	Vial 5
**Instrument Name**	Instrument 1	**User Name**	
**Acq Method**	without column.m	**Acquired Time**	3/17/2025 4:51:13 PM
**IRM Calibration Status**	Success	**DA Method**	111.m
**Comment**			

**Method part to run:**	Acquisition Only	**Sample Group**	
**Info.**		**Stream Name**	LC 1
**Acquisition SW Version**	6200 series TOF/6500 series Q-TOF B.09.00 (B9044.0)		

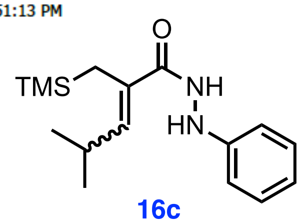

16c

User Chromatograms

Fragmentor Voltage 150 **Collision Energy** 0 **Ionization Mode** ESI

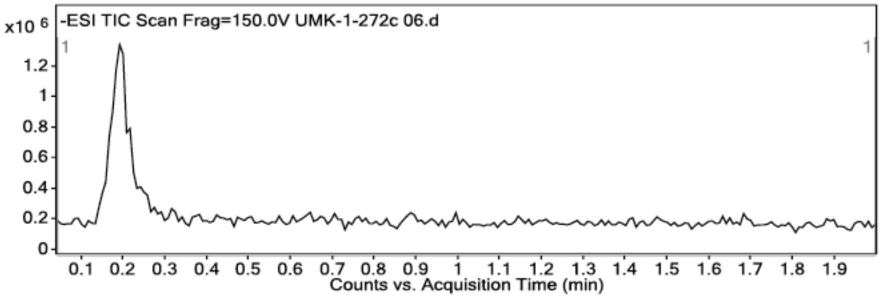

User Spectra

Fragmentor Voltage	Collision Energy	Ionization Mode
150	0	ESI

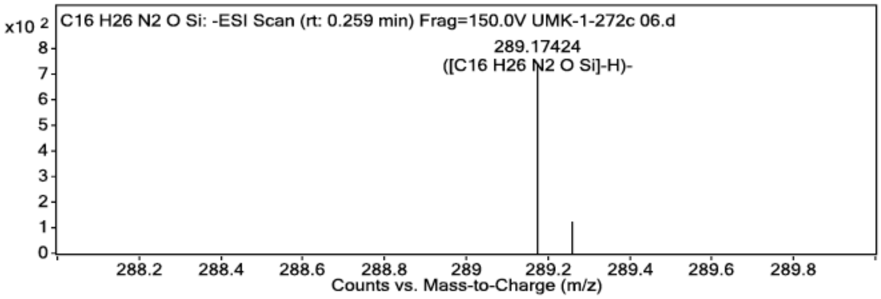

Peak List

m/z	z	Abund	Formula	Ion
60.98635		139.01		
61.98471		176.86		
95.02815		157.42		
116.29712		180.9		
122.02959		184.09		
126.00767		115.66		
170.04323		149.06		
229.85633		117.41		

Agilent Technologies Page 1 of 3 Printed at: 5:02 PM on: 3/17/2025

Figure S92. High resolution mass spectrum of **16c**.

Qualitative Analysis Report

**Data Filename**	UMK-1-278b 02.d	**Sample Name**	sample
**Sample Type**	Sample	**Position**	Vial 2
**Instrument Name**	Instrument 1	**User Name**	
**Acq Method**	without column.m	**Acquired Time**	3/17/2025 4:33:51 PM
**IRM Calibration Status**	Success	**DA Method**	111.m
**Comment**			

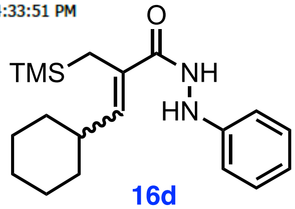

16d

**Method part to run:**	Acquisition Only	**Sample Group**	
**Info.**		**Stream Name**	LC 1
**Acquisition SW Version**	6200 series TOF/6500 series Q-TOF B.09.00 (B9044.0)		

User Chromatograms

Fragmentor Voltage 150 **Collision Energy** 0 **Ionization Mode** APCI

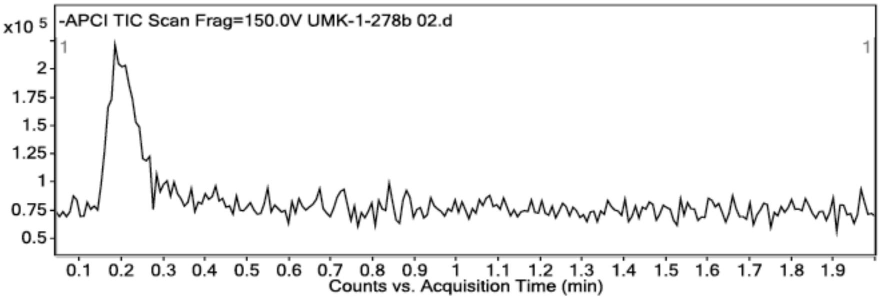

User Spectra

Fragmentor Voltage	Collision Energy	Ionization Mode
150	0	APCI

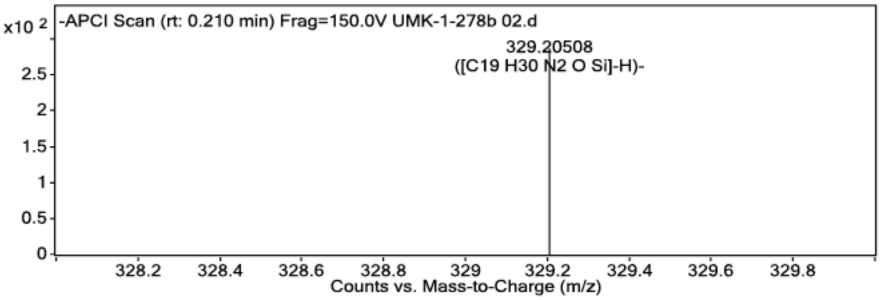

Peak List

m/z	z	Abund	Formula	Ion
184.93489		115.01		
211.14795		119.61		
248.97555		1749.19		
301.10249		114.88		
311.17032		232.38		
329.20508		286.92	C19 H30 N2 O Si	(M-H)-
330.21344		281.41		
354.88554		112.69		

Agilent Technologies Page 1 of 2 Printed at: 5:46 PM on: 3/17/2025

Figure S93. High resolution mass spectrum of **16d**.

Qualitative Analysis Report

**Data Filename**	UMK-1-220c 04.d	**Sample Name**	Sample
**Sample Type**	Sample	**Position**	Vial 1
**Instrument Name**	Instrument 1	**User Name**	
**Acq Method**	without column.m	**Acquired Time**	12/19/2024 4:33:07 PM
**IRM Calibration Status**	Success	**DA Method**	111.m
**Comment**			

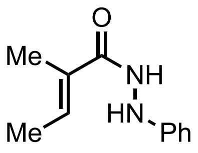

18

**Method part to run:**	Acquisition Only	**Sample Group**	
**Info.**		**Stream Name**	LC 1
**Acquisition SW Version**	6200 series TOF/6500 series Q-TOF B.09.00 (B9044.0)		

User Chromatograms

Fragmentor Voltage 120 **Collision Energy** 0 **Ionization Mode** APCI

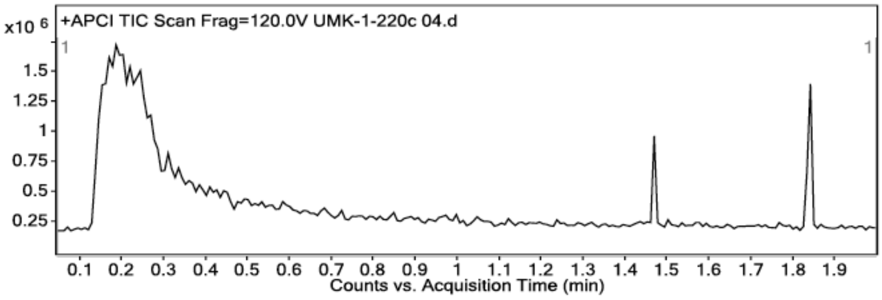

User Spectra

Fragmentor Voltage	Collision Energy	Ionization Mode
120	0	APCI

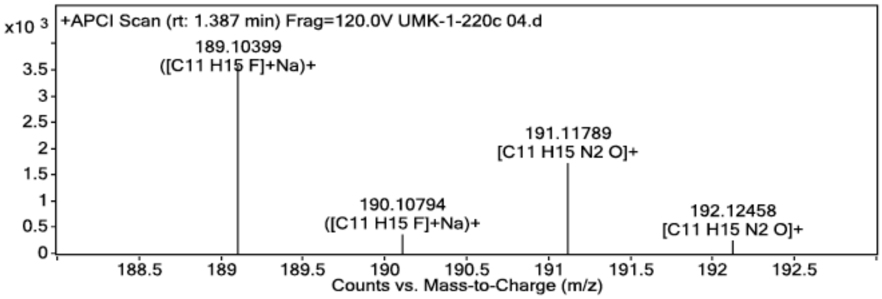

Peak List

m/z	z	Abund	Formula	Ion
189.10399	1	3577.88	C11 H15 F	(M+Na)+
190.10794	1	361.78	C11 H15 F	(M+Na)+
191.11789	1	1721.3	C11 H15 N2 O	M+
192.12458	1	242.9	C11 H15 N2 O	M+
225.19657		541.26	C13 H25 N2 O	M+
284.12088	1	1018.2	C18 H11 O	(M+C3H5)+
285.12089	1	237.49	C18 H11 O	(M+C3H5)+
371.22042		244.01		

Agilent Technologies   Page 1 of 3   Printed at: 4:46 PM on: 12/19/2024

Figure S94. High resolution mass spectrum of **18**.

Qualitative Analysis Report

**Data Filename**	UMK-I-294b.d	**Sample Name**	UMK-I-294b
**Sample Type**	Sample	**Position**	P1-A1
**Instrument Name**	6530B LC Q-TOF	**User Name**	Hasan CAN (hcan)
**Acq Method**	ESI_Pos.m	**Acquired Time**	8/20/2025 5:52:04 PM (UTC+03:00)
**IRM Calibration Status**	Success	**DA Method**	hcan.m
**Comment**			

**Sample Group**		**Info.**	
**Stream Name**	LC 1	**Method Version**	2025-0326-1400-23939
**Override DA Method Version**		**Data File Version**	2025-0820-1452-00578
**Acquisition Workstation**	DESKTOP-L73MD3C	**DA Workstation**	DESKTOP-L73MD3C
**Acquisition Time (Local)**	8/20/2025 5:52:04 PM (UTC+03:00)	**Acquisition SW Version**	6200 series TOF/6500 series Q-TOF (11.0.203.0)
**QTOF Driver Version**	11.00.00	**QTOF Firmware Version**	15.851
**Tune Mass Range Max.**	3200		

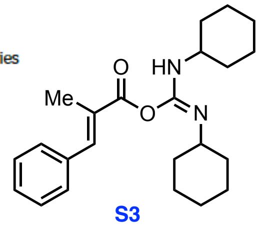

S3

Chromatograms

Fragmentor Voltage 90 **Collision Energy** 0 **Ionization Mode** ESI

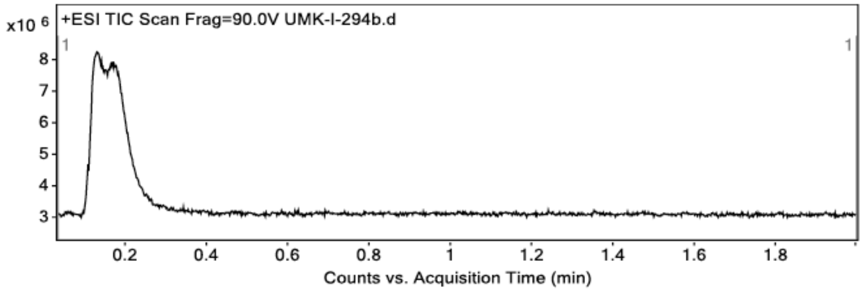

Spectra

Fragmentor Voltage	Collision Energy	Ionization Mode
90	0	ESI

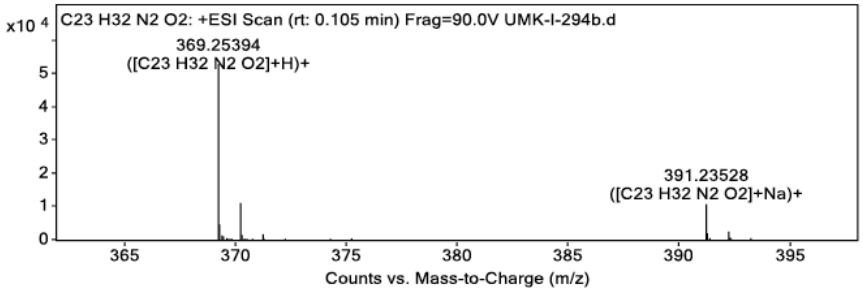

Peak List

m/z	z	Abund	Formula	Ion

Figure S95. High resolution mass spectrum of **S3**.

Qualitative Analysis Report

**Data Filename**	UMK-1-172b 01.d	**Sample Name**	Sample
**Sample Type**	Sample	**Position**	Vial 2
**Instrument Name**	Instrument 1	**User Name**	
**Acq Method**	without column.m	**Acquired Time**	10/18/2024 10:54:03 AM
**IRM Calibration Status**	Success	**DA Method**	111.m
**Comment**			

**Method part to run:**	Acquisition Only	**Sample Group**	
**Info.**		**Stream Name**	LC 1
**Acquisition SW Version**	6200 series TOF/6500 series Q-TOF B.09.00 (B9044.0)		

User Spectra

Fragmentor Voltage	Collision Energy	Ionization Mode
150	0	APCI

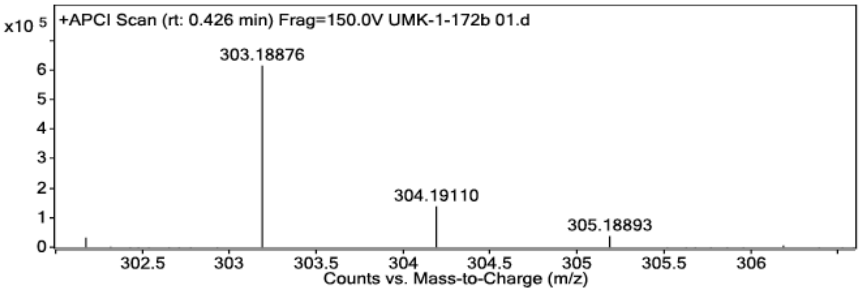

Peak List

m/z	z	Abund
223.09802	1	64304.84
301.17323	1	135772.55
303.18876	1	615285.81
304.1911	1	138266.59
391.23031	1	245355.11
392.23234	1	75384.16
515.30905	1	49262.58
531.30528		120675.39
553.28739	1	64767.58
925.49764	1	55214.96

Formula Calculator Element Limits

Element	Min	Max
C	16	17
H	27	28
O	0	1
N	1	2
Si	0	1

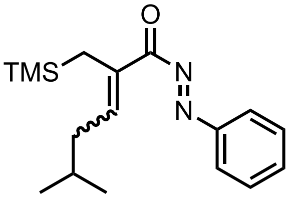

10aa

--- End Of Report ---

Agilent Technologies   Page 1 of 1   Printed at: 2:20 PM on: 10/18/2024

Figure S96. High resolution mass spectrum of **10aa**.

Qualitative Analysis Report

**Data Filename**	UMK-1-180bt 03.d	**Sample Name**	Sample
**Sample Type**	Sample	**Position**	Vial 1
**Instrument Name**	Instrument 1	**User Name**	
**Acq Method**	without column.m	**Acquired Time**	12/10/2024 4:43:10 PM
**IRM Calibration Status**	Success	**DA Method**	111.m
**Comment**			

**Method part to run:**	Acquisition Only	**Sample Group**	
**Info.**		**Stream Name**	LC 1
**Acquisition SW Version**	6200 series TOF/6500 series Q-TOF B.09.00 (B9044.0)		

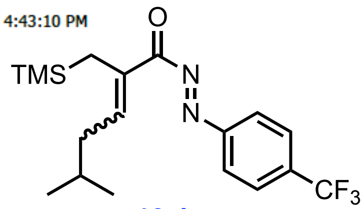

10ab

User Chromatograms

Fragmentor Voltage 120 **Collision Energy** 0 **Ionization Mode** APCI

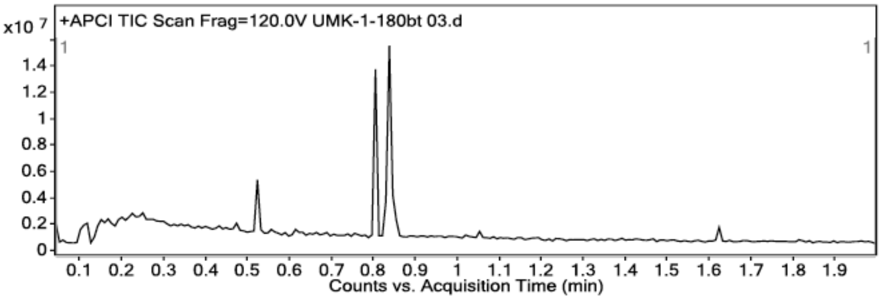

User Spectra

Fragmentor Voltage	Collision Energy	Ionization Mode
120	0	APCI

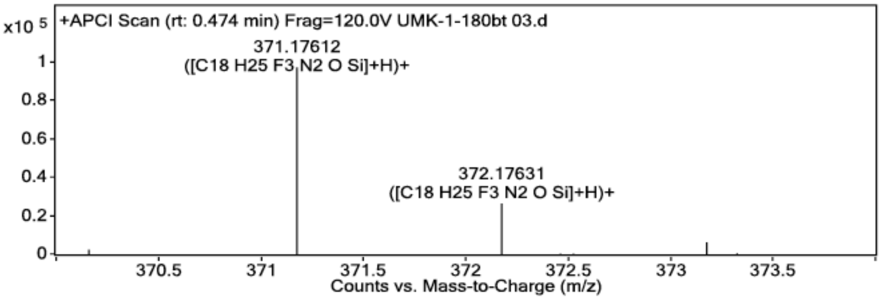

Peak List

m/z	z	Abund	Formula	Ion
253.16698	1	3781.58	C15 H22 N2	(M+Na)+
284.11695	1	3102.52	C13 H20 F3 Si	(M+Na)+
369.16012	1	9221.42	C18 H24 F3 N2 O Si	M+
370.15969	1	2253.38	C18 H24 F3 N2 O Si	M+
371.17612	1	97047.23	C18 H25 F3 N2 O Si	(M+H)+
372.17631	1	26189.76	C18 H25 F3 N2 O Si	(M+H)+
373.17612	1	6026.12	C18 H25 F3 N2 O Si	(M+H)+
383.15151	1	3675.49	C18 H14 F2 N O Si	(M+C4H9)+

Agilent Technologies Page 1 of 7 Printed at: 5:00 PM on: 12/10/2024

Figure S97. High resolution mass spectrum of **10ab**.

Qualitative Analysis Report

**Data Filename**	UMK-1-240b 16.d	**Sample Name**	sample
**Sample Type**	Sample	**Position**	Vial 2
**Instrument Name**	Instrument 1	**User Name**	
**Acq Method**	without column.m	**Acquired Time**	1/10/2025 2:32:15 PM
**IRM Calibration Status**	Success	**DA Method**	111.m
**Comment**			

**Method part to run:**	Acquisition Only	**Sample Group**	
**Info.**		**Stream Name**	LC 1
**Acquisition SW Version**	6200 series TOF/6500 series Q-TOF B.09.00 (B9044.0)		

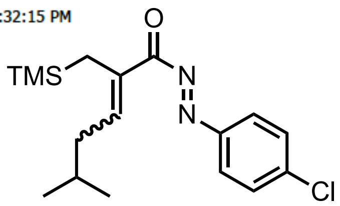

10ac

User Chromatograms

Fragmentor Voltage 150 **Collision Energy** 0 **Ionization Mode** APCI

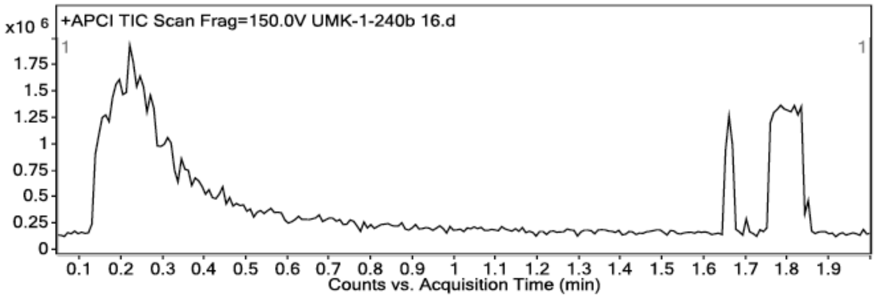

User Spectra

Fragmentor Voltage 150 **Collision Energy** 0 **Ionization Mode** APCI

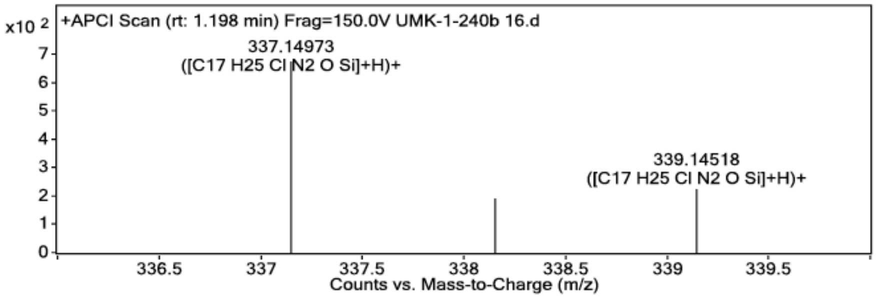

Peak List

m/z	z	Abund	Formula	Ion
284.11623		281.56		
288.2893		284.46		
317.20053		136.08		
337.14973	1	675.24	C17 H25 Cl N2 O Si	(M+H)+
338.15437	1	190.43	C17 H25 Cl N2 O Si	(M+H)+
339.14518	1	223.59	C17 H25 Cl N2 O Si	(M+H)+
340.13977		136.34		
385.28523		102.21		

Agilent Technologies Page 1 of 2 Printed at: 2:46 PM on: 1/10/2025

Figure S98. High resolution mass spectrum of **10ac**.

Qualitative Analysis Report

**Data Filename**	UMK-1-248b 01.d	**Sample Name**	sample
**Sample Type**	Sample	**Position**	Vial 1
**Instrument Name**	Instrument 1	**User Name**	
**Acq Method**	without column.m	**Acquired Time**	1/28/2025 3:30:12 PM
**IRM Calibration Status**	Success	**DA Method**	111.m
**Comment**			

**Method part to run:**	Acquisition Only	**Sample Group**	
**Info.**		**Stream Name**	LC 1
**Acquisition SW Version**	6200 series TOF/6500 series Q-TOF B.09.00 (B9044.0)		

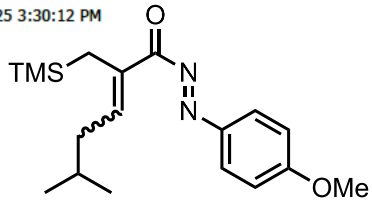

10ad

User Chromatograms

Fragmentor Voltage 120 **Collision Energy** 0 **Ionization Mode** APCI

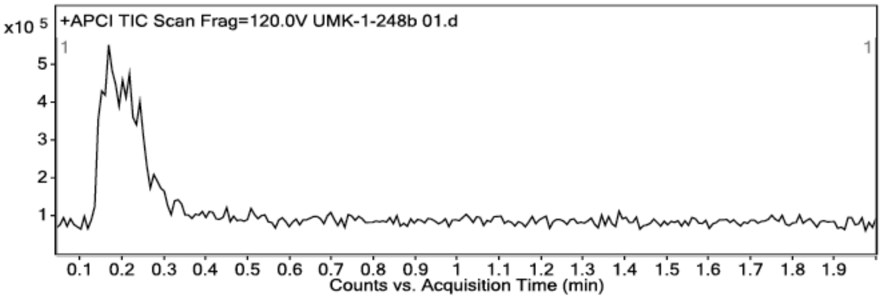

User Spectra

Fragmentor Voltage 120 **Collision Energy** 0 **Ionization Mode** APCI

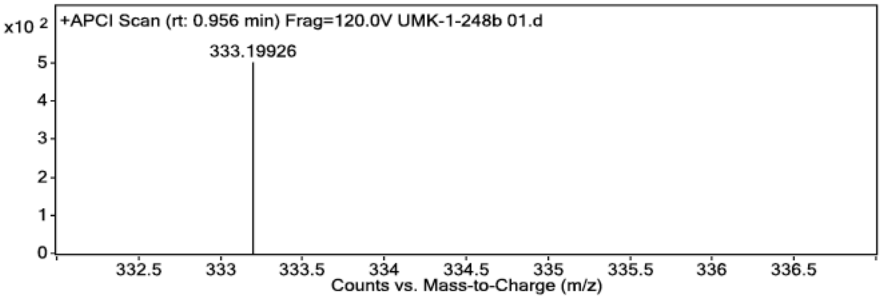

Peak List

m/z	Abund	Formula	Ion
333.19926	501.28	C18 H28 N2 O2 Si	(M+H)+
357.15548	117.9		
454.37325	138.09		
485.29698	285.68		

Formula Calculator Element Limits

Element	Min	Max
C	0	18
H	0	28
N	0	2

Agilent Technologies Page 1 of 2 Printed at: 3:39 PM on: 1/28/2025

Figure S99. High resolution mass spectrum of **10ad**.

Qualitative Analysis Report

**Data Filename**	UMK-1-266b 04.d	**Sample Name**	sample
**Sample Type**	Sample	**Position**	Vial 32
**Instrument Name**	Instrument 1	**User Name**	
**Acq Method**	without column.m	**Acquired Time**	2/26/2025 10:14:34 AM
**IRM Calibration Status**	Success	**DA Method**	111.m
**Comment**			

**Method part to run:**	Acquisition Only	**Sample Group**	
**Info.**		**Stream Name**	LC 1
**Acquisition SW Version**	6200 series TOF/6500 series Q-TOF B.09.00 (B9044.0)		

User Spectra

Fragmentor Voltage	Collision Energy	Ionization Mode
150	0	ESI

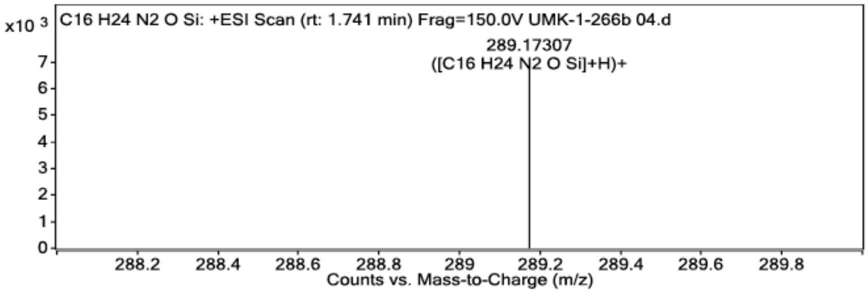

Peak List

m/z	z	Abund	Formula	Ion
122.06146		232.69		
123.07287		130.45		
137.10127		101.29		
138.10093		172.37		
152.12169	1	1094.9		
153.12069	1	154.43		
154.13309		220.35		
168.12979		100.25		
174.11402		547.14		
175.11024		231.1		
180.12848		223.88		
192.12486	1	1234.81		
193.13702	1	136.57		
195.13174		254.21		
197.15297		626.4		
201.09872		189.78		
217.12748		243.63		
229.12184		105.46		
229.1813		434.46		
266.14053		129.9		
271.19171		194.84		
273.1418		1458.93		

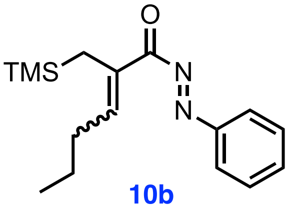

10b

Agilent Technologies Page 1 of 2 Printed at: 10:27 AM on: 2/26/2025

Figure S100. High resolution mass spectrum of **10b**.

Qualitative Analysis Report

**Data Filename**	UMK-1-276b 10.d	**Sample Name**	sample
**Sample Type**	Sample	**Position**	Vial 6
**Instrument Name**	Instrument 1	**User Name**	
**Acq Method**	without column.m	**Acquired Time**	3/17/2025 5:08:00 PM
**IRM Calibration Status**	Success	**DA Method**	111.m
**Comment**			

**Method part to run:**	Acquisition Only	**Sample Group**	
**Info.**		**Stream Name**	LC 1
**Acquisition SW Version**	6200 series TOF/6500 series Q-TOF B.09.00 (B9044.0)		

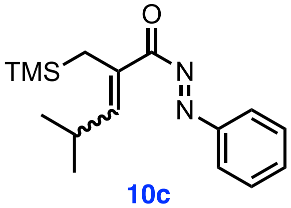

10c

User Spectra

Fragmentor Voltage	Collision Energy	Ionization Mode
150	0	APCI

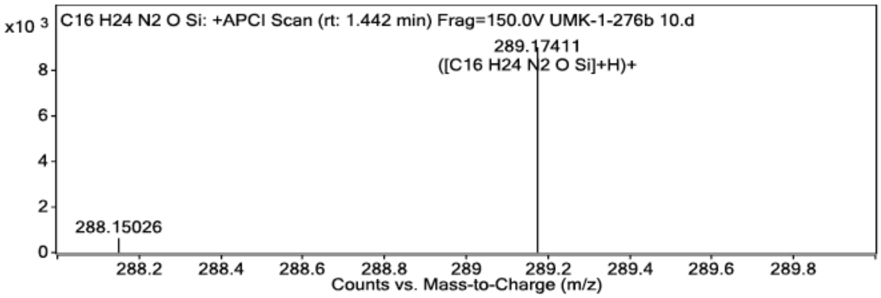

Peak List

m/z	z	Abund	Formula	Ion
120.08349		1499.97		
121.07057		222.8		
122.07303		435.55		
134.07001		212.05		
135.07015		266.05		
136.07701		289.92		
137.08369		194.06		
137.13191		415.33		
138.13069		265.23		
148.08644		505.83		
149.09012		286		
152.144		875.65		
153.1405		253.03		
156.07138		253.22		
157.07748		811.61		
163.09705		209.06		
171.09031		574.17		
174.09367		203.13		
175.07066		744.05		
177.1143		212.37		
180.13723		510.56		
185.09953		464.61		

Agilent Technologies   Page 1 of 3   Printed at: 5:19 PM on: 3/17/2025

Figure S101. High resolution mass spectrum of **10c**.

Qualitative Analysis Report

**Data Filename**	UMK-1-284b 09.d	**Sample Name**	sample
**Sample Type**	Sample	**Position**	Vial 3
**Instrument Name**	Instrument 1	**User Name**	
**Acq Method**	without column.m	**Acquired Time**	3/17/2025 5:03:54 PM
**IRM Calibration Status**	Success	**DA Method**	111.m
**Comment**			

**Method part to run:**	Acquisition Only	**Sample Group**	
**Info.**		**Stream Name**	LC 1
**Acquisition SW Version**	6200 series TOF/6500 series Q-TOF B.09.00 (B9044.0)		

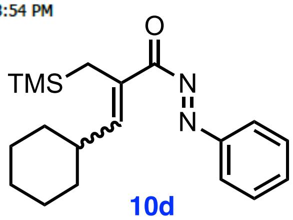

10d

User Chromatograms

Fragmentor Voltage 150 **Collision Energy** 0 **Ionization Mode** APCI

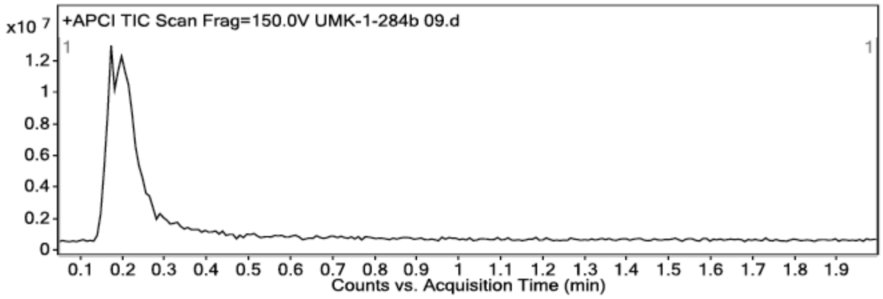

User Spectra

Fragmentor Voltage	Collision Energy	Ionization Mode
150	0	APCI

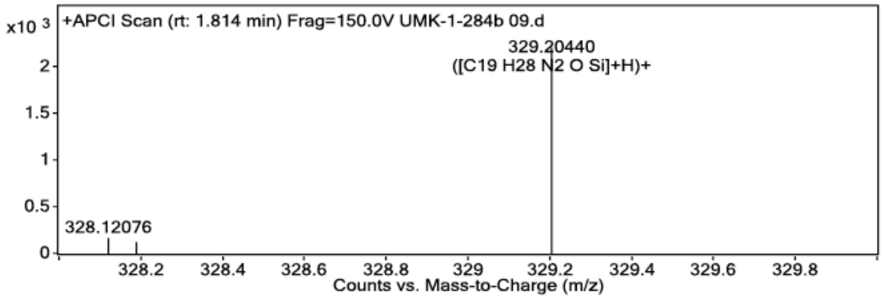

Peak List

m/z	z	Abund	Formula	Ion
120.08307		1085.79		
121.08965		234		
122.07163		342.56		
123.09846		533.83		
132.057		180.26		
135.07923		146.14		
136.07839		186.45		
137.13213		327.58		

Agilent Technologies Page 1 of 3 Printed at: 5:51 PM on: 3/17/2025

Figure S102. High resolution mass spectrum of **10d**.

Qualitative Analysis Report

**Data Filename**	UMK-II-36b.d	**Sample Name**	UMK-II-36b
**Sample Type**	Sample	**Position**	P1-A4
**Instrument Name**	6530B LC Q-TOF	**User Name**	Hasan CAN (hcan)
**Acq Method**	ESI_Pos.m	**Acquired Time**	8/20/2025 6:00:20 PM (UTC+03:00)
**IRM Calibration Status**	Success	**DA Method**	hcan.m
**Comment**			

**Sample Group**		**Info.**	
**Stream Name**	LC 1	**Method Version**	2025-0326-1400-23939
**Override DA Method Version**		**Data File Version**	2025-0820-1500-14984
**Acquisition Workstation**	DESKTOP-L73MD3C	**DA Workstation**	DESKTOP-L73MD3C
**Acquisition Time (Local)**	8/20/2025 6:00:20 PM (UTC+03:00)	**Acquisition SW Version**	6200 series TOF/6500 series Q-TOF (11.0.203.0)
**QTOF Driver Version**	11.00.00	**QTOF Firmware Version**	15.851
**Tune Mass Range Max.**	3200		

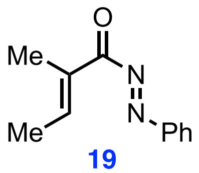

19

Chromatograms

Fragmentor Voltage 90 **Collision Energy** 0 **Ionization Mode** ESI

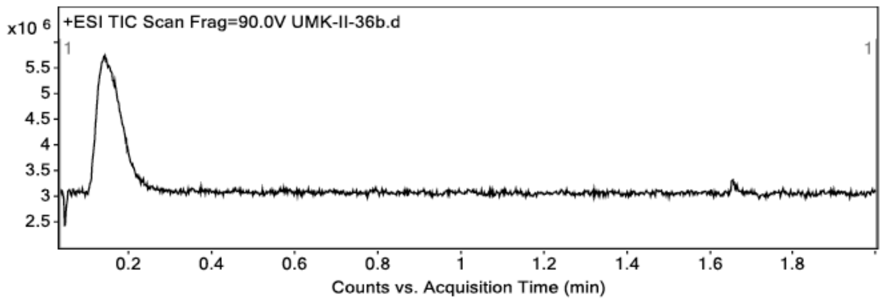

Spectra

Fragmentor Voltage	Collision Energy	Ionization Mode
90	0	ESI

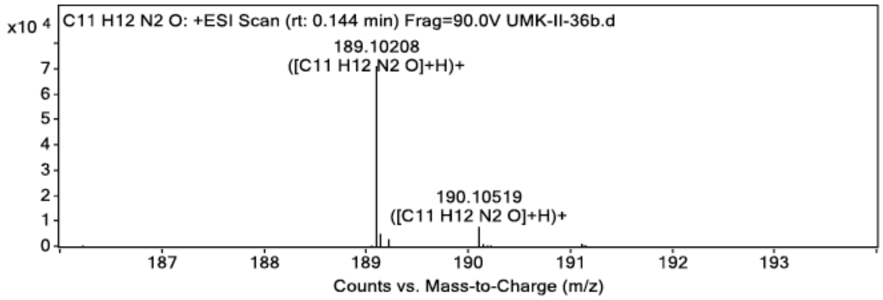

Peak List

m/z	z	Abund	Formula	Ion

Figure S103. High resolution mass spectrum of **19**.

Qualitative Analysis Report

**Data Filename**	UMK-1-164b 11.d	**Sample Name**	UMK-1-164b
**Sample Type**	Sample	**Position**	Vial 1
**Instrument Name**	Instrument 1	**User Name**	
**Acq Method**	without column.m	**Acquired Time**	10/15/2024 4:44:53 PM
**IRM Calibration Status**	Success	**DA Method**	111.m
**Comment**			

**Method part to run:**	Acquisition Only	**Sample Group**	
**Info.**		**Stream Name**	LC 1
**Acquisition SW Version**	6200 series TOF/6500 series Q-TOF B.09.00 (B9044.0)		

User Spectra

Fragmentor Voltage	Collision Energy	Ionization Mode
120	0	APCI

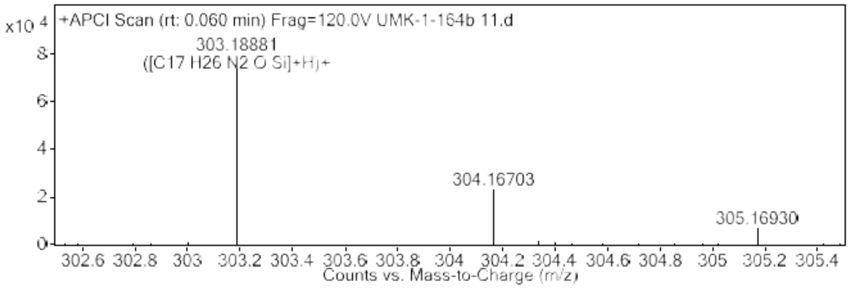

Peak List

m/z	z	Abund	Formula	Ion
293.13863	1	73386.91		
303.18881		75481.84	C17 H26 N2 O Si	(M+H)+
350.15053	1	61134.86		
396.18038	1	247482		
397.18376	1	63406.05		
410.19582	1	80447.05		
424.21084	1	128718.92		
426.19155	1	113844.23		
1221.98767	1	146288.44		
1521.96925	1	97260.91		

Formula Calculator Element Limits

Element	Min	Max
C	16	17
H	25	26
O	1	1
N	1	2
Si	0	1

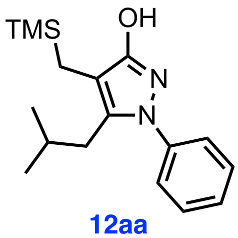

12aa

Formula Calculator Results

Formula	Best	Mass	Tgt Mass	Diff (ppm)	Ion Species	Score
C17 H26 N2 O Si	TRUE	302.1815	302.1814	-0.31	C17 H27 N2 O Si	47.59

--- End Of Report ---

Agilent Technologies Page 1 of 1 Printed at: 4:55 PM on: 10/15/2024

Figure S104. High resolution mass spectrum of **12aa**.

Qualitative Analysis Report

**Data Filename**	UMK-1-224c 02.d	**Sample Name**	Sample
**Sample Type**	Sample	**Position**	Vial 2
**Instrument Name**	Instrument 1	**User Name**	
**Acq Method**	without column.m	**Acquired Time**	12/19/2024 3:56:23 PM
**IRM Calibration Status**	Success	**DA Method**	111.m
**Comment**			

**Method part to run:**	Acquisition Only	**Sample Group**	
**Info.**		**Stream Name**	LC 1
**Acquisition SW Version**	6200 series TOF/6500 series Q-TOF B.09.00 (B9044.0)		

User Chromatograms

Fragmentor Voltage 120 **Collision Energy** 0 **Ionization Mode** APCI

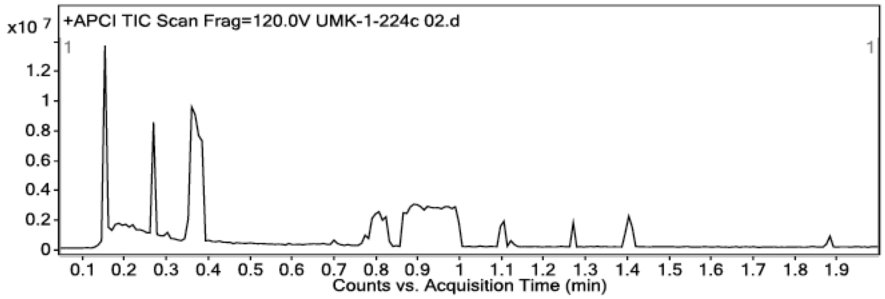

User Spectra

Fragmentor Voltage	Collision Energy	Ionization Mode
120	0	APCI

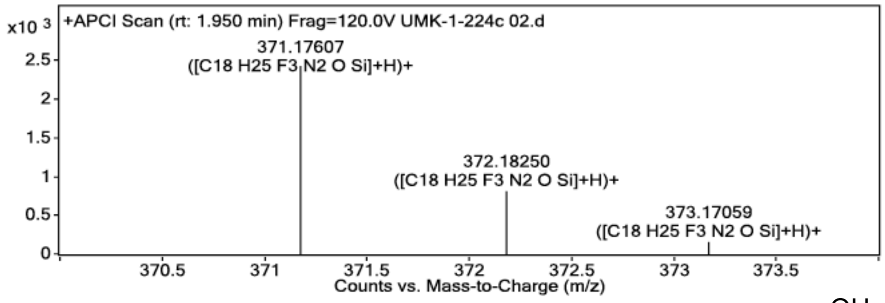

Peak List

m/z	z	Abund	Formula	Ion
189.09324	1	766.01	C9 H16 N Si	(M+Na)+
191.11051		451.14	C12 H14 F N	M+
284.11925	1	1436.92	C16 H16 F3	(M+F)+
294.9461		258.1	C13 H F3 N Si	(M+K)+
371.17607	1	2428.25	C18 H25 F3 N2 O Si	(M+H)+
372.1825	1	809.72	C18 H25 F3 N2 O Si	(M+H)+
385.22768		420.23	C16 H23 F3 N2 Si	(M+C4H9)+
485.29067	1	1638.16		

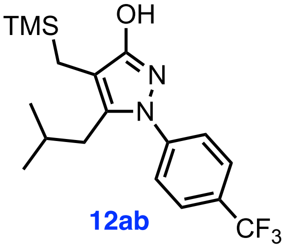

Figure S105. High resolution mass spectrum of **12ab**.

Qualitative Analysis Report

**Data Filename**	UMK-1-242b 11.d	**Sample Name**	sample
**Sample Type**	Sample	**Position**	Vial 4
**Instrument Name**	Instrument 1	**User Name**	
**Acq Method**	without column.m	**Acquired Time**	1/10/2025 12:05:54 PM
**IRM Calibration Status**	Success	**DA Method**	111.m
**Comment**			

**Method part to run:**	Acquisition Only	**Sample Group**	
**Info.**		**Stream Name**	LC 1
**Acquisition SW Version**	6200 series TOF/6500 series Q-TOF B.09.00 (B9044.0)		

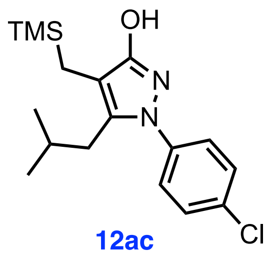

12ac

User Chromatograms

Fragmentor Voltage 120 **Collision Energy** 0 **Ionization Mode** ESI

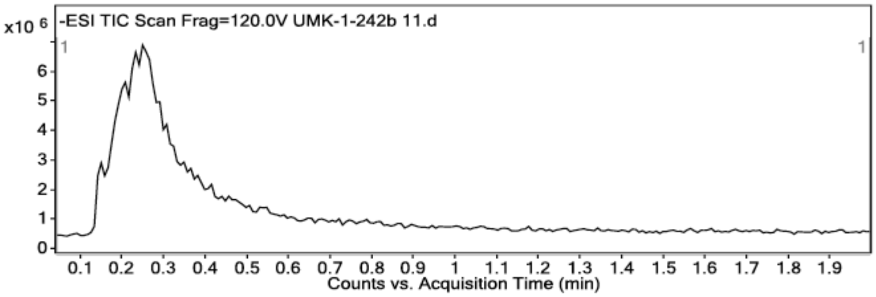

User Spectra

Fragmentor Voltage	Collision Energy	Ionization Mode
120	0	ESI

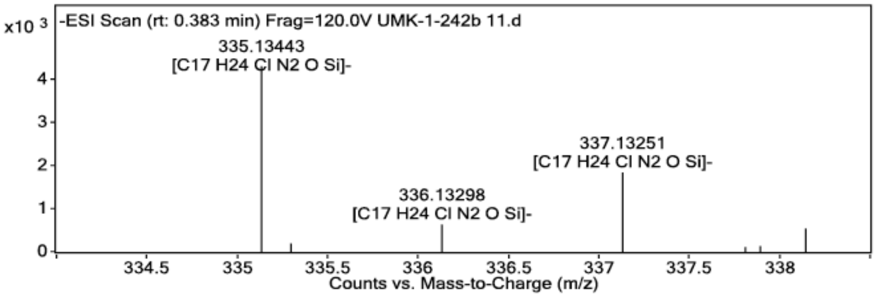

Peak List

m/z	z	Abund	Formula	Ion
61.98804		1489.78		
112.98583		932.65	C7 H Si	M-
248.96903		1141.85	C13 H2 Cl N2 Si	M-
311.16847		857.71		
325.18048		910.12		
335.13443	1	4293.53	C17 H24 Cl N2 O Si	M-
337.13251	1	1826.88	C17 H24 Cl N2 O Si	M-
560.86764		1243.67		

Agilent Technologies Page 1 of 2 Printed at: 12:24 PM on: 1/10/2025

Figure S106. High resolution mass spectrum of **12ac**.

Qualitative Analysis Report

**Data Filename**	UMK-1-250b 04.d	**Sample Name**	sample
**Sample Type**	Sample	**Position**	Vial 1
**Instrument Name**	Instrument 1	**User Name**	
**Acq Method**	without column.m	**Acquired Time**	2/12/2025 10:37:59 AM
**IRM Calibration Status**	Success	**DA Method**	111.m
**Comment**			

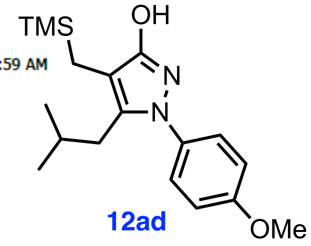

12ad

**Method part to run:**	Acquisition Only	**Sample Group**	
**Info.**		**Stream Name**	LC 1
**Acquisition SW Version**	6200 series TOF/6500 series Q-TOF B.09.00 (B9044.0)		

User Chromatograms

Fragmentor Voltage 120 **Collision Energy** 0 **Ionization Mode** APCI

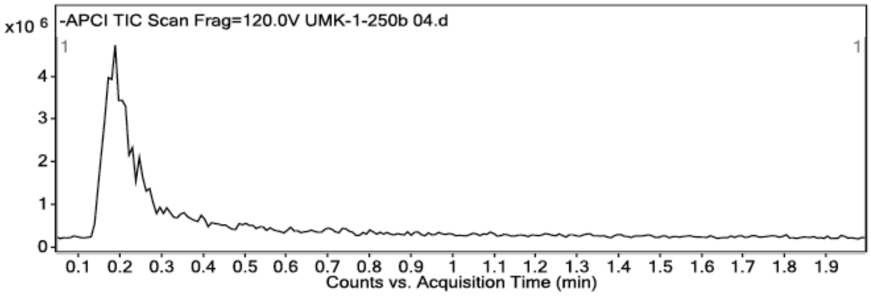

User Spectra

Fragmentor Voltage	Collision Energy	Ionization Mode
120	0	APCI

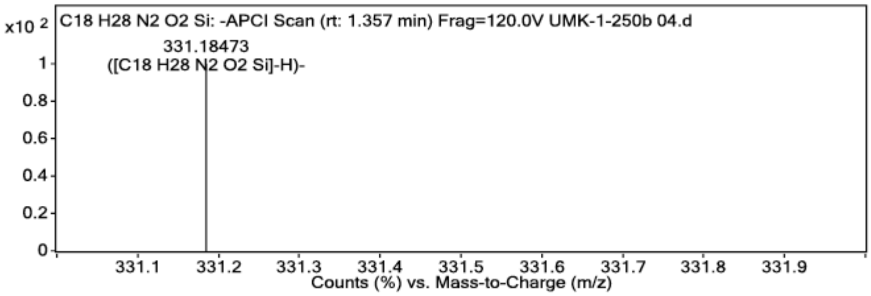

Peak List

m/z	Abund	Formula	Ion
109.04566	148		
126.90659	300.07		
132.05923	142.47		
157.05064	127.05		
161.0723	144.3		
162.02399	185.67		
165.05353	126.36		
184.01986	113.14		

Agilent Technologies Page 1 of 3 Printed at: 10:46 AM on: 2/12/2025

Figure S107. High resolution mass spectrum of **12ad**.

Qualitative Analysis Report

**Data Filename**	UMK-1-268b 07.d	**Sample Name**	sample
**Sample Type**	Sample	**Position**	Vial 33
**Instrument Name**	Instrument 1	**User Name**	
**Acq Method**	without column.m	**Acquired Time**	2/26/2025 10:27:56 AM
**IRM Calibration Status**	Success	**DA Method**	111.m
**Comment**			

**Method part to run:**	Acquisition Only	**Sample Group**	
**Info.**		**Stream Name**	LC 1
**Acquisition SW Version**	6200 series TOF/6500 series Q-TOF B.09.00 (B9044.0)		

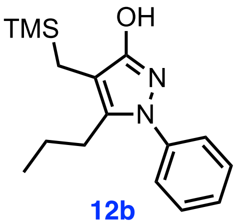

12b

User Chromatograms

Fragmentor Voltage 150 **Collision Energy** 0 **Ionization Mode** ESI

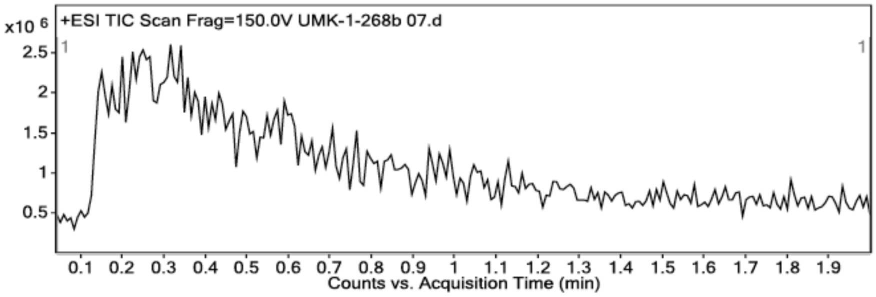

User Spectra

Fragmentor Voltage	Collision Energy	Ionization Mode
150	0	ESI

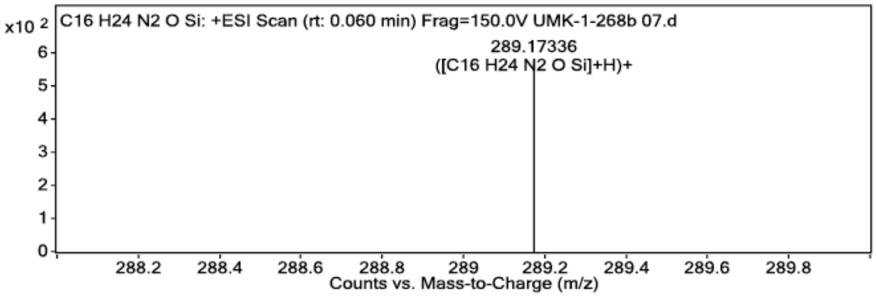

Peak List

m/z	z	Abund	Formula	Ion
122.06147		323.01		
123.07084		177.66		
136.05274		127.72		
137.10756		266.2		
138.09788		221.2		
152.12169	1	1427.36		
153.12367	1	173.51		
154.13586		316.69		

Agilent Technologies Page 1 of 3 Printed at: 10:34 AM on: 2/26/2025

Figure S108. High resolution mass spectrum of **12b**.

Qualitative Analysis Report

**Data Filename**	UMK-1-280b 13.d	**Sample Name**	sample
**Sample Type**	Sample	**Position**	Vial 7
**Instrument Name**	Instrument 1	**User Name**	
**Acq Method**	without column.m	**Acquired Time**	3/17/2025 5:20:50 PM
**IRM Calibration Status**	Success	**DA Method**	111.m
**Comment**			

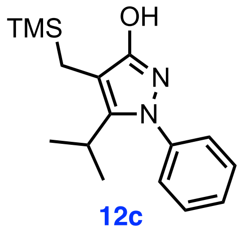

12c

**Method part to run:**	Acquisition Only	**Sample Group**	
**Info.**		**Stream Name**	LC 1
**Acquisition SW Version**	6200 series TOF/6500 series Q-TOF B.09.00 (B9044.0)		

User Chromatograms

Fragmentor Voltage 150 **Collision Energy** 0 **Ionization Mode** ESI

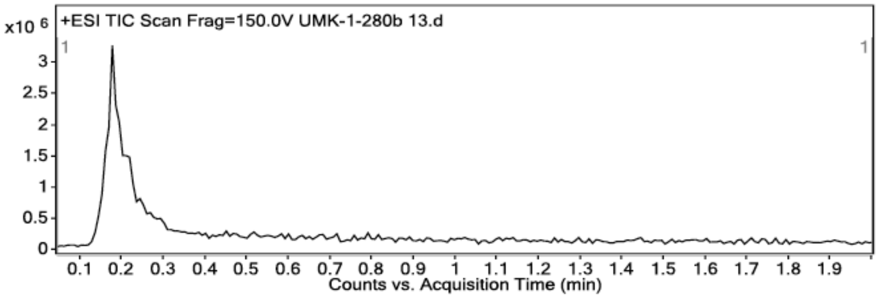

User Spectra

Fragmentor Voltage	Collision Energy	Ionization Mode
150	0	ESI

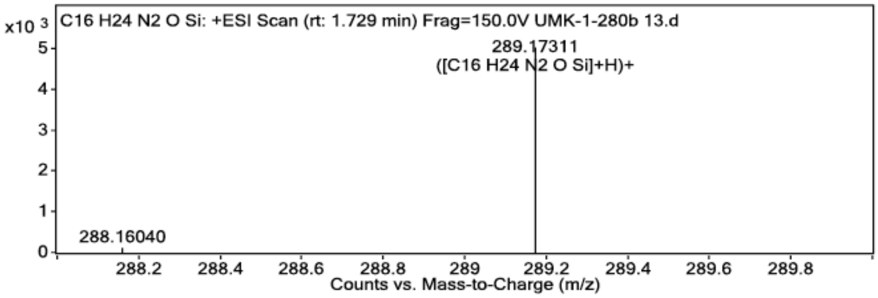

Peak List

m/z	z	Abund	Formula	Ion
273.13988	1	895.96		
274.14296	1	250.88		
287.15371		243.43		
288.1604		113.01		
289.17311	1	5027.09	C16 H24 N2 O Si	(M+H)+
290.17741	1	935.68	C16 H24 N2 O Si	(M+H)+
291.16769	1	250.07	C16 H24 N2 O Si	(M+H)+
325.22778	1	967.92		

Agilent Technologies   Page 1 of 2   Printed at: 5:29 PM on: 3/17/2025

Figure S109. High resolution mass spectrum of **12c**.

Qualitative Analysis Report

**Data Filename**	UMK-II-14b.d	**Sample Name**	UMK-II-14b
**Sample Type**	Sample	**Position**	P1-A3
**Instrument Name**	6530B LC Q-TOF	**User Name**	Hasan CAN (hcan)
**Acq Method**	ESI_Pos.m	**Acquired Time**	8/20/2025 5:57:35 PM (UTC+03:00)
**IRM Calibration Status**	Success	**DA Method**	hcan.m
**Comment**			

**Sample Group**		**Info.**	
**Stream Name**	LC 1	**Method Version**	2025-0326-1400-23939
**Override DA Method Version**		**Data File Version**	2025-0820-1457-31004
**Acquisition Workstation**	DESKTOP-L73MD3C	**DA Workstation**	DESKTOP-L73MD3C
**Acquisition Time (Local)**	8/20/2025 5:57:35 PM (UTC+03:00)	**Acquisition SW Version**	6200 series TOF/6500 series Q-TOF (11.0.203.0)
**QTOF Driver Version**	11.00.00	**QTOF Firmware Version**	15.851
**Tune Mass Range Max.**	3200		

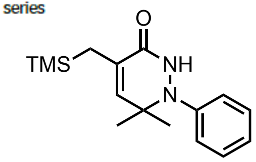

20a

Chromatograms

Fragmentor Voltage 90 **Collision Energy** 0 **Ionization Mode** ESI

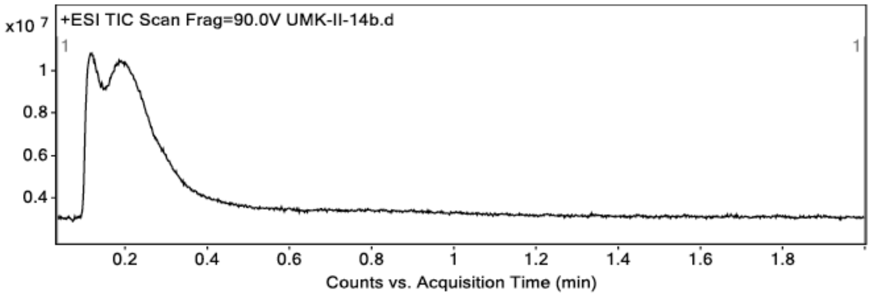

Spectra

Fragmentor Voltage	Collision Energy	Ionization Mode
90	0	ESI

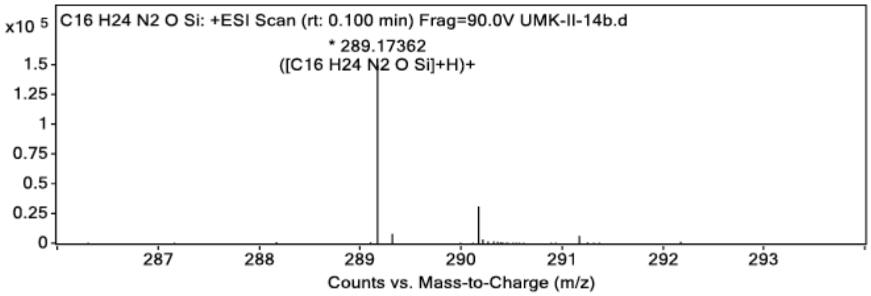

Peak List

m/z	z	Abund	Formula	Ion

Figure S110. High resolution mass spectrum of **20a**.

Qualitative Analysis Report

**Data Filename**	UMK-1-282f 01.d	**Sample Name**	sample
**Sample Type**	Sample	**Position**	Vial 1
**Instrument Name**	Instrument 1	**User Name**	
**Acq Method**	without column.m	**Acquired Time**	4/8/2025 2:07:32 PM
**IRM Calibration Status**	Success	**DA Method**	111.m
**Comment**			

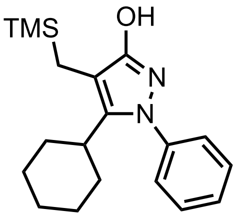

12d

**Method part to run:**	Acquisition Only	**Sample Group**	
**Info.**		**Stream Name**	LC 1
**Acquisition SW Version**	6200 series TOF/6500 series Q-TOF B.09.00 (B9044.0)		

User Chromatograms

Fragmentor Voltage 120 **Collision Energy** 0 **Ionization Mode** ESI

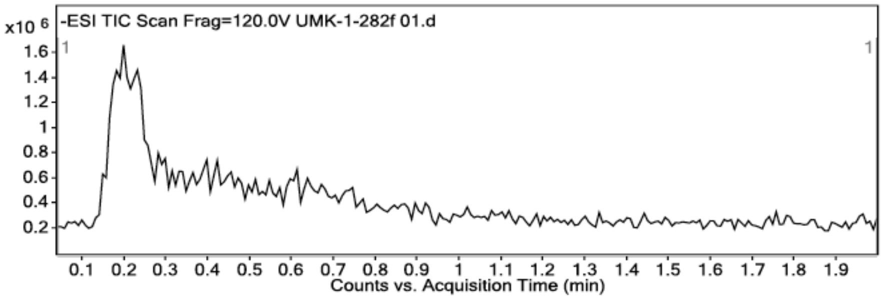

User Spectra

Fragmentor Voltage 120 **Collision Energy** 0 **Ionization Mode** ESI

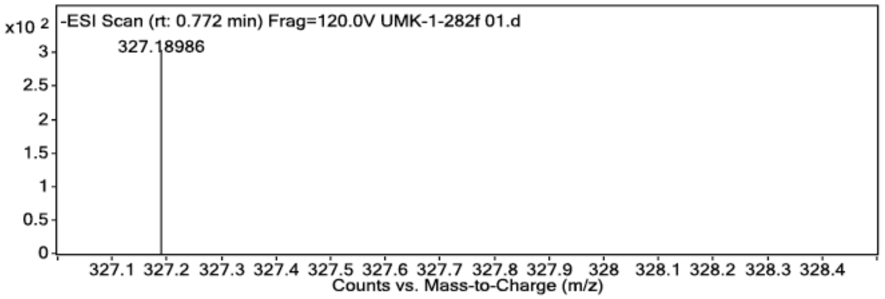

Peak List

m/z	z	Abund
61.98653		144.84
68.99146		207.71
98.95038		302.77
114.93459		136.77
119.93587		156.58
125.92784		185.45
129.01341		194.46
129.96775		127.05

Agilent Technologies   Page 1 of 3   Printed at: 2:19 PM on: 4/8/2025

Figure S111. High resolution mass spectrum of **12d**.

Qualitative Analysis Report

**Data Filename**	UMK-I-290d.d	**Sample Name**	UMK-I-290d
**Sample Type**	Sample	**Position**	P1-A2
**Instrument Name**	6530B LC Q-TOF	**User Name**	Hasan CAN (hcan)
**Acq Method**	ESI_Pos.m	**Acquired Time**	8/20/2025 5:54:51 PM (UTC+03:00)
**IRM Calibration Status**	Success	**DA Method**	hcan.m
**Comment**			

**Sample Group**		**Info.**	
**Stream Name**	LC 1	**Method Version**	2025-0326-1400-23939
**Override DA Method Version**		**Data File Version**	2025-0820-1454-46209
**Acquisition Workstation**	DESKTOP-L73MD3C	**DA Workstation**	DESKTOP-L73MD3C
**Acquisition Time (Local)**	8/20/2025 5:54:51 PM (UTC+03:00)	**Acquisition SW Version**	6200 series TOF/6500 series Q-TOF (11.0.203.0)
**QTOF Driver Version**	11.00.00	**QTOF Firmware Version**	15.851
**Tune Mass Range Max.**	3200		

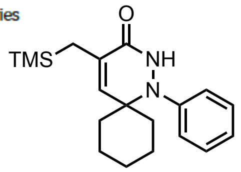

20b

Chromatograms

Fragmentor Voltage 90 **Collision Energy** 0 **Ionization Mode** ESI

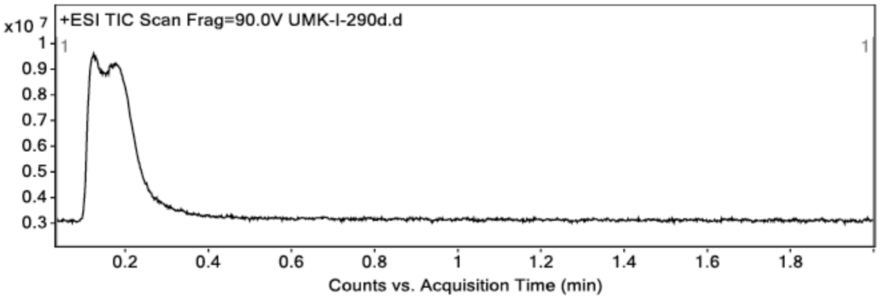

Spectra

**Fragmentor Voltage**	**Collision Energy**	**Ionization Mode**
90	0	ESI

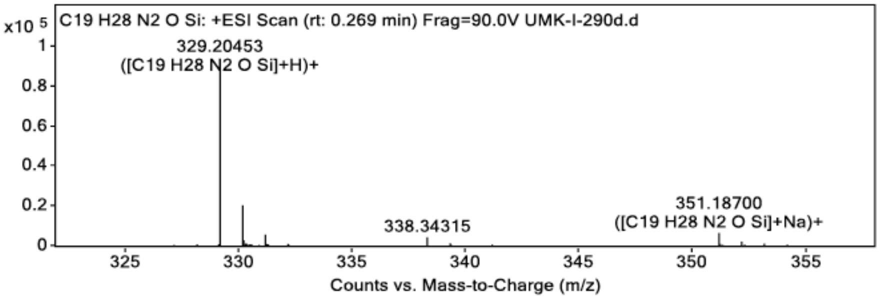

Peak List

m/z	z	Abund	Formula	Ion

Figure S112. High resolution mass spectrum of **20b**.

Qualitative Analysis Report

**Data Filename**	UMK-II-38b.d	**Sample Name**	UMK-II-38b
**Sample Type**	Sample	**Position**	P1-A5
**Instrument Name**	6530B LC Q-TOF	**User Name**	Hasan CAN (hcan)
**Acq Method**	ESI_Pos.m	**Acquired Time**	8/20/2025 6:03:04 PM (UTC+03:00)
**IRM Calibration Status**	Success	**DA Method**	hcan.m
**Comment**			

**Sample Group**		**Info.**	
**Stream Name**	LC 1	**Method Version**	2025-0326-1400-23939
**Override DA Method Version**		**Data File Version**	2025-0820-1502-59655
**Acquisition Workstation**	DESKTOP-L73MD3C	**DA Workstation**	DESKTOP-L73MD3C
**Acquisition Time (Local)**	8/20/2025 6:03:04 PM (UTC+03:00)	**Acquisition SW Version**	6200 series TOF/6500 series Q-TOF (11.0.203.0)
**QTOF Driver Version**	11.00.00	**QTOF Firmware Version**	15.851
**Tune Mass Range Max.**	3200		

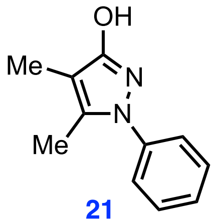

21

Chromatograms

Fragmentor Voltage 90 **Collision Energy** 0 **Ionization Mode** ESI

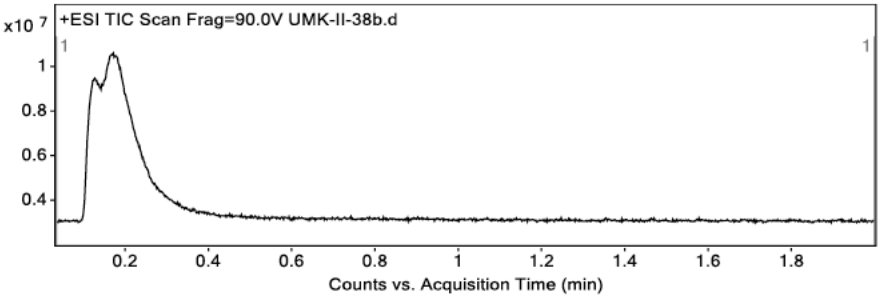

Spectra

Fragmentor Voltage	Collision Energy	Ionization Mode
90	0	ESI

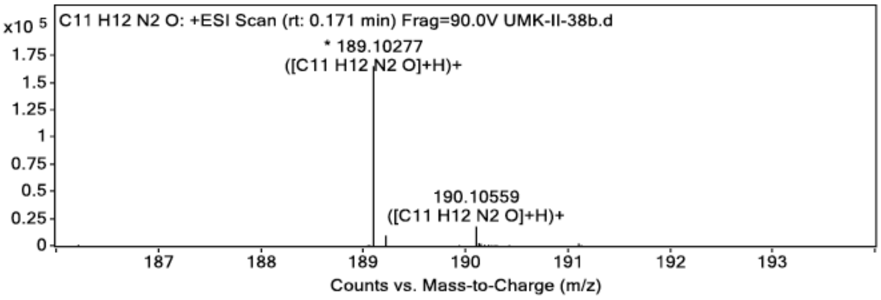

Peak List

m/z	z	Abund	Formula	Ion

Figure S113. High resolution mass spectrum of **21**.

Qualitative Analysis Report

**Data Filename**	UMK-1-234b 15.d	**Sample Name**	sample
**Sample Type**	Sample	**Position**	Vial 1
**Instrument Name**	Instrument 1	**User Name**	
**Acq Method**	without column.m	**Acquired Time**	1/10/2025 2:28:58 PM
**IRM Calibration Status**	Success	**DA Method**	111.m
**Comment**			

**Method part to run:**	Acquisition Only	**Sample Group**	
**Info.**		**Stream Name**	LC 1
**Acquisition SW Version**	6200 series TOF/6500 series Q-TOF B.09.00 (B9044.0)		

User Spectra

Fragmentor Voltage	Collision Energy	Ionization Mode
150	0	APCI

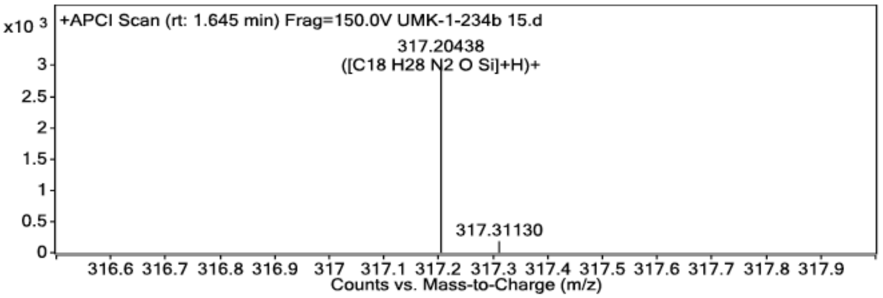

Peak List

m/z	z	Abund	Formula	Ion
130.15801		153.1	C8 H19 N	(M+H)+
244.25975		174.01		
284.1198		370.04		
288.29147	1	1495.72		
289.29209	1	323.37		
316.31806	1	588.47		
317.20438	1	3000.53	C18 H28 N2 O Si	(M+H)+
317.3113	1	182.28		
318.20837	1	713.18	C18 H28 N2 O Si	(M+H)+
319.21264	1	152.96	C18 H28 N2 O Si	(M+H)+
326.15651		488.4		
400.17066		244.11		
481.19501		212.24		
526.51803		162.56		
554.55177		187.01		

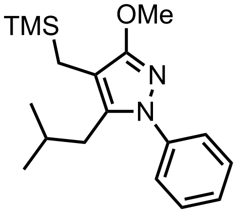

26

Formula Calculator Element Limits

Element	Min	Max
C	0	18
H	0	28
O	0	1
N	0	2
Si	0	1

Formula Calculator Results

Agilent Technologies   Page 1 of 2   Printed at: 2:39 PM on: 1/10/2025

Figure S114. High resolution mass spectrum of **26**.

Qualitative Analysis Report

**Data Filename**	UMK-I-312b.d	**Sample Name**	UMK-I-312b
**Sample Type**	Sample	**Position**	P1-A8
**Instrument Name**	6530B LC Q-TOF	**User Name**	Hasan CAN (hcan)
**Acq Method**	ESI_Pos.m	**Acquired Time**	8/20/2025 6:11:19 PM (UTC+03:00)
**IRM Calibration Status**	Success	**DA Method**	hcan.m
**Comment**			

**Sample Group**		**Info.**	
**Stream Name**	LC 1	**Method Version**	2025-0326-1400-23939
**Override DA Method Version**		**Data File Version**	2025-0820-1511-14695
**Acquisition Workstation**	DESKTOP-L73MD3C	**DA Workstation**	DESKTOP-L73MD3C
**Acquisition Time (Local)**	8/20/2025 6:11:19 PM (UTC+03:00)	**Acquisition SW Version**	6200 series TOF/6500 series Q-TOF (11.0.203.0)
**QTOF Driver Version**	11.00.00	**QTOF Firmware Version**	15.851
**Tune Mass Range Max.**	3200		

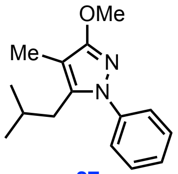

27

Chromatograms

Fragmentor Voltage 90 **Collision Energy** 0 **Ionization Mode** ESI

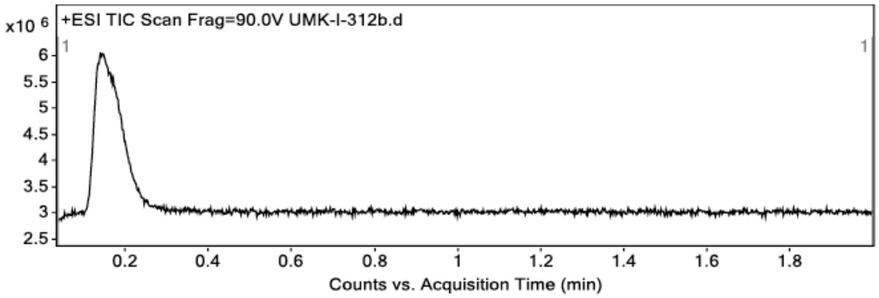

Spectra

Fragmentor Voltage 90 **Collision Energy** 0 **Ionization Mode** ESI

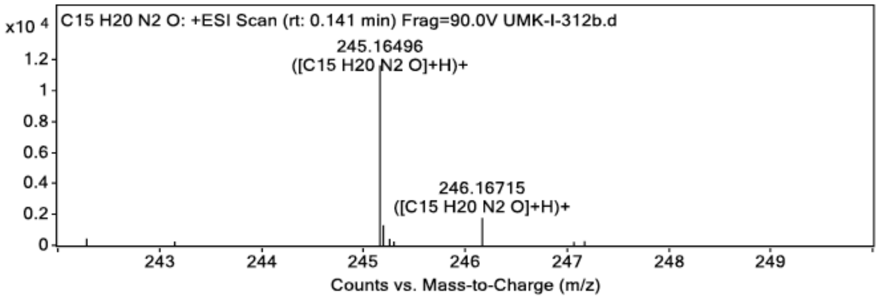

Peak List

m/z	z	Abund	Formula	Ion

Agilent Technologies   Page 1 of 2   Printed at: 10:47 AM on: 8/21/2025

Figure S115. High resolution mass spectrum of **27**.
